# Responses of New Zealand forest birds to management of introduced mammals

**DOI:** 10.1111/cobi.13456

**Published:** 2020-03-23

**Authors:** Nyree Fea, Wayne Linklater, Stephen Hartley

**Affiliations:** ^1^ Centre for Biodiversity and Restoration Ecology, School of Biological Sciences Victoria University of Wellington P.O. Box 600 Wellington 6140 New Zealand; ^2^ California State University Sacramento 6000 J Street Sacramento CA 95819 U.S.A

**Keywords:** bird populations, body size, cavity nesting, ecosystem management, endemism, invasive, mammalian predator, native biodiversity, predator control, threatened species, anidación en cavidades, biodiversidad nativa, control de depredadores, endemismo, especies amenazadas, invasivo, mamíferos depredadores, manejo de ecosistemas, poblaciones de aves, tamaño corporal, 最佳实践, 实验设计, 死亡率, 可再生能源, 采样偏差, 太阳能, 风能, 野生动物监测

## Abstract

Over the past 1000 years New Zealand has lost 40–50% of its bird species, and over half of these extinctions are attributable to predation by introduced mammals. Populations of many extant forest bird species continue to be depredated by mammals, especially rats, possums, and mustelids. The management history of New Zealand's forests over the past 50 years presents a unique opportunity because a varied program of mammalian predator control has created a replicated management experiment. We conducted a meta‐analysis of population‐level responses of forest birds to different levels of mammal control recorded across New Zealand. We collected data from 32 uniquely treated sites and 20 extant bird species representing a total of 247 population responses to 3 intensities of invasive mammal control (zero, low, and high). The treatments varied from eradication of invasive mammals via ground‐based techniques to periodic suppression of mammals via aerially sown toxin. We modeled population‐level responses of birds according to key life history attributes to determine the biological processes that influence species’ responses to management. Large endemic species, such as the Kaka (*Nestor meridionalis*) and New Zealand Pigeon (*Hemiphaga novaeseelandiae*), responded positively at the population level to mammal control in 61 of 77 cases for species ≥20 g compared with 31 positive responses from 78 cases for species <20 g. The Fantail (*Rhipidura fuliginosa*) and Grey Warbler (*Gerygone igata*), both shallow endemic species, and 4 nonendemic species (Blackbird [*Turdus merula*], Chaffinch [*Fringilla coelebs*], Dunnock [*Prunella modularis*], and Silvereye [*Zosterops lateralis*]) that arrived in New Zealand in the last 200 years tended to have slight negative or neutral responses to mammal control (59 of 77 cases). Our results suggest that large, deeply endemic forest birds, especially cavity nesters, are most at risk of further decline in the absence of mammal control and, conversely suggest that 6 species apparently tolerate the presence of invasive mammals and may be sensitive to competition from larger endemic birds.

## Abstract 2

*Article impact statement*: Management of introduced mammals across New Zealand has preferentially benefited large and endemic forest birds.

## Abstract (es)

Respuestas de Aves Habitantes de Bosques Neozelandeses al Manejo de Mamíferos Introducidos

**Resumen**

En los últimos 1000 años, Nueva Zelanda ha perdido entre el 40% y 50% de las especies de aves. De estas extinciones, más de la mitad se puede atribuir a la depredación por mamíferos introducidos. Las poblaciones existentes de aves de bosque todavía están siendo depredadas por mamíferos, principalmente ratas, zarigüeyas y mustélidos. La historia de manejo de los bosques en Nueva Zelanda durante los recientes 50 años representa una oportunidad única porque un programa variado de control de mamíferos depredadores ha creado un experimento de manejo replicado. Realizamos un metaanálisis de las respuestas a nivel poblacional de las aves de bosque ante diferentes niveles de control de mamíferos registrados en toda Nueva Zelanda. Recolectamos datos de 32 sitios tratados de manera única y de 20 especies existentes de aves que representan un total de 247 respuestas poblacionales a tres intensidades de control de mamíferos invasores (cero, bajo y alto). Los tratamientos variaron desde la erradicación de los mamíferos invasores por vía de técnicas terrestres hasta la eliminación periódica de los mamíferos por medio de toxinas dispersadas por vía aérea. Modelamos las respuestas a nivel poblacional de las aves de acuerdo con los atributos de historia de vida importantes para determinar los procesos biológicos que influyen sobre las respuestas de las especies al manejo. Las especies endémicas grandes, como la kaka (*Nestor meridionalis*) y la paloma de Nueva Zelanda (*Hemiphaga novaeseelandiae*), respondieron positivamente a nivel poblacional al control de mamíferos en 61 de los 77 casos para especies con un peso ≥20 g comparado con 31 respuestas positivas de 78 casos para especies con un peso <20 g. El ave cola de abanico (*Rhipidura fuliginosa*) y la curruca gris (*Gerygone igata*), ambas especies endémicas someras, junto con cuatro especies no endémicas (el mirlo [*Turdus merula*], el pinzón [*Fringilla coelebs*], el acentor [*Prunella modularis*] y el pájaro de anteojos [*Zosterops lateralis*]) que arribaron a Nueva Zelanda en los últimos 200 años presentaron una tendencia hacia las respuestas negativas o neutrales al control de mamíferos (59 de 77 casos). Nuestros resultados sugieren que las aves endémicas grandes y que habitan en las profundidades del bosque, especialmente aquellas que anidan en cavidades, tienen el mayor riesgo de vivir una declinación en el futuro si el control de mamíferos se ausenta. A la inversa, nuestros resultados también sugieren que seis especies aparentemente toleran la presencia de mamíferos invasores y podrían ser vulnerables a la competencia generada por aves endémicas de mayor tamaño.

## 摘要

不断增长的全球能源需求正在促进可再生能源作为化石燃料的替代能源的发展。然而, 可再生能源设施可能对野生动物产生不利影响。设施选址指南通常建议或要求项目开发者对施工前后的野生动物进行调查, 以预测风险并估计拟建项目的影响。尽管如此, 目前还没有已发表研究对野生动物调查类型的选择和设施内部、设施之间如何确定标准化调查类型做过定量分析。本研究评估了 628 篇同行评审文献、未发表报告及引文, 并分析了其中美国和加拿大的 525 篇文献或报告的数据 (涉及 203 个设施, 包含 193 个风能和 10 个太阳能设施), 以确定施工前后调查的频率及其随时间的变化;明确设计进行施工前后或影响控制分析的研究的频率; 施工前后收集的调查数据类型, 以及这些数据类型在不同时期和不同设施之间的标准化方法。在本研究的数据集中, 设施建设后的野生动物死亡率和栖息地利用的监测是常规标准做法 (446 份报告), 而在建设前对野生动物死亡率及栖息地利用的基线水平的评估较少 (84 份) 。 203 个设施中只有 22% (45 个) 同时提供了施工前和施工后的数据, 有 29% (59 个) 提供了实验研究设计。在进行了栖息地利用调查的 108 个设施中, 只有 3% 包含了动物发现概率的估计。因此, 现有数据普遍不能在不同建设时期和不同设施之间进行生物学数据的比较。我们建议使用实验研究设计并遵循类似的野外研究方法, 以提高对可再生能源影响野生动物的认识。 **【翻译: 胡怡思; 审校: 聂永刚】**

## Introduction

Introduced mammalian predators are responsible for over half of bird extinctions worldwide (Doherty et al. 2016); endemic species on islands are particularly vulnerable (Szabo et al. 2012; Bellard et al. 2016; Doherty et al. 2016). Predator‐suppression experiments are critical to the design of biodiversity conservation strategies, especially on islands that have been greatly affected by the introduction of mammalian predators (Simberloff 1995; Courchamp et al. 2003). Although individual bird species that benefit from predator suppression receive most research attention (e.g., Innes et al. 1999; Glen et al. 2012), the broader consequences of predator suppression on avifauna are less understood. Some species may benefit more than others and some may even be disadvantaged because, for example, the predators no longer suppress their competitors and thus a decline in densities of the subordinate species results (Mountainspring & Scott 1985).

The isolated islands that make up the New Zealand archipelago have undergone a Holocene extinction event, wherein 40–50% of native bird species have been lost since colonization by humans (Holdaway et al. 2001). Over half of these extinctions are attributed to invasive mammalian predators (Doherty et al. 2016). Many of the native bird species that remain in New Zealand forests continue to be threatened by predation from these mammals. Brushtail possums (*Trichosurus vulpecula*), ship rats (*Rattus rattus*), and stoats (*Mustela erminea*) are the primary agents of their ongoing decline (Innes et al. 2010).

New Zealand presents a unique opportunity for ecological study because a varied and sustained program of mammalian predator suppression over the past 50 years has created a broad‐scale, replicated management experiment with the potential to provide insight into the ecological forces structuring forest bird communities. Understanding the responses of birds to predator manipulations is particularly important in novel ecosystems, where modern‐day combinations of evolutionarily isolated native species and cosmopolitan invaders have arisen through deliberate or inadvertent human action (Hobbs et al. 2006), because the structuring and functioning of assemblages in these systems are not yet well understood.

Conservation sometimes aims to reduce densities of predatory invasive species to avoid extinctions of native species, with the ultimate aim of maintaining biodiversity at landscape scales while operating within budgetary constraints. Managers, therefore, require an understanding of the implications for native wildlife when different intensities and costs of management are implemented (Nichols & Williams 2006). In cases where the effects of invasive predators have been quantified, thresholds that signal the need for conservation action have been determined for a few New Zealand bird species. For example, effective protection of the Kokako (scientific names of bird species are in Table 1), a large endemic species, requires standardized abundance indices of ship rats and possums to be maintained below 1% (Innes et al. 1999). A negative relationship between nest success and ship rat abundance has been demonstrated for 2 small endemics, the North Island Robin (Armstrong et al. 2006) and the North Island Fantail (Fea & Hartley 2018). These types of density‐impact functions (i.e., relationship between the density of a pest and its impact on a valued resource) are useful for quantifying and predicting the impacts of mammalian predators on particular species (Norbury et al. 2015). Managers should also be equipped with an understanding of management outcomes on entire suites of species to more effectively, and efficiently, protect community assemblages. Because eradication and suppression of invasive mammals is planned over broader geographical scales (Courchamp et al. 2003; Russell et al. 2015), it is increasingly important that managers understand the broader effects on avian communities and the longer term implications of managing predators.

**Table 1:** New Zealand bird species included in analysis of responses of arboreal forest bird species to invasive mammal control

Common name*a*	Scientific name	Body mass (g)*b*	Endemism level	Cavity nests*c*	No mammal control*d*	Low mammal control*d*	High mammal control*d*	Threat category*e*
Pigeon	*Hemiphaga novaeseelandiae*	650.0	Genus	N	3	5	8	NT
NI Kaka	*Nestor meridionalis septentrionalis*	425.0	Family	Y	1	3	4	AR‐rec
SI Kaka	*Nestor meridionalis meridionalis*	500.0	Family	Y	3	1	1	T‐nv
NI Kokako	*Callaeas wilsoni*	220.0	Family	N	0	1	2	AR‐rec
Tui	*Prosthemadera novaeseelandiae*	90.0	Genus	N	3	8	8	NT
NI Saddleback	*Philesturnus rufusater*	70.0	Family	Y	0	0	1	AR‐rec
SI Saddleback	*Philesturnus carunculatus*	75.0	Family	Y	0	0	0	AR‐rec
RC Parakeet	*Cyanoramphus novaezelandiae*	70.0	Species	Y	1	3	4	AR‐rel
YC Parakeet	*Cyanoramphus auriceps*	40.0	Species	Y	1	1	1	NT
NI Robin	*Petroica longipes*	35.0	Species	N	0	2	5	AR‐dec
SI Robin	*Petroica australis*	35.0	Species	N	5	2	1	AR‐dec
Stitchbird	*Notiomystis cincta*	30.0	Family	Y	0	0	2	T‐nv
Bellbird	*Anthornis melanura*	26.0	Genus	N	3	6	7	NT
Yellowhead	*Mohoua ochrocephala*	25.0	Family*f*	Y	0	1	0	AR‐rec
Whitehead	*Mohoua albicilla*	14.5	Family*f*	N	1	5	5	AR‐dec
Brown creeper	*Mohoua novaeseelandiae*	13.0	Family*f*	N	2	1	1	NT
NI Tomtit	*Petroica macrocephala toitoi*	11.0	Species	N	2	8	7	NT
SI Tomtit	*Petroica macrocephala macrocephala*	11.0	Species	N	4	2	1	NT
NI Fantail	*Rhipidura fuliginosa placabilis*	8.0	Species	N	1	9	7	NT
SI Fantail	*Rhipidura fuliginosa fuliginosa*	8.0	Species	N	2	1	1	NT
NI Rifleman	*Acanthisitta chloris granti*	7.0	Family	Y	2	7	5	AR‐dec
SI Rifleman	*Acanthisitta chloris chloris*	7.0	Family	Y	4	1	1	NT
Grey warbler	*Gerygone igata*	6.5	Species	N *b*	4	8	8	NT
Blackbird	*Turdus merula*	90.0	Introduced	N	2	8	5	NE
Chaffinch	*Fringilla coelebs*	21.0	Introduced	N	2	8	5	NE
Dunnock	*Prunella modularis*	21.0	Introduced	N	1	1	3	NE
Silvereye	*Zosterops lateralis*	13.0	Introduced	N	2	8	5	NE

a Abbreviations: NI, North Island; SI, South Island; RC, Red‐crowned; YC, Yellow‐crowned.

b Ordered by average female body weight, largest to smallest, endemic species followed by nonendemic species.

c Obligate cavity nesters: Y, yes; N, no. Gray Warbler excluded from cavity‐nesting group because it suspends an enclosed nest from a branch.

d Number of studies recording a species’ response at the specified (no, low, and high) level of mammal control.

e Abbreviations: NT, not threatened; AR, at risk; T, threatened; rec, recovering; nv, nationally vulnerable; rel, relict; dec, declining) (Robertson et al. 2017); NE, nonendemic and arrived in New Zealand in last 200 years (Heather et al. 2015; Robertson et al. 2017).

f Mohoua species were assigned as endemic at family level (Aidala et al. 2013). For analysis, species in this genus were treated separately because they differ markedly in coloration and body weight.

Recognizing which life history attributes of birds increase their vulnerability to predation and decline can help one predict the likely outcomes of different management approaches. Previous investigations into extinctions of New Zealand forest bird species show that large, flightless birds that primarily nest on the ground have been especially vulnerable to extinction from hunting and introduced predators (Cassey 2001; Bromham et al. 2012). Cavity nesting is also associated with population declines of extant forest bird species in New Zealand (O'Donnell 1996; Parlato et al. 2015).

Deep endemism (i.e., belonging to an endemic family or higher taxonomic order) affects the risk of extinction for bird species across the world, including in New Zealand (Bennett & Owens 1997; Duncan & Blackburn 2004; Doherty et al. 2016). Walker et al. (2017) compared 2 periods of occupancy data for New Zealand forest birds (1969–1979 and 1999–2004) and concluded that deeply endemic forest bird species are undergoing the greatest range declines across the New Zealand mainland. Endemism is expected to have a causative relationship with vulnerability to introduced mammalian predators because it reflects the length of time bird species have evolved in the absence of a mammalian predator.

Removal of invasive predators is likely to generate long‐term positive responses for some native birds (Salo et al. 2007), and in New Zealand positive effects on forest bird populations have been observed (Innes et al. 2010; O'Donnell & Hoare 2012). A previous meta‐analysis of biodiversity outcomes showed a significant positive response for native New Zealand birds when possums were managed to low levels (Byrom et al. 2016). Evidence of this was based on survival outcomes measured at an individual level and from 6 studies in which bird populations were measured 1–2 years after the management intervention. Improved survival of individuals and short‐term population increases may not, however, always translate to population increases in the long term (Smith et al. 2010), and population responses may vary considerably among sites. A meta‐analysis of bird population responses to mammal control across 7 sites on South Island, for example, showed species’ responses are variable and there is no clear relationship between the direction or magnitude of a population response across species with shared life history traits (Hoare et al. 2013).

Large‐scale management of introduced mammals in New Zealand began in the 1950s and has since developed into a range of techniques from complete eradication inside fenced mainland islands to self‐resetting traps (Parkes et al. 2017). To date, there has been no comprehensive and quantitative meta‐analysis of the population responses of forest birds to the variety of mammalian predator suppression that has been carried out throughout New Zealand. We conducted a meta‐analysis of multiyear studies on forest bird species from sites across New Zealand to identify species that benefit the most or least when intensity of invasive mammal management is increased and, conversely, to identify species that are particularly vulnerable or robust in the absence of management. We further aimed to quantify how vulnerability may depend on the interaction between management and life history attributes that may influence predator–prey dynamics: body size, endemism, and cavity nesting.

## Methods

### Meta‐Analysis Scope

We searched online databases and published studies to identify potential projects for inclusion in the meta‐analysis. Information was taken from peer‐reviewed articles, online reports, and data summaries obtained directly from project managers. To be included a project had to have a description of the management regime at the site and results of bird population monitoring (means, sampling error, and sample sizes) for ≥1 bird species. Details of the resources and specific criteria used to identify potentially eligible projects are given in Supporting Information. Four types of study design were used within qualifying projects: treatments across time with monitoring across multiple years and no major change in management (T), comparisons of estimates before and after a change in management (BA), comparisons of a site subject to invasive mammal control (i.e., an impact) relative to a site not receiving this treatment (i.e., the control) (CI), and studies that combined before, after, control, and impact assessments (BACI) (Table 2).

**Table 2:** Biodiversity projects for which bird population responses were monitored, sufficient data were reported for a meta‐analysis, and management of mustelids, possums, and rats was described

ID*a*	Project name (treatment identifier)*b*	Control intensity*c*	Control type*c*	Control method*d*	First year	Last Year	Temporal extent (years)	Spatial extent (ha)	Years sampled (total)	Study design*e*	No. spp.*f*	*N* *g*	$\bar{X}$ *^h^*	*SE* *h*	Source*i*
1	Maungatautari	High	eMuPR	Ground	2002	2011	10	3,400	4	BA	7 (4)	Y	Y	Y	Fitzgerald & Innes unpublished Supporting Information
2	Maungatautari (reintroduced)	High	eMuPR	Ground	2008	2011	4	3,400	2	BA	3	Y	Y	Y	Fitzgerald & Innes unpublished Supporting Information
3	Zealandia	High	eMuPR	Ground	1995	2016	22	225	9	BA	4 (4)	Y	Y	Y	Miskelly 2018
4	Zealandia (reintroduced)	High	eMuPR	Ground	1995	2016	22	225	9	BA	7	Y	Y	Y	Miskelly 2018
5	Boundary Stream (MI)	High	hMuPR	Ground	1996	2006	11	800	11	T	4 (1)	Y	Y	Y	Ward‐Smith et al. unpublished Supporting Information
6	KMA (Rata Ridge)	High	hMuPR	Ground	2005	2005	1	850	1	CI	5	Y	Y	Y	Baber et al. 2009
7	Pureora (Waipapa)	High	hMuPR	GA	1978	1999	22	2,500	4	CI	11 (2)	Y	Y	Y	Smith & Westbrooke 2004
8	RNRP (MI)	High	hMuPR	Ground	1998	2011	14	825	14	BA	10 (2)	N*	Y	Y	Harper et al. unpublished Supporting Information
9	Rotopounamu	High	hMuPR	Ground	2010	2012	3	600	3	T	9 (1)	Y	Y	Y	McNickle unpublished Supporting Information
10	Te Urewera (MI)	High	hMuPR	Ground	1997	2011	15	586	15	T	12 (4)	Y	Y	Y	Moorcroft unpublished Supporting Information
11	Wainuiomata (MI)	High	hMuPR	GA	2005	2015	11	1,200	11	BA	8 (1)	N*	Y	Y	Crisp 2016 Supporting Information
12	Eglinton (Walker Creek)*j*	Low	lMuPR	Ground	2005	2009	5	100	5	T	1	Y	Y	Y	Greene & Pryde 2012
13	Hamilton	Low	lMuPR	Ground	2004	2012	9	1,000	5	BA	3 (3)	Y	Y	Y	Fitzgerald & Innes unpublished Supporting Information
14	RNRP (Lakehead)*j*	Low	lMuPR	Ground	1997	2011	15	4,000	15	BA	10 (2)	N*	Y	Y	Harper et al. unpublished Supporting Information
15	Rangitoto Island	Low	eP	GA	1990	1999	10	2,311	3	BA	4 (4)	Y	Y	Y	Spurr & Anderson 2004
16	Aorangi (Aorangi)	Low	pp3	GA	2013	2017	5	29,900	5	BA	9 (2)	Y	Y	Y	Fea unpublished Supporting Information
17	Tararua (Project Kaka)	Low	pp3	GA	2009	2012	3	22,000	3	BA	7	N*	Y	Y	Griffiths unpublished Supporting Information
18	Aorangi (Remutaka)	Low	pp6	Aerial	2014	2017	4	24,000	4	T	8 (3)	Y	Y	Y	Fea unpublished Supporting Information
19	Catlins*j*	Low	pp6	Aerial	1998	2002	5	4,750	5	BA	1	Y	Y	Y	Katzenberger & Ross 2017
20	Maungatautari (Pirongia)	Low	pp6	GA	2002	2011	10	16,862	4	BA	8 (2)	Y	Y	Y	Fitzgerald & Innes unpublished Supporting Information
21	Otago (Hampden)*j*	Low	pp6	Aerial	2005	2009	5	720	5	T	1	Y	Y	Y	Hamilton unpublished Supporting Information
22	Pureora (Waimanoa)	Low	pp6	Aerial	1978	1999	22	450	4	BACI	11 (2)	Y	Y	Y	Smith & Westbrooke 2004
23	Tararua (Hutt Catchment)	Low	pp6	Aerial	2009	2012	4	10,000	4	T	6 (1)	N*	Y	Y	Griffiths unpublished Supporting Information
24	Tongariro	Low	pp6	GA	2005	2012	8	20,000	8	T	9 (3)	Y	Y	Y	Guillotel unpublished Supporting Information
25	Boundary Stream (Cashes)	No	N	N	1996	2006	11	0	11	T	5	Y	Y	Y	Ward‐Smith et al. unpublished Supporting Information
26	Eglinton (Knobs Flat)*j*	No	N	N	2005	2009	5	0	5	T	1	Y	Y	Y	Greene & Pryde 2012
27	Kowhai Bush*j*	No	N	N	1976	2001	26	0	4	T	7 (4)	Y	Y	Y	Barnett 2011
28	Otago (Dunedin)*j*	No	N	N	2005	2009	5	0	5	T	1	Y	Y	Y	Hamilton unpublished Supporting Information
29	RNRP (NT)*j*	No	N	N	2003	2011	9	0	9	T	10 (2)	N*	Y	Y	Harper et al. unpublished Supporting Information
30	Tararua (NT)	No	N	N	2009	2012	4	0	4	T	6 (1)	N*	Y	Y	Griffiths unpublished Supporting Information
31	Waitutu (Poteriteri)*j*	No	N	N	2006	2010	5	0	5	T	6	Y	Y	Y	Greene et al. 2013
32	Waitutu (Waitutu)*j*	No	N	N	2006	2010	5	0	5	T	6	Y	Y	Y	Greene et al. 2013

a Unique project treatment number and location in Fig. 1.

b Abbreviations: MI, mainland island, reintroduction or reintroduction of nonresident species (edit may have changed meaning, but original was unclear) to the site; NT, no treatment.

c High, control included projects with eradication of mustelids, possums, and rats (eMuPR) and sites with high‐intensity mustelid, possum, and rat control (hMuPR); low, control included sites with low‐intensity mustelid or possum and rat control or both (lMuPR), eradication of possums but not mustelids or rats (eP), and periodic possum suppression performed every 2–4 years or every 5–8 years (pp3 or pp6, respectively); no, control included sites with no management of mustelids, possums, or rats (N).

d Ground, mammal control delivered via ground‐based methods (poisons laid in bait stations and lethal trapping); aerial, mammal control delivered via aerially sown 1080; GA, mammal control mixture of ground‐based methods and aerially sown toxins; N, no mammal control conducted at the site.

e Study design determined the type of comparison in the meta‐analysis: BA, before and after management; T, over time where no change in management occurred; CI, sites where treatment did (impact) and did not (control) occur.

f Total number of species included in analysis; nonendemic species in parentheses).

g Reported number of samples sizes: Y, metric reported; N*, metric not reported but data acquired through direct contact with managers.

h Mean and SE reported: Y, yes; N, no.

i Data sourced directly from project managers or from online reports are noted in column as Supporting Information and are available there.

j South Island sites.

Where taxa had closely related North Island and South Island species or subspecies, we combined taxa into a single nationwide group. Migratory species (e.g., cuckoos) were excluded. Responses of the New Zealand Falcon (*Falco novaeseelandiae*) and the New Zealand Kingfisher (*Todiramphus sanctus*) were rarely reported, so these native species were also excluded. Two projects combined records of 2 native nectarivores (Bellbird and Tui) under a common group name honeyeater. Because we could not assign these counts to either species, we excluded these data from the analysis. Other bird species not covered in this review were those that are flightless or call mostly at night (i.e., Morepork [*Ninox novaeseelandiae*] and Weka [*Gallirallus australis*]) and Kiwi (*Apteryx* spp.). We included 4 nonendemic species (Blackbird, Chaffinch, Dunnock, and Silvereye) that are common throughout New Zealand forests (Heather et al. 2015). The first 3 of these species were intentionally introduced to New Zealand from Europe from 1860 to 1885, and Silvereye self‐introduced from Australia or the South Pacific after 1830 (Heather et al. 2015).

### Intensity of Mammal Management

Management methods, including their temporal and spatial coverage, varied considerably across projects. We broadly grouped 7 management categories into 3 levels of management (Table 2). High‐intensity control included treatments to eradicate mustelids, possums, and rats (eMuPR) and sites with intensive spatial and temporal control of mustelids, possums, and rats (hMuPR). Eradication occurred in mainland forests surrounded by mammal exclusion fences. Ruffell et al. (2015) found that high‐intensity control significantly reduces indices of abundance for possums and rats, whereas less intense programs have variable, but generally low, success at reducing possum and rat indices. We followed the criteria of these authors for categorizing high‐intensity mustelid, possum, and rat control as treatments with poison bait stations targeting mustelids, possums, and rats placed at an average density of >1/1.5 ha; bait stations that were active over spring and summer; and replacement of bait at least every 12 weeks over spring and summer, when many bird species are nesting. Low‐intensity control included management of mustelids, possums, and rats (lMuPR), but not at the intensity described above; sites where eradication of possums occurred but not mustelids or rats (eP); and sites that received possum control every 2–4 years (pp3) or every 5–8 years (pp6). No control included sites without management of mustelids, possums, or rats (N).

Population data for 20 extant, diurnal, arboreal, forest bird taxa were collated (Table 2). Population responses were almost entirely estimated from standardized point counts (Dawson & Bull 1975), except for 1 study that involved counts along transects. In total, we collated 247 bird population responses (197 from endemic species and 50 from nonendemic species) from a total of 32 treatments for which criteria for inclusion in the meta‐analysis were satisfied. Fifteen of the treatments involved predominately ground‐based techniques (i.e., mammalian toxins delivered in bait stations and lethal trapping), although 3 of these sites received additional treatment via aerially sown sodium fluoroacetate. Nine treatments relied primarily on aerially sown toxin (i.e., pp3 and pp6) (Table 2), and 8 sites received no mammal control (Table 2). Eleven of the treatments were in South Island and 21 were in North Island or northern offshore islands (Fig. 1 & Table 2) (Supporting Information contains sources of unpublished data). Fifty‐five studies were not included in the meta‐analysis, but their details are in Supporting Information for completeness.

**Figure 1:**
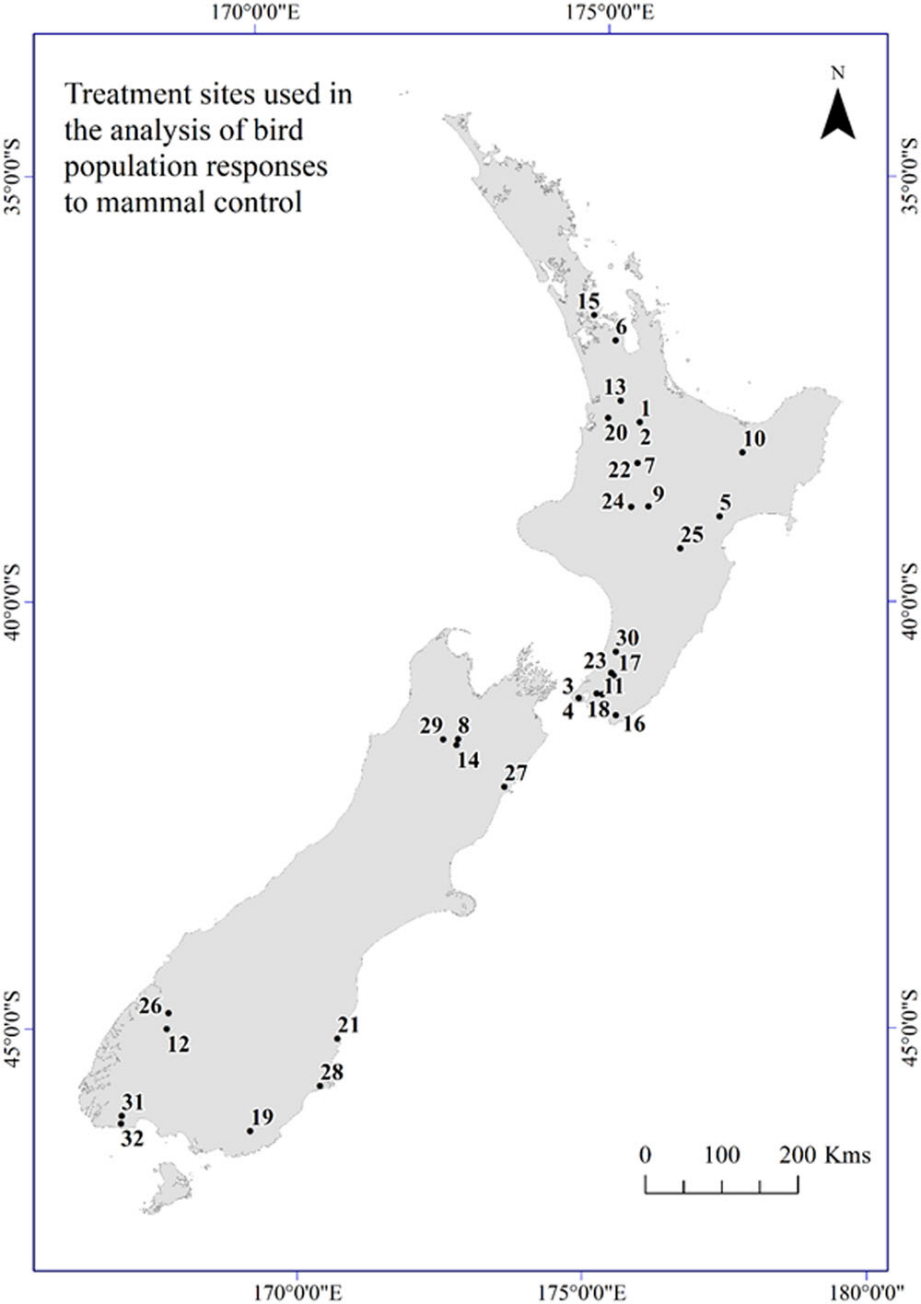
Locations in New Zealand of the uniquely treated sites in the biodiversity projects (names and details of treatments and the associated projects in Table 2).

### Analyses

We used the standardized mean difference (SMD) to quantify population responses to different intensities of mammal management for each bird species in our meta‐analysis. The SMD (also known as Hedges’ *G*) transforms all responses to a common metric (in SD units), which allows one to calculate responses of bird population within projects and summaries across projects (e.g., Dochy et al. 2003; Smith et al. 2010).

Using information on the mean, variance, and sample sizes of bird counts from within each treatment of each project, we calculated an SMD by comparing across time for the BA, BACI, and T study designs and across space for the control‐impact (CI) study design (Table 2).

For projects where bird species were reintroduced to a site (Zealandia and Maungatautari [Table 2]), the early period was chosen as the year or years immediately before reintroductions of the species occurred. Because reintroductions of the Stitchbird to Maungatautari continued into the late period (Doerr et al. 2017), the population response for this species therefore included recently released individuals. Population responses were not given for reintroduced species that were not encountered in the late period (Tomtit in Zealandia, Kaka in Maungatautari).

With temporal study designs we compared the first 1–2 years of data (early period) to the final 1–2 years of data (late period). We required a minimum gap of 1 year between the early and late periods. Where there was only a single year of pretreatment sampling, or where only 4 years of data were available, we used the first year only for the early period and the final 2 years of data for the late period.

For the 3 projects that had a control‐impact study design, we compared the single year of data from the impact site to the single year of data from the control (or reference) site or sites (Smith & Westbrooke 2004; Baber et al. 2009). For the Baber et al. (2009) project, because the 2 reference sites received similar management, we compared the treated site (Kokako Management Area) with the nearest reference site (Rata Ridge).

Where data were drawn from 2 years, weighted means and variances (incorporating within‐ and between‐year variance) were calculated using the method adapted from Hartley et al. (2006) (formulae in Supporting Information). Calculations of the SMD, and the associated uncertainty, were performed using the escalc function from the metafor package within the statistical computing software R (Viechtbauer 2010). Summary responses were estimated for each species at each control intensity, each level of endemism, and according to whether the species is an obligate cavity nester or not with univariate, random effects meta‐analysis (i.e., the RE model for subgroup, see Supporting Information).

We considered the influence of bird body mass (log_10_ average female body weight), cavity nesting (i.e., obligate cavity nesters, species that primarily nest in tree cavities), and endemism on the strength of native bird responses. Endemism included 3 levels: nonendemic, species that had arrived in New Zealand in the last 200 years; shallow endemics, taxa endemic to New Zealand at the level of species or genus; and deep endemics, taxa endemic at the level of family. Data on life history attributes (Table 1) were sourced from Heather et al. (2015) and Aidala et al. (2013).

The influence of life history attributes on SMDs was investigated via multivariate metaregression models. We included intensity of mammal control (3 levels, ordinal), bird body mass (log10 average female body weight, continuous), endemism (nonendemic, shallow endemic, deep endemic, and ordinal), and cavity nesting (yes or no, binomial) as moderators in a metaregression conducted with the 'rma.mv’ function in the metafor package in R (Viechtbauer 2010). To allow for the possibility of unordered responses, we included quadratic terms for the variables control intensity and level of endemism. Intensity of mammal control was included as a main effect and in interaction with body size, endemism, and cavity nesting. We included a fixed effect to investigate the influence of study design on population responses (i.e. T, BA, or CI). To test for the influence of the temporal or spatial scale of the treatment on population responses, we included the fixed effects number of years between SMD comparisons and size of the treated area. For random effects, we included treatment nested within project. To control for potentially confounding effects of phylogenetic relatedness (Bromham et al. 2012), we included a multilevel, nested variable for taxon (order, family, genus, species).

For each bird species, we described the strength of the linear association between population responses (SMD) and increased management effort with Pearson correlation coefficients. We checked for multicollinearity between variables with chi‐square tests of independence between 2 categorical factors, point biserial correlation between a dichotomous factor and a continuous variable, and analysis of variance (ANOVA) for associations between categorical factors and a continuous variable (all in R base package). Evidence of collinearity was set as Cramer's *V* coefficients or correlation coefficients (*r*) ≥ 0.70 (where *r* is also the square root of the coefficient of determination [*r* ^2^] from the linear ANOVA model). None of the life history variables were strongly correlated, although the variables endemism and cavity nesting were moderately correlated (*V* = 0.65, *p* < 0.00001, *n* = 247). Five of the 8 deeply endemic species were cavity nesters and zero out of 4 introduced species were cavity nesters. Both variables were nevertheless kept in the analyses because they measured different traits that are biologically distinct.

## Results

**Figure 2:**
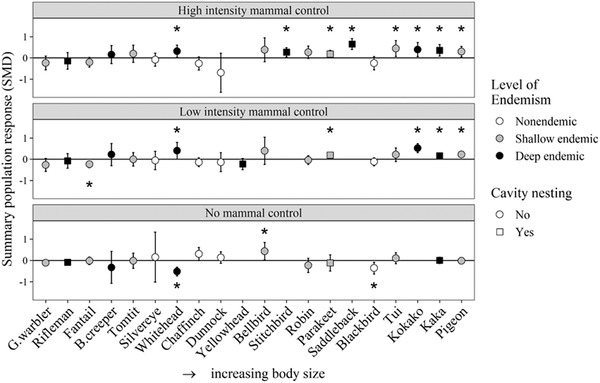
Forest‐bird population response to different intensities of invasive mammal control (summary standardized mean difference [SMD] and 95% CI, n = 247) in New Zealand. (G.warbler, Grey Warbler; B.creeper, Brown Creeper; *Significant responses according to 95% CIs that do not intercept 0 [above 0, positive population responses to the treatment; below 0, negative population responses]; nonendemic, species arrived in New Zealand within the last 200 years; shallow endemic, species endemic at level of species or genus; deep endemic, species endemic at the level of family). Bird species ordered by average female body weight.

Larger species responded positively to an increase in intensity of mammal control (meta‐regression, *β* of the linear interaction = 0.213, *p* < 0.0001) (Table 3). There were 61 positive population responses from 77 cases for species ≥20 g compared with 31 positive responses from 78 cases for species <20 g. Population responses to high‐intensity control were significantly positive for 7 out of 9 endemic bird species with a body weight ≥20 g (Kaka, Kokako, Parakeet, Pigeon, Saddleback, Stitchbird and Tui) and for 1 out of 6 small (<20 g) endemic species (Whitehead) (Fig. 2). Treatment‐specific responses that contributed to summary SMD responses are available in Supporting Information.

**Table 3:** Effect of life history attributes on population responses (standardized mean difference [SMD]) of New Zealand bird species to increased intensity of mammal control

Response variable	Effect	Variables*a*	*K* *b*	Coefficients*c*	LCI	UCI	*p*
SMD	Fixed	Intercept	247	−0.027	−0.565	0.510	0.921
		Mammal control		−0.251	−0.565	0.062	0.116
		Mammal control †		−0.065	−0.331	0.202	0.635
		Bird mass (log10)		0.147	−0.066	0.359	0.176
		Endemism		0.193	−0.097	0.484	0.192
		Endemism †		−0.032	−0.247	0.184	0.774
		Cavity nesting (no vs. yes)		−0.111	−0.473	0.252	0.550
		Temporal extent		−0.006	−0.030	0.017	0.596
		Spatial extent		0.000	0.000	0.000	0.582
		Study design (before‐after vs. control‐impact)		−0.107	−0.672	0.457	0.709
		Study design (before‐after vs. time series)		−0.082	−0.398	0.234	0.610
		Mammal control × bird mass		0.213	0.127	0.298	<0.0001^***^
		Mammal control † × bird mass		−0.040	−0.124	0.045	0.358
		Mammal control × endemism		0.393	0.161	0.625	0.001^***^
		Mammal control † × endemism		−0.290	−0.454	−0.125	0.001^***^
		Mammal control × endemism †		−0.009	−0.180	0.162	0.920
		Mammal control † × endemism †		−0.151	−0.258	−0.044	0.006^**^
		Mammal control × cavity nesting		−0.198	−0.389	−0.008	0.042^*^
		Mammal control † × cavity nesting		0.255	0.070	0.439	0.007^**^
			No. levels	Variance	SD		
	Random	Project	19	0.056	0.238		
		Treatment: project	32	0.008	0.089		
		Order	3	0.000	0.000		
		Family: order	15	0.000	0.000		
		Genus: family: order	17	0.000	0.000		
		Species: genus: family: order	20	0.061	0.247		
		Test for residual heterogeneity	QE	2840.200			<0.0001^***^
		Test of moderators	QM	83.511			<0.0001^***^

a Code: †, quadratic terms.

b Total number of bird population responses.

c Coefficients for SMD effect size estimated from multivariate, random effects meta‐analysis models.

*p ≤ 0.05; **p ≤ 0.01; ***p ≤ 0.001.

Endemism explained significant amounts of variation in species’ responses; 5 of the 7 deeply endemic species registered significantly positive responses to high‐intensity mammal control (Fig. 2). Seven bird species did not appear to benefit from high‐intensity control of invasive mammals (nonsignificant, negative summary SMDs). These included 1 deep endemic, the Rifleman; 2 common shallow endemics, the Fantail and Grey Warbler; and all 4 of the nonendemic species: Blackbird, Chaffinch, Dunnock, and Silvereye. The shallow and nonendemic species tended to have slight negative or neutral responses to mammal control (59 of 77 cases). The Fantail exhibited an overall significant negative response to low‐intensity management of mammals (SMD = −0.240, *p* = 0.003) (Fig. 2 & Supporting Information).

**Figure 3:**
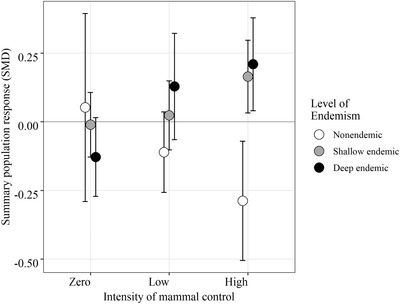
Population responses of New Zealand forest birds (summary standardized mean difference [SMD], 95% CIs, n = 247 population responses) to invasive mammal control across New Zealand relative to level of endemism (nonendemic, species arrived in New Zealand within the last 200 years; shallow endemic, species endemic at level of species or genus; deep endemic, species endemic at the level of family).

When responses were combined across species in the metaregression, we saw strong and significant effects for the interaction between control intensity and endemism and body size (Table 3). Generally, the deep endemic species and the relatively large shallow endemics had more positive relationships with increased management effort (Supporting Information) than small shallow and nonendemic species. The significant interaction between endemism and mammal control was mostly driven by contrasting population responses for nonendemic species and endemic species. Both shallow and deep endemic species switched from negative (nonsignificant) population trends in the absence of mammal control to increasingly positive responses with greater management effort. Introduced species showed a negative relationship with increasing intensity of mammal control (Fig. 3).

**Figure 4:**
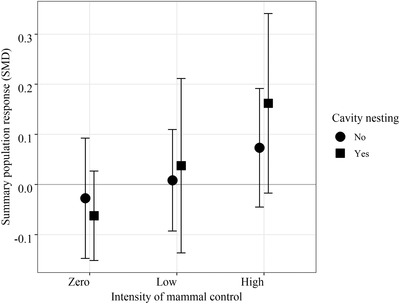
Population responses of New Zealand forest birds (summary standardized mean difference [SMD] responses, 95% CIs, n = 247 population responses) to invasive mammal control across New Zealand relative to whether a species nests in cavities or not.

Cavity nesting had a significant influence on species’ responses to mammal control (metaregression, *β* of the quadratic interaction = 0.255, *p* = 0.007) (Table 3); twice as many (4 vs. 2) cavity‐nesting species registered significant positive responses at sites receiving high‐intensity control than at sites receiving low‐intensity control (Fig. 2). However, responses were highly variable for the cavity nesters for all control intensities (Fig. 4), and sample sizes were limited by the absence of these species at managed and unmanaged sites.

At the level of individual taxa, only 2 species showed significantly positive correlations with increasing intensity of mammal control (the Kaka and Robin). For the 12 endemic species with sufficient numbers of population estimates to derive a correlation, 9 showed (mostly nonsignificant) positive correlations, whereas all 4 nonendemic species showed (nonsignificant) negative correlations with intensity of control. We were unable to estimate a correlation coefficient for Kokako, Saddleback, Stitchbird, and Yellowhead because of the paucity of population data for these 4 deeply endemic species (correlations in Supporting Information).

## Discussion

### Birds that Benefit

Body size influenced population responses of New Zealand forest birds; large endemic birds, on average, responded positively to predator suppression more often than small birds. Predation pressure from invasive mammals is likely to inhibit the population growth of large birds because this group typically has lower reproductive rates (smaller clutch sizes and fewer nesting attempts) and tends to live longer and reach reproductive age later than their smaller‐bodied counterparts (Cassey 2001).

Our results indicate that possum predation on large birds may be a significant problem because low‐intensity control of mammals, which is generally effective at controlling possums, but not mustelids and rodents (Ruffell et al. 2015), was associated with significant population increases for the 3 largest bird species in our analyses (Kaka, Kokako, and Pigeon [all >200 g]). Our findings also suggest that larger bird species are likely driving the positive response to possum control observed for native birds (Byrom et al. 2016).

We found clear differences in responses to mammal control between species that arrived recently (in the past 200 years) and species that were established in New Zealand before humans settled (shallow and deep endemics). Our results support findings from other studies that show species with the greatest evolutionary distinctiveness (i.e., family‐level endemics) are the ones most at risk when there is no mammal control (Doherty et al. 2016; Walker et al. 2017). Many of New Zealand's deeply endemic species have already been extirpated from large areas of their historical ranges (Holdaway 1989; Doherty et al. 2016), and in our study, 4 deeply endemic species were absent from unmanaged sites (Kokako, Saddleback, Stitchbird, and Yellowhead) and rarely detected at managed sites.

Although we had limited sample sizes for these species in our study, previous work demonstrates the vulnerability of these species to predation from introduced mammals (O'Donnell 1996; Innes et al. 1999; Basse et al. 2003; Dilks et al. 2003; Hooson & Jamieson 2003; Taylor et al. 2005; Toy et al. 2018), and these species have been brought close to extinction on New Zealand's mainland since the introduction of mammalian predators. The absence of these deeply endemic species across many sites in our meta‐analysis‐weakened our results because these species likely had the most negative responses at sites lacking mammal control and the most positive responses to high‐intensity mammal control. Therefore, the slope of the relationship between mammal control and endemism we found is necessarily conservative, based primarily on less vulnerable species that continue to persist.

Cavity nesting is a trait of 5 of the 8 deep endemic species and 1 of the shallow endemics. It explains significant amounts of variation in species’ responses to mammal suppression. At sites receiving high‐intensity mammal control, 4 of the 5 cavity‐nesting species registered significant positive responses, yet only 2 of 4 cavity‐nesting species responded positively to low‐intensity mammal control. The population responses for cavity‐nesting species we found need to be treated with caution because sample sizes were limited and the Stitchbird and Saddleback responses were based entirely on reintroduced populations. Such populations have the capacity to grow more rapidly than resident populations and may have been nurtured by supplementary feeding. Nonetheless, it is likely that cavity nesting continues to be a risky behavior for endemic birds in New Zealand, as confirmed by the severity of range contractions for cavity‐nesting species across the mainland (Parlato et al. 2015). Compared with noncavity‐nesting species of similar size, obligate cavity nesters tend to have longer nest periods (Heather et al. 2015) and are therefore particularly vulnerable to predation by mammals while on the nest. Management at sites where cavity nesters occur should continue to aim for intensive mammal suppression to maintain cavity‐nesting species in those landscapes and provide refugia (e.g., artificial nesting cavities) that limit predator access (Briskie et al. 2014).

The significant effects of body size, endemism, and cavity nesting in our meta‐analysis is consistent with outcomes achieved in other projects where bird populations were monitored after mammal control (but were not eligible for inclusion in our meta‐analysis). For example, in the only published New Zealand study that employed a BACI project design and high‐intensity mammal control, Innes et al. (2004) found that the 2 largest species in the community were the only endemic species to respond positively to management (Pigeon and Tui). The large or deeply endemic bird species in a 12‐year study on South Island (O'Donnell & Hoare 2012) generally had the strongest positive responses to mammal control (Bellbird, Brown Creeper, Parakeet, Rifleman, Tui, and Yellowhead). An exception was the response of the largest deep endemic in this study, the Kaka, that did not appear to benefit from the high‐intensity mammal control at this site. The responses of the shallow endemics were either slightly positive (Fantail and Grey Warbler) or slightly negative (Tomtit), and 3 of the 4 nonendemic species in our study (Blackbird, Chaffinch and Silvereye) showed significantly negative trends in this South Island study.

High‐intensity management is generally only scalable to a few thousand hectares. Low‐intensity control characterizes the majority of mammalian predator control across New Zealand as a whole; 37–45% of New Zealand's total land area (10–12 million ha) is under some form of sustained possum control (Russell et al. 2015; Byrom et al. 2016; Parkes et al. 2017) and <1% receives high‐intensity mammal control through bait stations. For native forest habitat that is formally protected, 17% receives sustained possum control (calculations in Supporting Information). Therefore, the responses of bird populations to low‐intensity mammal control, as we defined it here, are most applicable for interpreting and predicting bird population responses to control across most of New Zealand's managed forests. For ongoing protection of the most vulnerable species in these remote forests, ecologically informed strategies that align with the Department of Conservation's current practice should be maintained, for example, intensification of predator control following heavy seed fall to prevent population irruptions of the mammalian predators and ensure greater nesting success for endemic bird species (Basse et al. 2003; O'Donnell & Hoare 2012; Elliott & Kemp 2016).

### Birds that Do Not Benefit

High‐intensity mammal control, such as eradication of mammals from islands, is often regarded as the gold standard for conservation of New Zealand birds. It is interesting to note, therefore, that there were 7 species whose variable responses did not equate to an overall positive response to high‐intensity mammal control. One of these is a deeply endemic, small‐bodied species that nests in cavities (Rifleman). It is puzzling that the overall responses of the Rifleman were not positive as they were for the other deeply endemic cavity nesters (but see discussion on insectivore competition below). They are known to be sensitive to mammal predation on the nest, as is their alpine relative, the Rock Wren (*Xenicus gilviventris*), and mammal control can improve nesting success for these 2 species (Elliott & Kemp 2016). These 2 wrens are the last surviving members of the ancient Acanthisittidae family and further research is required to understand how management might best protect these declining endemics (Robertson et al. 2017; Walker & Monks 2018).

Other species that did not exhibit an overall positive response to management included 2 small shallow endemics, Fantail and Grey Warbler, plus 4 species that arrived in New Zealand in the last 200 years (Blackbird, Chaffinch, Dunnock, and Silvereye). Our results suggest that where high‐intensity control reduces populations of invasive mammals, large‐bodied endemic bird species recover, and populations of these opportunistic, contemporarily common species tend to slight negative or neutral responses. We agree with Miskelly (2018) that these shallow endemics and recently arrived species are likely to be more robust in the presence of invasive mammals than other New Zealand endemic birds because they have retained mammal‐adapted behaviors. For example, a resilient feature of the Fantail is its habit of placing nests on thin branches making them less accessible to mammalian predators (Fea & Hartley 2018).

Size‐based competitive hierarchies are common place (e.g. within avian feeding guilds, such as honeyeaters [Ford 1979] and scavengers [Wallace & Temple 1987]), and we suspect competition from recovered populations of large birds may explain why small‐bodied species do not necessarily increase in detections after mammal control in New Zealand. The smallest endemic forest species (Fantail, Grey Warbler, Rifleman, and Tomtit) are almost entirely insectivorous (Heather et al. 2015). Competition from large generalist birds that also include insects in their diet (e.g., Bellbird, Saddleback, Stitchbird, Tui, and Whitehead) could limit access for the small insectivorous species to patches of high‐quality habitat (Craig 1985). Size‐based competitive suppression is consistent with the observation that small‐bodied bird species are difficult to reintroduce into mammal‐free sanctuaries, especially if large birds have already become established (Empson & Fastier 2013).

Our main objective was to investigate patterns in the population‐level responses of avian assemblages at national scales. Our meta‐analysis of 32 treatments undertaken over a wide geographic scale and across 5 decades showed that control of mammalian predators typically benefits large‐bodied New Zealand birds, obligate cavity nesters, and deep endemic species. The corollary is that similar responses by most of the small endemic species were not detected and one, Fantail, showed a significant negative response to low‐intensity mammal predator control.

### Limitations and Recommendations

A key result from our meta‐analysis was that the responses of New Zealand birds to mammal control varied. This variability will not only be attributed to site‐specific biological factors, such as the initial densities of the animal populations (birds and introduced predators) prior to the commencement of management and the structure and composition of the forests, but also to environmental factors such as elevation and climate. There is also great variety in the design of management projects and the associated monitoring programs. Although we incorporated variables in our models to account for key differences across management programs (i.e., spatial and temporal extent of the management program and experimental design of the monitoring program), there remained significant heterogeneity between treatments in our meta‐analysis (Table 3) that is worth investigating further.

Our estimates of bird population responses were consistently derived from bird count data, especially the 5‐min bird count, which is the most common method used across New Zealand to monitor trends in bird populations (Hartley 2012). Where conspicuousness of New Zealand bird species has been studied alongside other measures of relative abundance or density, the relationship is generally proportional (Gill 1980; Innes et al. 2004; Katzenberger & Ross 2017). These studies suggest that overall, bird‐song detections generally have a strong monotonic relationship with underlying density.

An advantage of conducting a review of outcomes from projects that record data on multiple species is the reduced occurrence of the file‐drawer effect (i.e., the tendency for nonsignificant results to go unreported or unpublished [Rosenthal 1979]). Even when the primary focus of a project was a very rare species, studies often reported outcomes of other species for comparison. Furthermore, we used data obtained from 5 unpublished data sets, which further reduces publication bias in our study.

Interpretation of the results in this meta‐analysis required consideration of the differences in population responses of extant (*n* = 237) and reintroduced populations (*n* = 10). For reintroduced populations, the response is a comparison of the early period, when the species was entirely absent from the site, with a late period, when the species occurs. Therefore, the response can only be positive. That said, reintroduced populations are not so different from extant populations that persist in very low numbers at a site (e.g., Pigeon present occasionally during the precontrol counts in Zealandia [Miskelly 2018] and Kaka absent from initial counts in the Rotoiti Nature Recovery Project [project 8 in Table 2]).

Our meta‐analysis included a large sample of published and unpublished data; however, the data set was not balanced. Although there were similar numbers of treatments from each category for intensity of mammal control (11, 13, and 8 treatments for high‐intensity, low‐intensity, and zero mammal control, respectively), the number of population responses from the high‐ and low‐intensity treatments was over twice that of sites lacking mammal control (98, 100, and 49 respectively). Furthermore, of the 11 studies identified as having high‐intensity control, only 1 was on South Island, and 6 of the 8 projects that lacked mammal control are on South Island. Few of the projects we included used the more scientifically robust BACI experimental design (Underwood 1991).

We urge conservation researchers, practitioners, and biodiversity managers to consider these possible sources of bias when developing future avian monitoring programs. In particular, there is a need for more BACI studies of bird populations at sites on South Island. Better experimental designs and additional studies in other regions may strengthen our inferences about the species‐ and assemblage‐wide responses of birds to suppression of introduced mammalian predators, increasing confidence in our conclusions about the positive responses of large‐bodied, deep endemic, and cavity‐nesting species and may address remaining uncertainties about the mechanisms underlying responses of small‐bodied endemics and reintroduced populations. As mammalian predator control is intensified and expanded over larger areas, work to improve the ability to predict community‐level responses of the avian assemblage to particular management regimes is increasingly required.

## Supporting information

Further details, such as resources and databases used in the search for eligible projects (Appendix S1), initial criteria for identifying potentially eligible projects for the meta‐analysis (Appendix S2), methods for aggregated population estimates (Appendix S3), projects that involved population monitoring of birds over multiple years and included descriptions of management treatments (Appendix S4), Pearson correlation results between species’ responses and intensity of mammal control (Appendix S5), treatment‐specific responses that contribute to the summary SMDs in Fig. 2 (Appendix S6), species‐specific responses across different management intensities (Appendix S7), correlations between responses of bird species and control intensity according to body mass and level of endemism (Appendix S8), sources of data cited in Table 2 (Appendices S9‐S19), and current coverage of mammal control across New Zealand (Appendix S20), are available online. The authors are solely responsible for the content and functionality of these materials. Queries (other than absence of the material) should be directed to the corresponding author.

Supporting Material

Supporting Material

Supporting Material

Supporting Material

Supporting Material

Supporting Material

Supporting Material

Supporting Material

Supporting Material

Supporting Material

Supporting Material

Supporting Material
