# Supplementary material for: Responses of New Zealand forest birds to management of introduced mammals
Source: Conserv Biol. 2020 Mar 23;35(1):35–49. doi: 10.1111/cobi.13456 (PMC7984369; doi:10.1111/cobi.13456)
Supplement: Supplementary file 1 — Further details, such as resources and databases used in the search for eligible projects (Appendix S1), initial criteria for identifying potentially eligible projects for the meta‐analysis (Appendix S2), methods for aggregated population estimates (Appendix S3), projects that involved population monitoring of birds over multiple years and included descriptions of management treatments (Appendix S4), Pearson correlation results between species’ responses and intensity of mammal control (Appendix S5), treatment‐specific responses that contribute to the summary SMDs in Fig. 2 (Appendix S6), species‐specific responses across different management intensities (Appendix S7), correlations between responses of bird species and control intensity according to body mass and level of endemism (Appendix S8), sources of data cited in Table 2 (Appendices S9‐S19), and current coverage of mammal control across New Zealand (Appendix S20), are available online. The authors are solely responsible for the content and functionality of these materials. Queries (other than absence of the material) should be directed to the corresponding author. [file COBI-35-35-s010.docx]

**Appendix S1.** List of resources and databases used in the data search for responses of bird populations to invasive mammal control. We used the terms “New Zealand bird population”, “New Zealand bird count”, “New Zealand Mainland Island” and known reserve names as the key search phrases for our database searches. Where possible the search contained all terms. Terms within categories were linked with the Boolean operator “OR” and terms between categories were linked with the Boolean operator “AND”. Below is a list of sources for results from New Zealand bird population studies. Data searching occurred between 01 January 2015 and 01 July 2017.

| 1. Bibliographies from (Byrom et al. 2016), Innes et al. (2010) and Smith et al. (2010) to identify projects |
| --- |
| 1. Database managers of government and community led projects to request unpublished reports / data summaries |
| 1. Web of Knowledge |
| 1. Google Scholar |
| 1. Biological Sciences’ (VUW library database) |
| 1. Biosis previews |
| 1. ProQuest database (<http://search.proquest.com/pqdtglobal/index>) |
| 1. VUW Research Archive (VUW library database: <http://researcharchive.vuw.ac.nz/>) |
| 1. NZ Research (VUW library database: <http://nzresearch.org.nz/>) |
| 1. Science Direct |
| 1. Directory of Open Access Journals |
| 1. Scopus |

**Literature Cited**

Byrom AE, Innes J, Binny RN. 2016. A review of biodiversity outcomes from possum-focused pest control in New Zealand. Wildlife Research **43**:228-253.

Innes J, Kelly D, Overton J, Gillies C. 2010. Predation and other factors currently limiting New Zealand forest birds. New Zealand Journal of Ecology **34**:86-114.

Smith RK, Pullin AS, Stewart GB, Sutherland WJ. 2010. Effectiveness of Predator Removal for Enhancing Bird Populations. Conservation Biology **24**:820-829.
