# Supplementary material for: Responses of New Zealand forest birds to management of introduced mammals
Source: Conserv Biol. 2020 Mar 23;35(1):35–49. doi: 10.1111/cobi.13456 (PMC7984369; doi:10.1111/cobi.13456)
Supplement: Supplementary file 2 — Supporting Material [file COBI-35-35-s001.docx]

**Appendix S2.** Initial criteria for selection of a project where bird population responses were reported over multiple years, and where management of introduced mammalian predators (namely stoats (*Mustela erminea*), possums (*Trichosurus vulpecula*), and ship rats (*Rattus rattus*)) was described.

| 1. Annual sampling through either transect and point count surveys of bird detections (i.e. birds seen and heard) or re-sighting of marked adults. |
| --- |
| 1. Where two uniquely treated sites shared boundaries (e.g. the Wainuiomata-Reference site and the Wainuiomata Mainland Island site; or the Wellington City site and the Zealandia site), the site receiving the highest intensity treatment only was included in the meta-analysis. |
| 1. We included time series or ’before’/’after’ studies spanning a minimum of three years. |
| 1. Where multiple seasons were counted, choice of season followed this order: Spring-Summer-Autumn-Winter. |
| 1. Projects that compared counts across different seasons were not included, as this violated the assumption for constant detectability across counts. |
| 1. Reporting of the means, variance and sample sizes of bird count data, and description of the intensity of mammal management at the site. |
| 1. Where bird population data from the same site were presented in two different publications with no change in treatment at the site, the results across the longest time series were used. |
| 1. Where population monitoring was carried out at the same site and was presented in two different publications, if the studies monitored different time frames, or different treatments then both were included. |
| 1. For data that were only available in published figures, we used GetData Graph Digitizer software (http://www.getdata-graph-digitizer.com/; accessed January 2017) to digitise means and variance. |

**Appendix S3.** Methodology for aggregating estimates of sample size, mean and variance across the two years within each period used for calculations of the standardised mean difference.

| Weighted mean for the two year period = ($w_{1}\bar{x}_{1}$) + ($w_{2}\bar{x}_{2}$) [Eqn 1]  Where, $\bar{x}_{i}$ = mean number of detections in year *i*  and, $w_{i}$ = weight assigned to year *i*,  according to its proportional sample size: *w_i_ = n_i_*/ *N*  *N =* ${n_{1}+ n}_{2}$  Where*, N* = the sum of sample sizes across the two years within a period  and*, n_i_* = the number of independent bird counts from a single year.  Pooled variation within years = $\left( w_{1} s_{1}^{2} \right)+\left( w_{2} s_{2}^{2} \right)$ [Eqn 2]  Where, $s_{i}^{2}$ = variance of the mean in year *i*.  Variation between years = $((w_{1} (\bar{x}_{1})^{2})+{(w}_{2} (\bar{x}_{2})^{2}))-\left( w_{1}\bar{x}_{1}+w_{2}\bar{x}_{2} \right)^{2}$ [Eqn 3]  Total variance for the two-year period = Eqn 2 + Eqn 3 |
| --- |

**Appendix S5.** The relationship of bird population responses to increased intensity of control. Correlation coefficients (*r_p_*) are estimated using Pearson correlation tests for association between ordered variables. Taxa with North Island and South Island (sub-) species are combined, except for the yellowhead, whitehead and brown creeper (all members of the *Mohoua* genus) which differ in colouration and body weight. Key to terms: *n* = number of treatments; NA - refers to species with insufficient detections to conduct the test. **· =** *P* ≤ 0.10, * = *P* ≤ 0.05. Bird species are ordered by average female body weight, largest - smallest, with non-endemic species (the blackbird, chaffinch, dunnock and silvereye) after endemic species. See Fig. 3 for a graphical representation of this data.

| **Species** | ***n*** | ***r_p_*** | ***P*-value** |  |
| --- | --- | --- | --- | --- |
| Pigeon | 16 | 0.402 | 0.123 |  |
| Kaka | 13 | 0.564 | 0.045 | * |
| Kokako | 3 | NA | NA |  |
| Tui | 19 | 0.325 | 0.175 |  |
| Saddleback | 1 | NA | NA |  |
| Parakeet | 11 | 0.462 | 0.153 |  |
| Robin | 15 | 0.534 | 0.040 | * |
| Stitchbird | 2 | NA | NA |  |
| Bellbird | 16 | -0.030 | 0.913 |  |
| Yellowhead | 1 | NA | NA |  |
| Whitehead | 11 | 0.311 | 0.351 |  |
| Brown creeper | 4 | 0.586 | 0.414 |  |
| Tomtit | 24 | 0.219 | 0.304 |  |
| Fantail | 21 | -0.091 | 0.694 |  |
| Rifleman | 20 | -0.042 | 0.859 |  |
| Grey warbler | 20 | -0.099 | 0.677 |  |
| Blackbird | 15 | -0.043 | 0.878 |  |
| Chaffinch | 15 | -0.454 | 0.090 | **·** |
| Dunnock | 5 | -0.556 | 0.331 |  |
| Silvereye | 15 | -0.137 | 0.626 |  |

**Appendix S6a.** The effect of *high* intensity mammal control on *endemic* bird species from a meta-analysis using the standardised mean difference. Presentation conventions as described for Figure S1f.

**Appendix S6b.** The effect of *low* intensity mammal control on *endemic* bird species from a meta-analysis using the standardised mean difference. Presentation conventions as described for Figure S1f.

**Appendix S6c.** Population trends for *endemic* bird species in the absence of mammal control from a meta-analysis using the standardised mean difference. Presentation conventions as described for Figure S1f.

**Appendix S6d.** The effect of *high* intensity control on *non-endemic* bird species from a meta-analysis using the standardised mean difference. Presentation conventions as described for Figure S1f.

**Appendix S6e.** The effect of *low* intensity control on *non-endemic* bird species from a meta-analysis using the standardised mean difference. Presentation conventions as described for Figure S1f.

**Appendix S6f.** The effect of *no* control on *non-endemic* bird species from a meta-analysis using the standardised mean difference. Effect sizes (and 95% confidence intervals) are presented: separately for each project; within a summary random effect model for that species (i.e. RE model for Subgroup); and as an effect size for all non-endemic birds across all studies lacking control (RE Model for All Studies). Negative effect sizes indicate that bird populations declined on average in the absence of invasive mammal control. An effect size is significantly different from zero when the confidence intervals do not overlap zero. Species are ordered from the top by descending weight.

**Appendix S6.** Treatment-specific and summary population responses for bird species in New Zealand derived using standardised mean difference effect sizes.


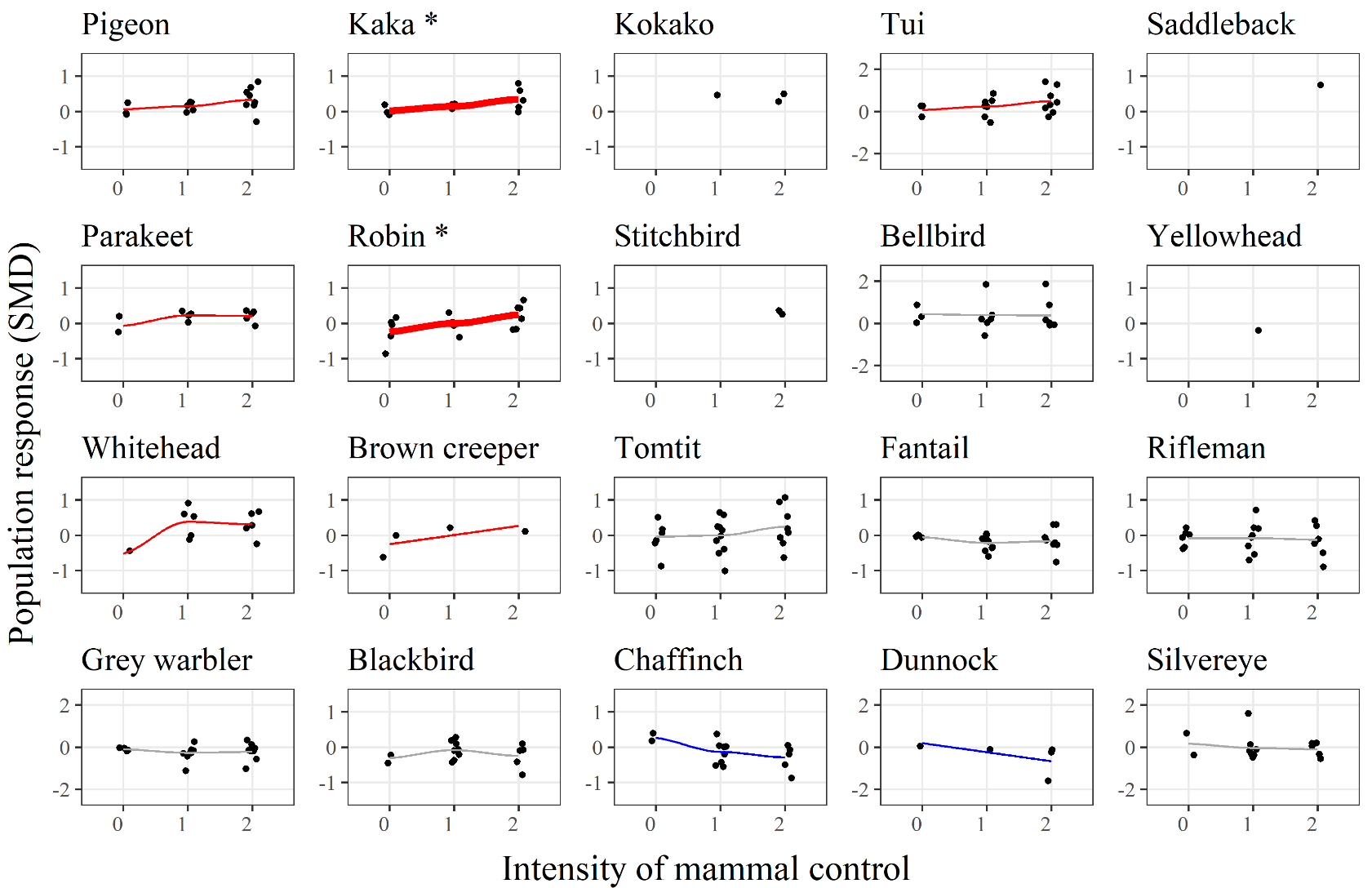


**Appendix S7.** Species specific responses (standardised mean difference (SMD)) across the three mammal control intensities. Control intensity: 0 = no mammal control, 1 = low intensity mammal control, 2 = high intensity mammal control. Trend lines indicate the general trend, colour-coded red when Pearson’s *r* > +0.3, blue when *r* < -0.3 and grey when 0.3 > *r* ≥ -0.3. * and bold lines = *P* ≤ 0.05 according to Pearson correlation tests (see Table S5 for these results). Trends were not plotted where there were ≤ 3 population responses. Panels ordered according to average female body mass of endemic species, from heaviest to lightest, followed by the four non-endemic species, from heaviest to lightest.


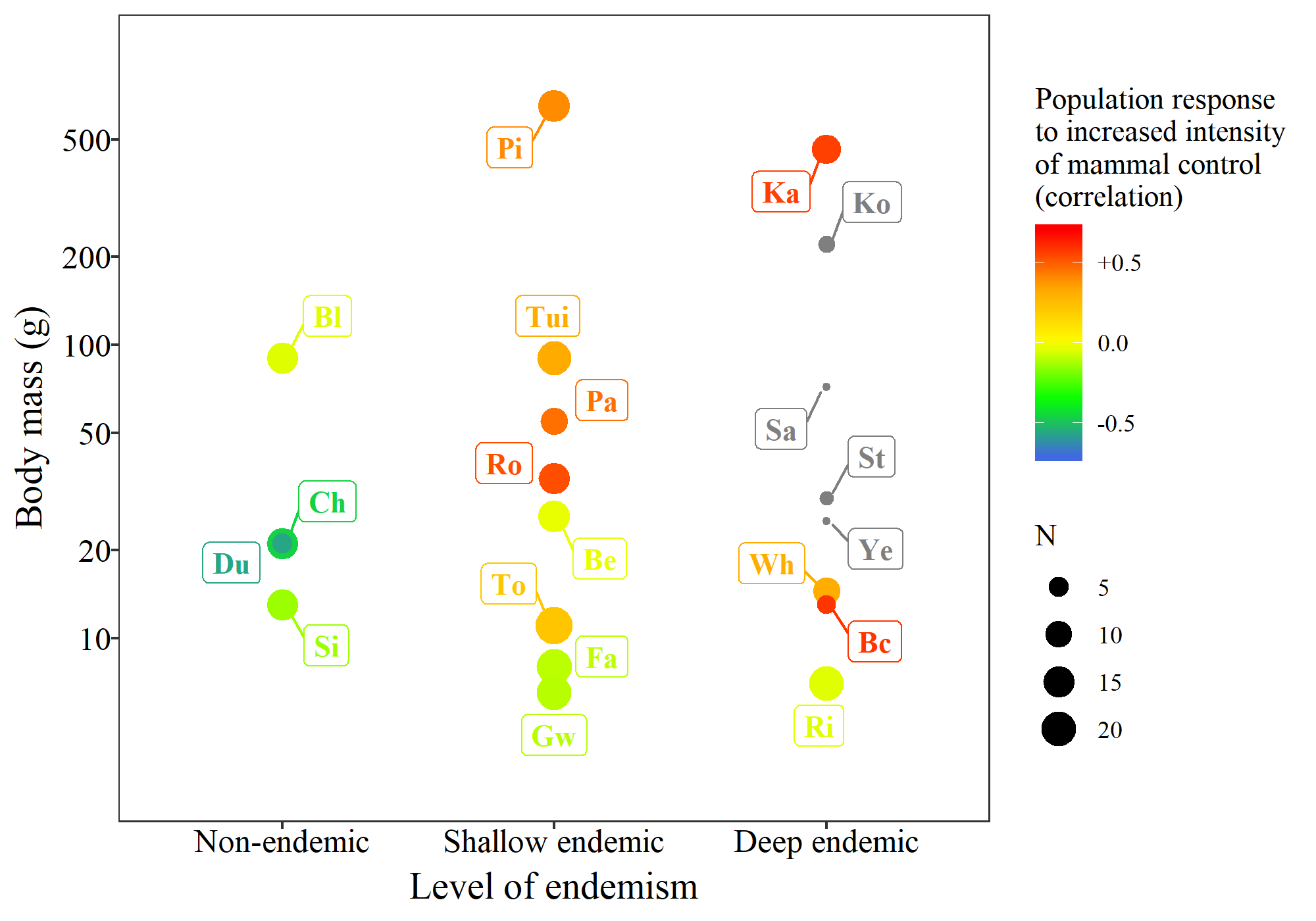


**Appendix S8.** The associations between bird population response (standardised mean difference (SMD)) and control intensity, according to body mass and level of endemism (Non-endemic = anthropogenically or self-introduced after 1800, Shallow endemics = species are endemic at the level of species or genera, Deep endemics = species are endemic at the level of family). Please refer to Table 2 for information on body mass and level of endemism for each species (and sub-species). Key for terms in figure: Population response (correlation): Pearson correlation coefficients derived for associations between population responses and intensity of mammal control (available in Table S5); N: number of population responses from unique treatments of mammal control (max = 24, tomtit). Key for abbreviations (please refer to Table 2 for scientific names of bird species): Bc-Brown Creeper, B-Bellbird, Bl – Blackbird, Ch-Chaffinch, Du-Dunnock, Fa-Fantail, Gw-Grey Warbler, Ka-Kaka, Ko-Kokako, Pa-Parakeet, Pi-Pigeon, R-Rifleman, Ro-Robin, Sa-Saddleback, Si-Silvereye, St-Stitchbird, To-Tomtit, Wh-Whitehead, Ye-Yellowhead. Bird species in grey are nationally rare and population data were lacking across all treatments.
