# Supplementary material for: Responses of New Zealand forest birds to management of introduced mammals
Source: Conserv Biol. 2020 Mar 23;35(1):35–49. doi: 10.1111/cobi.13456 (PMC7984369; doi:10.1111/cobi.13456)
Supplement: Supplementary file 3 — Supporting Material [file COBI-35-35-s002.docx]

**Appendix S4.** Biodiversity projects where bird population responses were reported over multiple years, and where management of introduced mammalian predators (namely stoats (*Mustela erminea*), possums (*Trichosurus vulpecula*), and ship rats (*Rattus rattus*)) was described. Key for terms in columns: Project (identifier) - name of project with an identifier for the unique treatment type, in parentheses: MI - Mainland island, reintroduced - species reintroduced to the site, NT - Non-treatment; Control intensity / Control type: ‘high’ intensity control included projects where eradication of mustelids, possums and rats (eMuPR), plus occasionally mice (eMMuPR) occurred, as well as sites with high-intensity rat, possum and mustelid control (hMuPR); ‘low’ intensity control included sites with low intensity rat, possum and/or mustelid control (lMuPR, lMuR), eradication of possums but not rats or mustelids (eP) and periodic possum control performed every 2-4 years or every 5-8 years (pp3 and pp6 respectively); ‘no’ control included sites with no control of possums, rats or mustelids (*n*); Total (yrs): total number of annual counts; Study design: comparison between before and after management (BA), studies across time where no change in management occurred (T), comparison of sites where treatment did (impact) and did not (control) occur (CI), and studies involving a before-after-control-impact design (BACI); *N* (reporting of sample sizes): U - sample size is unclear, Y - sample sizes stated, N - not reported; $\bar{x} ($Means) and *SE*s: Y - means reported, N - not reported, LM - estimates generated in linear models or S - survival models, I - incomplete reporting (of error); Test used (for statistical analysis): NP - Non-parametric, NS - not stated, N - no test performed, P - *P*-values only supplied, LM - Linear models, t - *t*-tests. Source: the appendix or data file number in Supporting Information is given after the citation for data sourced directly from project managers or from online reports. Rows shaded grey indicate South Island sites, rows in bold indicate projects that qualified for inclusion in the meta-analysis via the standardised mean difference (SMD).

| **Project (identifier)** | **Control intensity** | **Control type** | **First year** | **Last**  **year** | **Extent (yrs)** | **Total (yrs)** | **Study design** | ***n*** | $\bar{\boldsymbol{x}}$ | ***SE*** | **Test used** | **Source** |
| --- | --- | --- | --- | --- | --- | --- | --- | --- | --- | --- | --- | --- |
| Kapiti Island | high | eMMuPR | 1991 | 2002 | 12 | 6 | BA | U | N | N | NS | Miskelly and Robertson (2003) |
| Tiritiri Matangi | high | eMMuPR | 1987 | 2010 | 24 | 10 | BA | U | Y | N | NP | Graham et al. (2013) |
| Tiritiri Matangi (reintroduced) | high | eMMuPR | 1987 | 2010 | 24 | 10 | T | U | Y | N | NP | Graham et al. (2013) |
| Auckland | high | eMuPR | 2009 | 2014 | 6 | 6 | CI | N | LM | N | LM | Ruffell and Didham (2017) |
| Cape Kidnappers | high | eMuPR | 2015 | 2015 | 1 | 1 | T | Y | Y | Y | N | McLennan (2017) |
| **Maungatautiri (MI)** | **high** | **eMuPR** | **2002** | **2011** | **10** | **4** | **BA** | **Y** | **Y** | **Y** | **NP** | **Fitzgerald and Innes (2014)** |
| **Maungatautari (reintroduced)** | **high** | **eMuPR** | **2008** | **2011** | **4** | **4** | **BA** | **Y** | **Y** | **Y** | **NP** | **Fitzgerald and Innes (2014)** |
| Maungatautari (MI) | high | eMuPR | 2010 | 2010 | 1 | 1 | CI | Y | Y | Y | N | Iles and Kelly (2014) |
| Orokonui | high | eMuPR | 2005 | 2015 | 11 | 11 | BACI | U | Y | N | N | Onley unpublished (2017) |
| Orokonui (reintroduced) | high | eMuPR | 2008 | 2015 | 8 | 8 | T | U | Y | N | N | Onley unpublished (2017) |
| **Zealandia** | **high** | **eMuPR** | **1995** | **2016** | **22** | **9** | **BA** | **Y** | **Y** | **Y** | **P** | **Miskelly (2018)** |
| **Zealandia (reintroduced)** | **high** | **eMuPR** | **1995** | **2016** | **22** | **6** | **BA** | **Y** | **Y** | **Y** | **P** | **Miskelly (2018)** |
| Auckland | high | hMuPR | 2009 | 2014 | 6 | 6 | CI | N | LM | N | LM | Ruffell and Didham (2017) |
| Bay of Plenty (Kaharoa) | high | hMuPR | 1990 | 1996 | 7 | 7 | BA | N | Y | N | N | Innes et al. (1999) |
| Bay of Plenty (Rotoehu) | high | hMuPR | 1990 | 1996 | 7 | 7 | BA | N | Y | N | N | Innes et al. (1999) |
| Benneydale | high | hMuPR | 2011 | 2014 | 4 | 4 | BA | Y | S | S | N | Armstrong (2017) |
| **Boundary Stream (MI)** | **high** | **hMuPR** | **1996** | **2006** | **11** | **11** | **T** | **Y** | **Y** | **Y** | **N** | **Ward-Smith et al. (2006)** |
| Dart 1 | high | **hMuPR** | 2001 | 2009 | 9 | 9 | T | Y | N | N | LM | Hoare et al. (2013) |
| Hurunui (South) | high | **hMuPR** | 1995 | 2009 | 15 | 14 | T | Y | N | N | LM | Hoare et al. (2013) |
| **KMA (Rata Ridge)** | **high** | **hMuPR** | **2005** | **2005** | **1** | **1** | **CI** | **Y** | **Y** | **Y** | **LM+NP** | **Baber et al. (2009)** |
| Landsborough 1 | high | hMuPR | 1998 | 2009 | 12 | 9 | T | U | Y | N | LM | O'Donnell and Hoare (2012) |
| Landsborough 1 | high | hMuPR | 1998 | 2009 | 12 | 9 | T | U | Y | N | LM | Hoare et al. (2013) |
| Motatau | high | hMuPR | 1996 | 2001 | 6 | 6 | BACI | Y | Y | N | LM | Innes et al. (2004) |
| Pureora (Mapara) | high | hMuPR | 1990 | 1996 | 7 | 7 | T | N | Y | N | N | Innes et al. (1999) |
| **Pureora (Waipapa)** | **high** | **hMuPR** | **1978** | **1999** | **22** | **4** | **CI** | **Y** | **Y** | **Y** | **LM** | **Smith and Westbrooke (2004)** |
| **RNRP (MI)** | **high** | **hMuPR** | **1998** | **2011** | **14** | **14** | **BA** | **Y** | **Y** | **Y** | **N** | **Harper et al. (2012)** |
| RNRP (MI) | high | hMuPR | 1997 | 2009 | 13 | 13 | T | Y | N | N | LM | Hoare et al. (2013) |
| **Rotopounamu** | **high** | **hMuPR** | **2010** | **2012** | **3** | **3** | **T** | **Y** | **Y** | **Y** | **N** | **McNickle (2012)** |
| **Te Urewera (MI)** | **high** | **hMuPR** | **1997** | **2011** | **15** | **15** | **T** | **Y** | **Y** | **Y** | **N** | **Moorcroft unpublished (2017)** |
| Trounson (MI) | high | hMuPR | 1995 | 2010 | 16 | 16 | BA | N | Y | I | N | Anon (2011); Beauchamp (2001) |
| Trounson (MI) | high | hMuPR | 1996 | 2002 | 6 | 6 | T | Y | Y | Y | NP | Gillies et al. (2003) |
| **Wainuiomata (MI)** | **high** | **hMuPR** | **2005** | **2015** | **11** | **11** | **BA** | **Y** | **Y** | **Y** | **N** | **Crisp unpublished, 2016** |
| Auckland | low | hMuPR | 2009 | 2014 | 6 | 6 | CI | N | LM | N | LM | Ruffell and Didham (2017) |
| **Eglinton (Walker Creek)** | **low** | lMuPR | **2005** | **2009** | **5** | **5** | **T** | **Y** | **Y** | **Y** | **N** | **Greene and Pryde (2012)** |
| Flora | low | lMuPR | 2002 | 2010 | 9 | 9 | T | Y | LM | LM | LM | Masuda et al. (2014) |
| **Hamilton** | **low** | lMuPR | **2004** | **2012** | **9** | **5** | **BA** | **Y** | **Y** | **Y** | **LM** | **Fitzgerald and Innes (2013)** |
| Napier | low | lMuPR | 2009 | 2014 | 6 | 6 | BA | N | N | N | NP | MacLeod et al. (2015) |
| **RNRP (Lakehead)** | **low** | lMuPR | **1997** | **2011** | **15** | **15** | **BA** | **Y** | **Y** | **Y** | **N** | **Harper et al. (2012)** |
| Wellington | low | lMuPR | 2011 | 2015 | 5 | 5 | T | Y | Y | Y | LM | McArthur et al. (2016) |
| Eglinton **(**Knobs Flat) | low | lMuR | 1983 | 1993 | 11 | 10 | BA | Y | N | N | LM | O'Donnell (1996) |
| Hawdon | low | lMuR | 1983 | 1993 | 11 | 11 | BA | Y | N | N | LM | O'Donnell (1996) |
| Mt Stokes | low | lMuR | 1983 | 1993 | 11 | 9 | BA | Y | N | N | LM | O'Donnell (1996) |
| Kapiti | low | eP | 1991 | 1997 | 7 | 4 | BA | U | N | N | NP | Empson and Miskelly (1999) |
| **Rangitoto** | **low** | **eP** | **1990** | **1999** | **10** | **3** | **BA** | **Y** | **Y** | **Y** | **LM** | **Spurr and Anderson (2004)** |
| **Aorangi (Aorangi)** | **low** | **pp3** | **2013** | **2017** | **5** | **5** | **BACI** | **Y** | **Y** | **Y** | **NP** | **Fea unpublished (2017)** |
| **Tararua (Project Kaka)** | **low** | **pp3** | **2009** | **2011** | **3** | **3** | **BACI** | **Y** | **Y** | **Y** | **LM** | **Griffiths (2014)** |
| Alexander | low | pp6 | 2012 | 2014 | 3 | 3 | BACI | Y | Y | N | NP | Peterson (2014) |
| **Aorangi (Remutaka)** | **low** | **pp6** | **2014** | **2017** | **4** | **4** | **BACI** | **Y** | **Y** | **Y** | **NP** | **Fea unpublished (2017)** |
| Auckland | low | pp6 | 2009 | 2014 | 6 | 6 | CI | N | LM | N | LM | Ruffell and Didham (2017) |
| Caples | low | pp6 | 2001 | 2009 | 9 | 9 | BA | Y | N | N | LM | Hoare et al. (2013) |
| **Catlins 1** | **low** | **pp6** | **1998** | **2002** | **5** | **5** | **BA** | **Y** | **Y** | **Y** | **LM** | **Katzenberger and Ross (2017)** |
| **Maungatautari (Pirongia)** | **low** | **pp6** | **2002** | **2011** | **10** | **4** | **BA** | **Y** | **Y** | **Y** | **N** | **Fitzgerald and Innes (2014)** |
| **Otago (Hampden)** | **low** | **pp6** | **2005** | **2009** | **5** | **5** | **T** | **Y** | **Y** | **Y** | **t** | **Hamilton (2009)** |
| **Pureora (Waimanoa)** | **low** | **pp6** | **1978** | **1999** | **22** | **4** | **CI** | **Y** | **Y** | **Y** | **LM** | **Smith and Westbrooke (2004)** |
| Rolleston | low | pp6 | 2012 | 2014 | 3 | 3 | BACI | Y | Y | N | NP | Peterson (2014) |
| **Tararua (Hutt Catchment)** | **low** | **pp6** | **2009** | **2012** | **4** | **4** | **BACI** | **N*** | **Y** | **Y** | **LM** | **Griffiths (2014)** |
| **Tongariro** | **low** | **pp6** | **2005** | **2012** | **8** | **8** | **T** | **Y** | **Y** | **Y** | **N** | **Guillotel unpublished (2017)** |
| **Wainuiomata (Reference)** | **low** | **pp6** | **2005** | **2015** | **11** | **11** | **T** | **N*** | **Y** | **Y** | **N** | **Crisp unpublished, 2016** |
| Benneydale | no | N | 2011 | 2014 | 4 | 4 | BA | Y | S | S | N | Armstrong (2017) |
| Blue Mountains | no | N | 1983 | 1993 | 11 | 9 | T | Y | N | N | LM | O'Donnell (1996) |
| **Boundary Stream (Cashes)** | **low** | **N** | **1996** | **2006** | **11** | **11** | **T** | **Y** | **Y** | **Y** | **N** | **Ward-Smith et al. (2006)** |
| Burwood | no | N | 1983 | 1993 | 11 | 6 | T | Y | N | N | LM | O'Donnell (1996) |
| Catlins 2 (Thisbe Stream) | no | N | 1983 | 1993 | 11 | 6 | T | Y | N | N | LM | O'Donnell (1996) |
| Catlins 2 (Catlins River) | no | N | 1983 | 1993 | 11 | 5 | T | Y | N | N | LM | O'Donnell (1996) |
| Dart 2 (terrace) | no | N | 1983 | 1993 | 11 | 4 | T | Y | N | N | LM | O'Donnell (1996) |
| Dart 2 (valley) | no | N | 1983 | 1993 | 11 | 3 | T | Y | N | N | LM | O'Donnell (1996) |
| **Eglinton (Knobs Flat)** | **no** | **N** | **2005** | **2009** | **5** | **5** | **T** | **Y** | **Y** | **Y** | **N** | **Greene and Pryde (2012)** |
| Hurunui North | no | N | 1995 | 2009 | 15 | 14 | T | Y | N | N | LM | Hoare et al. (2013) |
| **Kowhai Bush** | **no** | **N** | **1976** | **2001** | **26** | **4** | **T** | **Y** | **Y** | **Y** | **LM** | **Barnett (2011)** |
| Landsborough 2 | no | N | 1983 | 1993 | 11 | 3 | T | Y | N | N | LM | O'Donnell (1996) |
| Northland **(**Mataraua) | no | N | 1979 | 1993 | 15 | 2 | HR | Y | Y | N | NP | Pierce et al. (1993) |
| Northland **(**Omahuta) | no | N | 1979 | 1993 | 15 | 2 | HR | Y | Y | N | NP | Pierce et al. (1993) |
| Northland **(**Puketi) | no | N | 1979 | 1993 | 15 | 2 | HR | Y | Y | N | NP | Pierce et al. (1993) |
| Northland **(**Raetea) | no | N | 1979 | 1993 | 15 | 2 | HR | Y | Y | N | NP | Pierce et al. (1993) |
| Northland **(**Russell) | no | N | 1979 | 1993 | 15 | 2 | HR | Y | Y | N | NP | Pierce et al. (1993) |
| Northland **(**Waipoua) | no | N | 1979 | 1993 | 15 | 2 | HR | Y | Y | N | NP | Pierce et al. (1993) |
| **Otago (Dunedin)** | **no** | **N** | **2005** | **2009** | **5** | **5** | **T** | **Y** | **Y** | **Y** | **t** | **Hamilton (2009)** |
| Pelorus | no | N | 1983 | 2006 | 24 | 12 | HR | N | Y | Y | LM | Carpenter et al. (2017) |
| Poulter Valley | no | N | 1983 | 1993 | 11 | 4 | T | Y | N | N | LM | O'Donnell (1996) |
| **RNRP (NT)** | **no** | **N** | **2003** | **2011** | **9** | **9** | **T** | **Y** | **Y** | **Y** | **N** | **Harper et al. (2012)** |
| Rotoroa 1 | no | N | 1974 | 2006 | 33 | 16 | T | N | Y | N | NP | Elliott et al. (2010) |
| Rotoroa 2 | no | N | 2002 | 2009 | 15 | 7 | T | Y | N | N | LM | Hoare et al. (2013) |
| Rowallan | no | N | 1983 | 1993 | 11 | 12 | T | Y | N | N | LM | O'Donnell (1996) |
| **Tararua (NT)** | **no** | **N** | **2009** | **2012** | **4** | **4** | **BACI** | **N*** | **Y** | **Y** | **LM** | **Griffiths (2014)** |
| Waikaia | no | N | 1983 | 1993 | 11 | 7 | T | Y | N | N | LM | O'Donnell (1996) |
| **Waitutu (Poteriteri)** | **no** | **N** | **2006** | **2010** | **5** | **5** | **T** | **Y** | **Y** | **Y** | **N** | **Greene et al. (2013)** |
| **Waitutu (Waitutu)** | **no** | **N** | **2006** | **2010** | **5** | **5** | **T** | **Y** | **Y** | **Y** | **N** | **Greene et al. (2013)** |
| Windbag | no | N | 1983 | 1993 | 11 | 10 | T | Y | N | N | LM | O'Donnell (1996) |

**Literature Cited**

Anon. 2011. Trounson Kauri Park Mainland Island Annual Report 2010/2011. Department of Conservation, Northland, New Zealand. Available from <https://www.doc.govt.nz/Documents/conservation/land-and-freshwater/land/trounson-mainland-island-annual-report-2010-11.pdf> (accessed February 2020).

Armstrong DP. 2017. Population responses of a native bird species to rat control. Journal of Wildlife Management **81**:342-346.

Baber M, Brejaart R, Babbitt K, Lovegrove T, Ussher G. 2009. Response of non-target native birds to mammalian pest control for kokako (*Callaeas cinerea*) in the Hunua Ranges, New Zealand. Notornis **56**:176-182.

Barnett C. 2011. Changes in the observed bird abundance in a modified forest at Kowhai Bush, Kaikoura. Notornis **58**:131-138.

Beauchamp T. 2001. Trounson Kauri Park Mainland Island Annual Technical Report 2000/2001. Pages 1-79. Department of Conservation, Whangarei, New Zealand.

Carpenter JK, Kelly D, Clout MN, Karl BJ, Ladley JJ. 2017. Trends in the detections of a large frugivore (Hemiphaga novaeseelandiae) and fleshy-fruited seed dispersal over three decades. New Zealand Journal of Ecology **41**:41-46.

Elliott GP, Wilson PR, Taylor RH, Beggs JR. 2010. Declines in common, widespread native birds in a mature temperate forest. Biological Conservation **143**:2119-2126.

Empson RA, Miskelly CM. 1999. The risks, costs and benefits of using brodifacoum to eradicate rats from Kapiti Island, New Zealand. New Zealand Journal of Ecology **23**:241-254.

Gillies CA, Leach MR, Coad NB, Theobald SW, Campbell J, Herbert T, Graham PJ, Pierce RJ. 2003. Six years of intensive pest mammal control at Trounson Kauri Park, a Department of Conservation "mainland island", June 1996-July 2002. New Zealand Journal of Zoology **30**:399-420.

Graham M, Veitch D, Aguilar G, Galbraith M. 2013. Monitoring terrestrial bird populations on Tiritiri Matangi Island, Hauraki Gulf, New Zealand, 1987-2010. New Zealand Journal of Ecology **37**:359-369.

Greene TC, Dilks PJ, Westbrooke IM, Pryde MA. 2013. Monitoring selected forest bird species through aerial application of 1080 baits, Waitutu, New Zealand. New Zealand Journal of Ecology **37**:41-50.

Greene TC, Pryde MA. 2012. Three population estimation methods compared for a known South Island robin population in Fiordland, New Zealand. New Zealand Journal of Ecology **36**:340-352.

Hoare JM, Monks A, O'Donnell CFJ. 2013. Do population indicators work? Investigating correlated responses of bird populations in relation to predator management. Ecological Indicators **25**:23-34.

Iles JM, Kelly D. 2014. Restoring bird pollination of Fuchsia excorticata by mammalian predator control. New Zealand Journal of Ecology **38**:297-306.

Innes J, Hay R, Flux I, Bradfield P, Speed H, Jansen P. 1999. Successful recovery of North Island kokako *Callaeas cinerea wilsoni* populations, by adaptive management. Biological Conservation **87**:201-214.

Innes J, Nugent G, Prime K, Spurr EB. 2004. Responses of kukupa (*Hemiphaga novaeseelandiae*) and other birds to mammal pest control at Motatau, Northland. New Zealand Journal of Ecology **28**:73-81.

Katzenberger J, Ross J. 2017. Mohoua ochrocephala abundance in the Catlins following aerial 1080 control. New Zealand Natural Sciences **42**:1-8.

MacLeod JL, R. D, C. L, B.M. S, S. GA. 2015. Possum control and bird recovery in an urban landscape, New Zealand. Conservation Evidence **12**:44-47.

Masuda BM, McLean M, Gaze P. 2014. Changes in passerine populations during ongoing predator control at a community-based conservation project: A case study to evaluate presence-absence surveys. Notornis **61**:75-83.

McLennan JA. 2017. The Cape to City programme: baseline bird counts in treatment and non-treatment areas. Pages 1-35. John A McLennan Environmental Services Ltd, Hawke's Bay, New Zealand. Available from <https://www.pfhb.nz/assets/Image-Gallery/McLennan-2017-The-Cape-to-City-programme-baseline-bird-counts-in-treatment-and-non-treatment-areas.pdf> (accessed February 2020).

Miskelly CM. 2018. Changes in the forest bird community of an urban sanctuary in response to pest mammal eradications and endemic bird reintroductions. Notornis **65**:132-151.

Miskelly CM, Robertson HA. 2003. Response of forest birds to rat eradication on Kapiti Island. Science Posters. Department of Conservation, Wellington, New Zealand. Available from <http://www.doc.govt.nz/Documents/science-and-technical/SciencePoster37.pdf> (accessed February 2020).

O'Donnell C. 1996. Monitoring mohua (yellowhead) populations in the South Island, New Zealand, 1983-93. New Zealand Journal of Zoology **23**:221-228.

O'Donnell C, Hoare J. 2012. Quantifying the benefits of long-term integrated pest control for forest bird populations in a New Zealand temperate rainforest. New Zealand Journal of Ecology **36**:131-140.

Peterson A. 2014. The long-term impacts of an aerial 1080 application on non-target forest species. Msc thesis. Pages 1-98. School of Biological Sciences. University of Canterbury, Christchurch, New Zealand.

Pierce R, Atkinson R, Smith E. 1993. Changes in bird numbers in six Northland forests 1979–1993. Notornis **40**:285-293.

Ruffell J, Didham RK. 2017. Conserving biodiversity in New Zealand's lowland landscapes: does forest cover or pest control have a greater effect on native birds? New Zealand Journal of Ecology **41**:23-33.

Smith ANH, Westbrooke IM. 2004. Changes in bird conspicuousness at Pureora Forest. Notornis **51**:21-25.

Spurr EB, Anderson SH. 2004. Bird species diversity and abundance before and after eradication of possums and wallabies on Rangitoto Island, Hauraki Gulf, New Zealand. New Zealand Journal of Ecology **28**:143-149.
