# Supplementary material for: Responses of New Zealand forest birds to management of introduced mammals
Source: Conserv Biol. 2020 Mar 23;35(1):35–49. doi: 10.1111/cobi.13456 (PMC7984369; doi:10.1111/cobi.13456)
Supplement: Supplementary file 4 — Supporting Material [file COBI-35-35-s004.pdf]

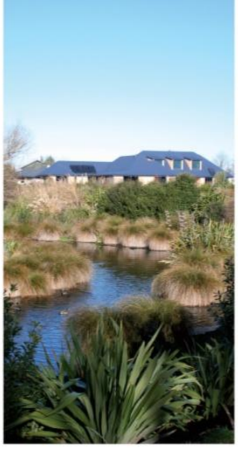

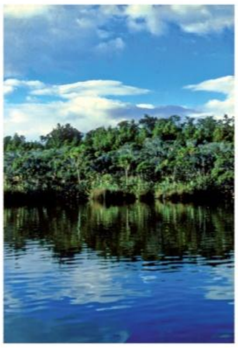

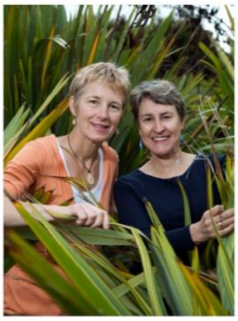

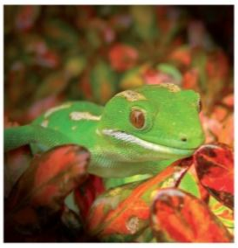

Changes in bird abundance at Maungatautari after 2006 pest mammal eradication, results to 2011

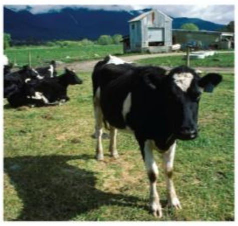

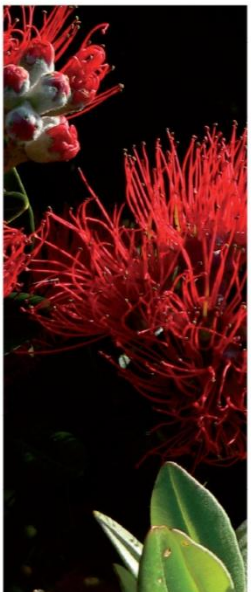

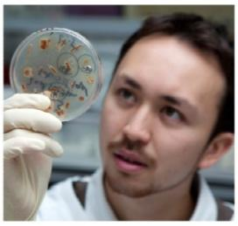

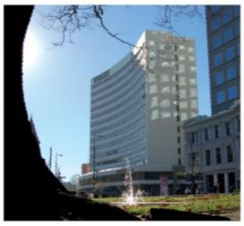

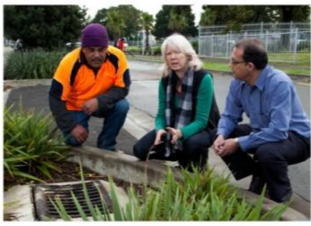

Landcare Research
Manaaki Whenua

Changes in bird abundance at Maungatautari after 2006 pest mammal eradication, results to 2011

Neil Fitzgerald, John Innes

Landcare Research

Prepared for:

Ministry of Business, Innovation and Employment

Level 3, 33 Bowen Street
PO Box 5762
Wellington 6145
New Zealand

February 2014

Landcare Research, Gate 10 Silverdale Road, University of Waikato Campus, Private Bag 3127, Hamilton 3240, New Zealand, Ph +64 7 859 3700, Fax +64 7 859 3701, www.landcareresearch.co.nz

Reviewed by:	*Approved for release by:*
Norman Mason<br>Researcher<br>Landcare Research	Bill Lee<br>Portfolio Leader – Managing Biodiversity<br>Landcare Research
Landcare Research Contract Report:	LC1744

Disclaimer

This report has been prepared by Landcare Research for Ministry of Science and Innovation. If used by other parties, no warranty or representation is given as to its accuracy and no liability is accepted for loss or damage arising directly or indirectly from reliance on the information in it.

TELARC SAI registered

ISO 14001

© Landcare Research New Zealand Ltd 2014

No part of this work covered by copyright may be reproduced or copied in any form or by any means (graphic, electronic or mechanical, including photocopying, recording, taping, information retrieval systems, or otherwise) without the written permission of the publisher.

Contents

Summary v

1 Introduction 1

2 Background 2

2.1 Treatment site (pest fenced) 2

2.2 Non- treatment sites (non-fenced) 2

3 Objective 3

4 Methods 3

4.1 5-minute bird counts 7

4.2 Maximum counts of tūī, bellbird, and New Zealand pigeon 8

5 Results 8

5.1 5-minute bird counts 8

5.2 Maximum count of tūī, bellbird, and New Zealand pigeon 11

6 Discussion and conclusions 12

7 Recommendations 14

8 Acknowledgements 14

9 References 14

Appendix 1 – Mean number of birds per 5-minute count 17

Appendix 2 – Scientific names of birds used in text 19

Appendix 3 – Scientific names of mammals used in text 20

Appendix 4 – Scientific names of plants used in text 21

Summary

Project and Client

- In 2006 the Maungatautari Ecological Island Trust completed construction of a fence designed to exclude all pest mammals around more than 3300 ha of native forest, and then eradicated virtually all mammals from within the fenced area. This report describes and compares pre- and post-eradication bird counts undertaken each 3 years at Maungatautari Scenic Reserve and ‘non-treatment’ (other pest control) sites between 2002 and 2011 by Landcare Research, Hamilton. This research was supported by core funding for Crown Research Institutes from the Ministry of Business, Innovation and Employment’s Science and Innovation Group.

Objective

- To investigate whether there have been changes in avifaunal abundance or composition at Maungatautari that can be attributed to the eradication of pest mammals.

Methods

- 5-minute bird counts were undertaken at Maungatautari Scenic Reserve (pre and post eradication treatment), Pirongia Forest Park (non-treatment), and Hakarimata Scenic Reserve (non-treatment) between 8 November and 1 December 2011.
- The maximum number of tūī, New Zealand pigeon and bellbirds observed at any one time was recorded in half-hour periods, and the weighted means of these maxima were calculated for treatment and non-treatment areas.
- Results were compared with counts previously undertaken in 2002, 2005, and 2008.

Results

- At Maungatautari, four native species (New Zealand pigeon, shining cuckoo, tomtit, and tūī) and one introduced species (greenfinch) were significantly more abundant in 2011 (5 years after pest eradication) than in 2002 (4 years before pest eradication).
- At Maungatautari, two native species (tūī and tomtit) were significantly more abundant in both post-eradication counts than both pre-eradication counts.
- At Maungatautari, two native species (grey warbler and silvereye) and one exotic species (Eurasian blackbird) were significantly less abundant in 2011 than in 2002.
- At Maungatautari , one exotic species (Eurasian blackbird) was significantly less abundant in both post-eradication counts than both pre-eradication counts.
- At non-fenced study sites, one native species (tūī) and one introduced species (Eastern rosella) were significantly more abundant in 2011 than in 2002.
- There was no clear consistent pattern of change in the maximum number of tūī, bellbird or New Zealand pigeon counted at one time, through time or in response to fencing.

Conclusions

- The removal of virtually all pest mammals from Maungatautari has resulted in increased abundance and diversity of native bird species and a decline in an exotic bird species between 2002 and 2011.
- Maximum counts of tūī, bellbirds of New Zealand pigeons undertaken in November–December did not show clear pattern of change at Maungatautari. These counts are more appropriately undertaken outside the nesting season (e.g. autumn–winter) when birds may be flocking.

Recommendations

- 5-minute bird counts should be repeated in November–December 2014 both at Maungatautari and at non-treatment sites to monitor changes in avifauna resulting from eradication of mammalian pests at Maungatautari.
- Maximum counts of tūī, bellbirds, and New Zealand pigeon should not be repeated in the breeding season in future. We are not currently planning to start a new series of non-breeding flock counts. However, if done in the future these should be carried out in late winter for tūī and bellbirds, and autumn for New Zealand pigeon.
- There is scope to further investigate mechanisms and pattern of avian response to release from mammalian predation. This could include:
    - conducting bird counts at other pest-free sites to give examples of long-term dynamics of avian assemblages and abundance change at sites with high (fenced sanctuaries) and low (offshore islands) potential for avian dispersal. Does dispersal limit avian recovery in mainland sanctuaries?
    - comparing breeding productivity and success at Maungatautari and non-fenced sites to help quantify increased recruitment to the local population and spillover to more distant areas.

1 Introduction

On 31 August 2006 the Maungatautari Ecological Island Trust (MEIT) completed construction of a 47-km-long mammal pest fence encircling more than 3300 ha of native forest in the Waikato Region. This forest includes Maungatautari Mountain Scenic Reserve (2398 ha) combined with adjoining Māori and private land, and is surrounded by developed farmland. In November 2006, MEIT began a programme that successfully eradicated—or persistently reduced to extremely low levels—all pest mammals except house mice (see Appendix 3 for scientific names of all mammal species) within the fenced area, creating the largest area of virtually pest free forest on the New Zealand mainland.

There is considerable opportunity in this project to research how the ecosystem responds to both mammal removal and subsequent (re)introductions of native species. Response to pest mammal removal is an example of 'ecological release', whereby the forest system will cease to be subjected to the strong interactions imposed on it by pests such as brushtail possums, ship rats, and stoats. To robustly test these changes, minimal scientific design would demand that observations are made at Maungatautari plus non-treatment 'control' blocks both before and after eradication.

To satisfy this requirement, bird counts were initially undertaken at Maungatautari and Pirongia Forest Park—a planned non-treatment area. Intensification of pest management at some of the non-treatment blocks since the first pre-eradication counts were conducted in 2002 prompted relocation of some of the count stations used in subsequent surveys (Innes et al. 2003, 2006; Fitzgerald et al. 2009), since impacts of ubiquitous predators generally overwhelm site effects (Innes et al. 2010).

Predictably, there are now no large Waikato Forests where no pest control at all is undertaken, so the most useful comparison of the effects of pest fencing of Maungatautari is now between the fenced site (Maungatautari) and non-fenced sites. As mentioned, the non-fenced sites have been subject to pest control regimes (especially intermittent aerial 1080) that variably manage but do not eradicate target pests, primarily possums. They thus have variable abundance of all widespread New Zealand pest mammal species, whereas Maungatautari has since eradication had zero or near-zero abundance of all mammals except mice (Speedy et al. 2007; Innes & Saunders 2011).

Initial pre-eradication bird counts were made at Maungatautari and Pirongia in November–December 2002 (Innes et al. 2003). Counts were also made in several central Waikato forest remnants, including at Hakarimata Scenic Reserve in November 2002 to investigate background bird abundance in the region (Innes et al. 2005).

In November 2011, comparable counts were made at Maungatautari, Pirongia, and Hakarimata. Additional count stations were also established at Pirongia and Maungatautari to provide better baseline data from this point on.

2 Background

The study sites have similar altitude, topography, vegetation, and histories of pest impacts and control before 2006 when mammalian pests were mostly eradicated from Maungatautari.

The non-fenced sites have been subject to episodic management of a few pest species, mainly possums. They thus have variable abundance of all widespread New Zealand pest mammal species, whereas Maungatautari has, since eradication, had zero or near-zero abundance of all mammals except mice (Speedy et al. 2007; Innes & Saunders 2011).

2.1 Treatment site (pest fenced)

Maungatautari is an extinct andesite volcano 15 km west of Putaruru, with forest types ranging from lowland rimu/tawa forest to montane forest dominated by tāwari-kāmahi and tāwheowheo (Clarkson et al. 2002)—scientific names of plants are listed in Appendix 4. Contiguous native forest cover ranges in altitude from 240 to 797 metres above sea level.

Before eradication, Maungatautari was treated with aerially applied 1080 (sodium monofluoroacetate) in June 1997 and July–August 2002. These aerial operations primarily targeted possums but non-target mammalian pests would also have been killed, including many ship rats, feral cats and stoats (Innes et al. 1995; Gillies & Pierce 1999; Murphy et al. 1999).

2.2 Non- treatment sites (non-fenced)

Pirongia Forest Park (16862 ha) consists of five separate forested blocks, including highly eroded basaltic and andesitic volcanic cones. The largest of these blocks (13 606 ha) encompasses Pirongia Mountain, 35 km west of Maungatautari. Both have the same forest classes, and although Pirongia has a larger altitudinal range (90–959 m) of native vegetation than Maungatautari, they have very similar vegetation at similar altitudes (Nicholls 1979; Clarkson et al. 2002). To improve comparability, we placed bird survey lines at similar altitudes at Pirongia Mountain and Maungatautari as much as possible.

Hakarimata Scenic Reserve (1831 ha) occupies part of a low-lying greywacke range 51 km north west of Maungatautari. This reserve predominantly comprises similar rimu/tawa and tawa-dominated vegetation to Maungatautari. A notable exception is a relatively small area characterised by large kauri trees (Nicholls 1979; Clarkson et al. 2002). We placed bird survey lines along a walking track that traverses the range.

In July 2007, 1080-laced cereal pellets and non-toxic prefeed were sown over Pirongia. A 460-ha area was excluded because it is treated annually by the Pirongia Te Aroaro O Kahu Restoration Society with a number of different toxins in bait stations to reduce possum and rat numbers to low densities during the breeding season of most forest bird species. By 2011 this area was increased to 715 ha. Although count stations are not within this restoration area, there is likely to be increased avian dispersal over a larger, but unknown area.

The northern half of Hakarimata Scenic Reserve was treated in 2001/2002 with a combination of aerially applied 1080-laced carrot and Feratox™ (cyanide) and Pestoff™

(brodifacoum) in bait stations. In 2005 the entire area was treated with aerially sown 1080 coated cereal baits and Feratox™ and Pestoff™ in bait stations (D. Byers, Waikato Regional Council, Hamilton, pers. comm.). These operations reduced possum abundance to low levels and are also likely to have killed other mammalian predators, including ship rats, feral cats, and stoats (Innes et al. 1995; Gillies & Pierce 1999; Murphy et al. 1999).

3 Objective

To investigate whether there have been changes in avifaunal abundance or composition at Maungatautari that can be attributed to the eradication of pest mammals.

4 Methods

Birds were counted at fenced and various non-fenced sites in 2002, 2005, 2008 and 2011. However, only data from lines that were counted in all years were used in the analyses (Figure 1, 2, and 3). Additional count stations were also established in 2011 at the fenced and non-fenced sites to provide a new baseline against which future changes in bird abundance could be assessed.

Non-fenced sites were selected with similar vegetation, altitude, and pre 2002 pest control history as Maungatautari.

Two bird count indexing techniques were used throughout the study—5-minute bird counts (5mbc), and maximum counts of three key species; tūī, bellbird, and New Zealand pigeon. The same techniques were used in all counts at all sites from 2002 to 2011, although spacing between 5mbc stations was reduced in 2011.

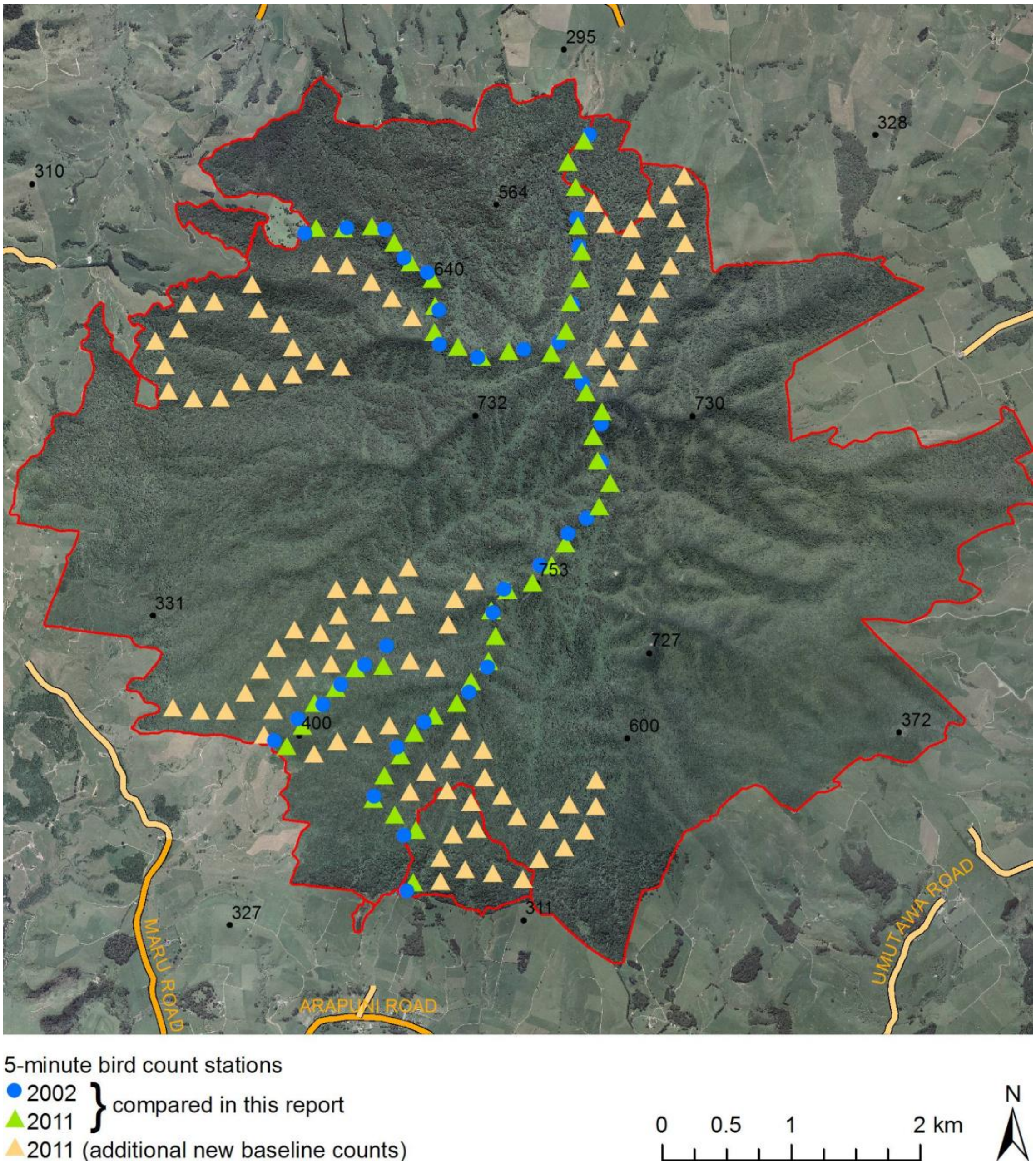

Figure 1 Locations of 5-minute bird-count stations counted in 2002 and 2011 at Maungatautari (fenced site). The pest fence is indicated in red. Count stations in the same area in both years were used to compare differences in bird counts and additional count stations established in 2011 will be used to monitor future changes.

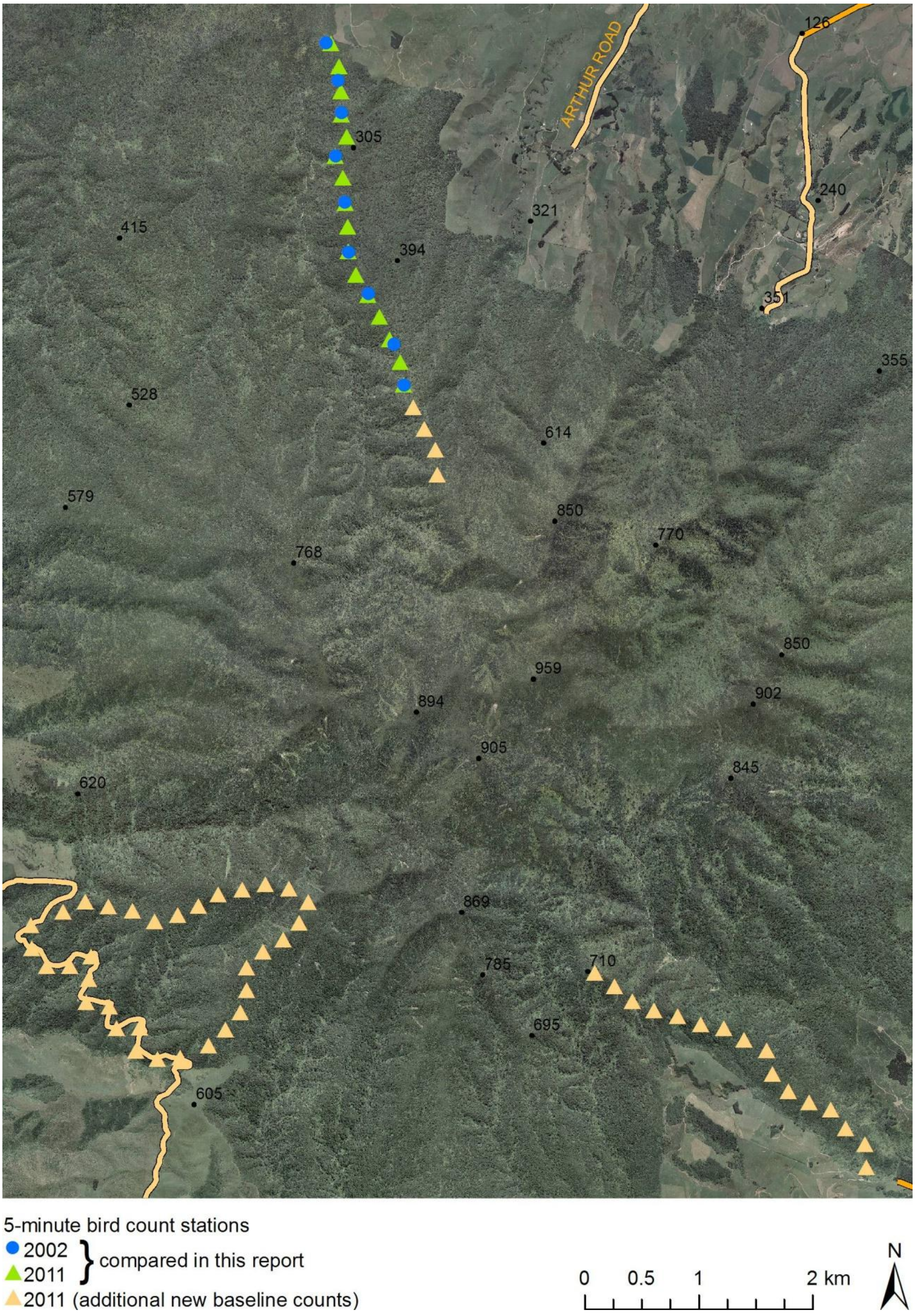

Figure 2 Locations of 5-minute bird-count stations counted in 2002 and 2011 at Pirongia Mountain (non-fenced site). Count stations in the same area in both years were used to compare differences in bird counts and additional count stations established in 2011 will be used to monitor future changes.

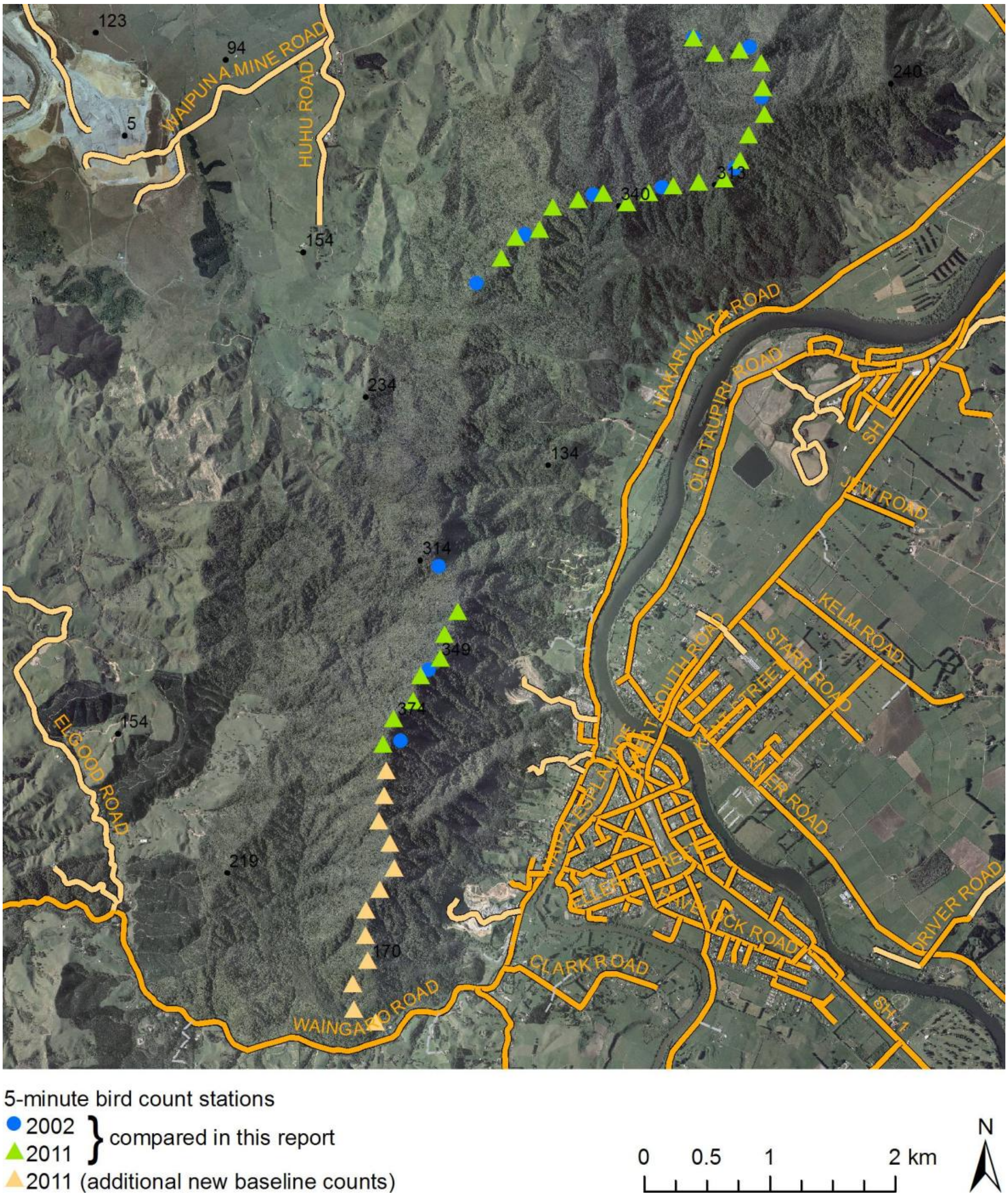

Figure 3 Locations of 5-minute bird-count stations counted in 2002 and 2011 at Hakarimata Scenic Reserve (non-fenced site). Count stations in the same area in both years were used to compare differences in bird counts and additional count stations established in 2011 will be used to monitor future changes.

4.1 5-minute bird counts

The 5mbc technique (Dawson & Bull 1975) was chosen to allow comparisons with many other counts made around the Waikato (Innes et al. 2003) and New Zealand (Hartley 2012). The technique gives a measure of abundance not absolute density of birds, but provides valuable and repeatable indices in relation to those from a non-treatment block, provided all counts are made by experienced observers at the same time of year.

In 5mbcs, all birds seen or heard within 100 m of the (stationary) observer in a 5-minute period are recorded.

In 2002–2008, each station was counted twice, on different days by different observers, and the mean of the two counts calculated. In 2011 each station was counted once only.

In 2011, counts were made along the same tracks as some of the original 2002 counts, although the location of some of the count stations along the tracks differed. New, additional count stations were also established in 2011 to provide a more reliable baseline against which future counts can be compared.

When the count stations used for this work were first established they were spaced 15 minutes walking apart. The stations were spaced by time rather than distance to accommodate the second count technique (maximum counts of key species per ½-hour). Each station was counted on two different days by different observers and the mean of these counts used for analysis. Of the stations established in 2002, 35 stations from Maungatautari, 9 from Pirongia and 11 from Hakarimata were used for comparison with 2011 counts.

In 2011 the study design was modified to increase sampling intensity and provide a new baseline for monitoring future changes. Count stations were spaced by distance, at least 200 m apart, using handheld GPS, allowing many more stations to be counted in the same space and time than the previous method. In 2011, 145 stations were counted at Maungatautari, including four new lines of count stations exploiting the large network of monitoring tracks that have been established by MEIT (Figure 1). We compared 50 of these stations with 2002 data. At Pirongia, 69 stations were counted along four lines in 2011 (Figure 2) and we compared 16 of these stations with 2002 data. At Hakarimata Scenic Reserve, 38 stations were counted in 2011 (Figure 3), and we compared 26 of these stations with 2002 data.

Count data were analysed in two ways:

- The mean number of each bird species per station in the first pre-eradication counts (2002) were compared with the most recent post-eradication counts (2011) using two-sample t-tests assuming unequal variance, for both fenced and non-fenced sites.
- Boxplots were drawn for 12 of the most abundant species at Maungatautari and counts of these species in each count year were compared using the multiple comparison procedure “Duncan’s new multiple range test” (de Mendiburu 2013). This procedure enables the two pre, and two post-eradication count years to be compared. There were insufficient non-fenced stations counted in all years to perform this analysis for those data.

Data were analysed in the R statistical computing environment (version 3.0.2, R Core Team 2013).

4.2 Maximum counts of tūī, bellbird, and New Zealand pigeon

The second technique involved counting the maximum number of tūī, New Zealand pigeons, and bellbirds seen or heard within 100 m of the observer at any one time (in practice, over about 5 seconds) during a 30-minute period while walking slowly. This technique was first trialled by Landcare Research in forests around the Waikato and on Kapiti Island in 2002 (Innes et al. 2003). This measure quantifies the flock size, or aggregation of each species and is believed to be robust with regard to differences between observers (Fitzgerald et al. 2009). The maximum number of each species was recorded in 15-minute intervals while walking between 5-minute count stations. Two adjacent 15-minute intervals were aggregated into 30-minute intervals, and the mean of the 30-minute maxima (i.e. average 'flock size') were then calculated for the fenced and non-fenced areas.

Throughout the study observer effort was spread equally between treatment and non-treatment sites to minimise observer bias between the treatments. In 2011 counts were made between 8 November and 1 December—a time of year when many bird species are conspicuous, and also when previous surveys were done (Fitzgerald et al. 2009).

5 Results

5.1 5-minute bird counts

Thirty-one species of birds (19 native, 12 introduced) were counted in one or more of the four count years (Appendix 1). Three native species were recorded in counts at Maungatautari for the first time in 2011 at stations used in previous years (whitehead, stitchbird, and North Island robin) and one species (New Zealand falcon) was recorded at a station newly established in 2011. Whitehead and stitchbird were translocated to the site in 2009 and North Island robin in 2011.

The mean counts of 5 bird species were significantly higher in 2011 than 2002 at Maungatautari, comprising 4 natives: New Zealand pigeon ($t = –2.0728$, $P = 0.04235$), shining cuckoo ($t = –2.1832$, $P = 0.0319$), tomtit ($t = –6.9143$, $P < 0.0001$), and tūī ($t = –7.6816$, $P < 0.0001$), and 1 exotic species: greenfinch ($t = –2.6574$, $P = 0.0104$). At the same lines the mean counts of 3 species were significantly lower in 2011 than in 2002, comprising two native species: grey warbler ($t = 3.3701$, $P = 0.0011$), and silvereye ($t = 3.2535$, $P = 0.0017$), and one exotic species: Eurasian blackbird ($t = 2.1051$, $P = 0.0385$).

At non-fenced sites the mean count of two species was significantly higher in 2011 than in 2002. One of these was the native tūī ($t = –2.2922$, $P = 0.0285$), and the other the exotic eastern rosella ($t = –2.1698$, $P = 0.0341$).

Multiple comparison analysis indicates the mean number of tūī counted at Maungatautari in the two count years following pest mammal eradication was significantly higher than prior to the eradication. There was no significant difference between the two pre-eradication counts or the two post-eradication counts of tūī (Figure 4).

The mean number of silvereyes counted at Maungatautari in 2002 and 2011 were significantly different from each other but not significantly different from the other years, and

there was no significant differences in mean number of bellbirds or New Zealand pigeons between years (Figure 4), suggesting there was no clear pre–post eradication difference in these species.

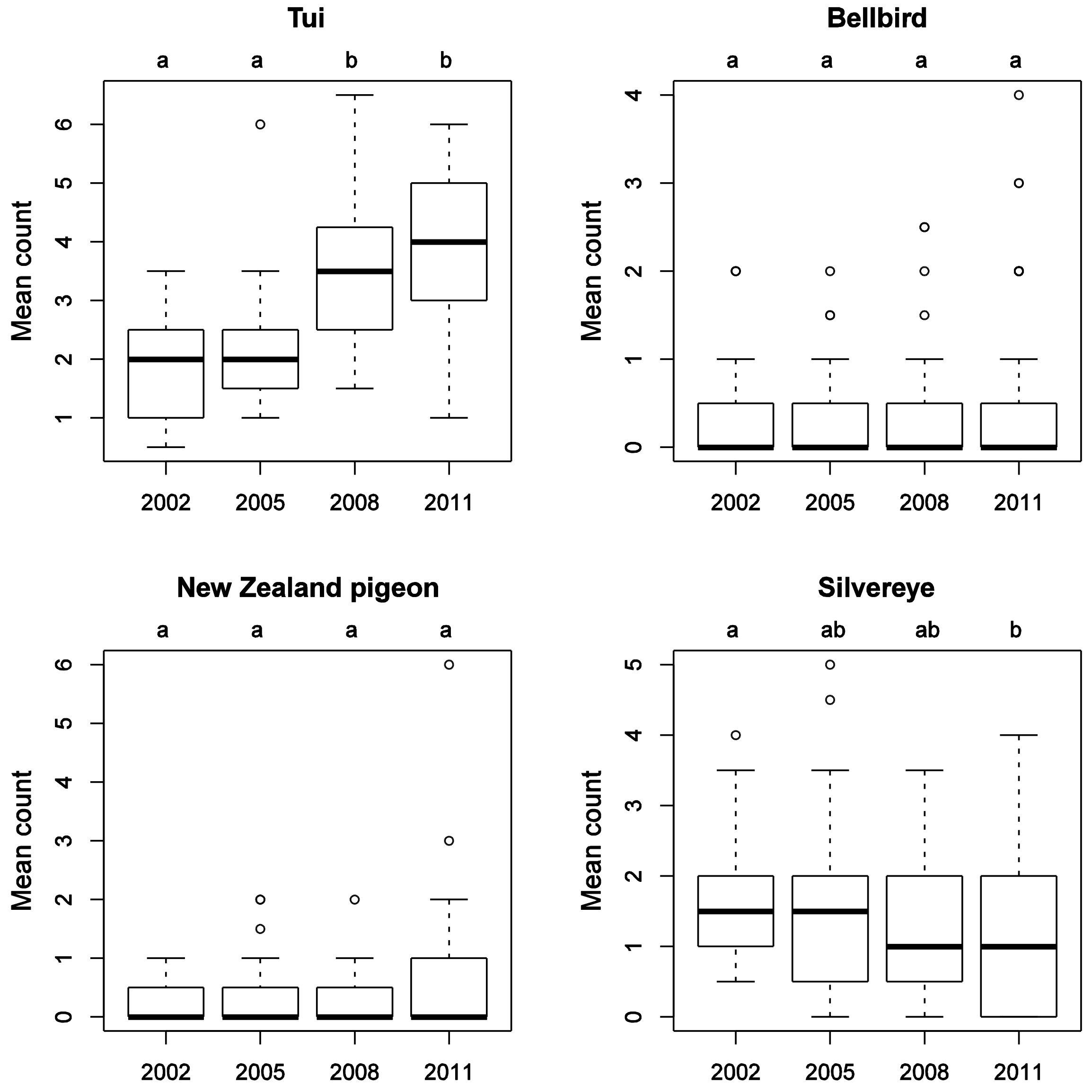

Figure 4 Box plots of mean number of four omnivorous native birds; tūī, bellbird, New Zealand pigeon, and silvereye per 5-minute count station at Maungatautari before (2002 and 2005) and after (2008 and 2011) pest mammal eradication. The thick horizontal line shows the median value (i.e. 50% of the data are greater and 50% less than this value), the box shows the upper and lower quartiles (25% of the data is greater than the upper quartile and 25% less than the lower quartile), the dashed whiskers indicate the maximum and minimum values excluding outlying data which are shown as open circles. Mean count are not significantly different ($P < 0.05$) in years with the same letter.

The mean counts of tomtits in both post-eradication counts at Maungatautari were significantly different from pre eradication counts (Figure 5). Grey warbler, shining cuckoo, and New Zealand fantail did not show consistent patterns of pre–post eradication change (Figure 5).

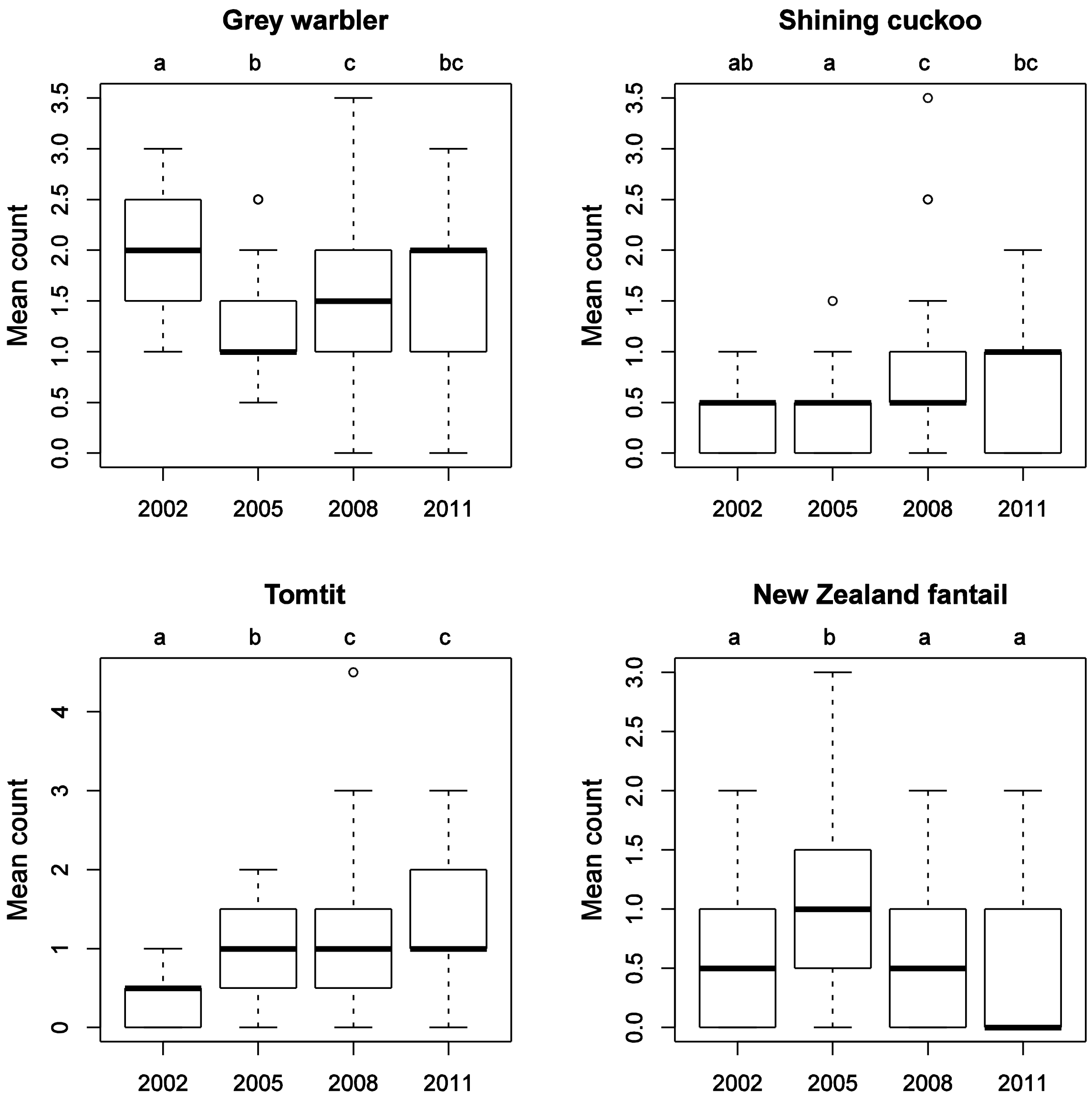

Figure 5 Box plots of mean number of four insectivorous native birds; grey warbler, shining cuckoo, tomtit, and New Zealand fantail per 5-minute count station at Maungatautari before (2002 and 2005) and after (2008 and 2011) pest mammal eradication. Mean count are not significantly different ($P < 0.05$) in years with the same letter.

The mean number of Eurasian blackbirds was significantly lower in both post-eradication counts at Maungatautari compared to pre eradication counts (Figure 6). None of the other sufficiently abundant exotic species—European goldfinch, European greenfinch, or chaffinch—showed consistent patterns of pre–post eradication change (Figure 6).

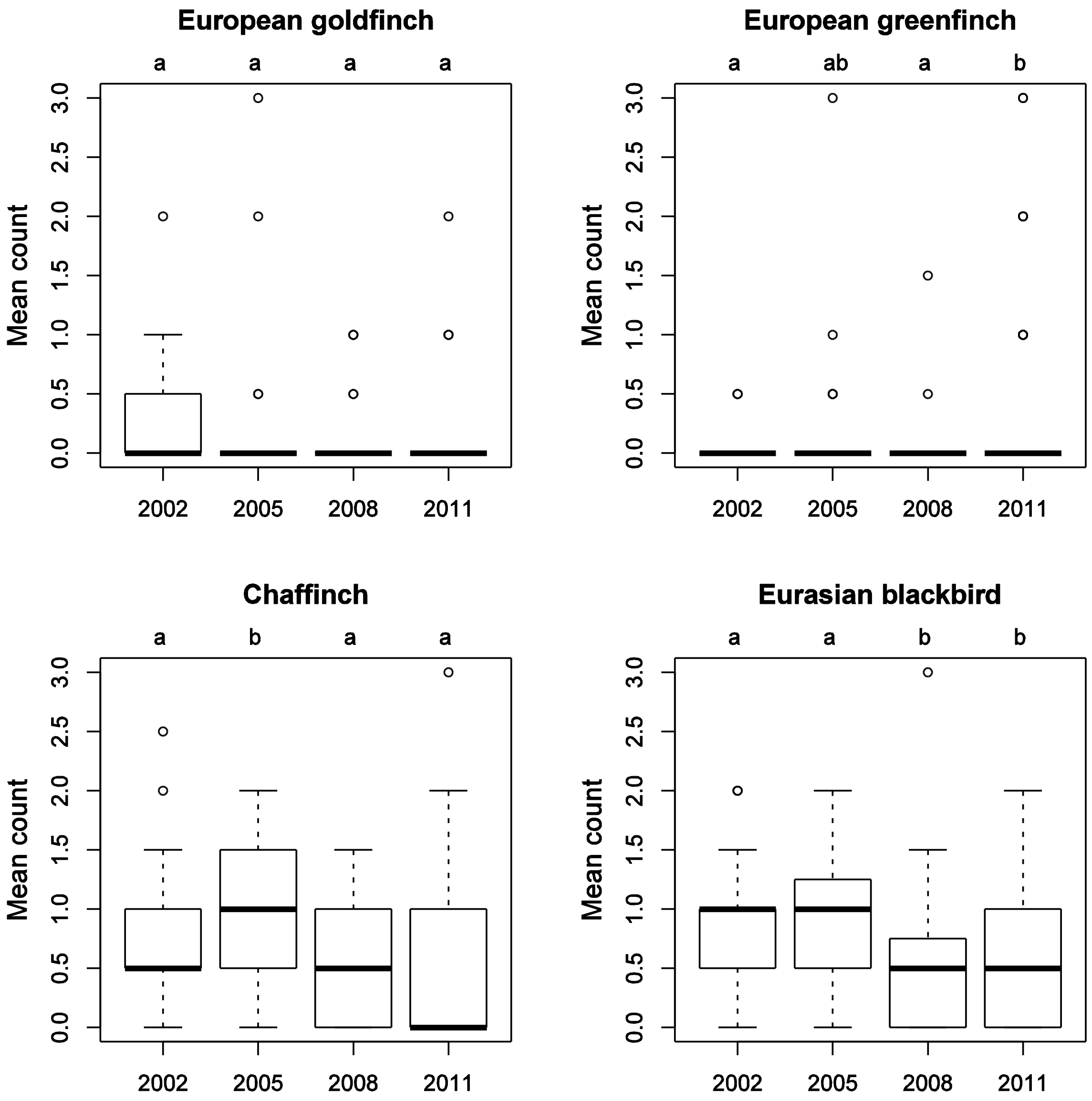

Figure 6 Box plots of mean number of four exotic species; European goldfinch, European greenfinch, chaffinch, and Eurasian blackbird per 5-minute count station at Maungatautari before (2002 and 2005) and after (2008 and 2011) pest mammal eradication. Mean count are not significantly different ($P < 0.05$) in years with the same letter.

5.2 Maximum count of tūī, bellbird, and New Zealand pigeon

The mean maximum number of tūī, bellbirds, and New Zealand pigeons observed *at one time* per 30 minutes was similar at Maungatautari and the non-treatment area in 2002. During subsequent surveys there was some variation in the maximum numbers of the three species, but in general there was no consistent pattern of change (Table 1).

Table 1 Mean (and standard error) of the maximum number of tūī, bellbird, and New Zealand pigeon counted at one time while walking bird count lines at Maungatautari (fenced) and non-fenced sites

Site	Year	Tūī	Bellbird	NZ Pigeon
Non-fenced	2002	1.37 (0.02)	0.35 (0.01)	1.20 (0.03)
Non-fenced	2011	1.75 (0.02)	0.60 (0.04)	0.95 (0.01)
Fenced	2002	1.90 (0.01)	0.70 (0.01)	0.73 (0.01)
Fenced	2005	1.82 (0.01)	0.93 (0.01)	0.43 (0.01)
Fenced	2008	3.31 (0.03)	0.23 (0.01)	0.88 (0.01)
Fenced	2011	2.64 (0.02)	1.00 (0.02)	0.91 (0.02)

6 Discussion and conclusions

Five species—four native (New Zealand pigeon, shining cuckoo, tomtit, tūī) and one exotic (European greenfinch)—were significantly more abundant at Maungatautari in 2011 than in 2002 (before pest eradication). Over the same period, three species—two native (grey warbler and silvereye) and one exotic (Eurasian blackbird)—became less abundant at Maungatautari. However, multiple comparison analysis of all years showed that only tūī and tomtits were significantly more abundant and Eurasian blackbirds less abundant in 2011 than both pre-eradication years.

Analysis of bird counts at non-fenced sites showed two species also increased there—one native (tūī) and one exotic (eastern rosella).

The increase in tūī in all areas is consistent with increases in numbers in other Waikato forests and urban areas (Fitzgerald & Innes 2013, Innes et al. 2013). Tūī respond rapidly and conspicuously to reduced predation by possums and ship rats, and disperse over large distances (10s of km). This leads to considerable ‘spillover’ benefits from pest control areas, but also makes it difficult to ascribe such increases to local pest control operations.

Four species translocated to Maungatautari since pest eradication were detected in our counts in 2011. Although the number of birds is currently too low for statistically significant changes in populations of these species to emerge, their presence alone is an ecologically significant change, contributing to the restoration of ecosystem processes and indigenous dominance.

The apparent decline of blackbirds following pest eradication at Maungatautari could be related to changing interspecific competition. Blackbirds have declined at some other sites of intensive pest control (O’Donnell & Hoare 2012) but not others (Elliott et al. 2010; Smith & Westbrooke 2004), highlighting the complex and site-specific nature of avifaunal response to pest eradication.

The changes in abundance of several bird species at Maungatautari demonstrate a positive effect of pest eradication and fencing on native bird communities. We expect indigenous dominance to increase in future as endemic species increase in abundance and diversity (from reintroduction), and exotic species decline from increasing interspecific competition.

Interpretation of the bird counts is somewhat confounded by the lack of replication of treatments. This is an unavoidable limitation of the novel nature and scale of the Maungatautari Ecological Island project, and is not unusual when entire large ecological systems are being manipulated. Interpretation is further complicated by variable but generally increasing efforts by regional authorities and community groups to control pests for the benefit of native wildlife at many unfenced forests. Nevertheless, these counts provide valuable information on changes in bird abundance at Maungatautari as the forest ecosystem responds to release from exotic mammals over the subsequent 5 years. Changes in bird abundance are likely to be uneven and non-linear as factors other than mammalian predation, for example, inter- and intraspecific competition and annual variation in food availability, cause limitation (Fitzgerald et al. 2009). There is scope to study the factors driving avian community change at pest-free sites. Additional research into plant phenology and nesting success and juvenile dispersal at Maungatautari, and long-term changes in avian community assemblage and abundance at other pest-free sites, such as offshore islands, should be considered as suitable and relevant comparisons. This research would help with our understanding and prediction of avian community change at pest-free mainland sites such as Maungatautari, and the effect of these sites on bird communities in the wider landscape—so-called spillover or halo effects.

The periodic aerial application of 1080 and ground-based use of a variety of other toxins and trapping are now firmly established possum control regimes in native forest in the Waikato Region. While large-scale 1080 can, in addition to reducing possum numbers, result in significant reductions in ship rat populations for up to 2 years (G. Nugent, Landcare Research, Lincoln, pers. comm.), relatively small-scale and infrequent possum control operations may result in higher ship rat abundance for much of the time (Sweetapple & Nugent 2007). The net effect of periodic possum control operations on forest birds in our non-fenced study areas is likely to be much less dramatic than the complete removal of the majority of mammalian pest species at Maungatautari.

Aggregation of birds is driven both by population size and behaviour, so while an increase in the number of birds at Maungatautari is likely to be reflected in larger numbers of these birds being observed at one time, this is confounded by seasonal changes in behaviour. Many non-territorial species flock together during the non-breeding season, particularly around important food resources. For example, New Zealand pigeon historically gathered in mega-flocks of hundreds or thousands of individual birds at important winter food sources (Lyver et al. 2008). Counts conducted in November and December, while appropriate for indices measuring overall abundance, are ostensibly less suitable for measuring changes in flock size of tūī, bellbirds or New Zealand pigeons, so we will not continue these in future. We do not currently intend to instigate a new series of non-breeding flock counts, but if done in the future these should be carried out in late winter for tūī and bellbirds, and autumn for New Zealand pigeon

Comparisons between pest-fenced areas where mammals are eradicated, and non-fenced areas where pest control may vary and never achieve complete eradication of most pest species is a relevant reflection of current and anticipated future conservation efforts on mainland New Zealand. Large-scale, complete eradication and exclusion of pests and the subsequent reintroduction of locally extinct native taxa are currently the most intensive and comprehensive level of ecosystem restoration undertaken. However, questions have been raised about the costs and benefits of such projects (Scofield et al. 2011), so it is important to investigate differences that might accrue at pest-fenced sites relative to other, less intensive

conservation efforts, particularly as the period of monitoring increases and episodic fluctuations can be separated from long-term trends.

7 Recommendations

- 5-minute bird counts should be repeated in November–December 2014, at Maungatautari and non-treatment sites, to monitor changes in avifauna resulting from eradication of mammalian pests at Maungatautari.
- Maximum counts of tūī, bellbirds, and New Zealand pigeon should not be repeated in the breeding season in future. We are not currently planning to start a new series of non-breeding flock counts. However, if done in the future these should be carried out in late winter for tūī and bellbirds, and autumn for New Zealand pigeon.
- There is scope to further investigate mechanisms and pattern of avian response to release from mammalian predation. This could include:
  - conducting bird counts at other pest-free sites to give examples of long-term dynamics of avian assemblages and abundance change at sites with high (fenced sanctuaries) and low (offshore islands) potential for avian dispersal. Does dispersal limit avian recovery in mainland sanctuaries?
  - comparing breeding productivity and success at Maungatautari and non-fenced sites to help quantify increased recruitment to the local population and spillover to more distant areas.

8 Acknowledgements

We would like to thank Bill and Sue Garland for allowing access across their property adjoining Maungatautari to conduct bird counts. In addition to the authors, bird counts were carried out by Corinne Watts, Danny Thornburrow, Scott Bartlam, and Sam Cave. Guy Forrester and Lucy Bridgman gave valuable advice and assistance with statistical analysis of the data. Comments from Norman Mason substantially improved the manuscript. This research was supported by core funding for Crown Research Institutes from the Ministry of Business, Innovation and Employment's Science and Innovation Group.

9 References

Allan Herbarium 2000. New Zealand Plant Names Database. Landcare Research, New Zealand. http://nzflora.landcareresearch.co.nz/ (accessed 18 June 2012).

Clarkson B, Merrett M, Downs T comp. 2002. Botany of the Waikato. Hamilton, Waikato Botanical Society Inc., University of Waikato. 136 p.

Dawson D, Bull P1975. Counting birds in New Zealand forests. Notornis 22: 101–109.

de Mendiburu F 2013. agricolae: Statistical Procedures for Agricultural Research. R package version 1.1–4. http://CRAN.R-project.org/package=agricolae

Elliott P, Wilson P, Taylor R, Beggs J 2010. Declines in common native birds in a mature temperate forest. Biological Conservation 143: 2119–2126.

Fitzgerald N, Innes J 2013. Hamilton City bird counts: 2004–2012. Landcare Research Contract Report LC1484 for Hamilton City Council.

Fitzgerald N, Innes J, Forrester G 2009. Changes in bird counts at Maungatautari after pest mammal eradication: results to 2008. Landcare Research Contract Report 0910/044 for the Foundation for Research, Science and Technology.

Gill B ed. 2010. Checklist of the birds of New Zealand, Norfolk and Macquarie islands, and the Ross Dependency, Antarctica. Wellington, Te Papa Press in association with the Ornithological Society of New Zealand. 500 p.

Gillies C, Pierce R 1999. Secondary poisoning of mammalian predators during possum and rodent control operations at Trounson Kauri Park, Northland, New Zealand. New Zealand Journal of Ecology 23: 183–192.

Hartley L 2012. Five-minute bird counts in New Zealand. New Zealand Journal of Ecology 36: 268–278.

Innes J, Burns B, Fitzgerald N, Thornburrow D, Watts C 2003. Pre-mammal eradication bird counts at Maungatautari and Pirongia, November–December 2002. Landcare Research Contract Report 0203/095 for the Foundation for Research, Science and Technology.

Innes J, Fitzgerald N, Bartlam S, Watts C 2013. Bird counts in Waikato Halo blocks, November 2012. Landcare Research Contract Report LC1488 for Waikato Regional Council.

Innes J, Fitzgerald N, Thornburrow D, Watts C, Burns B 2006. Further bird counts at Maungatautari and Pirongia, December 2005. Landcare Research Contract Report 0506/149 for the Foundation for Research, Science and Technology.

Innes J, Fitzgerald N, Watts C, Thornburrow D, Blackwell H, Lancaster E, Burns B 2005. Distribution, movements, and nesting success of Waikato tūī. Abstract of paper presented at the Ornithological Society of New Zealand AGM and Conference, 5 June 2005, Hamilton, New Zealand. Notornis 52: 173.

Innes J, Kelly D, Overton J, Gillies C 2010. Predation and other factors currently limiting New Zealand forest birds. New Zealand Journal of Ecology 34: 86–114.

Innes J, Saunders A 2011. Eradicating multiple pests: an overview. In: Veitch CR, Clout MN, Towns DR eds. Island invasives: eradication and management. Gland, Switzerland, IUCN. Pp. 177–181.

Innes J, Warburton B, Williams D, Speed H, Bradfield P 1995. Large-scale poisoning of ship rats in North Island indigenous forests. New Zealand Journal of Ecology 19: 5–17.

King CM ed. 2005. The handbook of New Zealand mammals. Oxford University Press. 630 p.

Lyver P, Taputu T, Kutia S, Tahi B 2008. Tūhoe Tuawhenua mātauranga of kererū (*Hemiphaga novaseelandiae novaseelandiae*) in Te Urewera. New Zealand Journal of Ecology 32: 7–17.

Murphy E, Robbins L, Young J, Dowding J 1999. Secondary poisoning of stoats after an aerial 1080 poison operation in Pureora Forest, New Zealand. New Zealand Journal of Ecology 23: 175–182.

Nicholls JL 1979. Waikato Forest Class Map. Forest Service Mapping Series 6. Wellington, New Zealand Forest Service.

O'Donnell CFJ, Hoare JM 2012. Quantifying the benefits of long-term integrated pest control for forest bird populations in a New Zealand rainforest. New Zealand Journal of Ecology 36: 131–140.

R Core Team 2013 R: A language and environment for statistical computing. R Foundation for Statistical Computing, Vienna, Austria. http://www.R-project.org/.

Scofield PR, Cullen R, Wang M 2011. Are predator-proof fences the answer to New Zealand's terrestrial faunal biodiversity crisis? New Zealand Journal of Ecology 35: 312–317.

Smith A, Westbrooke I 2004. Changes in bird conspicuousness at Pureora Forest. Notornis 51: 21–25.

Speedy C, Day T, Innes J 2007. Pest eradication technology – the critical partner to pest exclusion technology: the Maungatautari experience. In: Witmer GW, Pitt WC, Fagerstone KA eds. Managing Vertebrate Invasive Species: Proceedings of an International Symposium. Fort Collins, CO, USDA/APHIS/WS, National Wildlife Center. Pp.155–126.

Sweetapple PJ, Nugent G 2007. Ship rat demography and diet following possum control in mixed podocarp-hardwood forest. New Zealand Journal of Ecology 31: 186–201.

Appendix 1 – Mean number of birds per 5-minute count

Table 2 Mean numbers of birds counted per 5-minute count stations at non-fenced (Pirongia and Hakarimata) and fenced (Maungatautari) sites in November–December counted in 2002, 2005, 2008, and 2011, and 2011 counts at all stations including new baseline stations (*). + Indicates species reintroduced to Maungatautari since 2006

	Non-fenced Mean count (standard error)					Fenced Mean count (standard error)				
**Species**	**2002**	**2005**	**2008**	**2011**	**2011***	**2002**	**2005**	**2008**	**2011**	**2011***
Australian magpie	0.05 (0.05)	0.06 (0.06)	0.06 (0.06)	0 (0)	0.03 (0.02)	0.13 (0.05)	0.09 (0.05)	0.09 (0.05)	0.04 (0.04)	0.05 (0.02)
Bellbird	0.02 (0.02)	0.06 (0.06)	0.11 (0.07)	0.14 (0.08)	0.09 (0.04)	0.5 (0.1)	0.47 (0.09)	0.39 (0.12)	0.62 (0.13)	0.6 (0.08)
Chaffinch	0.4 (0.13)	0.33 (0.19)	0.44 (0.15)	0.36 (0.08)	0.43 (0.06)	0.69 (0.1)	1.06 (0.1)	0.67 (0.09)	0.64 (0.11)	0.77 (0.07)
Common myna	0 (0)	0 (0)	0 (0)	0.05 (0.05)	0.05 (0.03)	0.04 (0.02)	0 (0)	0 (0)	0.02 (0.02)	0.02 (0.01)
Common starling	0 (0)	0 (0)	0 (0)	0.07 (0.07)	0.03 (0.03)	0 (0)	0 (0)	0 (0)	0.04 (0.03)	0.03 (0.01)
Dunnock	0 (0)	0 (0)	0 (0)	0 (0)	0 (0)	0.01 (0.01)	0 (0)	0 (0)	0 (0)	0 (0)
Eastern rosella	0.05 (0.05)	0.06 (0.06)	0.22 (0.17)	0.26 (0.08)	0.22 (0.05)	0.34 (0.08)	0.34 (0.08)	0.31 (0.09)	0.28 (0.08)	0.39 (0.06)
Eurasian blackbird	0.38 (0.12)	0.28 (0.15)	0.39 (0.11)	0.24 (0.07)	0.3 (0.05)	0.83 (0.09)	0.91 (0.09)	0.54 (0.1)	0.56 (0.09)	0.63 (0.06)
European goldfinch	0.1 (0.07)	0.39 (0.2)	0.06 (0.06)	0.07 (0.04)	0.09 (0.03)	0.2 (0.07)	0.17 (0.1)	0.11 (0.05)	0.1 (0.05)	0.21 (0.04)
European greenfinch	0.05 (0.05)	0 (0)	0 (0)	0.05 (0.03)	0.07 (0.03)	0.04 (0.02)	0.16 (0.09)	0.06 (0.04)	0.34 (0.11)	0.39 (0.06)
Grey warbler	1.23 (0.13)	0.83 (0.17)	1.17 (0.2)	0.98 (0.09)	1.17 (0.07)	1.97 (0.08)	1.24 (0.1)	1.6 (0.13)	1.5 (0.11)	1.44 (0.06)
House sparrow	0 (0)	0 (0)	0 (0)	0.02 (0.02)	0.01 (0.01)	0 (0)	0 (0)	0 (0)	0 (0)	0 (0)
Kaka+	0 (0)	0 (0)	0 (0)	0 (0)	0 (0)	0 (0)	0 (0)	0.04 (0.03)	0 (0)	0.01 (0.01)
Long-tailed cuckoo	0 (0)	0 (0)	0 (0)	0 (0)	0.01 (0.01)	0 (0)	0 (0)	0 (0)	0 (0)	0 (0)
Morepork	0 (0)	0 (0)	0 (0)	0 (0)	0 (0)	0 (0)	0 (0)	0.01 (0.01)	0 (0)	0.01 (0.01)
New Zealand falcon	0 (0)	0 (0)	0.06 (0.06)	0 (0)	0 (0)	0 (0)	0 (0)	0 (0)	0 (0)	0.02 (0.01)
New Zealand fantail	0.68 (0.16)	0.39 (0.14)	0.5 (0.2)	0.48 (0.13)	0.33 (0.06)	0.71 (0.1)	1.07 (0.13)	0.53 (0.09)	0.58 (0.1)	0.66 (0.06)
New Zealand kingfisher	0.68 (0.14)	0.22 (0.12)	0.33 (0.17)	0.55 (0.11)	0.38 (0.06)	0.3 (0.06)	0.19 (0.05)	0.29 (0.07)	0.3 (0.09)	0.3 (0.05)

New Zealand pigeon	0.2 (0.08)	0.33 (0.17)	0.39 (0.16)	0.21 (0.06)	0.19 (0.04)	0.27 (0.06)	0.37 (0.1)	0.27 (0.08)	0.6 (0.15)	0.64 (0.07)
North Island robin+	0 (0)	0 (0)	0 (0)	0 (0)	0 (0)	0 (0)	0 (0)	0 (0)	0.02 (0.02)	0.01 (0.01)
Rifleman	0 (0)	0.11 (0.11)	0.39 (0.26)	0.05 (0.05)	0.08 (0.04)	0 (0)	0 (0)	0 (0)	0 (0)	0 (0)
Shining cuckoo	0.45 (0.17)	0.11 (0.07)	0.44 (0.15)	0.48 (0.09)	0.32 (0.05)	0.4 (0.06)	0.33 (0.06)	0.83 (0.13)	0.64 (0.09)	0.61 (0.05)
Silvereye	1.88 (0.26)	0.44 (0.13)	1.67 (0.17)	1.29 (0.15)	1.01 (0.11)	1.76 (0.16)	1.46 (0.19)	1.3 (0.15)	1.04 (0.15)	0.97 (0.09)
Song thrush	0 (0)	0 (0)	0 (0)	0.02 (0.02)	0.03 (0.02)	0.06 (0.03)	0 (0)	0.04 (0.02)	0.02 (0.02)	0.02 (0.01)
Stitchbird+	0 (0)	0 (0)	0 (0)	0 (0)	0 (0)	0 (0)	0 (0)	0 (0)	0.04 (0.03)	0.04 (0.02)
Swamp harrier	0 (0)	0 (0)	0 (0)	0 (0)	0.02 (0.01)	0.04 (0.02)	0.03 (0.02)	0 (0)	0.02 (0.02)	0.03 (0.01)
Tomtit	0.28 (0.11)	0.56 (0.18)	0.72 (0.15)	0.29 (0.09)	0.45 (0.07)	0.33 (0.06)	0.84 (0.11)	1.23 (0.16)	1.34 (0.13)	1.19 (0.08)
Tūī	0.82 (0.21)	0.5 (0.14)	1.5 (0.25)	1.38 (0.12)	1.49 (0.1)	1.87 (0.15)	2.23 (0.15)	3.5 (0.22)	3.76 (0.19)	3.41 (0.1)
Welcome swallow	0 (0)	0 (0)	0 (0)	0.02 (0.02)	0.06 (0.03)	0 (0)	0.06 (0.06)	0 (0)	0 (0)	0 (0)
Whitehead+	0 (0)	0 (0)	0 (0)	0 (0)	0 (0)	0 (0)	0 (0)	0 (0)	0.12 (0.07)	0.04 (0.02)
Yellowhammer	0 (0)	0 (0)	0 (0)	0 (0)	0.03 (0.02)	0.01 (0.01)	0 (0)	0.01 (0.01)	0 (0)	0.01 (0.01)

Appendix 2 – Scientific names of birds used in text

Table 3 Scientific and common names of birds used in text. Bird names follow Gill (2010)

Common name in text	Scientific name	Order	Origin
Australian magpie	*Gymnorhina tibicen*	Passerine (perching) birds	Introduced
Bellbird	*Anthornis melanura*	Passerine (perching) birds	Native
Chaffinch	*Fringilla coelebs*	Passerine (perching) birds	Introduced
Common myna	*Acridotheres tristis*	Passerine (perching) birds	Introduced
Common pheasant	*Phasianus colchicus*	Game birds and allies	Introduced
Common starling	*Sturnus vulgaris*	Passerine (perching) birds	Introduced
Dunnock	*Prunella modularis*	Passerine (perching) birds	Introduced
Eastern rosella	*Platycercus eximius*	Cockatoos, parrots and parakeets	Introduced
Eurasian blackbird	*Turdus merula*	Passerine (perching) birds	Introduced
European goldfinch	*Carduelis carduelis*	Passerine (perching) birds	Introduced
European greenfinch	*Carduelis chloris*	Passerine (perching) birds	Introduced
Grey warbler	*Gerygone igata*	Passerine (perching) birds	Native
House sparrow	*Passer domesticus*	Passerine (perching) birds	Introduced
Kaka	*Nestor meridionalis*	Cockatoos, parrots and parakeets	Native
Long-tailed cuckoo	*Eudynamys taitensis*	Cuckoos	Native
Morepork	*Ninox novaeseelandiae*	Owls	Native
New Zealand falcon	*Falco novaeseelandiae*	Falcons	Native
New Zealand fantail	*Rhipidura fuliginosa*	Passerine (perching) birds	Native
New Zealand kingfisher	*Todiramphus sanctus*	Kingfishers and allies	Native
New Zealand pigeon	*Hemiphaga novaeseelandiae*	Pigeons and doves	Native
North Island robin	*Petroica longipes*	Passerine (perching) birds	Native
Paradise shelduck	*Tadorna variegata*	Duck-like birds	Native
Rifleman	*Acanthisitta chloris*	Passerine (perching) birds	Native
Shining cuckoo	*Chrysococcyx lucidus*	Cuckoos	Native
Silvereye	*Zosterops lateralis*	Passerine (perching) birds	Native
Song thrush	*Turdus philomelos*	Passerine (perching) birds	Introduced
Swamp harrier	*Circus approximans*	Hawks and allies	Native
Tomtit	*Petroica macrocephala*	Passerine (perching) birds	Native
Tūī	*Prosthemadera novaeseelandiae*	Passerine (perching) birds	Native
Welcome swallow	*Hirundo neoxena*	Passerine (perching) birds	Native
Whitehead	*Mohoua albicilla*	Passerine (perching) birds	Native
Yellowhammer	*Emberiza citrinella*	Passerine (perching) birds	Introduced

Appendix 3 – Scientific names of mammals used in text

Table 4 Scientific and common names are from King (2005)

Common name in text	Scientific name
Cat	*Felis catus*
Brushtail possum	*Trichosurus vulpecula*
House mouse	*Mus musculus*
Ship rat	*Rattus rattus*
Stoat	*Mustela erminea*

Appendix 4 – Scientific names of plants used in text

Table 5 Scientific and common names are from Allan Herbarium (2000)

Common name in text	Scientific name
Kāmahi	*Weinmannia racemosa*
Miro	*Prumnopitys ferruginea*
Rimu	*Dacrydium cupressinum*
Tawa	*Beilschmiedia tawa*
Tāwari	*Ixerba brexioides*
Tāwheowheo	*Quintinia serrata*
