# Supplementary material for: Responses of New Zealand forest birds to management of introduced mammals
Source: Conserv Biol. 2020 Mar 23;35(1):35–49. doi: 10.1111/cobi.13456 (PMC7984369; doi:10.1111/cobi.13456)
Supplement: Supplementary file 5 — Supporting Material [file COBI-35-35-s003.pdf]

1. Introduction

This report covers the period 1 July 2005 to 30 June 2006, and is the eighth report written for Boundary Stream Mainland Island (subsequently referred to as BSMI) which is in its tenth year of operation.
Ecological restoration at BSMI continues to include multi-species pest management and multi-species or ecosystem focused result and outcome monitoring to measure the effect of management as outlined in Section 1 of the BSMI 1998-2000 Project Report. Methods of control and monitoring continue to be revised and improved to ensure effective and cost-efficient techniques and integrated pest management regimes.

1.1 Management objectives

The management objectives for BSMI defined in the strategic plan (Adams 1997) are as follows:

5.1 Ecosystem recovery: *The recovery of forest structure and ecosystem processes by the control to low levels, and where possible the elimination of animal and plant pests and the exclusion of domestic farm animals.*

5.2 Monitoring: *Monitoring of key environmental factors to establish baseline information and measure changes resulting from enhancement activities.*

5.3 Threatened species recovery: *The recovery of resident threatened species e.g. kakabeak, yellow-flowered mistletoe, kiwi, kereru, etc and the determination of the status of threatened species known to be present, and the detection of other species that may be present.*

5.4 (Re) Introductions: *The reintroduction of species formerly present or at risk in the region, e.g. North Island brown kiwi, North Island robin, North Island saddleback, North Island kokako, Pittosporum obcordatum, Dactylanthus taylorii, and Powelliphanta traversii 'Maungaharuru', etc.*

5.5 Research: *The encouragement and support of research (both within the Department and externally) on all aspects of the project.*

5.6 Community Interest: *The encouragement of local community and interest group involvement and assistance in the restoration programs i.e. neighbours, schools/polytechnics/universities, conservation groups, tangata whenua, businesses, etc.*

5.7 Sharing Knowledge: *The acquiring of knowledge by all those involved in the project and the sharing of the successes and failures for the benefit of like projects elsewhere.*

5.8 Infrastructure: *The maintenance of an infrastructure and the employment of suitable staff to effectively manage the project at a high level of professionalism and expertise.*
5.9 Staff Development: *The provision of opportunities for Conservancy staff development via the project through training, exchanges, secondments and redirection.*

1.2 Report scope and structure

This project report comprises the progress of pest control work undertaken at BSMI and subsequent result monitoring and outcome monitoring in BSMI and in the Comparison sites - Thomas' Bush and Cashe's Bush, from July 2004 to June 2005.

Aspects of the project not included within this report are:

- weed management
- wasp management
- climate data
- track management
- plant (base facilities) maintenance
- survey and conservation work on the native land snail (*Powelliphanta traversii*) within the Comparison site
- bat monitoring
- financial summaries
- strategic planning and management
- details of other reports or communication with other conservation practitioners or
- staff development and training

This report is set out in six chapters including this introduction, and a chapter including acknowledgements and references. Each Section within chapters 2, 3, 4 and 5 details specific short-term and long-term objectives which relate to the management objectives set out in Section 1.1 as above. Short-term objectives refer to those addressed in this report, while long-term objectives are those objectives which are to be met in a timeframe extending beyond this reporting period.

Chapters 2-5 include the following:

Chapter 2 details pest control methods and result monitoring across BSMI and Comparison sites, for possums and rodents, mustelids, ungulates and cats.

Chapter 3 details outcome monitoring results for general birds, vegetation and threatened plants, lizards, and invertebrate community monitoring, relating changes in the condition of these conservation assets to specific pest management programs where possible, or to general ecosystem recovery.

Chapter 4 describes the methods and progress of North Island robin, North Island brown kiwi, North Island kokako, and North Island saddleback reintroductions to BSMI.

Chapter 5 describes public awareness, community participation and research projects that are in progress at BSMI.

2. Multi-species pest management: pest control and result monitoring

2.1 Possum and rodent control

2.1.1 Summary

Possum indices in Boundary Stream Mainland Island (BSMI) were maintained below the target Wax Tag® index of 3% over the past year. On the other hand, new random tracking tunnel lines set up in December/January 2006 revealed that rat activity within BSMI was much higher than generally thought. This discovery raised some questions about the existing poison operation – the choice of poison and bait station spacing in particular. Increasing the internal bait station spacing from 150m x 150m to 75m x 150m (perimeter bait station spacing remain 100m) is recommended for the future rodent control operation.

2.1.2 Objectives

Short-term objectives

- To maintain possum Wax Tag® indices at < 3% and rat tracking indices at < 5% in BSMI.
- To assess the effectiveness of different baits and the bait station regime for controlling possums and rodents.
- To investigate the effectiveness of alternative methods of monitoring possums and rats (i.e. wax blocks, different tracking tunnel types and placements).

Long-term objectives

- To control possum and rat numbers to low levels sufficient to allow 'ecological restoration' of forest structure, indigenous biota and ecosystem processes.
- To provide a safe environment for the reintroduction of North Island robin, and North Island kokako.
- To establish meaningful target indices for possum and rodent control in the context of ecosystem management.

2.1.3 Methods

- **Possum and rodent control**

Philproof Feeder bait stations (Philproof Pest Control Products, P.O. Box 4385, Hamilton) set every 150m x 150m within the Reserve (358 internal stations) and stations spaced every 100m around the forest boundary (209 perimeter stations and 21 stations on Pohokura Road).

Rodenticide was placed in all internal and perimeter bait stations. Internal stations were checked and refilled if necessary every eight weeks and the perimeter stations monthly. Feratox (100mg of cyanide in a 20g ferafeed block) was also used in all perimeter bait stations to control possums invading from the buffer area.

Ditrac® (Diphacinone block, 0.05g/kg) was used to control rodents between June 2005 and February 2006. Nine blocks of Ditrac® were strung on wire into a 'necklace' and attached to each station to prevent the removal of whole baits. Each necklace was replaced if there were equal to or less than four baits left on the wire. Due to the cost of each Ditrac® block, baits were recycled unless they were mouldy.

In March 2005, the Ditrac® blocks were replaced with Racumin (another first generation anticoagulant - Coumatetralyl 0.375g/kg) paste. Racumin comes in 100g bags, three of these (300g) were enclosed in plastic ziplock bags which were stapled 30cm above the ground onto a tree or a post at 50m intervals between the existing bait stations. This was carried out on both internal and perimeter bait-station lines. The amount of bait was reduced to 200g per ziplock bag in May 2006.

- **Result monitoring**

Rodents

Tracking tunnels were used to index rodent activity in BSMI and the two Comparison sites. It has been noted, however, that some of the existing lines in BSMI are not randomly set up since they run along bait station lines (see Appendix 5). Between December 2005 and January 2006, 12 new random lines were established within BSMI (see Appendix 5) to detect the rodent activity more accurately. GPS coordinates of the start and end of each line randomly generated by the computer were used for this purpose. Setting up of these new lines was based on the tracking tunnels best practice (OLDDM-118330). Both old and new rodent lines within BSMI were run in February, April and May 2006 to compare the detection rates (expressed as the % of all tunnels that contained rat or mouse tracks).

Possums and rats are also caught in mustelid traps as by-catch (see also section 2.2). The data such as capture location and numbers may provide information on relative abundance and seasonal population change of these species.

Possum

Wax Tags® were used to monitor possum abundance within BSMI in June 2006. Line set up and practice refers to the 'Protocol for low-density possum monitoring using the WaxTag method' (National Possum Control Agencies, 2005, ISBN 0-9583736-2-0). The twelve new tracking tunnel lines established in February 2006 were also used as possum monitoring lines since they are random and meet the criteria stated in the Protocol.

Wax Tags® and luminescent strip were nailed to a tree approximately 30cm above the ground. Each Wax Tag® line consisted of 20 tags placed at 10m intervals. The date, line number and tag number were written down on the back of each Wax Tag® with a permanent waterproof marker. Wax Tags® were put out in the field on 16th June 2006 for seven nights. However, due to heavy snow falls (22nd June), they were not collected until 29th June 2006. All bite marks on Wax Tags® were identified and recorded onto a standard excel spread sheet (see the protocol). After bite mark analysis was completed, Possum Activity Index (PAI) was then calculated with 95% confidence intervals, using the formula provided in the Protocol.

2.1.4 Results

- **Poison operation**

Bait take remained high throughout the year regardless of the type of bait used. High pig interference on zip-locked Racumin bags was noted as concern. Also, some Racumin baits were going mouldy quickly due to moisture penetrating from small holes made by mice and rats on the zip-lock bags.

- **Monitoring**

Possum monitoring

No possum bite marks were found on any Wax Tags® (PAI = 0%). One had mouse bite marks (Line 8 in the 'Cecilies' block) and another had rat marks (Line 12 in the 'Te Tatimana' block). Three Wax Tags® were either damaged or lost due to heavy snow.

Rodent tracking indices

Rat indices have remained below the target level of 5% and well below the level in the Comparison sites (see Figure 1). However, the new random lines indicated that greater number of rats are present within BSMI. In February 2006, the rat indices for new random lines reached 15%. Following the introduction of Racumin in March 2006, the rat indices dropped to 6.7% although the number crept up again to 9.2% in May 2006.

Mouse indices were consistently high in both BSMI and the Comparison sites throughout the year. The difference in mouse activities between BSMI and the Comparison sites was significant only in May 2006. There was no significant difference in mouse detection between old and new lines within BSMI.

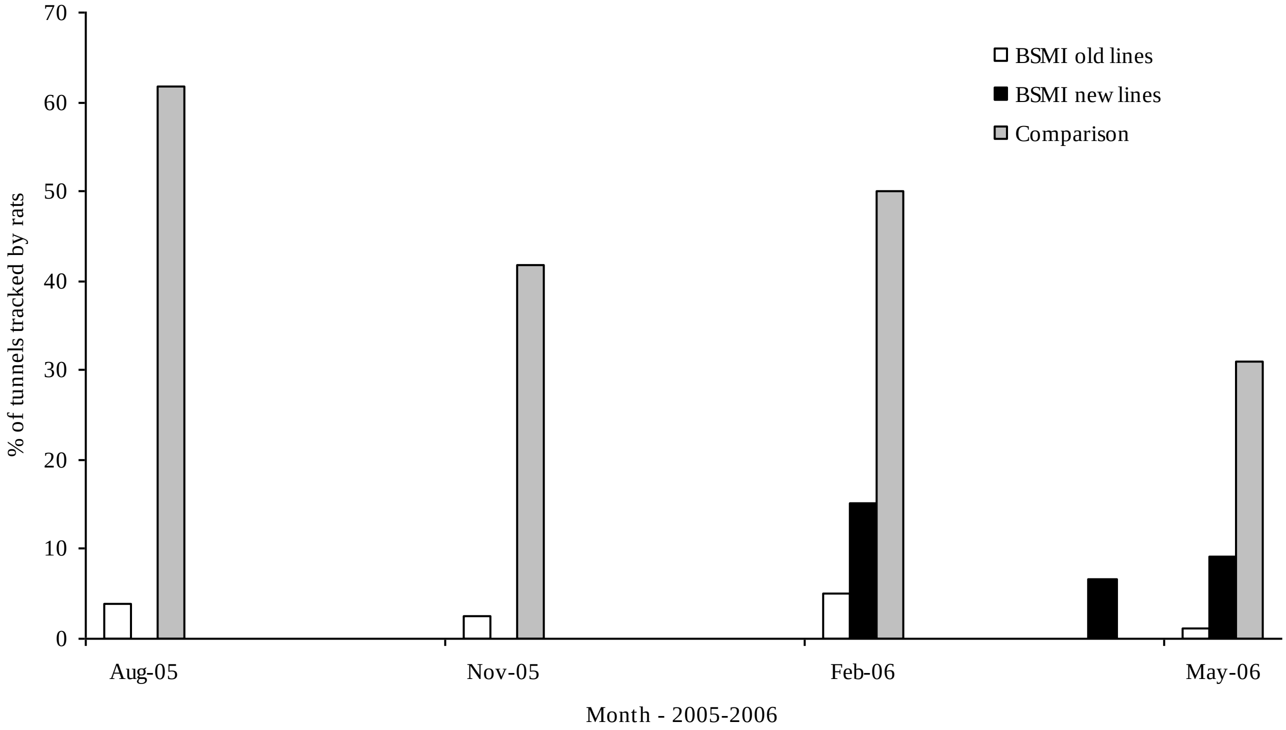

Figure 2.1.1 Rat indices results from BSMI and comparison sites from 2005-2006.

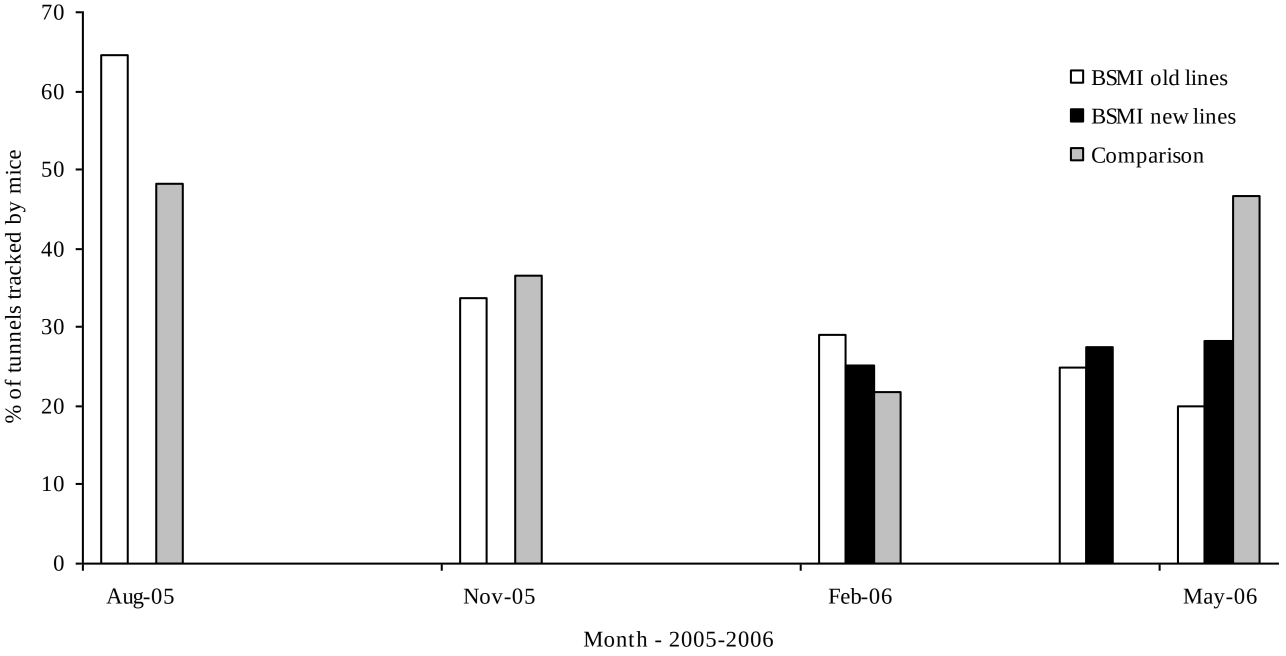

Figure 2.1.2 Mouse indices results from BSMI and comparison sites from 2005-2006.

Rat trap catch

One possum, 377 rats and 16 mice were caught as by-catch in mustelid Fenn and DOC 200 trap sets within and around the Reserve (see section 2.2). Of 377 rats, 151 were caught inside the Reserve ('Shines Falls' and 'Te Tatimana/Podocarps' trap lines scored the highest number of rats) (see Appendix 8 - Trap Line Inventory and Map) and the remainder on the perimeter and buffer trap lines.

2.1.5 Discussion

The new random tracking tunnel lines gave us a fresh insight into rat and mouse activity within BSMI. These new lines proved to be more sensitive in detecting relative abundance of rodents, and therefore, should replace old lines.

Ongoing possum and rat control continues to ensure the ecosystem restoration process within BSMI, in particular the recovery of North Island robin and North Island kokako population (see sections 4.1 and 4.3).

Feratox continues to be effective at controlling possums on the perimeter. Although the Waxtag® result was not conclusive due to weather interference (heavy snowfall during the monitoring period), it appears that possum numbers have been kept low inside the Reserve. Waxtag® is easy to use, relatively cheap ($0.85/tag), has a long storage life and has no risk of injuring non-target species. Therefore, Waxtag® should continue to be used annually for auditing possum activities within BSMI instead of conventional residual trap capture method using leg-hold traps.

Ditrac was less successful in controlling rats – as the rodent indices increased well above our target levels in February 2006. However, this may be as a result of recycling old baits. The current DOC best practice on rat control states that reusing of baits is not recommended, and this protocol should be followed in the future.

Racumin was relatively effective in reducing rat numbers although the rat indices remained above the target level of 5%. It seems that various factors such as pig interference on baits and bait palatability and life after zip-lock bags were nibbled might have affected the bait availability for rats. It is therefore recommended that Racumin baits should be placed only in weather-proofed stations (ie. Philproof bait station). The existing placement of stations was originally chosen to control possums, not rodents. The upsurge in rodent activity in February 2006 may indicate that the placement needs to be reviewed. According to the DoC best practice, placing should be no greater than 100m x 150m. Considering the resources and labour required for re-spacing bait stations as well as servicing them bimonthly, 75m x 150m spacing is the most practical option. The increase in bait station numbers would further enhance forest recovery and protection of native species.

2.1.6 Recommendations

- Use new random tracking tunnel lines instead of old lines, and retrieve old tunnels from the field when possible.

- Increase bait station spacing to 75m x 150m and continue monitoring the effects of Racumin baits on rodent abundance.
- Do not put bait out in plastic bags in presence of pigs.

References

Gillies, C., Williams, D. *Using tracking tunnels to monitor rodents and mustelids*. OLDDM-118330

National Possum Control Agencies. (2005). *Protocol for low-density possum monitoring using the WaxTag® method.* ISBN 0-9583736-2-0

2.2 Mustelid

2.2.1 Summary

The mustelid trapping regime at Boundary Stream Mainland Island (BSMI) continues to be an essential part of the project. Traps were serviced fortnightly for all internal and buffer lines and monthly for the perimeter line with bait types used being egg, fresh rabbit or salted rabbit. Three new DOC 200 double set trap lines have been installed within the Reserve to provide a more effective trapping regime. There was a dramatic 53% decrease in the number of stoats caught this period (47) compared to the previous reporting period (88). Stoats were caught steadily throughout the period with a peak occurring in February 2006. Four ferrets where caught this period, two on the perimeter and two on the buffer. Weasel captures have remained the same compared with the previous season although more were caught inside the Reserve. With suspicion that the original tracking tunnel lines set out in 2000 were not randomly placed, seven new lines were established within BSMI and first run in February 2006. Mustelid indicies maintained below 3% throughout the season, compared with up to 17% in the two Comparison sites. A stoat dog has been also used as a new tool to detect and control mustelids within the Reserve.

2.1.2 Objectives

Short term objectives

- To investigate differences in the trapped population of mustelids at BSMI determining differences between captures on the buffer, perimeter and internal trap lines.

Long-term objectives

- To increase the efficiency and cost effectiveness of mustelid control
    1. Investigating when control can be increased or reduced
    2. Improving trapping techniques (eg. bait type, trap type, trap location)
    3. Investigating alternative control options which may be beneficial to BSMI
       and contribute to research nationwide.

- To control mustelid numbers to a tracking level which will allow the survival and recovery of indigenous animal populations and ecological processes for which mustelids are a cause of decline.

- To provide a safe environment for current and proposed native species re-introductions (e.g., North Island kiwi, North Island kokako and North Island saddleback).

2.2.3 Methods

- ***Mustelid control***

Operation of the trap lines is separated into three key areas; internal lines (9) set within the forested area of the reserve itself, a single perimeter line around the forest edge and buffer
Lines (6) set throughout adjacent farmland and along the main road, (Refer to Appendix – Trap line Inventory and Map)

Internal trap lines

There have been additions to the previous 2004/05 trap layout with 3 new DOC 200 double set trap lines being installed (see photos); 25 sets through the Wallow/Cecilies block (July 2005), 26 sets through the Section Four/Kiwi block(Jan. 2006) and 19 sets along the Goat Hill track off Kamahi loop. MK VI Fenn trap lines along the Bell Rock Track, Main Walkway and Beech Ridge lines were baited with a fresh egg. All DOC 200 traps were baited with both egg and small piece of fresh rabbit. All traps were checked fortnightly.

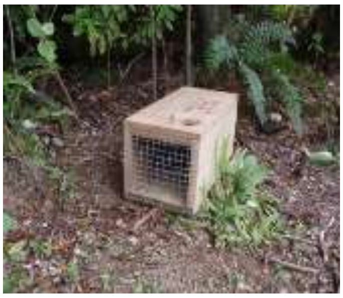
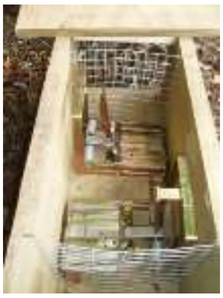

Wooden trap box fitted with double set Doc200 traps

Buffer trap lines

There has been one additional line of 14 double set Doc 200 traps placed through Naumai farm and baited with both egg and fresh rabbit. All MK VI lines were baited egg only, these being Woodstock, Pohokura Road and Bush Track, Rangi Pines and Thomas Bush. All buffer traps were checked fortnightly.

Perimeter trap line

Double set Mark IV Fenn traps have been operational in wooden boxes since March 2001. Trap placement is width ways across the inside of each box. The egg and salted rabbit meat bait trial that began in December 2004 was continued throughout this

period. The salted rabbit baits were made by soaking fresh rabbit meat for four days in a mix of one part salt to eight parts water. Odd numbered traps were baited egg only, with even numbered traps baited salted rabbit only. All perimeter traps were checked monthly and the old egg and salted rabbit bait were replaced.

All trapped mustelids were brought back to the field base for taking measurements, weighing, sexing and aging unless the body was too decomposed. The ratio of the length of the inter-orbital and post-orbital process (BSMI 2001/02 Annual Report) was used to age stoats in combination with weight and baculum size.

- ***Mustelid Monitoring***

The use of tracking tunnels to provide an indication of mustelid activity in BSMI and the two Comparison sites continued. It was suspected that the original tracking tunnel lines set out in 2000 were not randomly placed and hence not sensitive enough to detect mustelid activity correctly. To address this issue, seven new randomly placed mustelid tracking lines were established within BSMI in November 2005 to January 2006 (see Appendix for location of new tracking tunnel lines). GPS coordinates of the start and end of each line randomly generated by the computer were used for this purpose. Setting up of these new lines was based on the tracking tunnels best practice (OLDDM-118330). Both old and new mustelid lines within BSMI were run in February and May 2006 to compare the detection rates.

As a new tool to detect and control mustelids within the Reserve, one staff member has a suitable dog certified and under training. The dog has been taken into the field during routine trap line servicing whenever possible. The handler and the dog also searched through areas where there are currently no traps within 500m. Mark VI trap with fold-out corflute cover with wire mesh entrances were placed in areas where the dog indicated presence of mustelids.

2.2.4 Results

- ***Mustelid Trapping***

As well as DoC staff, two to three local and many long-term volunteers assisted with fortnightly/monthly checking and maintenance of traps.

During this period a total of 47 stoats, 52 weasels and 4 ferrets were caught in Fenn and DOC 200 traps. This compares to a total of 88 stoats, 52 weasels and eleven ferrets caught in the previous period. With a 9 % increase in trap numbers, the three new trap lines accounted for 15 of the 103 mustelid caught (14%).

Stoats were caught throughout the reporting period with peak capture (n=6) occurring in February 2006 (see figure 2.2.1). Overall stoat capture was approx 50% less than that of previous season (see table 2.2.2). The new buffer line caught no stoats, while the new internal lines caught five stoats (see table 2.2.3). Four stoats were also caught in conibear cat traps on buffer lines (2 on Thomas Bush, 1 on Pohokura Road, 1 on

Woodstock). The sex of a majority of trapped stoats caught over summer was often unidentifiable since warm weather quickening the decomposition process (see table 2.2.4)

All four ferrets caught this season were on the forest edge (2 on perimeter, 2 on Section Four Ridge line). There was a 64% decrease in the number of ferrets caught this period compared with eleven last period.

A total number of weasels caught this period was consistent with that of previous year (See table 2.2.2). Internal captures increased by 52% with 10 weasels caught on the new internal lines. There was a 16% decrease in the number of weasels captured on the perimeter line and a 53% decrease on the buffer line (see table 2.2.3). Weasel captures were highest during December and March, and low from June to October (see figure 2.2.1).

Figure 2.2.1 Total numbers of ferret, stoat, and weasel captures for all traps from July 2005 to June 2006.

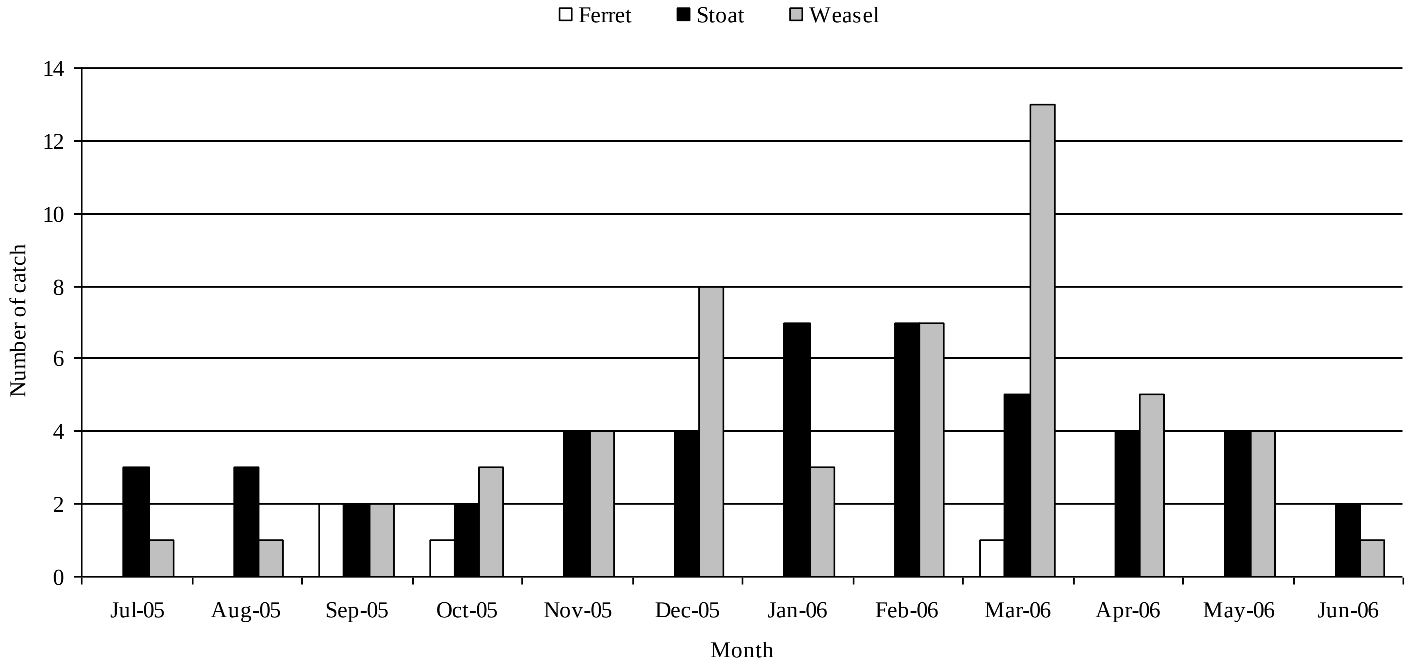

Table 2.2.2 Captures of Mustelid and trap type used between July 2005 and June 2006.

Location	No. traps		Stoats		Ferrets		Weasel	
	2004/05	2005/06	2004/05	2005/06	2004/05	2005/06	2004/05	2005/06
**Internal**								
MarkVI Fenn	197	**214**	24	**7**	0	**0**	17	**11**
DOC. 200	193	**262**	3	**7**	1	**0**	2	**18**
**Perimeter**								
Mark IV Fenn	373	**416**	24	**11**	4	**2**	12	**10**
DOC 200	20	**20**	0	**0**	0	**0**	1	**1**
**Buffer**								

Mark VI Fenn	278	**286**	25	**14**	5	**2**	13	**4**
DOC 200	20	**62**	5	**2**	0		0	**6**
Mark VI Fenn on Road	76	**84**	7	**2**	1	**0**	8	**2**
**Total**	**1137**	**1346**	**88**	**43 + 4 in connibear traps**	**11**	**4**	**52**	**52**

Figure 2.2.3 Locations of mustelid capture in the 2005-06 season

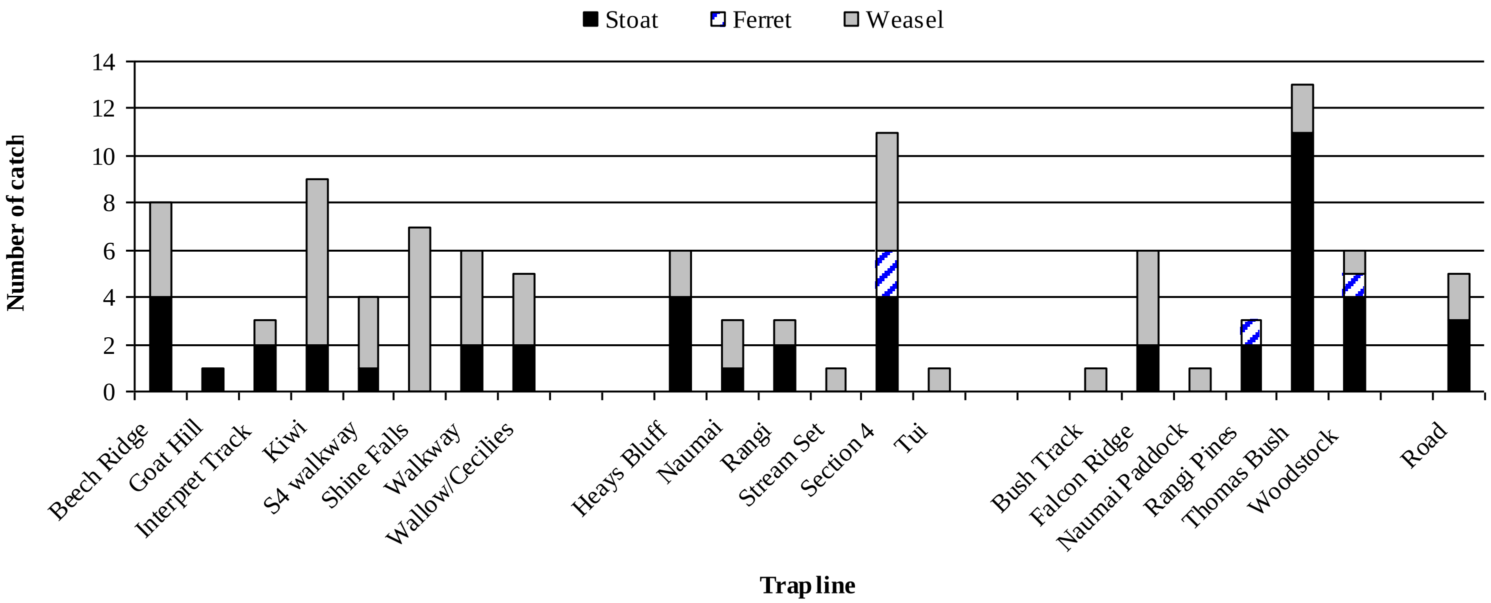

Internal **Perimeter** **Buffer**

Table 2.2.4 Sex and age of stoats caught at BSMI between July 2005 and June 2006. 'Unknown' contains animals that were too decomposed to sex or age.

Sex	Female (n= 13)			Male (n= 8)			Unknown
**Age**	**Adult**	**Sub- Ad Young**	**Unknown age**	**Adult**	**Sub-Ad Young**	**Unknown age**	
July	0	0	0	0	1	0	2
Aug	1	0	0	1	0	0	1
Sept	1	0	0	0	0	1	0
Oct	0	0	0	1	0	0	1
Nov	0	0	0	0	0	1	3
Dec	0	0	1	0	0	1	2
Jan	1	0	0	0	1	0	5
Feb	1	0	0	0	1	0	5
Mar	0	2	1	0	0	0	2
Apr	0	1	0	0	0	0	3
May	2	0	0	0	0	0	2
June	1	1	0	0	0	0	0
**Total**	**7**	**4**	**2**	**2**	**3**	**3**	**26**

A wide range of non-target species were also caught in mustelid traps (see table 2.2.5). There was a large decrease in both rat and hedgehog capture compared with that of previous season. On the other hand, by-catch of rabbits showed a slight increase from 116 last period to 134.

Table 2.2.5 By-catch from internal and buffer MK VI Fenns / DOC 200's and perimeter MK IV Fenns for the period July 2005 to June 2006.

	**2005/06**				**2004/05**
	**Numbers caught**				**Numbers caught**
**Species**	**Internal DOC 200's and Fenns**	**Perimeter Fenns**	**Buffer DOC 200's and Fenns**	**Total**	**Total**
Cat	3	6	15	**24**	22
Rabbit	1	30	103	**134**	116
Hare	0	0	0	**0**	0
Hedgehog	20	11	98	**129**	225
Possum	0	0	1	**1**	0
Rat	140	74	159	**373**	401
Mouse	11	4	0	**16**	5
Starling	0	2	2	**4**	2
Tui	2	0	2	**4**	8
Thrush	20	0	1	**1**	2

- ***Mustelid Monitoring***

Mustelids remained below 3% in BSMI this period, compared to consistently being detected in comparison sites with tracking rates of up to 17% (see table 2.2.6 and figure 2.2.7).

Table 2.2.6 Mustelid tracking tunnel results for the 2005-06 season

		**BSMI**			**Comparison sites**		
**Month**		% of tunnels tracked by mustelids	% of lines tracked by mustelids	Comments	% of tunnels tracked by mustelids	% of lines tracked by mustelids	Comments
**August 05**		0	0		17	67	
**November 05**		0	0		3	17	One stoat print in Cashes Bush
**February 06**	Old line	3	13	One stoat print in Section 4	0	0	
	New line	0	0				
**May 06**	Old line	0	0		17	33	Five stoat prints in Cashes Bush
	New line	0	0				

Figure 2.2.7 Percentage of tunnels tracked by mustelids in BSMI and Comparison sites from February 2001 to May 2006

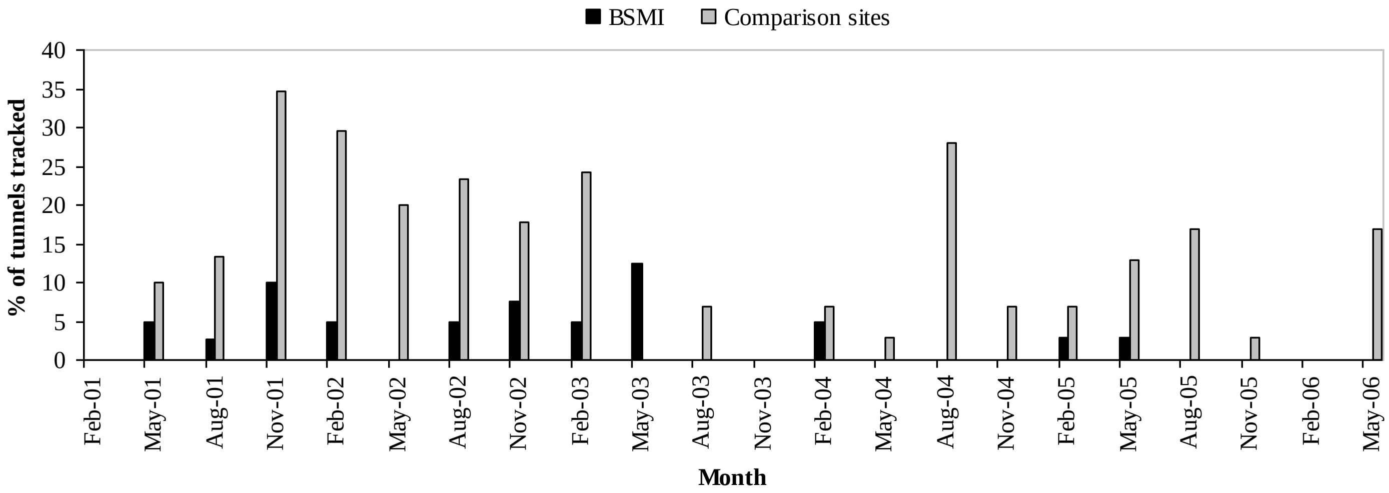

- ***Mustelid dog***

The mustelid dog was extensively used through Reserve during routine trapping and baitstation checks. The dog indicated presence of mustelids occasionally. No mustelids were caught by the dog or 'reactive' traps.

2.2.5 Discussion

Volunteer inputs remains significant for the mustelid program Maintaining the level of volunteer involvement is essential if the control regime is to continue to full potential.

This year there was an overall decrease of stoat and ferret captures despite the fact that more traps were in operation. There was an increase in the number of stoats and weasels caught inside the reserve which may be attributed to the placement of three new DOC 200 trap lines.

A large number of stoat and weasel captures concentrated in the Section 4/Kiwi area, which may indicate reinvasion and/or presence of resident population in the area. Any future changes in mustelid captures in this area may provide vital information for the control of mustelid. An increase in trap density within some areas may still need to be considered to ensure mustelid have minimal long term impact on native and re introduced species

The decrease in mustelid numbers caught on the buffer may suggest that a number of resident animals are being maintained to very low numbers in these areas. However, mustelid reinvasion may be greatly encouraged by the recent increase in rabbit population on farmland adjacent to BSMI since mustelid populations are known to fluctuate in response to variables such as food availability (ie. rabbits). There was also an increase in rabbit by-catch in mustelid traps this year. This raises a concern that traps set to target mustelid are being taking out of operation, and therefore shows strong support for double trap set systems as in most cases the second trap is still operational.

Low tracking indices within BSMI may suggest that current trapping regime is effectively maintaining mustelids to low levels within the Reserve. However, as large numbers of stoats and weasels are still being caught internally, it may require some time for mustelids to acclimatize with new tracking tunnels and to be detected. Also, tracking tunnels may need to be run more frequently over summer when they are most active in order to detect mustelid activities. In addition, the use of mustelid dog has proven to be helpful in identifying mustelid ‘hot spots’. The dog should continue to be utilized to audit efficiency of trapping and distribution of mustelid.

High number of rat and hedgehog by-catch also shows trapping efforts are successfully removing a range of pest animals. It has been noticed, however, Fenn traps are showing signs of wear and require upgrading. The Department has recently published the ‘mustelid control best practice’ on which DOC 200/250 traps are recommended for mustelids and a wide variety of other species such as rats and hedgehogs. Therefore, all Fenn traps should be replaced to new DOC traps when resource is available. This will help to achieve the long term objective of improving trapping efficiencies and techniques. Investigating mustelid catch and tracking tunnel results may determine whether control efforts can be reduced from fortnightly to monthly servicing of internal and buffer lines during mid winter, which would still meet the best practice.

2.2.6 Recommendations

- Continue to utilize ongoing volunteer involvement and training with trap line servicing and maintenance
- Utilize a mustelid indicator dog to audit the efficiency of the trapping program.
- Continue the salted rabbit meat trial until two years of data have been collected for analysis (December 2006)
.
- Plan for gradual replacement of the ageing MK4 fenn traps on the perimeter line with
DOC 250 single set traps starting 2006 / 07 year

2.3 Ungulate management

2.3.1 Summary

From July 2005 to June 2006, there were a total of 158 goats, 48 pigs and 16 deer shot in BSMI and the BSMI buffer. Numbers of ungulates killed in BSMI remains low, with 26 goats, 16 pigs and five deer removed. The majority of ungulate kills are from the BSMI buffer (132 goats, 32 pigs, and 11 deer). Observations of extensive pig rooting in certain areas of BSMI suggest that current pig control measures are inadequate to meet the short-term and long-term objectives outlined below.

2.3.2 Objectives

Short-term objectives

- To maintain goat numbers at zero within BSMI and at low levels in the surrounding buffer areas.
- To maintain deer and pigs at low levels within BSMI.
- To exclude domestic farm animals from BSMI.

Long-term objectives

- To enable the recovery of indigenous forest structure and ecosystem processes by controlling ungulates to low levels within BSMI.

2.3.3 Methods

- ***Goats***

Goat control consisted of opportunistic and targeted shooting. Project staff carried rifles while undertaking other duties in areas where goats were known or thought to be present, and to do so was practical. A targeted goat control operation was also carried out from 13th-17th February 2006 by area staff. A total of 32 hours were spent hunting BSMI and the BSMI buffer during this operation. Two goat control contractors undertook another ground based goat control operation from 15th-27th May 2006. Dogs were used to aid this operation.

An aerial goat control operation took place on 30th March 2006. Four shooters hunted for 2 hrs 50 mins in BSMI, BSMI buffer, Waitere, Waitere buffer, Cashes Bush, and Cashes buffer (refer figure 2.3.1). Rangiora Station was not shot due to difficulties in obtaining permission from the land owner. This report only includes animals shot in BSMI and the BSMI buffer.

- ***Pigs***

Pig control also consisted of opportunistic shooting, and by targeted hunting by project staff outside normal work hours. A small amount of project time was spent undertaking targeted pig control, however Paul MacDonald spent 110.5 hours pig hunting after hours, using 0-2 dogs in BSMI and the BSMI buffer. Some pig control was undertaken during the aerial shooting operation targeting goats on 30/3/06.

- ***Deer***

Control of deer in BSMI and the BSMI buffer was undertaken through opportunistic shooting by project staff while on other duties, and by some targeted hunting. Five hours were spent by project staff hunting deer, and some deer control was also carried out during the aerial goat shoot which took place on 30 March 2006.

2.3.4 Results

From July 2005 to June 2006, 158 goats, 48 pigs and 16 deer were shot in BSMI and the BSMI buffer. As in previous years, the numbers of ungulates killed in BSMI remains low, with 28 goats, 16 pigs and five deer removed. The majority of ungulate kills are from the BSMI buffer (132 goats, 11 deer, 32 pigs). Goat, pig, and deer observations and kills from BSMI and the BSMI buffer are shown in tables 2.3.1 and 2.3.2, respectively.

Table 2.3.1. Ungulates seen and shot in BSMI. Note that animals seen is not a reflection of abundance. NH-not hunted, NA-data not available.

	goats		pigs		deer	
	seen	shot	seen	shot	seen	shot
BSMI staff	31	14	76	15	16	4
Ground operation 13th-17/2/06	NH	NH	NH	NH	NH	NH
Contractors 15th-27/5/06	NA	12	NA	1	NA	1
Aerial shoot 30/3/06	0	0	0	0	0	0
Total	31	26	76	16	16	5

Table 2.3.2. Ungulates seen and shot in the BSMI buffer. Note that animals seen is not a reflection of abundance. NH-not hunted, NA-data not available.

	goats		pigs		deer	
	seen	shot	seen	shot	seen	shot
BSMI staff	159	75	42	21	22	2

Ground operation 13th-17/2/06	71	34	3	0	18	2
Contractors 15th-27/5/06	NA	18	NA	0	NA	0
Aerial shoot 30/3/06	7	5	14	11	8	7
Total	237	132	59	32	48	11

2.3.5 Discussion

As in previous years, the majority of ungulate kills in BSMI remains low in comparison to the number of kills in the buffer. Controlling ungulates in these buffer areas is crucial to minimise movements into the reserve. However control of ungulates on farmland surrounding BSMI is proving difficult due to the reluctance of adjacent land managers to allow ungulate control measures on their properties.
At present, pigs are causing much damage in the reserve, particularly in the lower Cecilies/Wallow area. Large areas of ground have been dug over, and parts of the walkway itself above Shines Falls have been rooted.

2.3.6 Recommendations

- Maintain ground control methods targeting goats.
- Encourage opportunistic shooting by project staff where practical.
- Continue to maintain accurate records of ungulate sightings and kills.
- Negotiate with land managers to allow greater control of ungulates on areas surrounding BSMI.

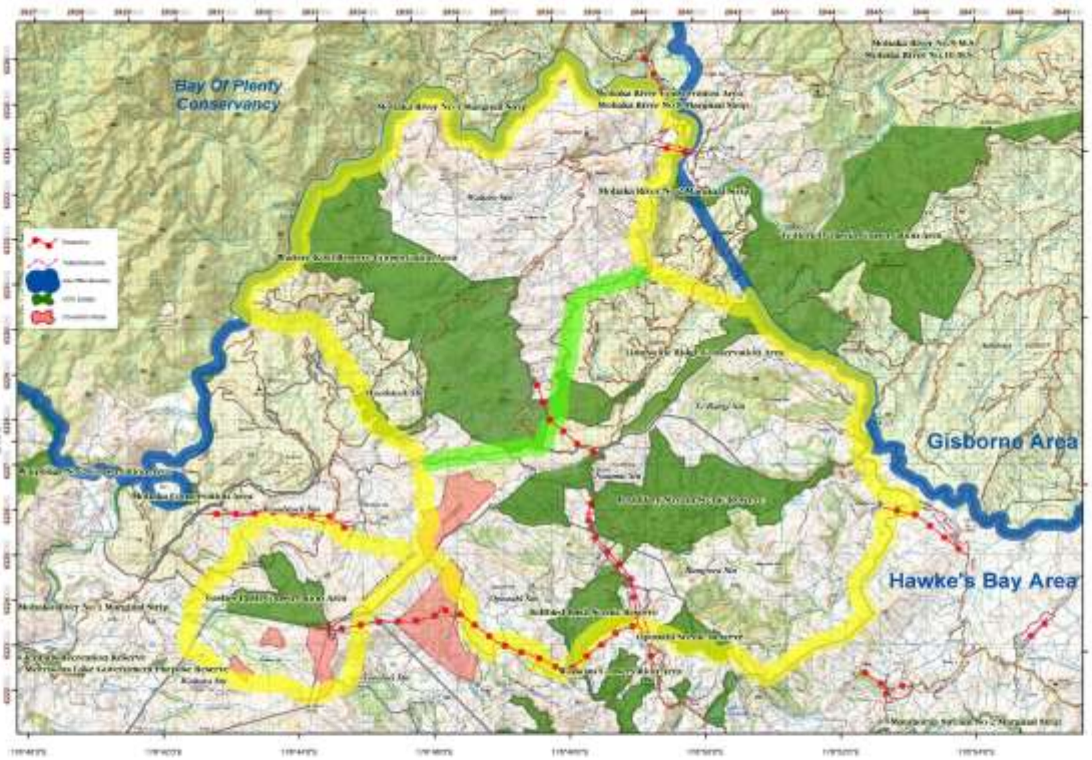

Figure 2.3.1. Goat control area for the Maungaharuru Range. Yellow Line depicts the control area. The green line separates the Waitere and BSMI buffer zones.

2.4 Cat management

2.4.1 Summary

Feral cat control at Boundary Stream Mainland Island (BSMI) is an important and established part of the Project's management efforts. During the 05/06 year there were a total of 53 traps targeting cats, consisting of 49 Conibear traps and four Greave's cage traps. Of these, 32 traps are on the perimeter, eight within the Reserve and 13 are on buffer lines.
The total number of cats captured during this period was 56, a reduction from the previous three years. Conibear traps were responsible for 25 captures, seven from Greave's cage traps, while 20 cats were caught as by-catch in Fenn traps, two in DOC 200 traps and two were shot. Sixty carcasses were sent to Jessica Hayes for research into feline immunodeficiency virus.

2.4.2 Objectives

Short-term objectives

- To control feral cats in and around BSMI to a level where none are sighted or trapped within the Reserve.
- No kiwi deaths within BSMI attributable to cat predation.

Long-term objectives

- To provide and maintain a safe environment for threatened species, in particular North Island brown kiwi and North Island kokako.

2.4.3 Methods

Between July 2005 and June 2006, a total of 49 Conibear traps and four Greave's cage traps were set. Eight Conibear traps were within the Reserve, and 28 set around the Reserve perimeter. Of the 13 Conibear traps established in buffer areas, eight were along the Thomas Bush and three on the Woodstock Regular Fenn trap buffer lines, and two traps were situated further along Pohokura Road close to Bellbird Bush Scenic Reserve.

All Conibear traps were set in chimney boxes and placed approximately 500 to 700 m apart, except along Pohokura Road where they are approximately 200 m apart.

Conibear traps on the perimeter were checked weekly, with the rabbit bait replaced and fishmeal lure applied on and around the trap box. The lure consisted of dried fishmeal and water (1:4), with glycerine added at a ratio of 1 part glycerine to 20 parts fishmeal liquid to reduce desiccation of the solution. The buffer line Conibear traps were checked fortnightly in

conjunction with mustelid trap inspections, while the internal traps were checked monthly during Fenn line inspections.

The four Greave’s cage traps remained set in areas of high public use such as along the road. These were checked every second day in the interests of animal welfare and legal requirements.

Cat carcasses which were in a usable state were sent to Jessica Hayes for research into feline immunodeficiency virus.

2.4.4 Results

A total of 56 cats were caught or shot during the 05/06 reporting year. This is a substantial drop in relation to the previous three years (figure 2.4.1). Thirty two cats were captured as part of the cat control operation, while by-catch from the mustelid traps (fenn and DOC 200 traps) contributed 22 captures to the cat control operation. A further two were shot.

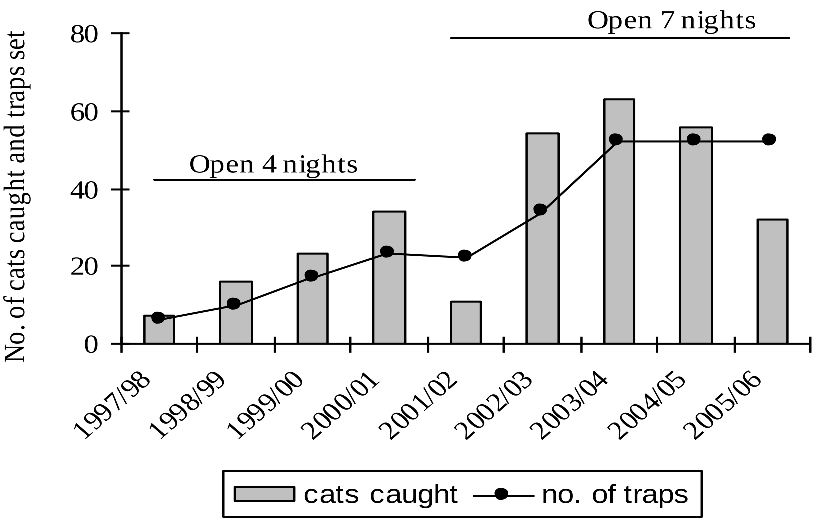

Figure 2.4.1 Number of cats caught as part of the cat control operation (Conibear and greaves traps). Note this does not include cats captured as by-catch from the mustelid control operation, or cats shot.

As in previous years, the majority of the cat captures took place either on the perimeter or the buffer traps. Only three cats were captured inside the reserve, 16 were caught on the buffer line, and 35 were caught on the perimeter. The two shot were also on or near the perimeter. Of the 38 cats that were aged, 22 were adults, 15 were sub-adults, and one was a kitten. 25 captured cats were fresh enough to be sexed, with 12 females and 13 males identified.

In addition to the captures, 22 cat observations were made. All but one of these took place on or near the perimeter. One was seen on the Woodstock buffer line.

Some by-catch took place in the greaves and conibear traps. Four stoats were caught in conibear traps, all on buffer lines. One rat, one sparrow and one Australasian harrier were also caught in conibear traps.
Sixty carcasses were sent to Jessica Hayes for research into feline immunodeficiency virus.

2.5.5 Discussion

The total lack of kiwi or kokako deaths attributed to cat predation during the 2005/06 year is an indication that the cat control efforts at Boundary Stream are meeting the long term objectives outlined.
The 56 cats destroyed during the 2005-06 period is a substantial drop from recent years, although one must be cautious in drawing too many conclusions from this result. While it is possible that the increase in trap numbers between 2001/2002 and 2003/04 has made an impact on the cat population there are other factors which could affect the cat capture rate, such as prey availability and climate. The low capture rate should not be interpreted as indicating a drop in the feral cat population.
The current level of cat control (number of traps) should be maintained for the near future, as it seems to be effective at minimising cat invasion into the reserve, as indicated by the very low capture (three) and sighting rate (none) inside Boundary Stream. The number of traps is also at a level where placing more would compromise the ability to be able to effectively service them with the current staff resources.

2.4.6 Recommendations

- Maintain current cat trapping regime.

3. Measuring ecosystem recovery: outcome monitoring

3.1 Bird monitoring

3.1.1 Summary

Five-minute bird counts were used to determine density, diversity, richness and evenness of bird communities in Boundary Stream Mainland Island (BSMI) and the Comparison sites.

Total bird density in BSMI and the Comparison sites was estimated at 15.95 birds per count site and 9.82 birds per count site, respectively. Excluding introduced species, the density was 15.08 in BSMI and 7.68 in the Comparison sites. This is a significant decrease in both areas from last year. Evenness and richness were both significantly higher in BSMI, while diversity was not significantly greater in BSMI. Bellbird, tui kereru and silvereye had the highest densities in both BSMI, while bellbird and silvereye dominated in the Comparison sites.

Kereru estimates, based on Distance Sampling suggested a 50% decrease in BSMI between autumn 2005 and autumn 2006. The comparison site count had a 68% decrease from autumn 2005. This results in a total population estimate of 1408 kereru for the Reserve, and 68 in Cashe's Bush.

3.1.2 Objectives

Short-term objectives

- To monitor bird communities in BSMI and Comparison sites using standard five-minute bird counts and ten-minute Distance Sampling counts for kereru.
- To investigate the effectiveness of management regimes in BSMI by monitoring change in bird diversity, richness and relative abundance.

Long-term objectives

- To compare long-term changes in bird diversity, richness and relative abundance in BSMI and Comparison sites.
- To restore bird populations to those reminiscent of a previous era as part of the BSMI ecosystem restoration project.

3.1.3 Methods

- ***Five-minute bird counts***

Monitoring bird communities in BSMI and Comparison sites uses standard five-minute bird counts as outlined in the BSMI 1996-98 project reports (refer also Dawson and Bull, 1975). Bird counts occurred from the 31 March to 12 April 2006. Data were analysed using Independent Samples T-Tests using SSPS software.

- ***Distance Sampling***

Distance Sampling was again used to estimate kereru numbers in BSMI and at one of the Comparison sites - Cashe's Bush. Methodology was the same as set out in the BSMI 2002/03 Annual Report, except that transect lines were visited until at least 80 observations were made, with each line being visited the same number of times to avoid biases (table 3.1.1). Counts were carried out in autumn, 21 to 31 March 2005. Data was analysed using DISTANCE 3.5 programme.

Table 3.1.1 Number of counts per transect for kereru distance sampling for the 2005/2006 period.

**Location**	**Transect**	**No. count sites per transect**	**No. counts/count site**	**Total number counts/transect**
**BSMI**				
Tumanako	Line A	10	2	20
Podocarps	Line B	10	2	20
Section 4	Line C	10	2	20
Boundary Stream	Line D	10	2	20
**Comparison site**				
Cashe's Bush	Line A	13	2	26
Cashe's Bush	Line B	7	2	14

- ***General bird observations***

Records were kept for other bird species seen during the course of work at BSMI. In particular falcon sightings were recorded and attempts made to determine whether falcon were breeding in the Reserve.

3.1.4 Results

- ***Five-minute bird counts***

In autumn 2006, 18 bird species were recorded in BSMI (12 native and six introduced) and 18 species in the Comparison sites (10 native and eight introduced - table 3.1.2). The total density of birds counted in BSMI and Comparison sites is significantly different ($P < 0.001$) at 15.95 birds per count site and 9.82 birds per count site respectively (figure 3.1.1). The density of birds at both sites has decreased compared to last year's counts. The total density of native species is also significantly higher in BSMI, at 15.08 per count site, than in the Comparison sites, with 7.68 per count site ($P < 0.001$ - figure 3.1.2).

Table 3.1.2 illustrates the mean density per count site for all species recorded. For native species, there were significantly more bellbird, tui, rifleman, robin and kereru ($P \leq 0.001$) in BSMI than the Comparison sites. There was a slight but insignificant difference in whitehead numbers between the sites. Falcons were recorded in the 5 minute counts from the comparison sites, but not in BSMI (although falcons are known to inhabit BSMI from incidental sightings).

The density of bellbird, tui and rifleman has decreased in BSMI but remained stable in the Comparison sites from last year, while tomtit remain at similar numbers (figure 3.1.3). Kereru numbers continue to fluctuate, and increased compared to last year, which is the inverse of the results from Distance sampling.

For introduced species there were significantly more chaffinch ($P < 0.05$) in the Comparison sites than BSMI.

There was a significant difference in evenness and richness ($P<0.001$) but not diversity ($P > 0.05$) in BSMI compared to the Comparison sites - table 3.1.2).

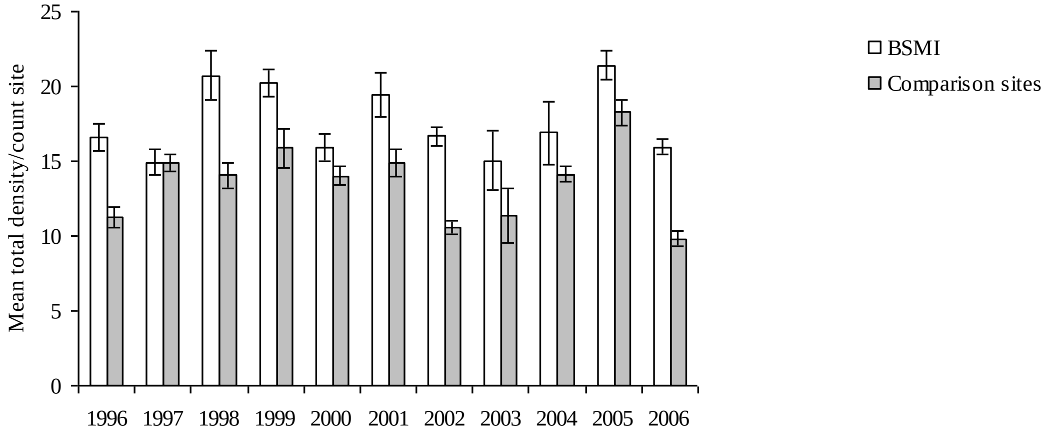

Figure 3.1.1 Annual trends in the mean bird density (both native and introduced species) per count site for BSMI and Comparison sites.

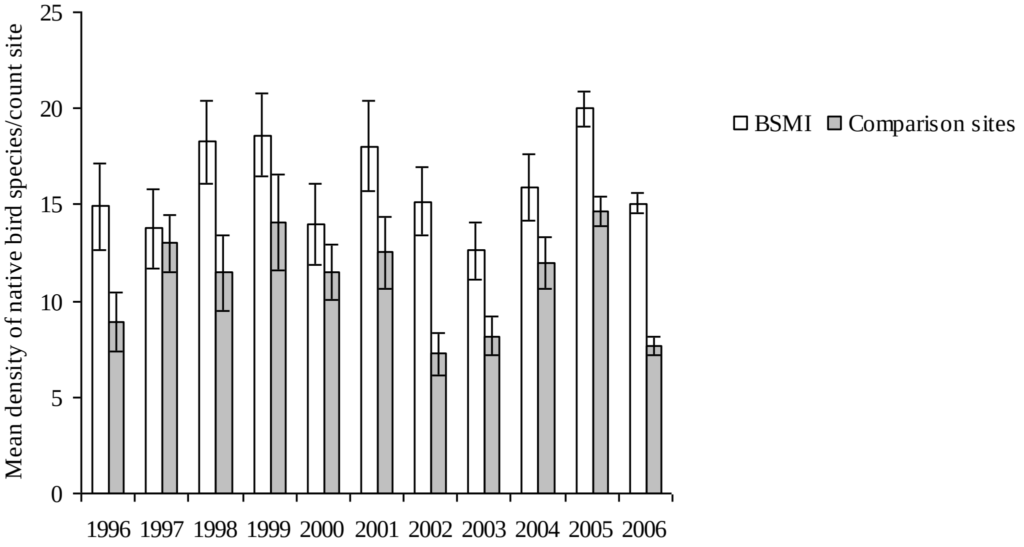

Figure 3.1.2 Annual trends in the mean bird density per count site for native species in BSMI and Comparison sites.

Table 3.1.2 Density of individual species, total density, richness, evenness and diversity for birds in BSMI and Comparison sites. Bold values indicate significance at the 0.05 level. A bold * indicates a significantly greater number in the Comparison sites.

	BSMI			Comparison sites			
**Species**	**Mean**	**SE**	**n**	**Mean**	**SE**	**n**	**P**
**Native**							
Australasian harrier *Circus approximans*	0.02	0.02	1	0	0	0	0.321
Bellbird *Anthornis melanura*	4.27	0.18	256	2.32	0.13	125	**<0.001**
Fantail *Rhipidura fuliginosa*	0.48	0.08	29	0.46	0.10	25	0.771
Grey warbler *Gerygone igata*	0.45	0.07	27	0.61	0.09	33	0.199
Kereru *Hemiphaga novaeseelandiae*	2.17	0.25	130	0.67	0.12	36	**<0.001**
New Zealand falcon *Falco novaeseelandiae*	0	0	0	0.02	0.02	1	0.322
North Island kokako	0.02	0.02	1	0	0	0	0.321
Rifleman *Achanthisitta chloris*	0.98	0.13	59	0.22	0.07	12	**<0.001**
Robin *Petroica australis longipes*	0.32	0.08	19	0	0	0	**<0.001**
Silvereye *Zosterops lateralis*	1.83	0.36	110	1.65	0.27	89	0.591
Tomtit *Petroica macrocephala*	0.58	0.06	35	0.50	0.07	27	0.295
Tui *Prosthemadera novaeseelandiae*	2.95	0.25	177	0.98	0.13	53	**<0.001**
Whitehead *Mohoua albicilla*	1.02	0.21	61	0.54	0.22	29	0.096
**Introduced**							
Blackbird *Turdus merula*	0.28	0.06	17	0.44	0.08	24	0.154
Chaffinch *Fringilla coelebs*	0.22	0.07	13	0.82	0.13	44	**<0.001***
Goldfinch *Carduelis carduelis*	0	0	0	0.06	0.06	3	0.303
Magpie *Gymnorhina tibicen*	0.33	0.08	18	0.13	0.06	8	0.055
Redpoll *Carduelis flammea*	0.10	0.10	6	0.11	0.05	6	0.949
Starling *Sturnus vulgaris*	0.12	0.05	7	0.11	0.06	6	0.897
Thrush *Turdus philomelos*	0.02	0.02	1	0.02	0.02	1	0.961
Yellowhammer *Emberiza citrinella*	0	0	0	0.06	0.06	3	0.070
Total density	15.95	0.53	957			530	**<0.001**
Total density (native species)	15.08	0.50	905			430	**<0.001**
Richness							**0.001**
Evenness							**<0.001**
Diversity							0.053

a) Bellbird

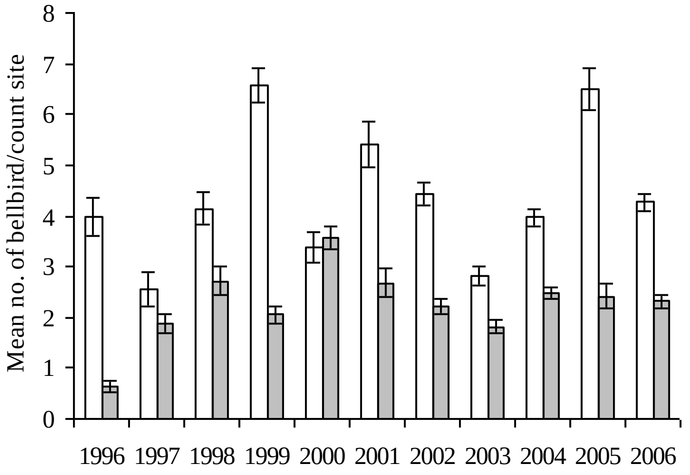

b) Tomtit

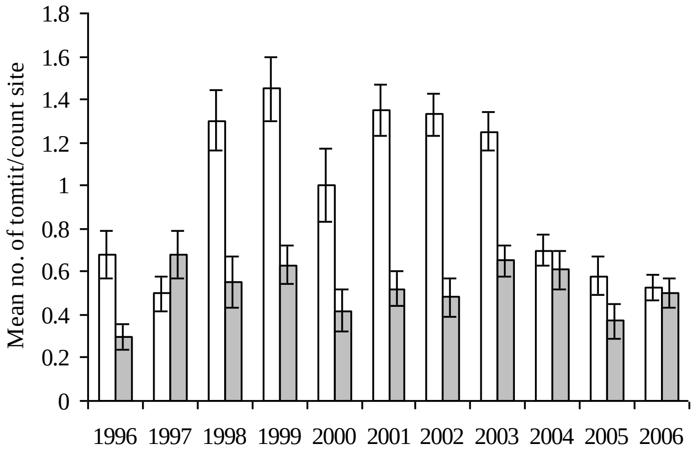

c) Kereru

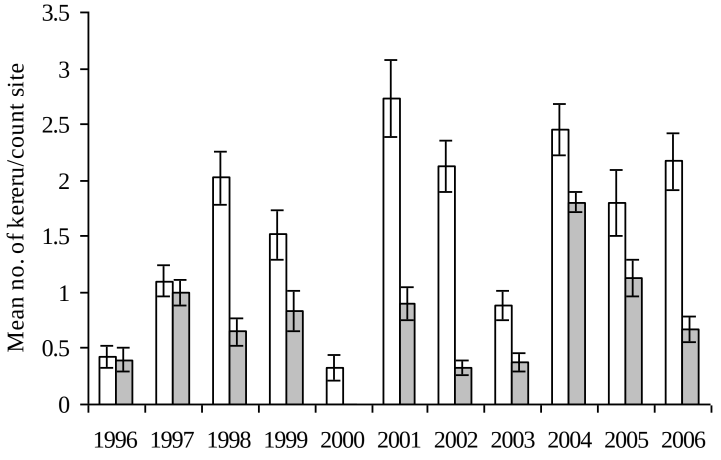

d) Tui

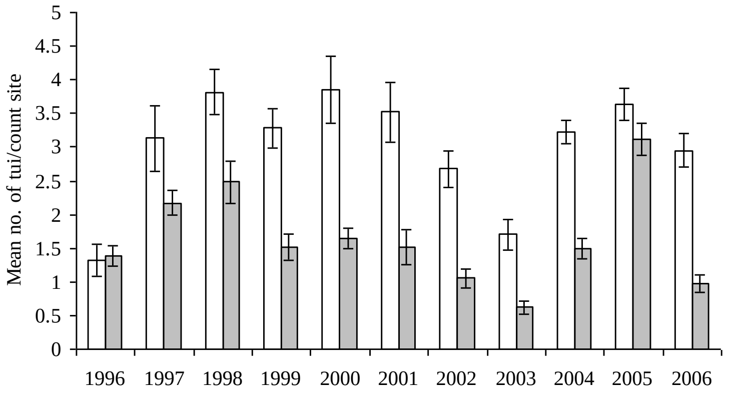

e) Rifleman

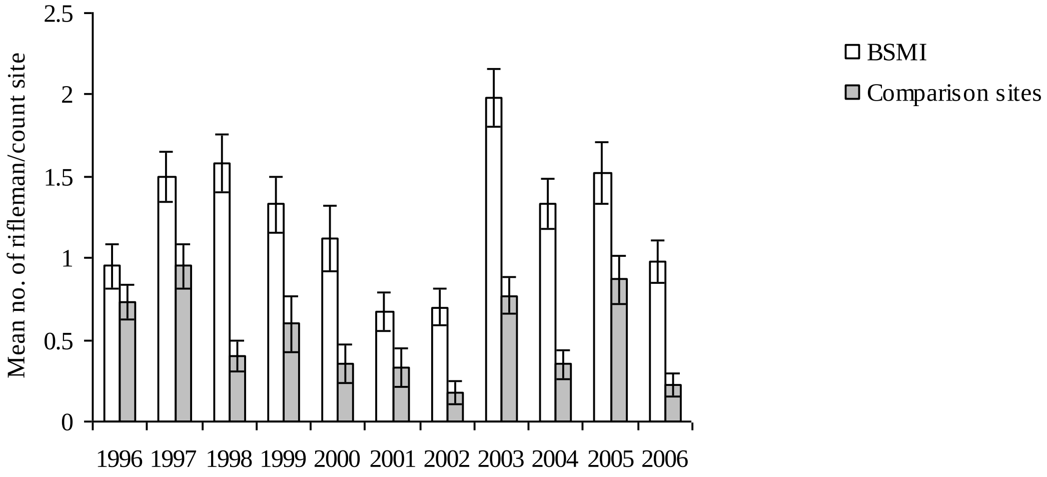

Figure 3.1.3 Mean density of (a) bellbird, (b) tomtit, (c) kereru, (d) tui and (e) rifleman detected per five-minute count in BSMI and Comparison sites.

Bellbird and tui were the most widespread species detected at 93.3% and 78.3% of count sites respectively, however, both species have decreased from last year. Kereru (up 5%), tomtits (up 23.1%) and fantail (up 57.5%), were detected at more of the count sites this year than in previous years. Whitehead (down 21.8%), silvereyes (down 38.3%), grey warbler (down 48.9%), and rifleman (down 27.3%) were detected at fewer count sites (figure 3.1.5).

(a)

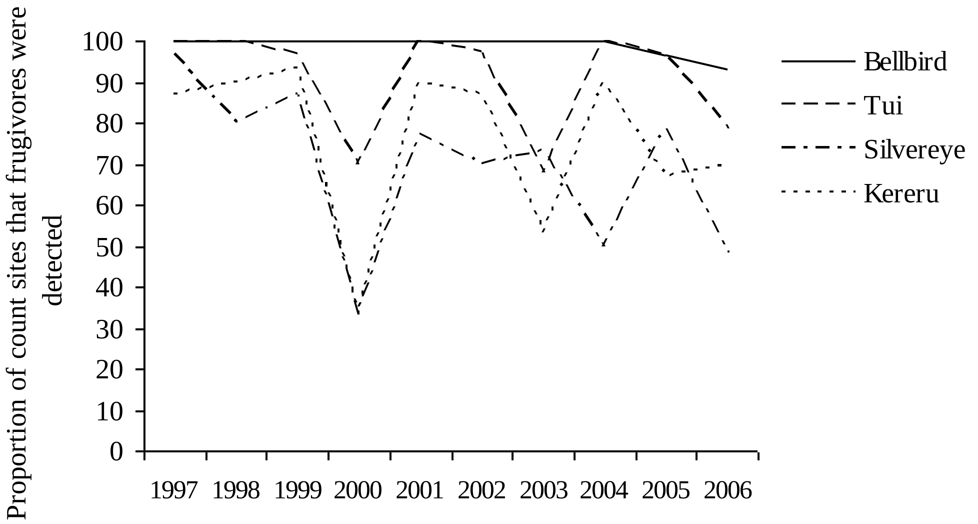

(b)

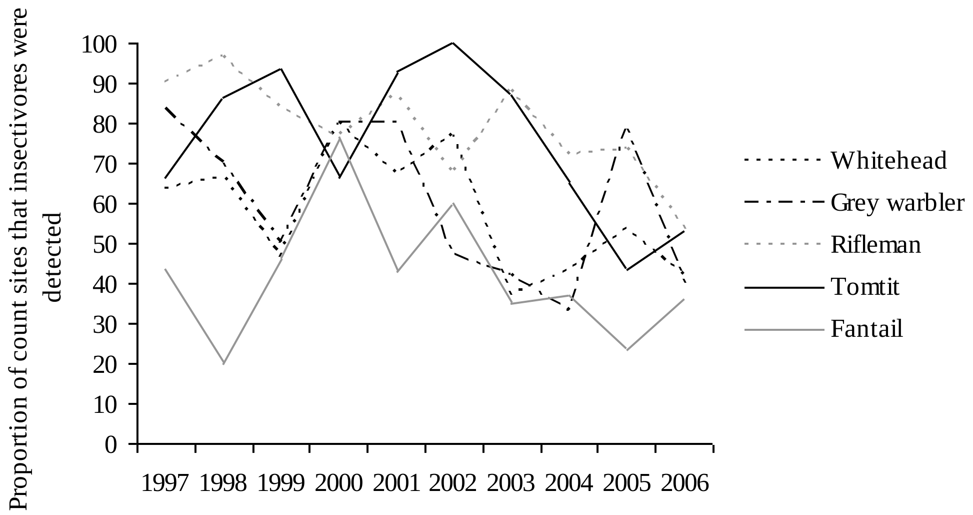

Figure 3.1.5 Proportion of count sites at which (a) frugivorous species and (b) insectivorous were detected in BSMI.

- ***Distance Sampling***

DISTANCE gave an estimate of 1.76 kereru/ha in BSMI for autumn 2006; and an estimate of 0.54 for the comparison site. This is a 50% decrease from autumn 2005 for BSMI and 68% decrease for the comparison site. Confidence intervals decreased in BSMI (figure 3.1.6), possibly because each line was visited twice, giving a sample size of 136 distance estimates. Using these estimates, a population size of 1408 (890-2238; 95% confidence) and 69 (35-131; 95% confidence) is suggested for BSMI and the comparison site, respectively.

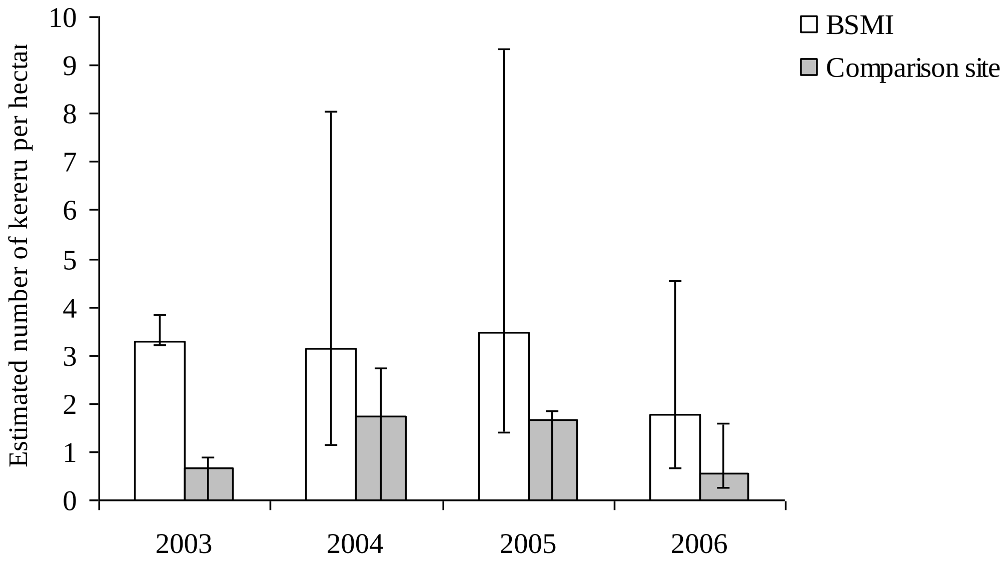

Figure 3.1.6 Kereru population estimates for BSMI and the comparison site (Cashe's Bush) for autumn 2003 to autumn 2006. Error bars indicate 95% confidence intervals.

- ***General bird observations***

Falcon continued to be present in the reserve during this period. They were seen and heard around much of the reserve. A pair was seen regularly through mid to late November near the Pohokura Road car park and appeared to be showing breeding behaviour. However, it appears that breeding was once again unsuccessful as the pair did not defend the area in December and fledglings were not observed. Falcon were seen regularly in Section Four and may be different birds to the Tumanako Loop Track pair. A falcon was also heard in Cashe's Bush.

A kaka was seen or heard on five different occasions in the Reserve or nearby in Bellbird Bush. The sighting in Bellbird Bush occurred in July, while the other sightings were from September through to October, possibly the same bird.

3.1.5 Discussion

Bird density at BSMI decreased significantly this year, and is possibly attributed to the increased rat numbers (see section 2.1). Mean bird density and mean native bird density continue to be significantly higher in BSMI than in the comparison sites, suggesting that if rats were the cause of the decline, management at BSMI is still having some effect in minimising the impact of predation.

Species richness, defined as the number of species within a community, and species evenness, which relates to the distribution of species over the individual count sites, are both significantly different between BSMI and the comparison sites. In BSMI, the bird community is dominated by bellbird, with high numbers of tui, kereru and silvereye, in that order. In the comparison sites, the dominant species are bellbird, and silvereye.
Species diversity does not significantly differ between BSMI and the Comparison sites, with both areas recording the same number of species, on average, per count site.

As usual, bellbird and tui were the most common and widespread species during the year, with both being detected at most count sites, however, with a slight decrease in detectability than last year. Fantail, kereru and tomtit increased, while whitehead, rifleman, grey warbler and silvereyes were detected at a lower percentage of the count sites than last year.

Kereru estimates for autumn have decreased. This is possibly due to the influx of rats, detected in February 2006. Distance Sampling continues to be an effective method of estimating kereru abundance and trends, and combined with other monitoring methods gives a good indication of the effectiveness of management techniques.

3.1.6 Recommendations

- Continue annual bird counts and annual kereru surveys in BSMI and the Comparison sites.

3.2 Vegetation monitoring

3.2.1 Summary

Vegetation monitoring occurs every five years. The next monitoring period is scheduled for 2009. Analysis of the 2004-05 monitoring is currently been conducted by Landcare Research.

3.2.2 Objectives

Short-term objectives

- To assess the change in vegetation composition, diversity and density in response to the intensive control of browsers using repeat photographs (photo points).

Long-term objectives

- To continue to use vegetation monitoring to assess changes in forest diversity and levels of control required

3.2.3 Methods

Annual checks on exclosure fences were conducted.

3.2.4 Results

No maintenance was needed on exclosure fences.

3.2.5 Recommendations

- Continue annual checks on exclosure fences and maintain if necessary

3.4 Lizard monitoring

3.4.1 Summary

This year's monitoring results showed that the number of skinks and geckos remains low at Boundary Stream Mainland Island (BSMI). Monitoring of artificial cover objects (ACO) lines, grids and gecko crevices appears successful in detecting the presence of lizards, though they are not yet sensitive enough to identify any changes in population size, probably due to low density of lizards within BSMI.

3.4.2 Objectives

Short term objectives

- To survey lizard populations in BSMI to assess lizard diversity and abundance.
- To monitor changes in gecko and skink diversity and abundance over time.

Long term objectives

- To determine the effectiveness of predator control on the recovery of lizard populations at BSMI.
- To assess the potential of lizard monitoring as an indicator of predator control effectiveness and/or ecosystem restoration.
- To assess the effectiveness of lizard houses and lizard shelters as a technique for monitoring gecko and skink populations respectively.

3.4.3 Methods

- ### Lizard house monitoring

All gecko houses in BSMI and Comparison sites were checked for gecko occupancy once in July-August 2005. Full details of methodology are in the BSMI 1998-2000 Project Report.

- ### Gecko crevice monitoring

A series of crevices along the Naumai Bluffs were checked in July and November 2005, and January 2006, and the number and species of gecko were noted (See the BSMI 2002-03 Annual Report for set up).

- ### Skink Artificial Cover Objects (ACO)

Distribution lines

Unfortunately, due to time constraints the ACO lines could not be checked in a systematic fashion, although some lines were checked on an opportunistic basis while staff were in the area. The Kamahi and Tumanako ACO lines were checked in November 2005 and January 2006, and the Heays Bluff line was checked in December 2005. Each corrugated iron cover or wooden plank was lifted and attempts were made to capture and the species identified.

Density grids

Five ACO grids (two along the Naumai Bluffs, two along the Kamahi loop track, and one along the Tumanako loop track) were checked in July using the same methodology as the distribution lines. For setup and configuration see the BSMI 2002-2003 Annual Report.

- ***Anecdotal observations***

All incidental sightings of geckos and skinks within and around BSMI were recorded in a species observation book.

3.4.4 Results

- ***Lizard House Monitoring***

Two forest geckos were found in the Thomas Bush lizard houses (numbers 10 and 28), and one forest gecko was found in Cecilies (number 6). No other houses were found to be occupied, although most were occupied by tree weta.

- ***Gecko crevice monitoring***

One common gecko was found in one of the crevices during the January survey. The days when Naumai Bluffs area and crevices were surveyed were fine and generally warm.

- ***Skink ACOs***

Corrugated iron and wooden ACOs

Two common skinks and four common geckos were found under the corrugated iron ACOs (One common skink on Heays Bluff, and one on the Naumai Bluffs; one common gecko was found on each of the Tumanako and Kamahi track ACO lines, and two at Goat Hill). No lizards were found under the wooden ACOs, and one unidentified skink was found under one of the ACOs on the Naumai Bluffs.

ACO grids

There were only three sightings of common skinks during ACO grid surveys this year, one on the Tumanako line, and two on the Naumai bluffs.

3.4.5 Discussion

Unfortunately the lizard monitoring setup has deteriorated. Several ACO's are missing, and many are inadequately marked. This is largely due to lizard monitoring being a low priority during busy times, and loss of information occurring during staff changeovers. While it would be a positive move to see the monitoring set up reviewed and maintenance requirements met, current resources do not allow this to happen.

Lizard finds in the gecko houses, ACO's and ACO grids continue to be low. This is possibly a reflection of low lizard abundance in BSMI, or of the lack of proven lizard monitoring techniques.

3.4.6 Recommendations

- Gecko houses should be checked once in the 2006/07 season, and gecko crevices should be checked in fine and calm days
- ACO grids and lines should be checked twice per year
- Missing shelters, tags and lines should be replaced and the all monitoring sites and descriptions should be documented properly should resources allow

3.5 Invertebrate monitoring

3.5.1 Summary

Pitfall traps were not monitored this year. A review of invertebrate methodology was undertaken. Tree weta abundance, monitored using weta houses, decreased slightly compared to last year's results in both BSMI and the Comparison sites.

3.5.2 Objectives

Short-term objectives

- To monitor and compare annual trends in weta numbers in BSMI and comparison sites.
- To review methodology and assess progress in meeting long term objectives.

Long-term objectives

- To assess the relevance of pitfall-trapped invertebrate numbers and species composition as an indicator of the success of indigenous ecosystem restoration.
- To identify changes in the relative abundance, body size, and order/family diversity of the active ground-based invertebrates in response to different conservation management regimes.
- To identify changes in the relative abundance of tree weta in response to different conservation management regimes.

3.5.3 Methods

All field methods for weta monitoring remain the same as for the BSMI 1998-2000 Project Report. The invertebrate pitfall traps were not opened this year, due to staff shortages. In July 2005, a review of the methodology was initiated, highlighting a number of inadequacies in both the design and analysis of the data. The results of this review are outlined.

3.5.4 Results

- **Review**

1. **Problem**: Individual pitfalls (2m apart) and groups of pitfall (10-15m apart) within lines are not independent and too close together

   Ideas considered:
   - Analyse by group or line - more accurate interpretation but increases standard error.
   - Reducing the number of pitfalls to one pitfall per group - not an option as there is too much variation between individual pitfalls.
   - Changing trap layout - not an option as we would lose the ability to compare with previous years.

   Possible solution:
   - Analyse by group, however this would reduce standard errors.

2. **Problem**: While field work only takes a small amount of time, sorting is incredibly time consuming.

   Ideas considered:

- Reducing the number of pitfalls - not an option as there is too much variation between individual pitfalls.
- Reducing monitoring to e.g. alternate years - high variation between years

Possible solutions:
- Sort only key species, principally carabids, large-bodied spiders, cave/ground weta; other suggestions include woodlice, centipedes, millipedes, earthworms, snails and slugs, and the larvae of certain flies.

3. **Problem**: No apparent trend in overall invertebrate numbers, or key groups such as beetles or arachnids

   Ideas considered:
   - Over generalisation – separate out the various components, at Order level at the very least. From the data may be able to see what groups are doing better from year to year but if they fluctuate then it may mean there are different drivers at work, not just management of pests.

   Possible solutions:

   - Analyse using bio-indicators, such as carabids, large-bodied spiders and cave weta, as above, however, this does mean a change in objectives

4. **Problem**: The pitfalls are left out for a month over summer, and the contents may have decomposed.

   Ideas considered:
   - Additional monitoring to determine whether two samples over two time periods instead of one four week sampling period gives better information (adding pitfalls and comparing the two regimes); however, this would increase the time required considerably.

   Possible solutions:
   - Sample in one month blocks, but once removed from field, preserve the sample in ethanol quickly to reduce risk of further deterioration. Deterioration hasn't been deemed a problem yet, but will need to be monitored in the future.

5. **Problem:** There is likely to be a great deal of variation from one year to the next due to prevailing weather conditions and sampling is always from mid December to mid January. The traps would catch different groups of emergent/active invertebrates, due to differing seasonal changes, the previous breeding conditions, the weather at the particular time, including temperature, etc - as not all the conditions are the same from year-to-year. This will affect the overall numbers, and certainly the relative numbers of the invertebrate groups.

   Ideas considered:

- Analysis data with regard to weather readings to determine which years are relatively normal and thus comparable. This may involve day-degree temperature analysis, with rainfall as another variable to account for, keeping in mind that with some inverts this may take a year or two to show.
- Leave samples out for 2-3 months (changing solution 1-2 times per month) to encompass a range of climatic changes. However, this would increase the time and resources required.

Possible solutions:
• Test to see if by excluding 'non-normal' years there are trends in the bio-indicator species. If so, this would suggest that weather is the key influencing factor. Include in any analysis a covariate of the El Nino Southern Oscillation values (December; free off the web) and see if it does significantly affect invertebrate numbers.
• If this doesn't work then leave samples out for three months – December to February, but only sort certain species rather than the whole sample to save time.

6. **Problem**: The non treatment sites have been compromised by management of ungulates, rodents and possums. Intensity of these operations vary from year to year. Controlling ungulates improves the understory and thus leaf litter. There is a lack of suitable non-treatment areas in the vicinity of BSMI which resulted in the treatment and non-treatment habitat types not being comparable, differing in aspect, altitude and vegetation.

Ideas considered:
• Concentrate efforts in monitoring BSMI. The ability to compare between treatment/non-treatment sites has been lost due to continuing changes to the management levels. This would still show changes in biodiversity in BSMI over time (ie using the baseline data as a 'control'), but would need a change in objectives.

Possible solutions:
• Recommendations suggest some sort of control is still required, even if it is compromised, ie low predator and ungulate control as opposed to no control – and therefore monitoring should continue in 'non-treatment' sites.
• Monitoring tree weta through weta houses appears to show trends between BSMI and comparative sites, although response in tree weta is due to aboreal predators i.e. ship rats. However, caution is needed as weta habitation may reflect the environment for example the abundance of suitable holes. This could be used to look at trends over time rather than comparing between sites.

7. **Problem**: The current spacing of bait stations (150 m grid) is presumed to be effective at targeting rats, but not mice. The impact of increased mice numbers in the treatment area on the invertebrate community is unknown. The impact of increased mice numbers on the smaller invertebrate size classes may be heavy. Some rodenticides are not very effective against mice. Part of the management of rodents requires frequent changes in toxins used, which may influence mice densities, as well as seasonal and yearly food source variations. Thus without good records of mice densities it is impossible to make any correlation with the invertebrate figures. Tracking tunnels are

not set up near the pitfall lines, thus it is impossible to compare mice activity with invertebrate abundance, as both are highly variable between habitats.

Ideas considered:

- Some beetles (carabids) and even millipedes have defence secretions which are certainly effective against birds and could be effective against mice. These could possibly be used as indirect bio indicators. However, with the current data, there are greater numbers of large carabids in the comparison sites, and confusing trends for the millipedes (all millipede species are grouped together).

Possible solutions:

- Analyse data in terms of mice numbers

- **Weta Houses**

Tree weta abundance in weta houses decreased, albeit insignificantly, in both BSMI and the Comparison sites. Within BSMI, there was an average of 0.44 tree weta per weta house, compared to 0.22 in the Comparison sites. With the exception of 1999, tree weta numbers in the Comparison sites remains approximately half of weta in BSMI.

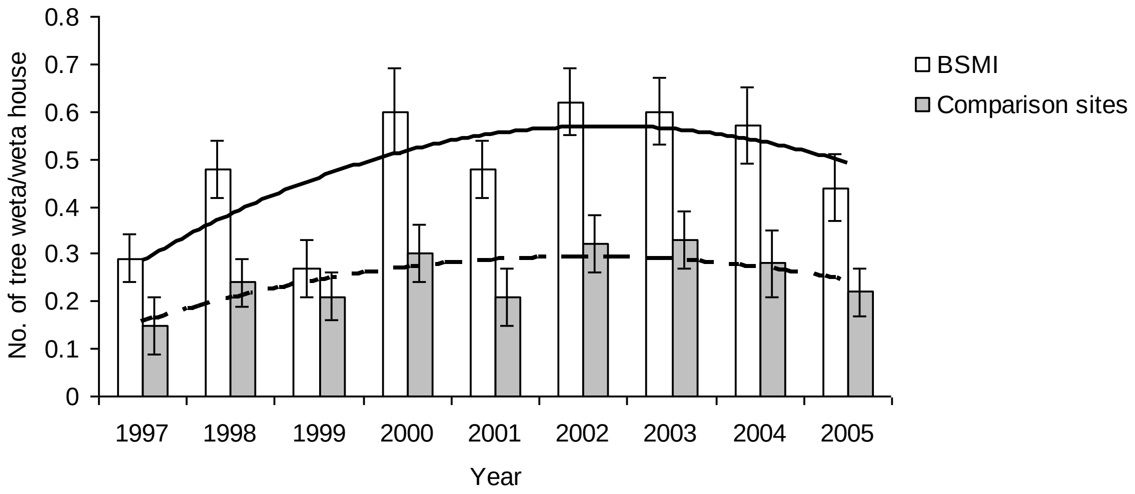

Figure 3.5.1 The average number of weta per house (mid-December) at BSMI and Comparison sites, 1997 to 2005. The solid line shows the polynomial trendline for BSMI, and the broken line shows the polynomial trendline for the Comparison sites.

Weta in all lines decreased, except for Lines 8 and 9 (both Cashes Bush), where weta abundance increased or remained stable respectively (Figure 3.5.2). Line 9 however, had only one tree weta.

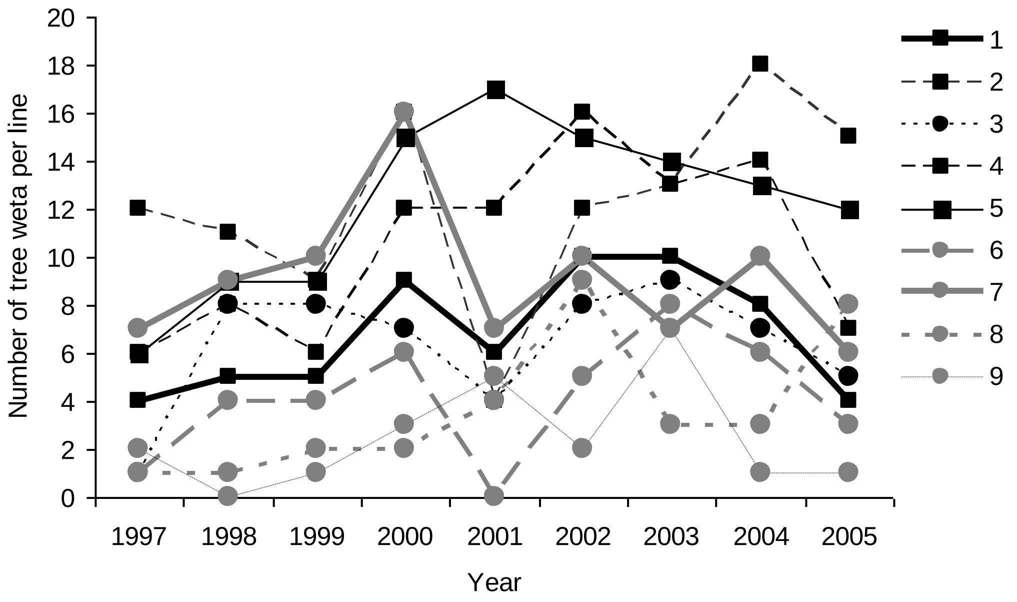

Figure 3.5.2 The number of weta per line. Lines 1-5 BSMI; 6 & 7 Thomas Bush (Comparison site); 8 & 9 Cashes Bush (Comparison site). Line1: cloud-capped, mixed low forest and shrub-treeland; Line 2: kamahi/rewarewa/kanuka, kamahi dominant; Line 3: tall tawa with large podocarps; Line 4: low secondary forest of wineberry and rangiora; Line 5: tall dry kanuka and grassland; Line 6: kamahi/rewarewa/kanuka, kamahi dominant; Line 7: tall dry kanuka; Line 8: totara, red beech with horopito and fuchsia understory; Line 9: high altitude broadleaved forest with beeches and podocarps. Vegetation descriptions follow Geoff Walls vegetation mapping (BSMI 1996-1998 Project Report).

3.5.5 Discussion

Pitfall trapping

In the past, trends in many of the invertebrate guilds appear to be non-existent or difficult to interpret. This is likely to be due to the confounding factors discussed in the BSMI 2003/04 Annual Report. These include changes in weather patterns, particularly temperature and humidity, the inter-relationship between various predator and prey species, impact of mice and their inverse relationship with rats, and possibly the reduction of possums and browsers over Maungaharuru Range which would have some impact on invertebrate numbers within the Comparison sites. The sampling period may be too short, too infrequent or at the wrong time of year. Due to these confounding factors, the sampling regime may be unable to determine changes in various invertebrate taxa with management.

Tree weta monitoring

The results from the tree weta monitoring suggest a small decline both within the Reserve and Comparison sites, in contrast to the past five years where abundance has remained stable, with the exception of 2001

3.5.6 Recommendations

- Flag the need for research into small mammal impacts on invertebrate populations
- Analyse the tree weta data, investigating potential size changes in weta over time and between BSMI and the Comparison sites

Acknowledgments

Ian Westbrooke, Alison Evans, Bruce McKinley, Chris Green, Brendon Christensen, Ian Stringer

4. Species reintroductions

4.1 Monitoring of North Island robin (*Petroica australis longipes*)

4.1.1 Summary

The population of North Island robin in Boundary Stream Mainland Island (BSMI) has increased by an estimated 62% since September 2003, to an estimated 136 birds. Dispersal is continuing with a large number of robins recorded in the outer blocks of the Reserve.

4.1.2 Objectives

Short-term objectives

- To monitor trends in robin numbers through biannual censuses, prior to and after the breeding season.

Long-term objectives

- To maintain a self-sustaining population of North Island robin within BSMI.

4.1.3 Methods

The robin survey was reduced to annually this year, undertaken prior to the breeding season to avoid the overabundance of juveniles causing an over-estimation in the population estimate. The survey occurred from 1-29 September 2005. The methods used were the same as reported in the BSMI 2000/01 Annual Report. In this report, only the September surveys will be discussed, as these surveys are considered a more accurate representation of the population.

- ***Data analysis***

Data were continued to be analysed using 'Mark-Recapture', as described in the BSMI 2001/2002 Annual Report. MARK determined that the preferred model this season was {ϕ, P_t}, where survival was constant and recapture was time dependent, with no distinction between adults and juveniles.

- ***Banding***

No robins were banded prior to the survey.

4.1.4 Results

The model suggests that the total BSMI population during September 2005 was 136 (CI: 83:221) (figure 4.1.1). This is an increase of around 62% on the 84 (CI: 52, 134) robins estimated in September 2003. The confidence intervals continue to be large, possibly due to the low number of birds banded. Two of the originally translocated birds (YM-Y and RM-G) were seen in the survey. The estimate of 136 is lower than the 172 robins estimated in autumn 2005 (BSMI 2004/05 Annual Report). This high autumn estimate is probably due to the abundance of young juveniles in the population, and many of these birds would have died during winter (D. Armstrong, pers. comm.).

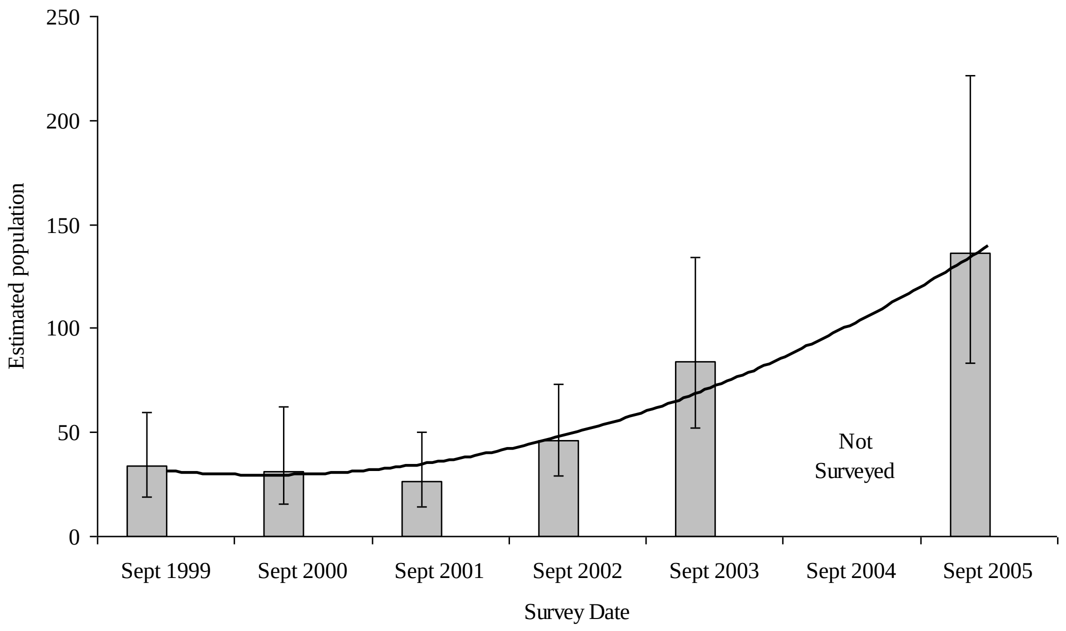

Figure 4.1.1 Estimated population sizes (± 95% CI) from September surveys using resighting probabilities given from model {ϕ, P_t}. Solid line depicts the polynomial trend line.

The majority of sightings of robins continue to be in 'Te Tatimana' with 28% of the robins seen in this block. 'Podocarps', 'Wallow', 'Tumanako' and 'Rob's Hole' blocks also had high numbers (see figure 2.1 in the BSMI 1996-1998 Project Report for a map of blocks). Numbers continue to increase in the outer areas of the Reserve, and while not recorded in the survey, robins have been heard in Bellbird Bush and for the first time, one was sighted in Thomas Bush.

4.1.5 Discussion

The robin population is steadily increasing each year. Robins are continuing to be recorded in higher numbers in BSMI blocks other than ‘Podocarps’, ‘Te Tatimana’ or ‘Tumanako’, which have until recently been their stronghold, confirming that dispersal outside of these areas is progressing.
The population while increasing is still relatively small, and is potentially prone to events such as increased rat or mustelid numbers. Continuing to monitor the robin population at BSMI will provide an indication of the health of all bird communities. The continued management of pests at BSMI will allow the population to become a source for other sites. Keeping an accurate record of the population size will assist the decision making when deciding to translocate, without detrimentally impacting on the BSMI.

4.1.6 Recommendations

- Survey the population once a year, in September

- Continue to band 10% of the census results to enable more reliable monitoring and improve future census estimates. Band robins between November and April to avoid inducing winter mortality.

- Continue attracting robins and feeding them with mealworms throughout the year

- Monitor neighbouring Reserves for the presence of robins at the same time as census

4.2 Reintroduction and monitoring of North Island brown kiwi (NIBK) (*Apteryx australis mantelli*)

4.2.1 Summary

The first wide scale kiwi call count survey (totalling 40 hours of listening) was conducted within BSMI this year.. This has provided baseline data of the population of BSMI kiwi. The presence of four missing birds was detected during the survey.

Nine monitored nesting attempts produced 10 chicks. The successful breeding season, however, was overshadowed by a number of transmitter failures and high mortality of both chicks and sub-adults. As at June 2006, the total number of kiwi monitored at BSMI was 19 (seven males and 12 females). It is crucial that all birds are fitted with the best quality transmitters in the future.

4.2.2 Objectives

Short-term objectives

- To maintain 20 adult kiwi within BSMI of even sex ratio and monitor them in accordance with the current Best Practice.

- To encourage the participation of the general public in the reintroduction of NIBK to BSMI and to raise awareness (especially with hunters) of the risks dogs pose to kiwi.

Long term objectives

- To establish a self-sustaining population of NIBK in BSMI through integrated management techniques.

- To contribute to the further development of techniques used in kiwi egg incubation, captive rearing, release of juveniles and subsequent monitoring, and to allow for research into aspects of the process to be undertaken.

4.2.3 Methods

- ***Reintroduction and monitoring kiwi in BSMI***

If kiwi numbers within BSMI dropped below 20 individuals, the kiwi population was supplemented with ONE juveniles (>800g) from the EKFP.

Techniques used to attach transmitters and monitor kiwi in BSMI have followed methods described in the Kiwi Best Practice Manual (Robertson *et al*. 2003 OLDDM-643237). The dispersal and survival of juvenile kiwi were monitored every other day, and weekly for territorial adults. The breeding activity of paired kiwi and kiwi of breeding age was checked through late May to late March. Nests were visited at night while males were off feeding and the age of eggs was determined by candling. If the eggs were fertile, the nest was revisited at 80-85 days since the start of incubation to band the first chick and at 90-110 days to band the

second chick. All infertile eggs were removed from the nest after the male had deserted. Some of these were sent to Suzanne Basset to assess the cause of failure and others were filled with Plaster of Paris to be used for display.

All chicks hatched within BSMI were fitted with a non-mortality chick transmitter (5.5g) at four to ten days of age and the transmitter was changed to mortality chick transmitter (11g) when chicks weigh between 400-500g. While chicks were carrying a non-mortality transmitter, their survival was monitored either by remote tracking at night to determine their movements or checking their locations during the day for two consecutive days. If movement of the chick was not detected, the bird was physically checked. Feather samples taken from chicks were sent to Jennifer Anderson (Allan Wilson Centre Genome Service, Massey University, Auckland) for DNA sexing.

- ***Kiwi call count survey***

As part of a long term study on the kiwi population change at BSMI, six listening sites were established throughout the Reserve (Appendix 1), based on protocols for the kiwi call scheme (see Kiwi Best Practice Manual for details OLDDM-643237). This is the first large scale kiwi call count survey within the Reserve, with hours of listening totalling 40 (i.e. the survey was conducted for 2 hours for 4 nights at each listening stations). Due to adverse weather conditions, the target of 48 hours was not met. The survey was conducted no earlier than one hour after dark on fine calm nights. Any kiwi heard during and outside listening hours were recorded on a standardised kiwi call scheme card.

- ***Necropsy of dead kiwi***

If a kiwi was found dead a thorough examination of the site was carried out to determine predator sign, and detailed notes made of how the body was found (see Kiwi Best Practice Manual (Robertson *et al.* 2003) for details). Kiwi not obviously killed by a predator were chilled in a refrigerator and sent for necropsy to the Wildlife Health Unit, Institute of Veterinary, Animal and Biomedical Sciences, Massey University. A full necropsy was carried out immediately on any kiwi obviously killed by a predator, or the body was frozen for later examination. All the findings were collated to determine the most likely predator (see Cuthbert, 2003; Lond, 1971; Lyver, 2000; Ratz et al, 1999; Ratz & Moller, 1997 for predator identification from bite marks).

- ***Search for missing kiwi***

If a bird was ‘lost’ to the monitoring population because it’s transmitter either failed or dropped prior to transmitter change, a number of attempts to recapture the missing bird were made. This included listening for the missing bird at night in his/her known territory followed by thorough search of the area by a kiwi dog and its handler during day.

4.2.4 Results

• *Reintroduction and monitoring of kiwi in BSMI*

No ONE birds were released into BSMI during this period. No losses (mortality or dispersal) of sub-adult and adult kiwi released prior to 2005/06 occurred. Of 17 sub-adult/adult birds, ten have formed pairs and bred this season.

The 2005/06 breeding season

There were 9 nesting attempts recorded this season (see Table 1 for the summary of the 2005/06 breeding season). In total, five pairs produced 15 eggs and hatched 10 chicks (80% fertility rate, 66.7% hatching success, and 83.3% hatching success of fertile eggs). Of 15 eggs, two died at late stage of incubation (one egg died at hatching and another died at 65-73 days old due to an unknown cause of death). Two chicks were not banded because they had hatched earlier and left their nest sooner than expected. In addition, a sub-adult male, *Koha* built two phantom nests during this period.

Pair	Clutch	Egg	Egg fertility	Chick	Sex	Estimated Hatch date	Current status (age as of June 30 2006)
Marama – Iwik	1	1	Fertile	**Podos**	Female	31/08/05	Alive (303 days old)
		2	Fertile	**Kairiri**	Male	14/09/05	Alive (289 days old)
	2	1	Fertile	Unbanded	-	-	-
		2	Fertile	**Shine**	Female	18/01/06	Alive (163 days old)
Hiccup – Mozza	1	1	Fertile	**Tui**	Female	25/09/05	Found dead 26/12/06 (83 days old at death) - stoat predation.
	2	1	Infertile	-			
		2	Fertile	- (egg died at 65-73 days old)			
	3	1	Fertile	**Winepare**	Female	24/02/06	Alive (126 days old)
Warren – Rikoriko	1	1	Fertile	**Genevieve**	Female	08/01/06	Found dead 01/04/06 (59 days old at death) – fell into a tomo
Ariki – Marangai	1	1	Infertile	- (egg rotten)			
		2	Fertile	**Maxwell**	Female	18/11/05	Alive (224 days old)
Oliver – Tufty	1	1	Infertile	- (egg rotten)			
		2	Fertile	- (egg died at hatching. Also had deformed umbilicus)			
	2	1	Fertile	Unbanded	-	-	-
		2	Fertile	**Heays**	Male	06/01/06	Drowned 12/02/06 (32 days old at death)

Table 1 The 2005-06 NIBK breeding summary at BSMI.

• *Progress of kiwi chicks hatched at BSMI during the 2005-06 season*

Of eight chicks hatched at BSMI this season, three were lost: one suspected to be killed by a stoat (*Tui*), one drowned (*Heays*) and one fell into a tomo (*Genevieve*) (See Table 1 in the

previous section). The age of these three chicks at the time of death ranged between 32 and 83 days old (average 58 days old). *Heays* drowned in a creek in the Tumanako block on 12/02/06 after beeping mortality but confirmed alive on the previous day. His body was sent to Brett Gartrell, Massey University for necropsy, and the result indicated that he was suffering from an infection in his yolk sac prior to his death.

All chicks (<304 days old) have stayed within one kilometre of their natal territories. Figure 1 shows the weight increase of chicks hatched in the wild during this period. All chicks except *Heays* gained weights over each routine checks.

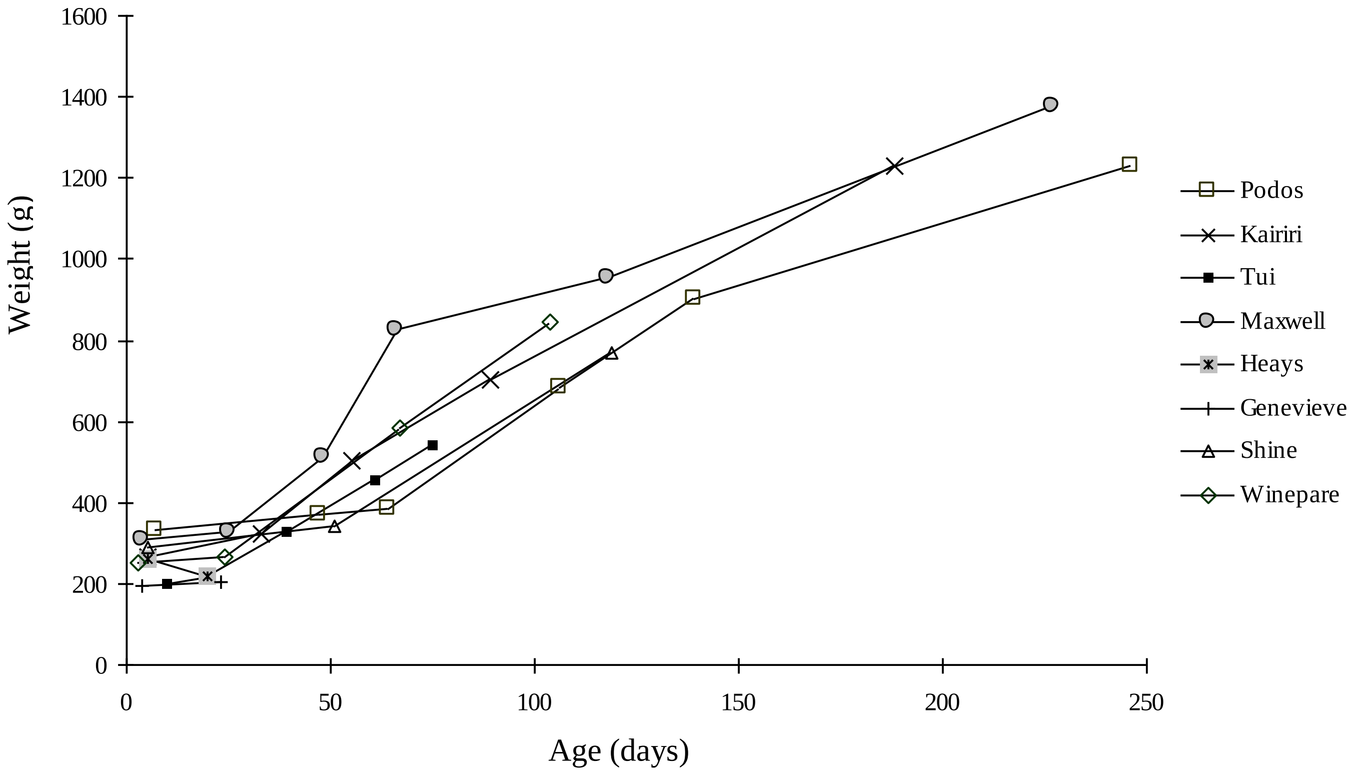

Figure 1 The growth of eight wild kiwi chicks hatched in BSMI during the 2005-06 period.

- ***Losses of sub-adult and adult kiwi***

Apart from the deaths of three kiwi chicks, there was one other kiwi death recorded this period. A sub-adult male, *Patch* fell into a tomo and was found in April 2006. In addition, five kiwi have been lost due to the fact that their transmitters had either failed or dropped prior to transmitter change. Transmitter failure was confirmed 09/09/2006 for *Cocoa*, 15/09/2005 for *Makino*, 27/03/2006 for *Oliver*, 31/03/2006 for *Warren* and *Mozza*'s transmitter fell off 26/04/2006. The transmitter of a juvenile kiwi, *Tui*, also failed on 06/12/05, but she was rediscovered three days later by *Pounui*, a certified kiwi dog.

Despite the production of ten chicks, due to the high number of mortality and transmitter failure, the total number of kiwi monitored in BSMI is now 19 (seven males and 12 females). Twenty of 34 kiwi released into BSMI since 2000 have been ‘lost’; one kiwi to dispersal, one

to *Coccidia*, four accidents, eight predation and six transmitter failures (see Table 2). Five of ten kiwi chicks hatched at BSMI since 2005 have been also lost; one predation, one transmitter failure and three accidents (see Table 3). There are a number of kiwi (at least four birds) which are not monitored with transmitters, but otherwise confirmed present within BSMI (see the section on the kiwi call count survey).

- ***Kiwi call count survey***

A total of 40 hours of listening was conducted between April and June 2006. The total number of kiwi heard was 19 (average 0.475 kiwi call/hour) (See table 2). During the survey, a male, *Cocoa,* whose transmitter failed in September 2005 was confirmed alive at the bottom of the 'Kamahi' block. An unknown male was also heard calling in close proximity of stream fenn 4 in the 'Te Tatimana' block.

**Location**	**Hours listened**	**Number of kiwi heard**		**Average kiwi call (call/hour)**
		Male	Female	
Top of the 'Tui' block	8	2	0	0.25
Goat hill	8	9	0	1.125
Crockery knob	4	2	1	0.75
Top of the 'Podocarps' block	6	1	0	0.17
Airstrip	8	2	2	0.5
Top of the 'Section 4' ridge	6	0	0	0
**Total**	**40**	**16**	**3**	**0.475**

Table 2 The result of kiwi call count survey conducted between April and June 2006.

- **Search for missing kiwi**

Several attempts have been made to locate two missing adult males, *Warren* and *Oliver*, both by listening in their territories at night and by using kiwi dogs during day. Both males have been heard calling at night. To date however, neither of them have been recaptured..

Table 2 Details for kiwi released into BSMI since 2000. Kiwi in **bold** are those which are either dead or which we have lost contact with.

Kiwi	Sex	Age at release (days)	Age (days) at June 30 2006 or when lost or found dead	Release weight (g)	Max weight loss (g)	Time (days) before weight increase	Time taken to regain release weight (days)	Maximum distance (m) moved from release site	Weight (g) at June 30 2006 or when lost or found dead	Current status
**Ari**	**M**	**80**	**286**	**1150**	**150**	**26**	**95**	**2857**	**1325**	**Found dead**
**Raina**	**F**	**70**	**623**	**850**	**100**	**41**	**57**	**3857**	**1750**	**Unknown**
**Kohu**	**M**	**132**	**1679**	**1000**	**250**	**360**	**76**	**6km+**	**-**	**Found dead**
Manu-iti	F	102	2083	875	200	644	0	1464	2840	In reserve
**Akenehi**	**F**	**105**	**539**	**850**	**0**	**29**	**0**	**1214**	**1800**	**Found dead**
**Kote**	**M**	**139**	**232**	**960**	**0**	**11**	**0**	**446**	**1050**	**Found dead**
**Grace**	**F**	**96**	**417**	**950**	**25**	**23**	**23**	**430**	**1900**	**Found dead**
**Kiri**	**F**	**118**	**411**	**925**	**75**	**23**	**36**	**1750**	**1600**	**Found dead**
**Whiri**	**M**	**99**	**1192**	**900**	**100**	**-**	**34**	**8400**	**1650**	**TX failed**
**Puke**	**M**	**118**	**418**	**875**	**0**	**43**	**0**	**1286**	**1425**	**Found dead**
Marangai	F	107	1759	1050	300	-	47	9286	2350	In reserve
**Honey**	**F**	**108**	**217**	**1100**	**0**	**0**	**0**	**107**	**1300**	**Found dead**
**Mohaka**	**M**	**206**	**1194**	**925**	**150**	**-**	**0**	**3142**	**-**	**Found dead**
Iwik	F	108	1691	1050	85	-	0	1348	2430	In reserve
**Koha I**	**M**	**149**	**159**	**900**	**-**	**-**	**-**	**-**	**-**	**Unknown**
Marama	M	154	1737	1025	100	46	46	1349	1300	In reserve
Koha II	M	192	1672	975	25	56	56	1339	1480	In reserve
**Cocoa**	**M**	**158**	**1343**	**1050**	**100**	**34**	**100**	**1991**	**1475**	**Tx failed**
**Oliver**	**M**	**148**	**1184**	**1100**	**175**	**61**	**55**	**571**	**1560**	**Tx failed**
Hiccup	M	192	1335	1050	50	340	147	816	1640	In reserve
**Aruhe**	**F**	**163**	**281**	**1125**	**-**	**-**	**-**	**-**	**-**	**Found dead**
Tufty	F	148	1271	1150	0	-	-	684	2050	In reserve
**Albert**	**M**	**190**	**581**	**1000**	**75**	**76**	**76**	**2765**	**1575**	**Found dead**
**Mozza**	**F**	**145**	**1147**	**1200**	**125**	**76**	**136**	**1253**	**2190**	**Tx dropped**
**Warren**	**M**	**283**	**1129**	**1300**	**25**	**48**	**48**	**1373**	**1920**	**Tx failed**
Rima-tekau	M	185	1132	1150	100	42	86	859	1490	In reserve
**Ora**	**M**	**130**	**530**	**1146**	**75**	**35**	**144**	**3030**	**875**	**Found dead**
**Karanga**	**F**	**147**	**585**	**1098**	**98**	**82**	**82**	**3879**	**1475**	**Found dead**
Rikoriko	F	207	991	1100	50	39	39	1294	2200	In reserve
Strauss	F	215	999	1225	225	39	98	1301	2640	In reserve
Alby	F	139	896	1100	0	0	0	5047	2040	In reserve
Ariki	M	154	911	1100	275	92	42	2110	1850	In reserve
Sorcha	F	190	895	1143	68	44	44	711	2330	In reserve
Fontane	M	163	602	1230	5	77	77	2944	1455	In reserve

Table 3 Details for kiwi hatched at BSMI. Kiwi with * hatched during the 2005-06 period. Kiwi in **bold** are those which are either dead or which we have lost contact with.

Kiwi	Sex	Parents	Weight when found (g)	Maximum distance (m) moved from nest	Age (days) at June 30 2006 or when lost or found dead	Weight (g) at June 30 2006 or when lost or found dead	Fate
**Makino**	**M**	**Marama/Iwik**	**202**	**1498**	**210**	**1012**	**Tx failed – 'lost' from monitoring population**
**Patch**	**M**	**Marama/Iwik**	**381**	**1202**	**389**	**1250**	**Found dead in a tomo**
*Podos	F	Marama/Iwik	330	1375	303	1230	Alive
*Kairiri	M	Marama/Iwik	267	1182	289	1230	Alive
***Tui**	**F**	**Hiccup/Mozza**	**201**	**911**	**92**	**590**	**Killed by a stoat**
*Maxwell	F	Ariki/Marangai	310	No GPS data available	224	955	Alive
***Heays**	**M**	**Oliver/Tufty**	**261**	**No GPS data available**	**37**	**219**	**Drowned**
***Genevieve**	**F**	**Warren/Rikoriko**	**197**	**No GPS data available**	**83**	**206.5**	**Found dead in a tomo**
*Shine	F	Marama/Iwik	291	No GPS data available	163	770	Alive
*Winepare	F	Hiccup/Mozza	250	802	126	845	Alive

- ***Public awareness***

Nest checks, banding of chicks and routine checks of adults were often assisted by locals or long-term volunteers. A journalist from the Napier Courier attended a transmitter change and wrote an article on the kiwi reintroduction project at BSMI. Kiwi listening was a draw card in two 'walk and talk' nights held in December 2005 and January 2006 organised by the Department of Conservation in conjunction with the Hawke's Bay Regional Council/Sport Hawke's Bay. More than 40 people attended each event.

4.2.5 Discussion

It has been a very successful breeding season this year, with the arrival of eight chicks including a chick from 17 months-old male, '*Ariki'*. However, the high mortality of these chicks due to accidents may indicate that the terrain of BSMI is harsh for kiwi chicks. If the chick mortality remains high in the next few years, it may be necessary to take some management actions to prevent further losses (i.e. ONE).

The rate of transmitter failure remained very high this year. Four out of 18 adult transmitters are suspected to have failed well in advance of the end of their expected battery life. Product quality and reliability is a major concern. If birds are found with transmitters which show signs of wearing out (e.g. epoxy peeling off, and damaged aerial) or low battery (e.g. weak signal and limited signal range) during regular checks, they must be replaced. All new transmitters should also be checked for such faults before being taken into the field, and any transmitters which do not meet the standard should be sent back to the manufacturer. In addition, in order to eliminate potential transmitter failures, it may be necessary to review the storage method and timing of purchase currently practiced at BSMI.

This year's kiwi call count survey provided the start of baseline data for the current population of BSMI kiwi. According to the Kiwi Best Practice Manual, call count surveys must be undertaken three consecutive years in order to obtain baseline data. Providing that resources are available next year another call count survey should be conducted.

Egg candling appears to be a relatively accurate way of determining the age of kiwi eggs and thus the timing of chick banding. As more pairs are expected to form and breed in the future, training more DoC staff and volunteers on candling and nest checks is required.

4.2.6 Recommendations

- Ensure all banded chicks are regularly monitored and every effort is made to ascertain cause of death.
- Ensure radio-tagged birds are fitted with the best transmitters available and replace any faulty transmitters as necessary.
- Discuss the best storage method and timing of transmitters with manufacturers.
- Conduct kiwi call count survey in April 2007 if resources are available.
- Train DoC staff and volunteers in kiwi egg candling and nest checks.

References

Cuthbert, R. (2003). Sign left by introduced and native predators feeding on Hutton’s shearwaters *Puffinus huttoni*. *New Zealand Journal of Zoology*. Vol 30: 163-170.

Lond (1971). Notes from the Mammal Society – No. 22. *Journal of Zoology*. Vol 164: 239-270.

Lyver, P. O’B. (2000). Idendifying mammalian predators from bite marks: a tool for focusing wildlife protection. *Mammal Review*. Vol 30, No. 1, 31-44.

Ratz, H., and Moller, H. (1997). *Identification of predators of Royal Albatross chicks at Taiaroa Head in February 1994*. Conservation Advisory Science Notes: 138. Department of Conservation.

Ratz, H., Moller, H., & Fletcher, D. (1999). Predator identification from bite marks on penguin and albatross chicks. *Marine Ornithology*. Vol 27: 149-156.

Robertson, H., Colbourne, R., Castro, I., Miller, C., & Cresswell, M. (2003). *Kiwi (Apteryx spp.) Best Practice Manual.* OLDDM-643237. Department of Conservation.

Appendix 1: Kiwi call count listening sites

Appendix 2: Map of territories of sub-adult and adult kiwi at BSMI

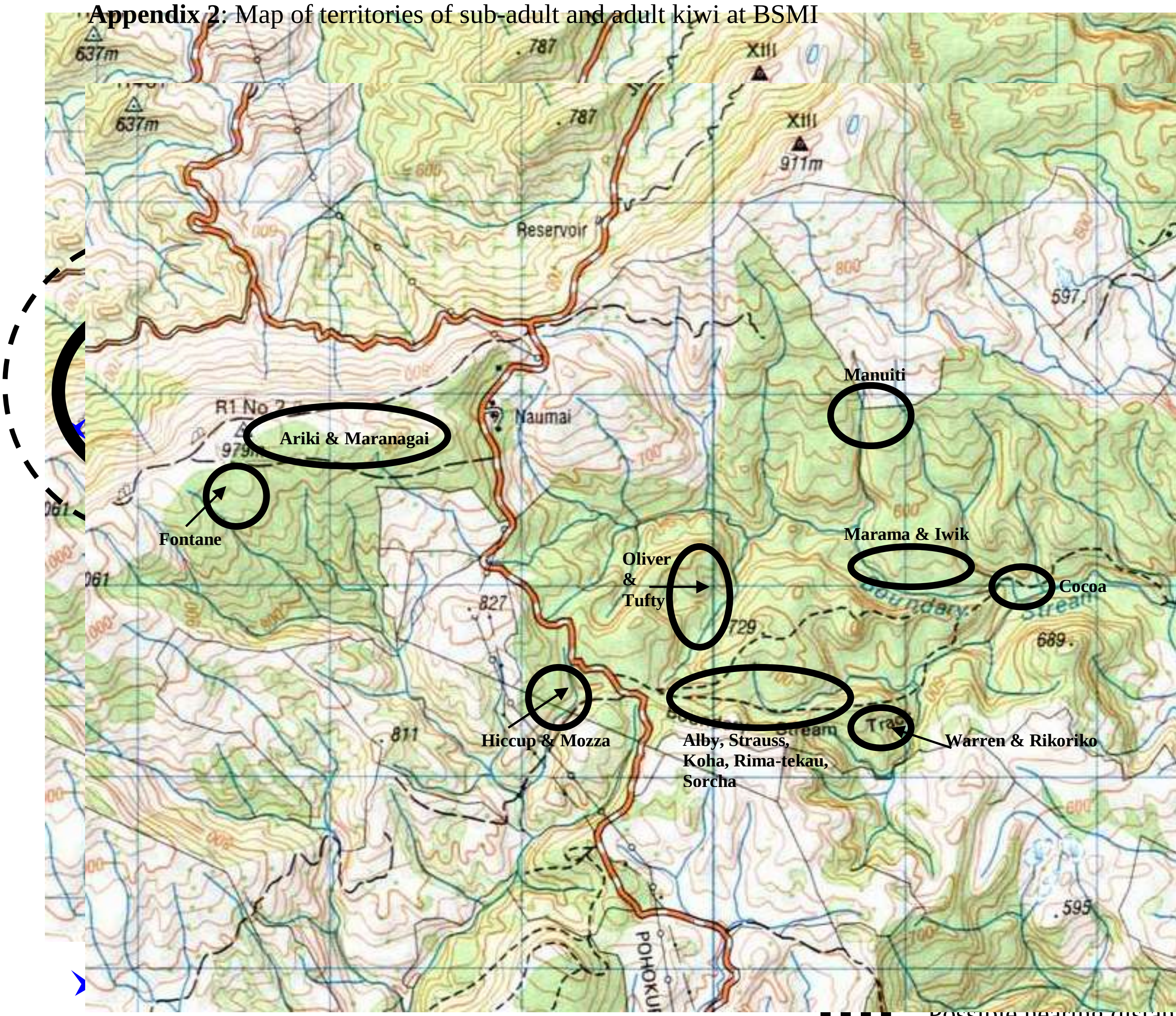

Listening sites for the 2005-06 kiwi call count survey

Possible hearing distan[illegible]

4.3 Reintroduction of North Island kokako *(Callaeas cinerea wilsoni)*

4.3.1 Summary

The kokako population at Boundary Stream was surveyed prior to the breeding season to determine the number and location of birds, and identify potential breeding pairs. 16 birds were located, including two breeding pairs. The breeding success of pairs was monitored. From four viable nests located, five chicks were produced raising the number of kokako confirmed in the reserve to 21 at the end of the breeding season.

4.3.2 Objectives

Short-term objectives

- To monitor the survival and location of kokako through annual surveys.
- To determine breeding success through locating nests and monitoring banded chicks.
- To provide a 'hands-on' volunteer project to encourage local community interest and involvement.

Long-term objectives

- To establish a wild self sustaining population of kokako as part of biodiversity restoration at BSMI.
- To strengthen the advocacy role of BSMI through enabling the public of Hawke's Bay and New Zealand to witness and appreciate the value of a restored indigenous ecosystem through returning kokako to part of their natural range.
- To contribute to the further development of knowledge and practices associated with translocating and managing kokako on the mainland.
- To help fulfil the goal within the North Island kokako recovery plan: *To improve the status of North Island kokako from 'endangered' to 'threatened' by restoring the national population to ca 1,000 pairs, within sustainable communities throughout the North Island, by the year 2020.*

4.3.3 Methods

Pre-breeding survey: The reserve was surveyed on a 150 x 150 metre grid from August to October, using the existing bait station lines. As per the guidelines provided by the Kokako Recovery Group, at each station three pre-recorded mew calls were played, followed by a five minute listening period. This was then repeated, before playing 30 seconds of local song, followed by another five minute listening period.

Monitoring of breeding success: Pairs located during the October survey were followed on a regular basis (weekly, wherever possible) to look for behaviour indicative of nesting. Where nests where located, they were also checked weekly and watched for a minimum of two hours per visit.

Where chicks were hatched, they were banded between 10 and 20 days of age, and only if their weight exceeded 100g. Chicks were fitted with a stainless steel 'E' size band, and a unique combination of plastic colour bands. Weekly monitoring of nests continued until chicks were fledged.

For more information on any of these methods, refer to the Kokako management folder, Threatened Species Occasional Publication 19.

4.3.4 Results

Pre-breeding survey: A total of 16 birds, out of 19 potentially live in the reserve, were found during the pre-breeding survey. The kokako found consisted of two breeding pairs, two juvenile pairs, two female-female pairs, and four singles. Three known birds were not located, and one bird was found dead on 12/9/05 (table 4.3.1). The dead kokako was sent to Massey University for necropsy, the results of which indicated the kokako had suffered severe haemorrhaging around the lower abdomen, probably from blunt trauma.

Table 4.3.1 All known Boundary Stream kokako, and their status as at October 2005

**Bird (sex)**	**Mate (sex)**	**Location**	**Status**
WY-BM (♂)	YR-WM (♀)	Tumanako	Breeding pair
M-YG (♂)	RG-(R)M (♀)	Tumanako	Breeding pair
M-Y (♂)	M-RY (♀)	Wallow	Juvenile pair
M-YW (♂)	M-G (♀)	Tumanako	Juvenile pair
YM-O (♀)	R-M (♀)	Te Tatimana	Female-female pair
BM-RO (♀)	RM-^{L}G (♀)	Podocarps	Female-female pair
Y^{L}G-WM (♂)	-	Te Tatimana	Single
M-B (♀)	-	Tumanako	Single
O-M (♀)	-	Tui	Single
M-W (♀)	-	Tui	Single
M-W (♂)	-	-	Not located
M-RW (♀)	-	-	Not located
GM-R (♀)	-	-	Not located
OM-G (♂)	-	-	Confirmed dead

Monitoring of breeding success: M-YG and RG-(R)M were found to be nesting on 15/11/05. At this time the female RG-(R)M was incubating eggs. Unfortunately, the nest was found to have failed on 29/11/05, following a period of very heavy rain. One

egg, which was infertile had been partially eaten, probably by a rat (I. Flux, pers. comm.). The second egg was still whole, and contained a dead embryo which had died at eight days.

On 23/12/05 M-YG and RG-(R)M were found to be re-nesting, at the incubation phase. Two chicks from this nest were banded on 18/1/06 (M-^{L}GR, ♂; M-Y^{L}G, ♀), and fledged around 1/2/06.

WY-BM and YR-WM were first found to be nesting on 19/10/05 while at the nest building phase. Two chicks from this nest hatched around 26/11/05, and were banded on 8/12/05 (M-OY, ♂; M-OW, ♀). These chicks fledged between 25/12/05 and 1/1/06.

WY-BM and YR-WM were found to be re-nesting on 30/1/06, again while at the nest building phase. One chick hatched around the 5/3/06, was banded on 21/3/06 (W^{L}G-M, ♀), and fledged around 7/4/06.

One of the female-female pairs (YM-O and R-M) were found to be nest building on 8/11/05, although no sign of incubation was seen (table 4.3.2).

Table 4.3.2 Summary of kokako breeding 05-06

Pair	Nesting confirmed	Stage when found	Estimate start of incubation	Estimate date hatched	Chicks	Date banded	Estimate Date fledged
Aviary pair ♂M-YG ♀RG-(R)M	15/11/05	Incubating eggs	Around 15/11/05	nest failure confirmed 29/11/05	-	-	-
	23/12/05	Incubating eggs	Around 15/12/06	4/1/06	♂M-LGR ♀M-YLG	18/1/06	1/2/06
Tumanako pair ♂WY-BM ♀YR-WM	19/10/05	Nest building	8/11/05	26/11/05	♂M-OY ♀M-OW	8/12/05	Between 25/12/05 and 1/1/06
	30/1/06	Nest building	15/2/06	5/3/06	♀WLG-M	21/3/06	Around 7/4/06
Female/female pair in Te Tatimana ♀ YM-O ♀ R-M	8/11/05	YM-O building a nest	No eggs laid	-	-	-	-

4.3.5 Discussion

The success of the 2004-05 breeding season for the kokako has continued into 2005-06. Although only two pairs bred this year, compared to three for the previous year,

the addition of five fledglings is a valuable contribution to the population. This success also indicates that Boundary Stream is a suitable habitat for kokako, and that the current level of predator control is adequate to allow successful breeding. The survival rate of these fledglings, however, will only become clear over time. The skew in the population towards female kokako, however, is cause for concern.

This was the first season at Boundary Stream where the kokako had to be monitored without the use of transmitters. While this led to extra pressure on staff and time resources, the excellent guidelines provided by the Kokako Recovery Group allowed an efficient pre-breeding survey and provided useful advice for monitoring the breeding behaviour.

As with any population founded by a small number of individuals, there is a risk of inbreeding depression developing at Boundary Stream. The Kokako Recovery Group has recommended the input of unrelated birds to increase the genetic variation of the Boundary Stream population. It is planned to release a further five pairs of kokako into Boundary Stream from the Otamatuna population, in Te Urewera National Park. This population has a high degree of genetic diversity and has the greatest potential to alleviate the potential genetic bottleneck at Boundary Stream.

4.3.5 Recommendations

- Continue to monitor the Boundary Stream kokako population through annual pre-breeding surveys.
- Continue to monitor pairs located during the pre-breeding survey for the duration of the breeding survey.
- Continue to band chicks.
- Maintain pest control either at the current level, or at a more intense level to keep predators at low enough levels to allow successful kokako breeding.
- Transfer 10 more kokako from Otamatuna in 2006/07.

4.4 Reintroduction and monitoring of North Island saddleback (tieke; *Philesturnus carunculatus rufusater*)

4.4.1 Summary

After the failure of the 2004 saddleback transfer the decision was made to transfer another 40 birds from Mokoia Island in March 2006. However before attempting to transfer more saddleback a trial was undertaken to investigate the use of backpack-mounted transmitters on saddleback on Mokoia Island. In April 2006 the decision was made to delay the transfer for a year due to concern at the high rat tracking indices. Details on the revised transfer plan and the transmitter trial are provided.

4.4.2 Objectives

Short-term objectives

- To reintroduce North Island saddleback to BSMI
- To monitor the presence and dispersal of saddleback as an indication of a successful transfer

Long-term objectives

- To monitor survival and breeding success of saddleback to determine the management level required to establish and maintain a self-sustaining population of saddleback in BSMI

4.4.3 Methods

In January 2005 it became apparent that the transfer of saddleback from Cuvier Island to BSMI had failed (BSMI 2004/05 Annual Report). The last sighting of a live saddleback in Boundary Stream took place in October 2005. It was decided to attempt another transfer of another 40 birds from Mokoia Island.

Mokoia supplementary transfer
The proposal to translocate 40 saddleback from Mokoia Island to BSMI was accepted in July 2005 (Sullivan, 2005). Based on the lessons learnt from the Cuvier Island transfer, a number of key changes were considered.

Disease screening
With the increased awareness of the impact of disease during transfers, it was decided to repeat the methods from the Cuvier transfer. This is by screening every bird for select diseases, hold the birds in captivity until the results show negative, then transport and release the birds. Due to the impact of the fungal disease Aspergillosis on the first transfer, it was decided to prophylactically treat all 40 birds while in

captivity. However, for this transfer the aviaries were adequate to hold the birds for extended periods of time if necessary. This would eliminate the need to transport them to a holding aviary and exacerbate any stress related diseases. The climate of Mokoia Island is within approximately two degrees of BSMI in May, and this will help the birds acclimatise to BSMI.

Timing

Late autumn was chosen as the birds should be at a heavier weight than prior to spring (K. Hale, pers. comm.). The birds should be able to become acclimatised before the coldest winter months. A capture in late autumn will prevent disturbing any breeding on Mokoia Island

Transport

The birds will be transported by helicopter, a 45 minute flight to BSMI, in modified cat boxes.

Transmitter trial

Due to the difficulty in finding non-transmittered birds, Kiwitrack Ltd, Havelock North, designed a lightweight backpack transmitter. The transmitters had an expected battery life of ten months. The transmitter weighed no greater than 2.7g. and were designed to be used in conjunction with a harness made by Sirtrack (0.2g; 1.5mm diameter "clout type" cord). The whole package weighed 4% of the average saddleback body weight.

All saddleback were to be fitted with a transmitter immediately prior to transit, provided the weight of the transmitter did not exceed 4% of the birds weight.

While the tail-mounted transmitters initially provided essential information at BSMI, such as dispersal and survival rates, they detached within 8-10 weeks. A considerable amount of information could not be obtained, particularly survival over the breeding season and breeding success rates, due to the difficulty in monitoring non-transmittered birds. As backpack transmitters had not been used on saddleback previously, the transmitters were trialled on six birds on Mokoia Island from September 2005 to January 2006.

Transmitters were attached to six saddleback, four males and two females, deemed all adults and potentially breeding birds at the time of capture. The birds were caught using 9 or 12 m mist nets, with a mesh size of 38 mm. Harnesses were secured by knotting and gluing the knots with superglue. Time to attach the transmitters took approximately 20 minutes each bird. The birds were banded with one or two colour bands and one metal band to assist with identification if the transmitter failed.

The saddleback were checked daily for the first eight days to ensure survival was not affected. Once the transmitters were deemed to be safe, the rate of monitoring was reduced to twice weekly. Each bird was monitored and time budgets collected until approximately 30 minutes of data was obtained per sampling period. A total of 30 non-transmittered saddleback, found in the same areas as the transmittered saddleback, were also monitored and were used as a comparison.

Behaviours were divided into categories foraging, commuting, nesting, preening resting and interacting (Table 4.4.1). An additional category "ground" was included where all behaviours were grouped together if the bird was less than 50 cm from the ground. This was due to the hypothesis that transmitters may cause the birds to spend more time on the ground due to increased energy expenditure, making them more prone to predation. It was also predicted that transmitters may cause an increase in preening due to skin abrasions, feather wear or attempts by the bird to dislodge the

transmitter. Resting was deemed to be an important factor to watch as increased energy expenditure of the bird resulting from carrying extra weight may cause it to rest more; likewise increased energy expenditure may cause the bird to need to forage more. However, if a bird needed to rest or preen more due to the transmitter, it may result in less time available to feed. Commuting was deemed important as the weight of the transmitter may interfere with flight, and indirectly impact on interaction with mates and competitors. Decreased ability for flight would also cause the bird to be more prone to predation.
Whether the birds lost condition due to the transmitters was not tested, as it would be impossible to determine if any weight changes were due to seasonal changes in food or breeding status. It was deemed sufficient that any changes caused by loss of condition would be detected in the behaviour.

Table 4.4.1: Definitions of behavioural categories

Ground	Any behaviour (foraging, calling, preening etc) that occurs within 50 cm from the ground
Foraging	Foraging
Interacting	Bonding behaviour including mutual preening, copulating, courtship feeding, following. Excluding calling.
Preen	Excluding mutual preening
Rest	Sitting, sleeping
Commuting	Flying, chased, chasing, jumping from branch to branch; excluding branch to branch while foraging,

Measure of success

The trial was deemed successful, and the transmitters acceptable for use if:

- No less than 50% (three) remain on the birds by the end of January
- There is no perceived impact, or potential impact, on survival
- There is no perceived impact on breeding success, compared to non-transmittered saddleback
- There is no detrimental behavioural patterns perceived to be caused by the transmitter, eg more time being spent on the ground
- There are no injuries caused by the backpack transmitter system

4.4.4 Results

Transmitter trial
One male was found dead four days after the transmitter was attached. Necropsy results strongly suggested stoat predation. Unfortunately, this bird was only monitored for 33 minutes in the three days prior to his death. In this time he spent 79% of that time on the ground. While this seems extremely high, the short monitoring period may have skewed that result making it hard to determine if the transmitter caused it to be more prone to predation. One female was rarely able to be monitored due to her high flighted activity. It became apparent that she did not have a

territory and was possibly a juvenile. Her signal was last heard on the 25th November, and it is possible the transmitter may have failed.
The four remaining saddleback were observed regularly for the remaining study period. The transmitters remained attached to all four, and were still going on three birds after 18 weeks.
Overall, there was no significant perceived impact on behaviour by the transmitter (One-way ANOVA; P= 0.815; figure 1). In particular, there was no difference in time spent on the ground of the remaining transmittered saddleback compared to non-transmittered saddleback, and while on the ground, there was no difference in behaviour, except that non-transmittered birds commuted more often. However, as there were only five incidences of non-transmittered birds commuting along the ground, it is not deemed to be critical. Overall, there were no differences in preening, resting, interacting or commuting. There was a slight difference in foraging behaviour, but this difference is probably due to the fact that one of transmittered birds (M-W) was breeding and foraged less. Unfortunately, due to the difficulty in tracking unmarked birds, we could not determine if there were differences in breeding success.

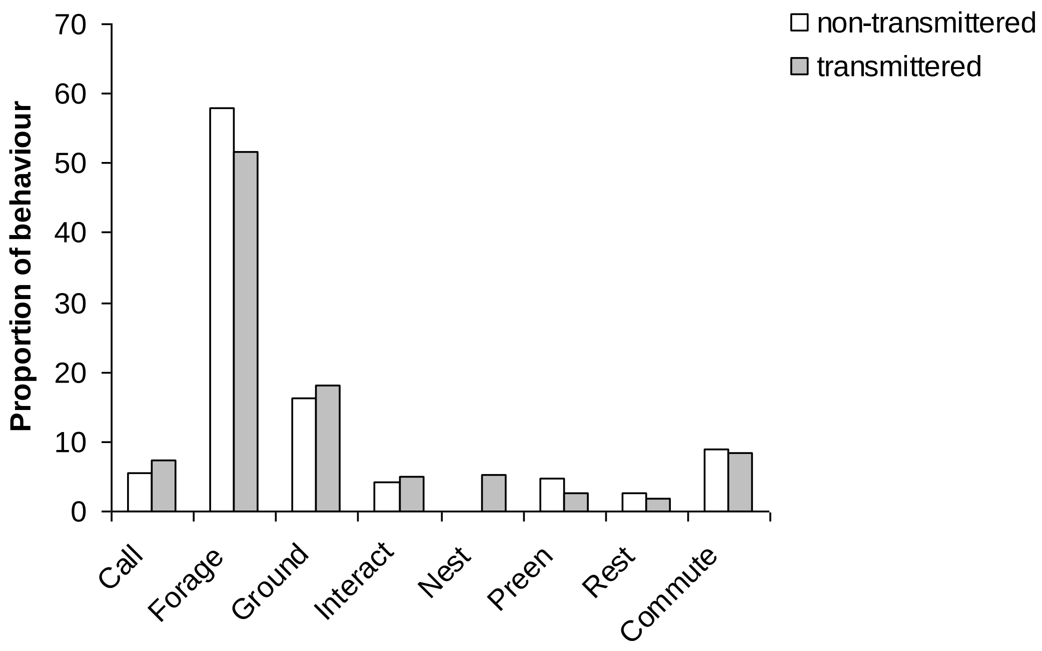

Figure 4.4.1: Proportion of time spent on behaviour.

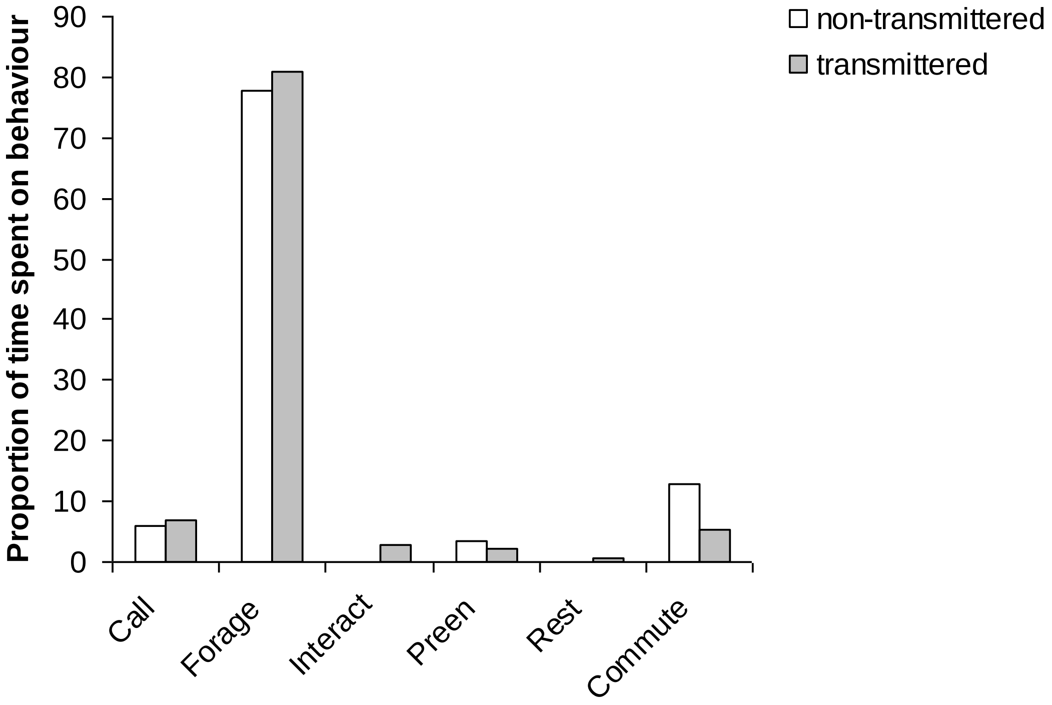

Figure 4.4.2: Proportion of time spent on behaviour while within 50cm of the ground.

The other four birds (three males and one female) all remained within their territories. The female (M-W) nested and produced two eggs, one which successfully hatched and fledged. Two males (G-M and R-M) were confirmed to have at least one fledgling each when the birds were checked on the 9 March 2006.

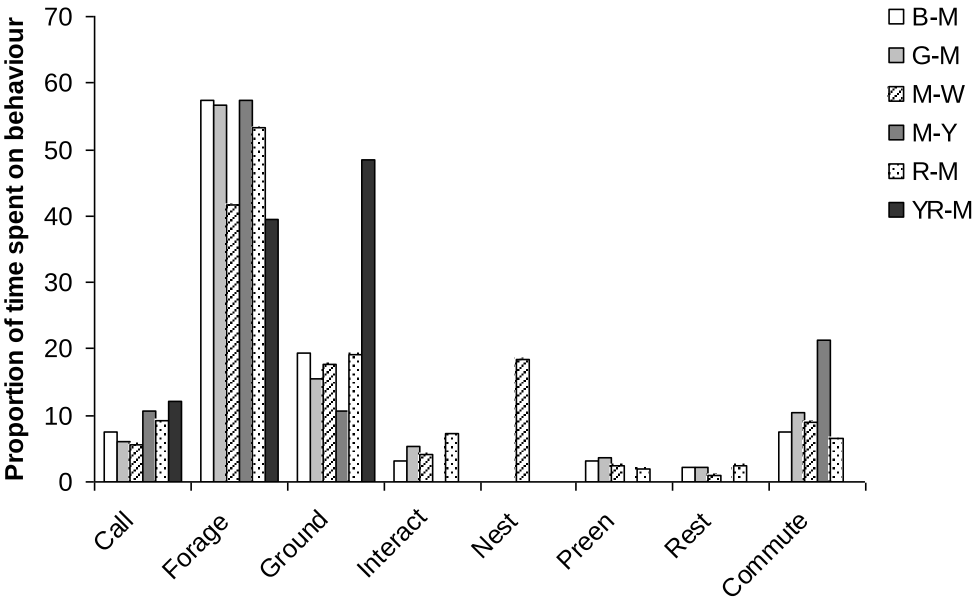

Figure 4.4.3: Proportion of time spent of defined behaviour by each transmittered bird.

Rodent tracking results

Mid February's rodent tracking results indicated that rat numbers had risen beyond an acceptable level to transfer saddleback. The decision was made to immediately change the poison from Ditrac to Racumin, and retest 10 days prior to the proposed transfer date. The rodent tracking tunnels were run on the 18-19th March, and while activity had decreased, it was still considered too high to proceed with the transfer. The transfer date was postponed to mid April 2007, dependant on rodent activity over the next 12 months.

4.4.5 Discussion

Transmitter trial
While only broad behavioural patterns were observed in this study in a small sample size, transmitters did not appear to impact on survival or detrimentally alter behaviour.
To assess whether the transmitters are acceptable for use by comparison with the measures of success:

- *No less than 50% (three) remain on the birds by the end of January* – transmitters remained on all four of the remaining monitored birds. One transmittered failed after seven weeks. No signal was received from the dispersed juvenile female after nine weeks, but because this bird could not be monitored adequately, it could not be confirmed if the transmitter failed, or the bird dispersed out of range.
- *There is no perceived impact, or potential impact, on survival* – one male died after four days of transmitter attachment from stoat predation. Because of the short time frame, data could not be collected on whether he was more vulnerable to stoat predation than non-transmittered birds, however, results from the entire trial suggest that this is not the case. Estimated annual survival rates were estimated to be approximately 89% in 1997 when the population was still expanding (Armstrong et al. 2005). With the trial population, survival was 83%, although it is possibly not comparable, as the male was predated on by a stoat in a presumably predator free environment.
- *There is no perceived impact on breeding success, compared to non-transmittered saddleback* – Three of the transmittered birds produced at least one fledgling. While experienced birds can have on average three chicks per pair, a population at carrying capacity has an average of 0.5 chicks per pair.
- *There is no detrimental behavioural patterns perceived to be caused by the transmitter, eg more time being spent on the ground* – there appeared to be no differences in behaviour between transmittered and non-transmittered birds; in particular, the transmittered birds did not spend more time on the ground
- *There are no injuries caused by the backpack transmitter system* – there were no injuries to the transmittered saddleback.

We assessed that the backpack transmitter designed by KiwiTrack are suitable for use on saddleback after release into BSMI and will not impact on survival or breeding success. There is a risk that some transmitters may fail prior to the estimated 10 month battery life, however, this risk is possible with all types of transmitters, and is not deemed to be higher than usual.

As the reintroduction is considered high risk, it is important that the predator activity at consistently low levels. The increase in rat tracking indices suggest that the rat control is not yet at a sufficient level to maintain low numbers of rats during years of high abundance.

4.4.6 Recommendations

- Proceed with planning the second saddleback transfer in April 2007, utilising the lesson learnt from the Cuvier transfer, and subject to rat tracking indices being reduced to acceptable levels (≤5%).

Acknowledgements:
Many thanks to Sarah King, Tamsin Ward-Smith, Bridget Evans, Travis Cullen, Hans Rook and Isabel Castro for assisting with the transmitter trial; and to Craig Gillies and Rhys Burns for assisting with the decision to delay the transfer to 2007.

5 Public awareness and community participation

5.1 Public awareness

5.1.1 Summary

Boundary Stream Mainland Island (BSMI) represents a focal point for advocating the Department's work and providing opportunities for the community to appreciate and participate in conservation on a local and national scale. The Project's approach to public awareness and community participation encompasses a wide range of activities. Staff changes near the end of this reporting period without crossover time may mean not all events/participation may be recorded. The species projects continue to provide a focal point for public interaction.
Three presentations and 11 guided walks were given during this period with the Tumanako Loop track continuing to be a popular walk with the public. Seventeen volunteers participated in the work programme, contributing 195 days. Progress on the Project continues to be documented in newsletters and media releases.

5.1.2 Objectives

Short-term objectives

- To organise events that promote and provide educational conservation opportunities for Schools, Polytechnics, Universities and interest groups.
- To inform the community of the BSMI Project and its progress through newsletters, brochures, and on-site interpretation.
- To inform national and local media of opportunities to cover Project events and developments.
- To develop a public awareness strategy for the BSMI Project.

Long-term objectives

- To maintain good relationships with local Iwi and, as appropriate, involve them in the BSMI Project.
- To foster good relationships with adjacent landowners/managers and keep them informed of the Project's activities and advances.
- To plan and provide for tracks, recreational facilities and inspiring interpretation that will enable visitors to experience and fully appreciate BSMI.

- To increase public awareness, participation and support of BSMI.

5.1.3 Methods

The methods used to promote public awareness and encourage community participation continue as stated in the BSMI 2000-2001 Annual Report.
A review of the Teacher Resource Kit was completed by Tamsin Ward-Smith, Catherine Tiffen, and Amelia. The kit was then trialled by two schools.

Public awareness of the BSMI Project continues to be promoted through staff/public interaction, inclusion of volunteers in duties, printed material, publications, and media coverage. Assessment of the success of these initiatives is quantified indirectly through the number of media releases and articles produced, the number of visiting groups to BSMI, and feedback received on these and the BSMI Project in general.

5.1.4 Results

During this period there have been numerous events at BSMI that have involved Iwi, local landowners and interested community groups. The events that have taken place during this period are outlined below. Additionally, ongoing regular contact and information sharing with landowners is always a high priority for the Project and often occurs as day-to-day meetings while working around BSMI. These meetings provide a relaxed opportunity for landowners to keep in touch with staff and keep up-to-date with the Project's progress. Maintaining good relationships and regular communication with Iwi and the local community is also a high priority for the Project, and the success achieved so far is reflected in the endorsement of all aspects of the Project and participation in events.

- ***Biodiversity***

Members of the public continue to be involved with checking on the released kiwi to monitor health and change transmitter bands. BSMI has also actively supported the "*Save Our Kiwi Hawke's Bay*" community project.
Consultation on the proposed second saddleback translocation continued although this translocation was cancelled due to high rat tracking indices.

- ***Conservation volunteer programme***

Volunteers continue to play a crucial role in Boundary Stream Mainland Island, although the release of the kokako has meant a crop off of regular local volunteer involvement.
Seventeen volunteers providing approximately 195 volunteer days.
Volunteers included students, international volunteers, and members of the local community. Opportunities to volunteer at BSMI continue to be advertised through the

Conservation Volunteers program on the DOC website, staff visits to Universities, and circulated emails to Polytechnics and Universities.

- ***Presentations***

Five presentations were given during this period to a minimum of 41 people (no numbers are available for three of the presentations given). In addition to this seven ‘events’ werer hosted involving a minimum of 114 people (no numbers were avaialbe for four events). Events ranged from a hui with Ngati Pahauwera to discuss saddleback/tieke translocations to hosting two ‘walk and talk’ events for the summer programme organised by the Department of Conservation in conjunction with Hawke’s Bay Regional Council and Sport Hawke’s Bay.

- ***Guided walks***

Fourteen guided walks were given to a minimum of 239 people (numbers were not available for one walk). The Tumanako continued to be the focus of these walks with the interpretation panels providing a good overview of the project. Most guided walks occurred over the busy spring and summer period (n=9).

- ***Written Material***

In March 2006, a BSMI newsletter was produced and mailed to volunteers, iwi, and posted on the internet updating the progress on species monitoring and pest control.

5.1.5 Discussion

All members of the BSMI team have continued to be involved in promoting the BSMI Project. This has taken the form of regular communication with landowners and Iwi, providing opportunities for groups to visit BSMI and participate in the Project as volunteers, informing local newspapers on upcoming events, public speaking, media releases and circulation of newsletters.

Feedback on newspaper articles, public presentations and guided walks has been positive. The kokako, saddleback, and kiwi reintroduction projects continue to give the public a focal point for their visit. Follow-up progress newsletters on these projects to schools and interest groups have helped to maintain interest in the Project and foster ownership within the community.

Volunteer contributions to the Project have again been enormous and have also provided opportunities for students to obtain valuable skills. Volunteers are becoming more involved in the day-to-day management of BSMI, particularly through checking trap lines and other activities. Every effort should be made to continue to advertise

BSMI volunteer opportunities both nationally (i.e. Universities) and internationally (i.e. web-site).

Iwi and landowner support has been instrumental in the recent progress achieved with the Project. Without their help and support, the Project would not be the success it is today.

5.1.6 Recommendations

- Involve schools and interest groups in future kiwi releases and other events of interest.

- Maintain and foster relationships with volunteers, locally, nationally, and internationally.

- Maintain follow up contact with schools and interest groups on the progress of the kiwi and kokako reintroduction projects.

- Produce an informative BSMI newsletter once a year and circulate to all interest groups.

- Develop a public awareness strategy for the BSMI project.

5.2 Research

5.2.1 Summary

Boundary Stream Mainland Island (BSMI) supported one new university study. This was a partial requirement of a Bachelor of Environmental Science degree (internship) *Kiwi conservation and tourism: lessons from the New Zealand Department of Conservation.*
Two research projects and one field trial continued from previous years - the PhD project "*Prevalence of Feline Immunodeficiency Virus in domestic and feral cats*", the M.Sc. "*Inbreeding depression: It's role in the risk of parasitic and microbiogical infections in the endangered saddleback*", and the field trial on the efficacy of salted rabbit as a lure for mustelid traps.
Boundary Stream Mainland Island contributed to two national kiwi research initiatives; a project undertaken by Dr. Karen Nutt, University of Waikato, who is researching the genetic variation of kiwi using microsatellite markers, and a trial of newly developed kiwi Egg Timer transmitters which should reduce the disturbance at the nest while maintaining monitoring outcomes. These studies have yet to be concluded.
The Bachelor of Science (Honours) project (*Using artificial nests to assess predation pressure in forest fragments*) was concluded. Efforts to retrieve information from the incomplete masters *Habitat use of translocated North Island Saddleback* were unsuccessful.
Two Department of Conservation Research and Development Series publications came out of research at BSMI this reporting period, one by Boundary Stream staff (Sullivan, W. 2006 *Mortality and dispersion of saddlebacks after reintroduction to Boundary Stream Mainland Island)*, and the other by Landcare Research (Jones, C and Toft, R. 2006: *Impacts of mice and hedgehogs on native forest invertebrates: a pilot study*). A third publication which was in press at the time of the last report was subsequently rejected due to changing criteria for Research and Development Series publications.

5.2.2 Objectives

Short-term objectives

- To provide project ideas and resources to students and other research organisations.
- To maintain and develop existing contacts with research institutions for the purpose of developing research initiatives.

Long-term objectives

- To promote 'research-by-management' with project specific and broad-scale applications.

- To encourage and support research initiatives through developing awareness of the BSMI restoration project.

5.2.3 Research initiatives

Kiwi Conservation and Tourism: Lessons from the New Zealand Department of Conservation
Arista Hickman

Abstract : Conservation practices and tourist appeal began early in New Zealand's history. In the two decades of its existence the New Zealand Department of Conservation has maintained and improved upon conservation and recreational tourism. The establishment of mainland islands in 1995-1996 marked the beginning of a new conservation effort. Through the triple bottom line of economic sustainability, social equity, and environmental protection, the Department of Conservation successfully manages conservation and tourism. As a showcase area for DOC, Boundary Stream Mainland Island also implements the triple bottom line through species and ecosystem conservation, community involvement, and economic stability. Other conservation and tourism programmes around the world can learn from the effective species preservation techniques and community relations and the failed species reintroductions of the Department of Conservation and specifically Boundary Stream Mainland Island. These lessons show the importance of indigenous inclusion, the benefits of educating the youth and communities, how community ownership of conservation projects and areas ensures their success, the advantages of community stake hold in ecotourism, and the need for frequent change and improvement in the field of conservation.

Prevalence of Feline Immunodeficiency Virus in domestic and feral cats. Jessica Hayes, University of Auckland; PhD. In progress.

Abstract: The aim of this research is to study the epidemiology of Feline Immunodeficiency Virus (FIV) in New Zealand. This involves determining the prevalence of FIV in the domestic and feral cat subpopulations, and the extent of FIV exchange between these subpopulations. The FIV genetic subtypes in New Zealand will also be established, along with whether there is a geographical influence on the genetic diversity of FIV. This project was discussed in the BSMI 2003-04 Annual Report. Further cat specimens (60) were supplied to Jessica over the course of the year. Due to staff turnover it is not known what the outcome of this research was.

Using artificial nests for assess predation pressure in forest fragments. Rebecca Lewis, Massey University; BSc (Honours).
This work, as discussed in the BSMI 2002/2003 Annual Report, has now been completed. Due to staff turnover results cannot be discussed as we have been unable to find them.

Inbreeding depression: It's role in the risk of parasitic and microbiogical infections in the endangered saddleback (*Philesturnus carunculatus*). Katrina Hale, School of Biological Sciences, University of Canterbury.

The New Zealand saddleback provides a model system to answer questions about bottleneck size and susceptibility of birds to parasites and pathogens. This species is ideal for such a study because it survives as a large number of island populations that were founded by differing number of individuals, enabling an understanding of whether populations that passed through more severe bottlenecks have higher parasite loads, reduced immune response, etc., than populations that are less inbred. Other native species such as the New Zealand robin and the bellbird are also being used to carry out between-species comparisons. The results of this project will provide direct and tangible benefits to the conservation of endangered species in New Zealand and around the world.

The New Zealand saddleback provides a model system to answer questions about bottleneck size and susceptibility of birds to parasites and pathogens. This species is ideal for such a study because it survives as a large number of island populations that were founded by differing number of individuals, enabling an understanding of whether populations that passed through more severe bottlenecks have higher parasite loads, reduced immune response, etc., than populations that are less inbred. Other native species such as the New Zealand robin and the bellbird are also being used to carry out between-species comparisons. The results of this project will provide direct and tangible benefits to the conservation of endangered species in New Zealand and around the world.

The use of small numbers of birds to start new populations is a common practice among conservation biologists but it may inadvertently increase the risk of inbreeding and disease susceptibility. Exactly how many individuals are required to found a new population and avoid the negative consequences is unknown. Such guidelines are vital if conservationists are to avoid subjecting endangered species to increased inbreeding depression. Such areas of study are vital to gaining an understanding of the risks of inbreeding depressions, parasitic infection and wildlife disease in New Zealand's unique avifauna. By understanding the link between inbreeding and risk of parasitic and microbiological infection, this research will provide better guidelines for the establishment and management of island populations that aim to minimise these risks.

The field work for this research included sampling the 40 saddleback transferred to BSMI in September 2004. The full write-up will be completed by the end of August 2006.

Kiwi genetic diversity study

Boundary Stream has contributed to a national project undertaken by Dr. Karen Nutt, University of Waikato, who is researching the genetic variation of kiwi using microsatellite markers. Of particular interest is understanding more about kiwi behaviour and if there is any evidence of extra-pair couplings amongst kiwi. This same genetic information may also contribute to a better understanding of the remaining genetic variation within Eastern North Island Brown Kiwi taxon.

Kiwi Egg Timer transmitter trial

The Egg Timer transmitter is a specially designed transmitter for adult male North Island brown kiwi with special functions which detect and report incubation, allowing

a much more accurate (and labour reduced) hatch date estimate. The transmitter and software were developed by Wildtech Ltd and KiwiTrack Ltd, under contract to Department of Conservation. A total of one hundred and fifty Egg Timer transmitters are going to be trialled through the 2006-07 breeding season by;

- Coromandel, Tongariro and Northland Kiwi Sanctuaries
- Taranaki Kiwi Trust
- The Whirinaki and Ohope kiwi projects
- Boundary Stream Mainland Island and Kaweka Forest Kiwi Project.

At Boundary Stream, three known breeding male kiwi will be fitted with Egg Timer as part of this national trial. The report on the trial will be completed by a contractor at the end of the 2006-07 season.

Saddleback backpack transmitter and harness trial

For the first transfer of saddleback to Boundary Stream, tail-mounted transmitters were used on saddleback. Due to their short battery life and versatility, essential information such as survival and breeding success was not obtained. Consequently, backpack transmitter was developed by Kiwitrack Ltd, with harness provided by Sirtrack Ltd (see details on 4.4 Saddleback section). As backpack transmitters had not been used on saddleback previously, the transmitters were trialled on six birds on Mokoia Island from September 2005 to January 2006. While only broad behavioural patterns were observed in this study in a small sample size, transmitters did not appear to impact on survival or detrimentally alter behaviour. Therefore, backpack transmitter may be recommended for post-release monitoring of future transfer of saddleback.

Salted rabbit trial

The egg and salted rabbit meat bait trial that began in December 2004 was continued throughout this period on the perimeter trap line (208 double set Mark IV traps). Odd numbered traps were baited egg only, with even numbered traps baited salted rabbit only. All perimeter traps were checked monthly. The trial will be reviewed in January 2007.

Publications

Two Department of Conservation Research and Development Series publications came out of research at Boundary Stream Mainland Island this reporting period, one by Boundary Stream staff (Sullivan, W. 2006 *Mortality and dispersion of saddlebacks after reintroduction to Boundary Stream Mainland Island),* and the other by Landcare Research (Jones, C and Toft, R. 2006: *Impacts of mice and hedgehogs on native forest invertebrates: a pilot study*). Both of these were reported on in the 2004-05 report. A third publication which was in press at the time of the last report was subsequently rejected due to changing criteria for Research and Development Series publications.

5.2.4 Discussion

Student studies at BSMI continue to assist in researching aspects of ecology at both a local and national level. These studies can potentially assist in making management decisions, and allow research to be conducted at a higher level than time and resource restrictions allow of BSMI staff. However, significant amount of time and resources often needs to be employed into each student, and in return, BSMI should expect high quality, useful data to be collected as well as written reports. Managers need to ensure that if the student pulls out of the study, full access to all data and ideas is prearranged and given. Attracting students to BSMI can be difficult, as with the high cost of tertiary education, student need projects with funding. To attract high quality students which produce valuable data and written reports, BSMI may need to offer substantial funding.

Being an active part of National trials is an important aspect of the BSMI Project. BSMI needs to continue to make the Project available for such trials.

5.2.5 Recommendations

- Continue to provide potential research topics to Universities at the start of each year.
- Investigate sources of funding for students.
- Seek input from universities into management of Boundary Stream Mainland Island where appropriate with the view to strengthening relationships and providing impetus for student involvement

6.0 Acknowledgements

Many thanks to everyone who assisted BSMI staff in the various projects at Boundary Stream Mainland Island with hard labour, technical skills and advice.

Many thanks to all those who assisted with the planning of second saddleback transfer and saddleback harness trial on Mokoia Island, in particular: local iwi: Ngati Pahauwera, Ngati Tu and Ngati Hineuru and Mokoia Island Trust Board; Hill Country Corporation, Empire World Trade and Hastings/Havelock North Forest and Bird (sponsors from the first transfer) for continuous support; Katrina Hale, Richard Jacob-Hoff, Kate McInnes and Stephanie Shaw for advice on disease screening; Dr Isabel Castro, Dr Doug Armstrong, Tim Lovegrove, Brent Stephenson John Adams for advice; Angela Charry and Hans Rook for monitoring saddleback on Mokoia Island for the harness trial; Sarah King for training BSMI staff with attaching harness and assisting saddleback capture for the harness trial; Brett Butland (ECHB Conservancy) for investigating potential sponsorship.

Many thanks to Craig Gillies, Brent Stephenson, Wayne Hutchinson, Elaine Wright, Kokako Recovery Group and Darren Peters for technical skills and advice throughout the year.

Much appreciation goes to a variety of local and international volunteers as well as contractors, DoC staff from other areas and university lecturers for their significant input and assistance to the BSMI project. In particular, Dave Barnard and Mary Gray who regularly assisted with trapping and kiwi monitoring; Mary Marshall, Teresa Hurst, Alastair Moffit, Rose Dawton, Ken Drury, Ian Flux (RD&I) and Dave Para who helped kokako survey, nest monitoring and chick banding; Ariana Longanecker, Ben Paris, Tom Studholme (Mt Bruce) and Sarah Kivi for assisting species monitoring and pest control; Doug Armstrong for advice on distance sampling; Rogan Colbourne and Hugh Robertson (RD&I) for advice on kiwi management; Tertia Thurley and Stacy Hill for training BS staff with kokako mist-netting and transfer; Rose Dawton and Ken Drury for assisting snail survey; John Winters for assisting robin survey; Brendon Christenson, Ian Stringer, Chris Green, Alison Evans and Ian Westbrook for assistance with invertebrate data analysis and monitoring; Howard Mathews (Maniapoto FC) and Phil Bradfield (Te Kuiti AO) for advice on racumin.

Many thanks to Ngati Pahauwera, Ngati Tu, Ngati Hineuru and Waimana Kaaku, DoC Opotiki staff (ie. Kevin Cannel, Andrew Glaser, Jane Haxton, Cody Thyne), Dave Para, Rhys Burns, Ken Hunt, John Adams for continuous support for kokako reintroduction project

Many thanks to Alan Lee, Hans Rook and Harry Verwey (Napier Area Office) with various maintenance jobs at the Reserve.

The work at BSMI could not progress without access across the private properties of the local landowners: Tom and Annabel Lo, Sue and Graeme Maxwell, James and

Tamela Brownrigg, Kerry Phillips and Marcus Worsnap, Murray Heays, Eric Sanderson and Peter Proctor.

7.0 References

Boundary Stream Mainland Island 1996-1998 Project Report. 2000. Department of Conservation, East Coast/Hawkes Bay Conservancy.

Boundary Stream Mainland Island Project Report 1998-2000. 2003. Department of Conservation, East Coast/Hawkes Bay Conservancy.

Boundary Stream Mainland Island Annual Report 2000-2001. 2003. Department of Conservation, East Coast/Hawkes Bay Conservancy.

Boundary Stream Mainland Island Annual Report 2001-2002. 2003. Department of Conservation, East Coast/Hawkes Bay Conservancy.

Boundary Stream Mainland Island Annual Report 2002/2003. 2004. Department of Conservation, East Coast/Hawkes Bay Conservancy.

Boundary Stream Mainland Island Annual Report 2003/2004. 2005. Department of Conservation, East Coast/Hawkes Bay Conservancy.

Boundary Stream Mainland Island Annual Report 2004/2005. 2005. Department of Conservation, East Coast/Hawkes Bay Conservancy.

Cuthbert, R. 2003. Sign left by introduced and native predators feeding on Hutton's shearwaters *Puffinus huttoni*. New Zealand Journal of Zoology. Vol. 30. p. 163-170.

Flux, I. and Innes, J. 2003. Kokako management folder. *Threatened Species Occasional Publication* 19. Department of Conservation, Wellington. 74pgs.

Jones, C and Toft, R. 2006. Impacts of mice and hedgehogs on native forest invertebrates: a pilot study. *Department of Conservation Research and Development Series 245*, Wellington. 32 p.

Lond. 1971. Notes from the Mammal Society – No. 22. *Journal of Zoology*. 164. p 239-270.

Lovegrove, T. 1991. Saddleback transfers from Stanley Island to Kapiti Island 1987-1989. *Science and Research Internal Report* No. 98. Department of Conservation, Wellington. 33 pgs.

Lyver, P. O'B. 2000. Identifying mammalian predators from bite marks: a tool for focusing wildlife protection. *Mammal Review*. Vol 30. No. 1. p. 31-44.

McCaw, J. Unpublished report. Ecology and Entomology Group, Soils, Plant and Ecological Science Division, Lincoln University, New Zealand.

Ratz, H. and Moller, H. 1997. Identification of predators of Royal albatross chicks at Taiaroa Head in February 1994. *Conservation advisory science notes*, Department. of Conservation, Wellington. 138 pgs

Ratz, H., Moller, H. and Fletcher, D. 1999. Predator identification from bite marks on penguin and albatross chicks. Marine Ornithology. Vol 27. p. 149-156.

Robertson, H.A. 2003. Kiwi (Apteryx spp.) recovery plan 1996-2006. *Threatened Species recovery plan 50*. Department. of Conservation, Wellington. 26 p.

Robertson, H.A. and Colbourne, R. 2003. *Kiwi (Apteryx spp.) Best Practice Manua*l. Internal Publication, Department of Conservation, Wellington. 101 pgs.

Thomas, M. 1999. Feasibility of using waxblocks to measure rodent and possum abundance and changes in population size. In *Progress in mammal pest control on New Zealand conservation lands*. Science for conservation 127 Department of Conservation, Wellington,79 pgs.

Sullivan, W. 2004. Proposal to translocate saddleback from Cuvier Island to Boundary Stream Scenic Reserve. *Internal Publication, Department of Conservation*, East Coast/Hawke's Bay Conservancy.

Sullivan,W. *Saddleback reintroduction to Boundary Stream Mainland Island: differences in initial mortality and dispersal patterns between adults and juveniles. September to November, 2004*. Department of Conservation Research and Development Series, Wellington.
