# Supplementary material for: Responses of New Zealand forest birds to management of introduced mammals
Source: Conserv Biol. 2020 Mar 23;35(1):35–49. doi: 10.1111/cobi.13456 (PMC7984369; doi:10.1111/cobi.13456)

Rotoiti Nature Recovery Project Annual Report 2010-11

Nelson Lakes Mainland Island, Nelson Lakes National Park

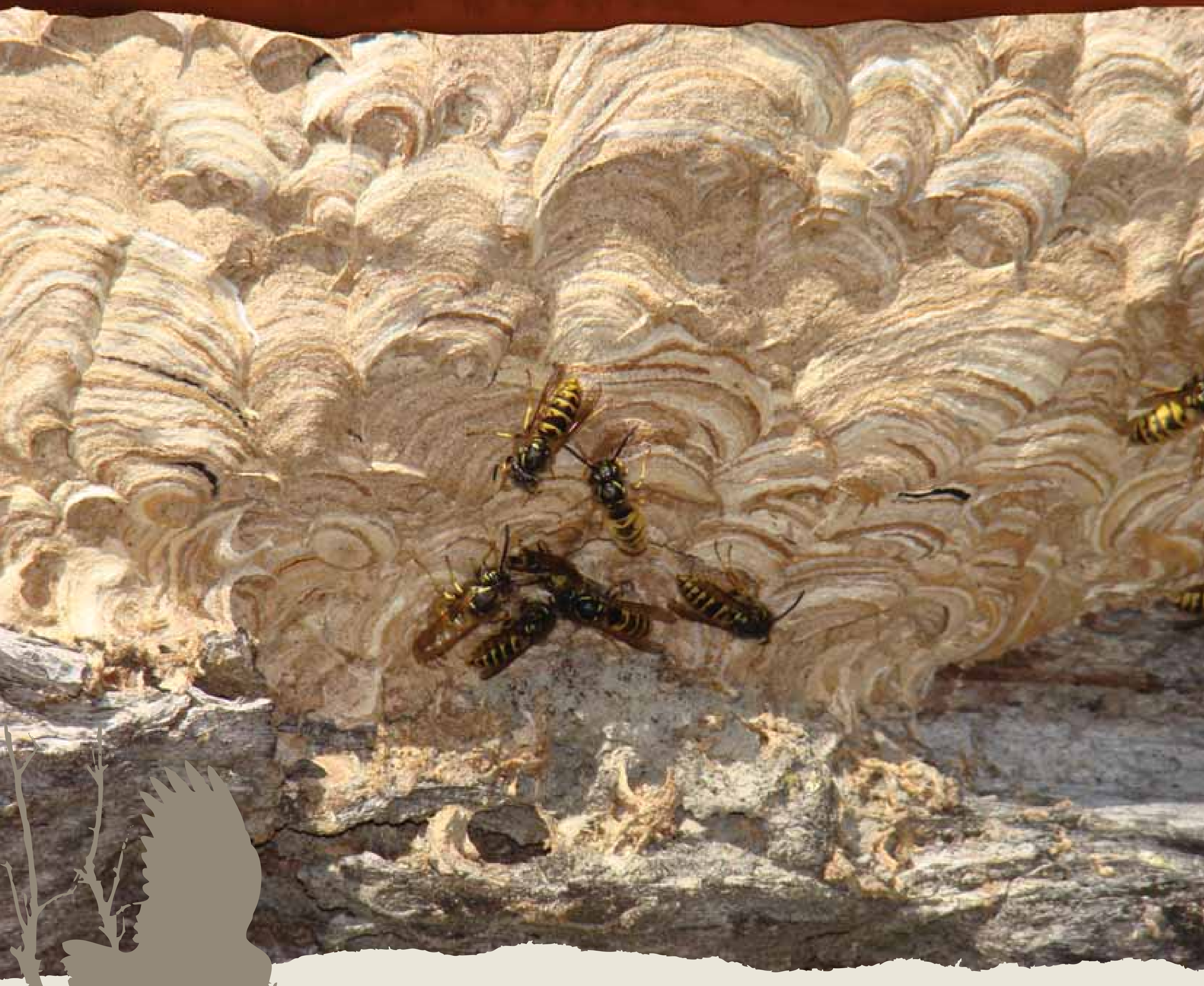

Department of Conservation
Te Papa Atawhai

newzealand.govt.nz

Rotoiti Nature Recovery Project Annual Report 2010-11

G. Harper, S. Forder, J. Henderson, N. Joice, P. Carter,
D. Chisnall, A. Doura, D. Rees

Cover image: Common wasps (*Vespula vulgaris*) on a nest in the Rotoiti Mainland Island. These introduced wasps reach their highest recorded densities in the world in the honeydew forest on the northern South Island.

© Copyright, New Zealand Department of Conservation

Occasional Publication No. 90
ISSN 0113-3853 (print), 1178-4113 (online)
ISBN 978-0-478-14938-8 (print), 978-0-478-14939-5 (online)

Published by: Department of Conservation, Nelson/Marlborough Conservancy, Private Bag 5, Nelson.

In the interest of forest conservation, we support paperless electronic publishing.

CONTENTS

Executive summary 1

1. Introduction 4

2. Biodiversity restoration objectives 6

2.1 Restore and maintain populations of kaka, mistletoe, *Pittosporum patulum* and *Powelliphanta* sp. 6
2.1.1 Introduction 6
2.1.2 Mustelid (stoat, ferret and weasel) control and monitoring 7
2.1.3 Feral cat control and monitoring 19
2.1.4 Possum control and monitoring 21
2.1.5 Deer control and monitoring 23
2.1.6 Kaka (*Nestor meridionalis*) monitoring 23
2.1.7 Kea (*Nestor notabilis*) nest protection 25
2.1.8 Weka (*Gallirallus australis*) monitoring 25
2.1.9 Mistletoe (*Alepis* and *Peraxilla*) monitoring 27
2.1.10 *Pittosporum patulum* monitoring 27
2.1.11 *Powelliphanta* sp. monitoring 27

2.2 Establish and maintain populations of great spotted kiwi and other native species 29
2.2.1 Introduction 29
2.2.2 Great spotted kiwi (*Apteryx haastii*) population management 29
2.2.3 Great spotted kiwi (*Apteryx haastii*) population monitoring 30

3. Learning objectives 33

3.1 Test the effectiveness of rodent control tools in a beech forest system 33
3.1.1 Introduction 33
3.1.2 Ship rat (*Rattus rattus*) control 33
3.1.3 Rodent population monitoring 35
3.1.4 South Island robin (*Petroica australis australis*) monitoring 37

3.2 Test the effectiveness of wasp control tools 40
3.2.1 Introduction 40
3.2.2 Wasp control and monitoring 40

3.3 Test the effectiveness of different translocation methods 43
3.3.1 Introduction 43

3.4 Determine long-term trends in bird abundance and forest health in response to ongoing management 45
3.4.1 Introduction 45
3.4.2 Five-minute bird counts 45
3.4.3 Vegetation plot monitoring 46
3.4.4 Beech seed fall monitoring 46
3.4.5 Tussock plot monitoring 47

3.5 Systematically record observations of previously unreported native and non-native organisms in RNRP 48
3.5.1 Introduction 48

3.6 Facilitate research to improve our understanding of the ecology and management of beech forest and alpine systems 48
3.6.1 Introduction 48
3.6.2 Research conducted during 2011 48
3.7 Analyse and report on the effectiveness of management techniques and ensure that knowledge gained is transferred to the appropriate audiences to maximise conservation gain. 49
3.7.1 Introduction 49
3.7.2 Reports generated during 2010/11 49
3.7.3 Hui, workshops, presentations and media articles 49

4. Community objectives 50

4.1 Increase public knowledge, understanding and support for mainland islands and ecological restoration nationally through education, experience and participation 50
4.1.1 Introduction 50
4.1.2 Friends of Rotoiti 50
4.1.3 Volunteers 52
4.1.4 Advocacy and education 52

5. Discussion 54

6. Recommendations 55

7. Acknowledgements 56

8. References cited 57

Appendix 1: RNRP datasets 58

Appendix 2: RNRP Reports generated 59

Appendix 3: Project reviews 60

Appendix 4: Research reports received 61

Appendix 5: Project management 62

Appendix 6: Bird count graphs 63

Executive summary

BIODIVERSITY RESTORATION OBJECTIVES

Restore and maintain populations of kaka, mistletoe, *Pittosporum patulum* and *Powelliphanta* sp.

Mustelid control continued in the Mainland Island during 2010-11, and monitoring indicated that the tracking tunnel target of <5% was exceeded once (8%) over the summer in the treatment area, while mustelid abundance remained high at the Rotoroa non-treatment site.

The upgrade of the current DOC200s to stainless steel continued as funding allowed.

Increased possum and cat control continued this season. The kaka encounter rate appears to be providing comparable abundance data between years, although breeding success or recruitment is not measured.

Monitoring of beech mistletoes and *Pittosporum patulum* was not required this year. *Powelliphanta* "Nelson Lakes" snail monitoring was not undertaken this season.

Establish and maintain populations of great spotted kiwi and other native species

Only 11 of 22 great spotted kiwi in the Mainland Island are currently radio-tagged due to failed or dropped transmitters and of these four are wild-raised chicks. One radio-tagged adult was killed by a predator, probably a dog. The Rotoiti Nature Recovery Project (RNRP) kiwi dog, 'Fen' proved to be very useful in finding several missing kiwi.

LEARNING OBJECTIVES

Test the effectiveness of rodent control tools in a beech forest system

There was no beech mast in autumn 2011. Tracking tunnel monitoring indicated a decrease in rodent abundance through 2010-11. A rat control operation using RatAbate™ paste (diphacinone) was carried out over 600ha and reduced rat tracking rates from 50% to <4% within six weeks. South Island robin nesting success was used as an outcome measure for rat control and nesting success was 83% (five nests fledged young from six attempts). The robin monitoring showed a small increase in robin numbers within the standard survey area in 2010.

Test the effectiveness of wasp control tools

The wasp bait X-stingish™ was used again under the experimental use arrangement with the Nelson based company, Entecol. The wasp population in the RNRP core area, Big Bush and around the St Arnaud village was controlled to low levels, although initial wasp densities were already considered to be quite low. The wider spacing between bait station lines in the core and the two experimental poison nodes outside the control area were again retested this season. Preliminary results indicate that wasp control could be effective up to 150m from poison bait stations.

Test the effectiveness of different translocation methods

Funding for an Operation Nest Egg (ONE) project was secured from the Bank of New Zealand Save the Kiwi Trust for the 2010-11 year. Three eggs were transferred to Willowbank from Gouland Downs. One chick died shortly after hatching. The two remaining chicks were released in autumn, but both died from misadventure. Two chicks from the previous year's release are alive in the Mainland Island.

Determine long-term trends in bird abundance and forest health in response to ongoing management

Five-minute bird counts were undertaken at Lakehead, on the St Arnaud Range track; and at the Rotoroa non-treatment site.

An alpine tussock seed fall transect was re-measured at Mt Misery in early 2011. No seed was noted.

Systematically record observations of previously unreported native and non-native organisms in RNRP

A system for recording new species was established in 2010. No new species were noted in 2011.

Facilitate research to improve our understanding of the ecology and management of beech forest and alpine systems

A student research paper using the five-minute bird count data from Rotoiti and Rotoroa and investigating responses of birds to pest control was produced (Langham 2010). A survey for bats was also repeated within the Mainland Island.

Analyse and report on the effectiveness of management techniques and ensure that knowledge gained is transferred to the appropriate audiences to maximise conservation gain

The 2009-10 report was published in November 2010 (Harper et al. 2010). A student research paper using the five-minute bird count data from Rotoiti and Rotoroa and investigating responses of birds to pest control was produced (Langham 2010). Staff also participated in two great spotted kiwi hui.

COMMUNITY OBJECTIVES

Increase public knowledge, understanding and support for mainland islands and ecological restoration nationally through education, experience and participation.

Friends of Rotoiti (FOR), the local conservation volunteer group formed in 2001, continued their mustelid, rodent, possum and wasp control, to support and enhance the work being done within the mainland island. This year, FOR contributed 248 workday equivalents. They carried out mustelid trapping over more than 5000ha of land adjacent to the RNRP and rodent trapping over 250ha in the St Arnaud village.

In addition, another 14 volunteers assisted the mainland island, with two general RNRP volunteers (one international, one New Zealander) and 12 trainee rangers, working the equivalent of 49 days.

Two editions of the *Revive Rotoiti* newsletter were published during the year, focussing on advocacy and highlighting the work being done in the RNRP. New interpretive displays were opened in September 2010 in the Nelson Lakes National Park Visitor Centre. The previous RNRP area has been replaced by new backlit displays and flip cards which can be updated as the project develops.

The FOR supporter group continues to grow, providing another avenue for advocacy and education of the mainland island story.

The RNRP PowerPoint show continues to be updated and was presented 20 times to 770 students staying at Rotoiti Lodge. Another six visiting groups to Nelson Lakes also received this PowerPoint presentation, with 143 participants. This year continued the change in preference to walks, with 680 students and visitors undertaking 45 guided RNRP walks. This year provided 71 opportunities for RNRP advocacy, with 1593 participants.

Work on improving robin nesting success and gathering information on weka was conducted by the RNRP with a view to increasing the numbers of these species, as they freely interact with humans and enhance visitors' experience of the Mainland Island.

1. Introduction

The Rotoiti Nature Recovery Project (RNRP) is a 'Mainland Island' project established in 1996 to enable the recovery of a representative portion of an alpine honeydew beech (*Nothofagus*) forest ecosystem at Lake Rotoiti in Nelson Lakes National Park.

The project began with infrastructure development and baseline monitoring across 825 ha of forest on the western St Arnaud Range. Comprehensive pest control began in 1997. The project was established with control/treatment sites so responses to management techniques at Lake Rotoiti could be compared with the control (non-treatment) site at nearby Lake Rotoroa. The first annual report covered the 1997-1998 business year.

South Island kaka (*Nestor meridonalis meridonalis*) were a key focus from the beginning of the project. DOC Science and Research Unit staff put considerable effort into radio-tracking kaka and monitoring nesting success in response to mustelid control. Kaka nesting success improved considerably and adult female mortality declined as a result of predator control when compared with non-treatment sites (Moorhouse et al. 2003).

In 2001-02 the extent of mustelid trapping was increased considerably and now over 5000ha on the western St Arnaud Range and southern Big Bush is under sustained predator control as part of the Mainland Island. Trapping is also carried out by a local volunteer group, Friends of Rotoiti (FOR) in adjacent areas, encompassing some additional 5000ha.

In addition to kaka, management of great spotted kiwi (*Apteryx haastii*) began in 2004 with the introduction of adult individuals from Gouland Downs in Kahurangi National Park. Additional introductions have ensured the successful establishment of a population. Some limited breeding has taken place over the past seven years, and five wild-raised kiwi chicks have survived, despite being known to be vulnerable to mustelid predation. An Operation Nest Egg (ONE) operation has continued with hand-reared chicks, sourced from eggs removed from adults on the Gouland Downs, being released into the Mainland Island. Two ONE chicks transferred in early 2010 have established successfully in the Mainland Island, but all three chicks hatched this season have died, either at the hand-rearing facility or shortly after transfer to Rotoiti.

The RNRP has been a leader in large-scale control of introduced wasps (*Vespula* spp.) Under an experimental use arrangement, historically with Landcare Research and more recently with the Nelson based company Entecol, the RNRP has been used as a trial site. Experiments have been undertaken with various toxins, X-stinguish™ in particular. Spacing of bait stations was again the focus of this year's research and effective reduction of wasp activity was achieved at much lower bait station density than previously deployed. Further trials of bait station density will continue over the next few years.

Rodent control has had a chequered history in the core area of the Mainland Island. Initial control of rodents, rats in particular, was effective with brodifacoum and 1080 between 1997 and 2000. After a Departmental review of the use of brodifacoum a switch to snap-trapping at a density of one trap/ha proved ineffective at controlling rat populations. The first rat control operation in over four years was run in the spring of 2010, over 600ha of the Core Area of the Mainland Island using diphacinone in bait stations. Rat tracking in the Core Area declined from 50% to <4% within 6 weeks after the start of the poison operation. The continued use of five-minute bird counts and robin (*Petroica australis*) monitoring provides a response measure for rodent control. On the basis of the successful poisoning operation the area under rat control is now being extended to approximately 900ha on the eastern shores of Lake Rotoiti.

Feral cats were targeted in and around the RNRP this season using cage traps, with a large amount of effort put in by trappers. Autumn was found to be the most effective time to cage trap, however a less labour intensive weka-proof cat trap is required. Continued trapping of possums, using Sentinel™ traps, has continued at a high level, targeting areas where fresh sign is found. Other pest species under management include ungulates, pigs and hedgehogs using a mixture of techniques.

Monitoring of native species' responses to pest control include browse-sensitive plants - three species of beech mistletoe, the critically threatened understory plant *Pittosporum patulum*, and *Griselinea littoralis*. Beech seedfall and *Chionochloa* tussock flowering are monitored as 'ecological drivers' of rodent and subsequent mustelid population increases, and 20x20 vegetation plots are monitored to determine the trends and responses of native vegetation to multiple species pest control.

Invertebrate monitoring has included *Powelliphanta* snails, as well as beech scale insects and honeydew production due to their importance as 'ecological drivers' in the honeydew beech forest ecosystem.

In addition to the 'core work' undertaken by RNRP staff, several students conduct research in the Mainland Island each year, which adds to our understanding of the alpine beech forest ecosystem functioning and improves pest control. Some time and money from the RNRP budget is used to support these projects.

The involvement of the local and wider community in the RNRP is essential for the success of the project and there is a strong theme of advocacy and participation. Volunteers have undertaken hundreds of days of work in support of the Project over the past 13 years by Friends of Rotoiti (FOR), trainee rangers, Conservation Corp crews and the Over-50s tramping club. Staff have also given time for other departmental and community initiatives and attended workshops and conferences to transfer knowledge to the wider community. Advocacy has included presentations to many school and community groups, guided walks, displays in the Nelson Lakes Visitor Centre, information panels within the Mainland Island and various printed media. Many events and achievements from the RNRP have also been picked up by local and national media, including the listing as one of the Top 25 Ecological Restoration Sites in Australasia.

Although day to day work on the Mainland Island progresses in response to annual or multi-annual ecosystem cycles, no operation of this scale can operate without a vision and objectives to provide guidance in the medium term. Therefore the publication of the Rotoiti Nature Recovery Project Strategic Plan for 2008-2013 (Brown and Gasson 2008) has provided the planning framework and goals for the operation for the next four to five years and has highlighted the three major themes running through the project, namely;

1. Research, learning and knowledge transfer to a burgeoning number of ecological research projects nationwide.
2. Protecting and restoring biodiversity for its intrinsic value.
3. Advocating the value of ecological restoration to the public.

It is essential these themes remain the core values for ongoing restoration work within the Rotoiti Mainland Island for the future. A Technical Advisory Group and external advisors contribute an essential role in overseeing and guiding these themes.

2. Biodiversity restoration objectives

2.1 RESTORE AND MAINTAIN POPULATIONS OF KAKA, MISTLETOE, *PITTOSPORUM PATULUM* AND *POWELLIPHANTA* SP.

2.1.1 Introduction

The proposed RNRP Strategic Plan 2008-2013 identifies six threatened species will be actively maintained for their biodiversity values. These populations and their New Zealand Threat Classification System rankings are:

- South Island kaka *Nestor meridionalis meridionalis* - Category 2: Nationally endangered;
- the beech mistletoes *Peraxilla colensoi, P. tetrapetala* and *Alepis flavida* - all Category 4: Declining;
- the heteroblastic tree *Pittosporum patulum* - Category 2: Nationally endangered;
- the carnivorous land snail *Powelliphanta* "Nelson Lakes" - Category 7: Range restricted.

The RNRP contains some further threatened species that may benefit from pest control. The above populations were specifically identified in the Strategic Plan 2008-2013 because a considerable amount of work has already been invested into monitoring and managing them through the preceding decade.

The kaka is an endemic forest parrot which is threatened by predation. Stoats (*Mustela erminea*) are the main predator of kaka, but all three introduced mustelids (stoats, ferrets and weasels) are targeted by mustelid control. Mustelid trapping has been shown to protect the local kaka population (Moorhouse et al. 2003), and mustelid control will continue for the foreseeable future. An upgrade from Fenn MkVI traps to DOC 200 and DOC 250 traps commenced in 2007 and was completed in late 2009. Feral cat control, although localised to date, may protect fledging kaka chicks which spend up to three days on the ground between emerging from their nest holes and flying. More intensive cat control project is now in place. Other native bird species are likely to benefit from predator control, particularly great spotted kiwi and New Zealand falcon, which nest on the ground.

The beech mistletoes, *Pittosporum patulum* and snails *Powelliphanta* "Nelson Lakes" are all threatened as a result of predation by the introduced brushtail possum (*Trichosurus vulpecula*). Possum numbers have been reduced and suppressed within the Mainland Island through a sustained poisoning and trapping project. As with mustelid control, possum control is considered to be effective, and will continue for the foreseeable future in order to protect biodiversity values.

In addition to being threatened by possums, *P. patulum* and *Powelliphanta* "Nelson Lakes" populations may be threatened by red deer (*Cervus elaphus scoticus*). Detrimental browsing of juvenile *P. patulum* plants has been attributed to red deer. Red deer may deleteriously impact *Powelliphanta* habitat through concentrated browsing and trampling in the mountain beech/tussock ecotone that is favoured by both deer and *Powelliphanta* "Nelson Lakes". Deer control is currently not part of the RNRP pest control programme, but has been supplemented by the initiation of limited access to the Mainland Island for recreational hunters in May 2010, principally through local NZ Deerstalker branch

members in a volunteer capacity. Hunters are allocated one of four blocks within the area and all animals shot recorded. Another probable problem species for high montane and alpine species are hares (*Lupus europeaus*) that are likely to degrade habitat.

2.1.2 Mustelid (stoat, ferret and weasel) control and monitoring

RNRP mustelid control methods

Mustelids are trapped over approximately 5,000 ha to the east and north of Lake Rotoiti. The goal of this trapping is to suppress stoat tracking below 5%, allowing successful kaka breeding. The 24 traplines have a total of 907 mustelid traps: 815 DOC 200's (441 stainless steel/372 combination) and 92 DOC 250's (Figure 1). During the summer and autumn when stoat numbers are high and juveniles are dispersing, trap lines are checked fortnightly. Intervals between trap checks are extended to up to six weeks over the winter and early spring when few stoats are present.

The spacing between traps remains 100m apart. All traps are single set, baited with hen eggs and enclosed within wooden boxes. We were forced to change over from the 'best practice' white hen eggs to the less favoured brown egg in April due to suppliers terminating production of white eggs. The box design is 'best practice' length for use in weka and kiwi areas.

Figure 1. RNRP and FOR mustelid trap locations

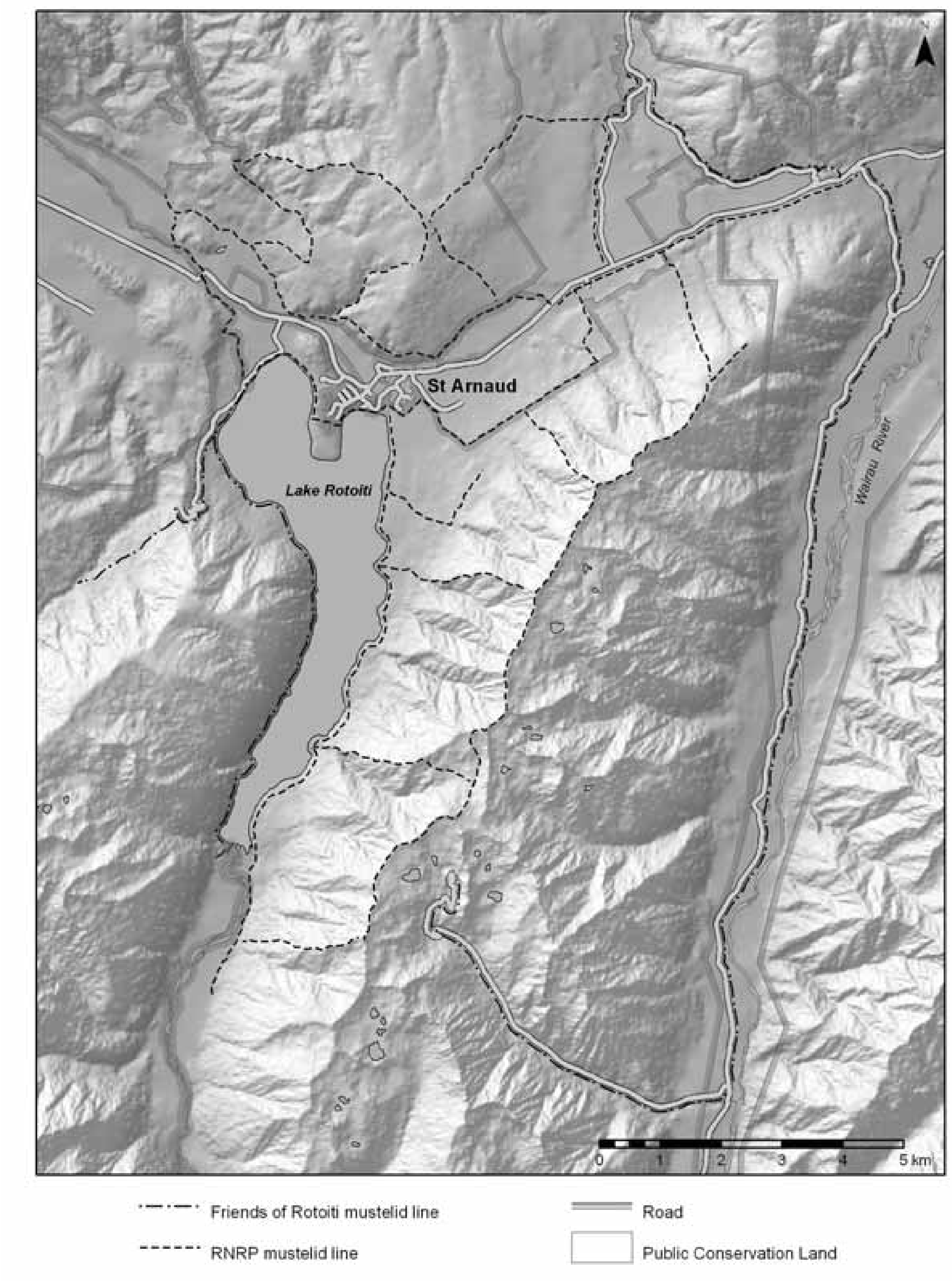

RNRP mustelid control results

This year 164 stoats, 28 weasels and one ferret were captured. The stoat and ferret figures were around the average capture rates for the extended 5000ha mustelid controlled area. However the number of weasels was very high and only exceeded by 32 in 2006-2007. The lighter set-off weight of DOC200's over Mk 6 Fenns is likely to have contributed to the increase in weasel captures.

Figure 2. Percentage captures of stoats in RNRP perimeter and internal traplines over 2010-11

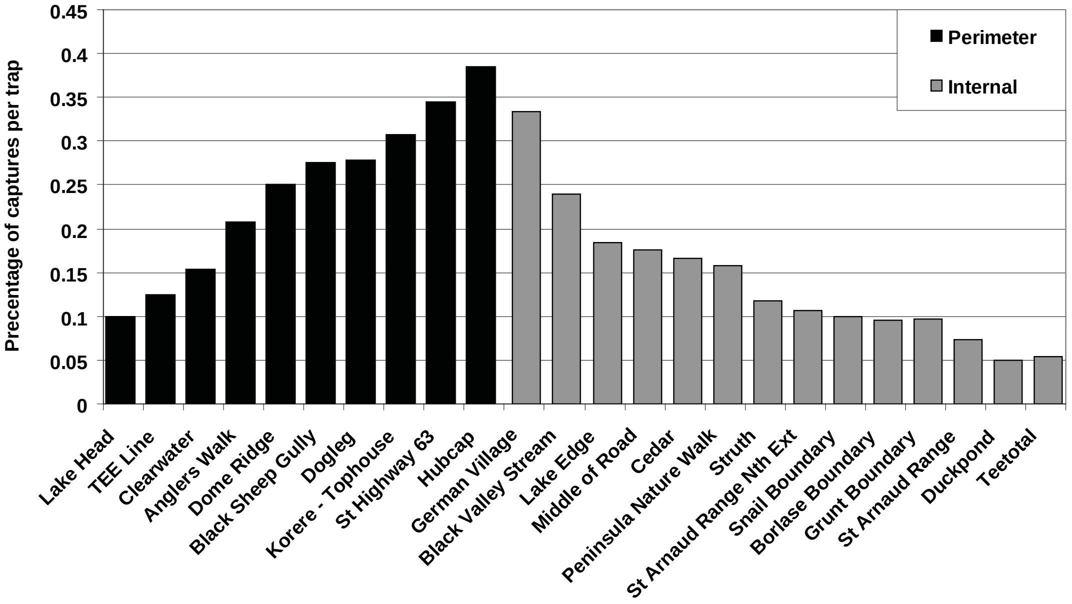

Of the 164 stoats captured for the 2010-11 season slightly more than ½ (88) were caught in the perimeter traps and the remaining 76 within the internal trap lines (Figure 2). The perimeter lines 'Hubcap', 'SH63' and 'Korere-Tophouse' had the highest capture rates. The 'St Arnaud Range' and 'Lakehead' traplines have been included in the internal lines as the FOR trap lines bound these lines. Of the internal lines the 'German Village', 'Black Valley Stream' and 'Lake Edge' lines had the highest captures. This may have been due to the long 'edges' on these lines, either between pasture/tussock and forest or lake edge and forest. 'Black Valley Stream' had high capture rates last year also. The highest capture rates for the internal lines were during the summer and autumn, probably due to reinvasion from outside the trapped area (Figures 5 & 6). Both the internal and external trap lines trapped stoats in all seasons, albeit in low numbers over winter and spring.

Non-target species caught this year were:

- 467 rats
- 160 hedgehogs
- 72 rabbits
- 1 mouse
- 6 cats
- 4 birds

Considerable effort was put into upgrading the marking of the stoat trap lines this year. Flagging tape was replaced with blue plastic triangles on trap lines and yellow triangles at trap locations. Above the bushline orange plastic marker poles were placed at each trap with one in between, unless the trap boxes were on an obvious ridge. It is planned to upgrade the rest of the trap lines in 2011-12 and have scheduled maintenance thereafter.

Figure 3. Map of stoat captures on RNRP trap lines for Jul to Aug 2010 & Jun 2011

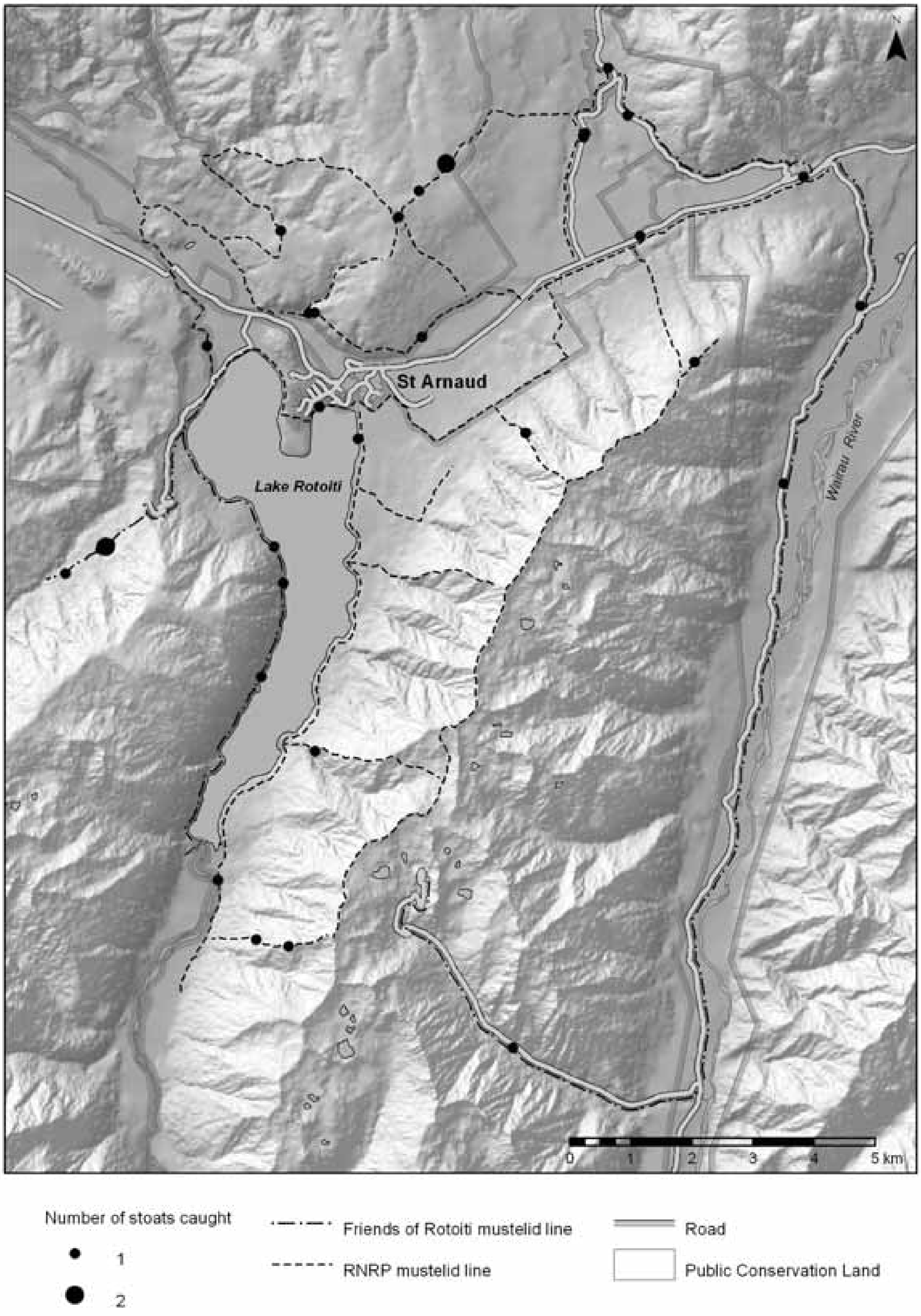

Figure 4. Map of stoat captures on RNRP trap lines for Sept to Nov 2010

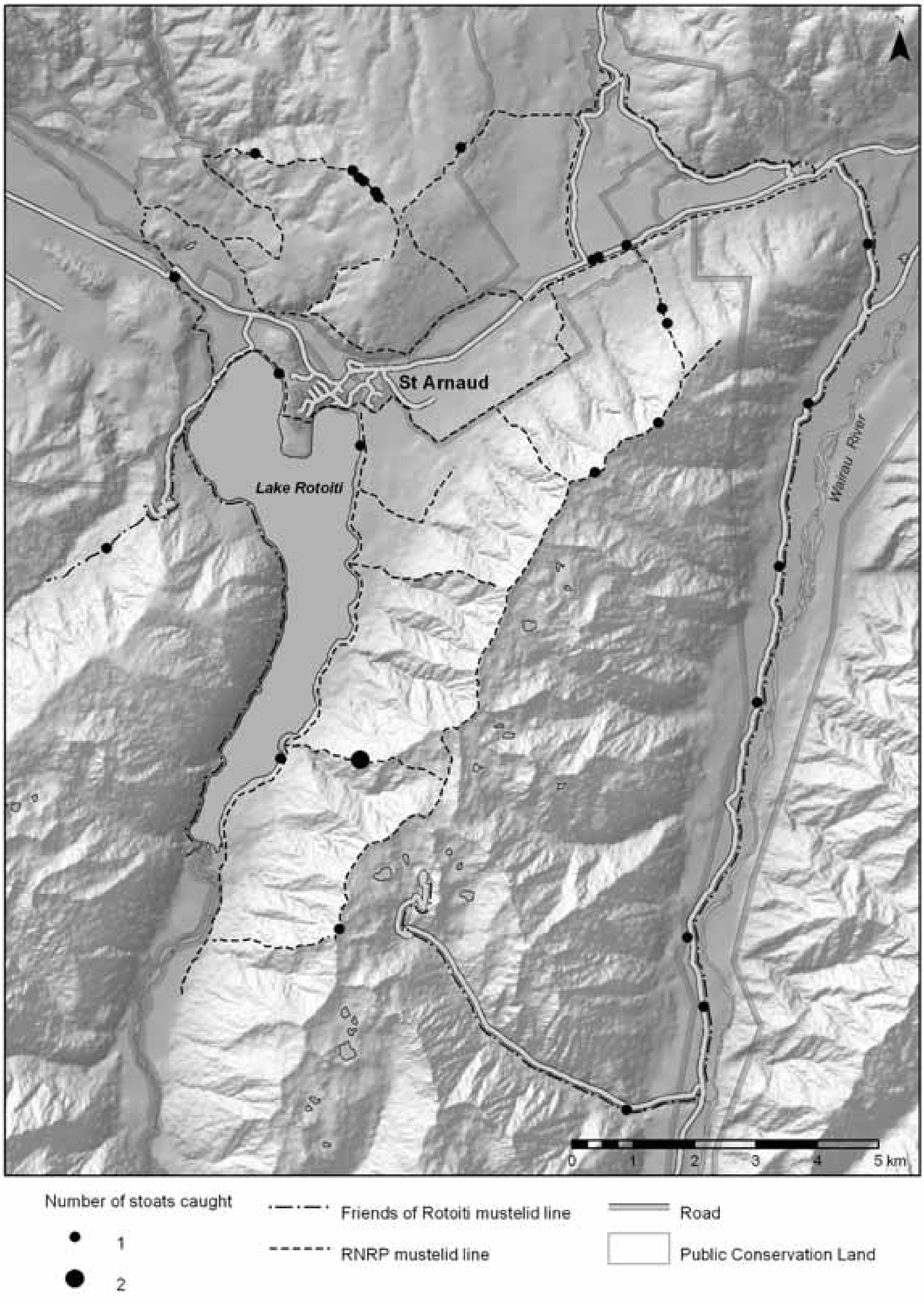

Figure 5. Map of stoat captures on RNRP trap lines for Dec 2010 to Feb 2011

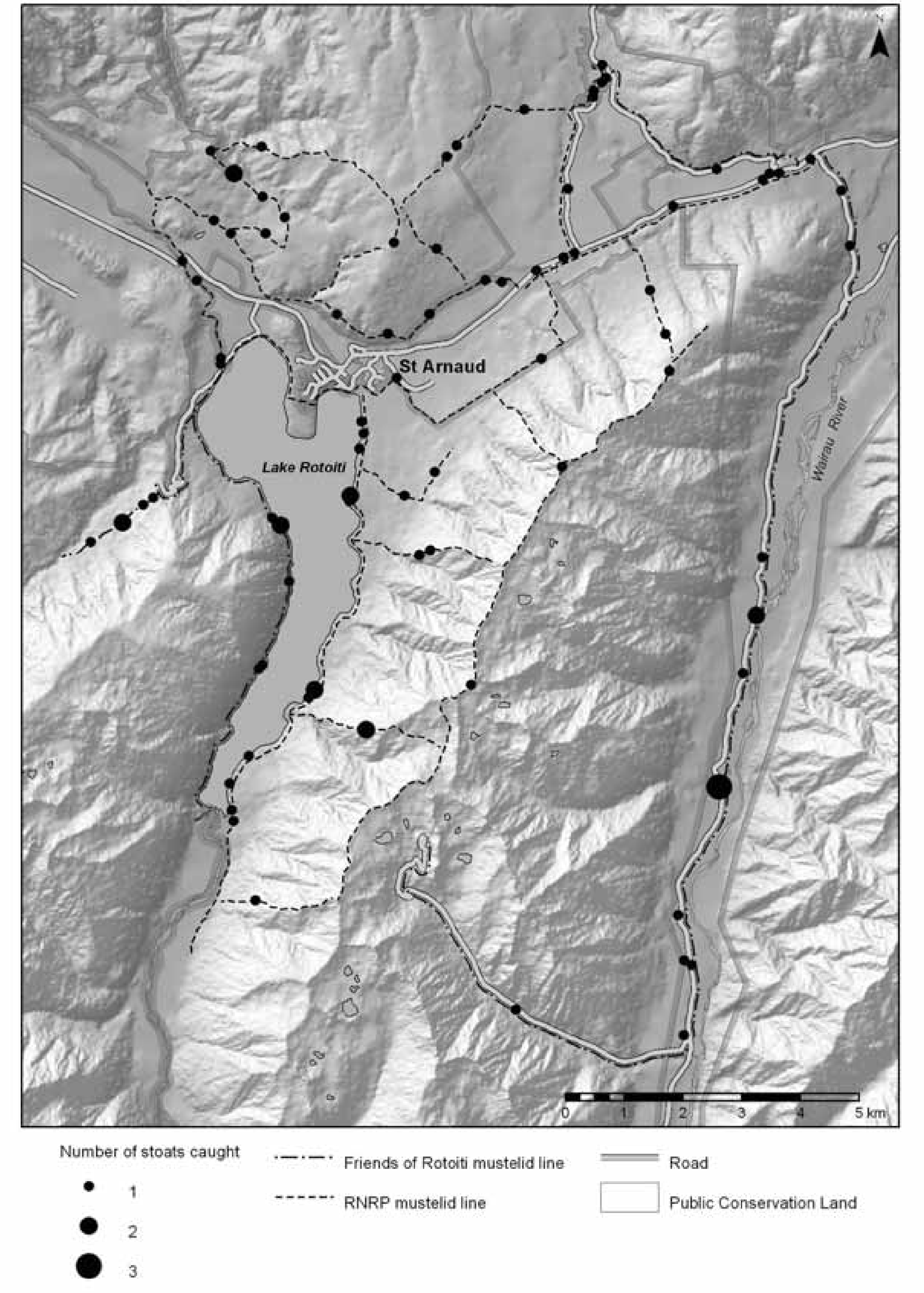

Figure 6. Map of stoat captures on RNRP trap lines for Mar to May 2011

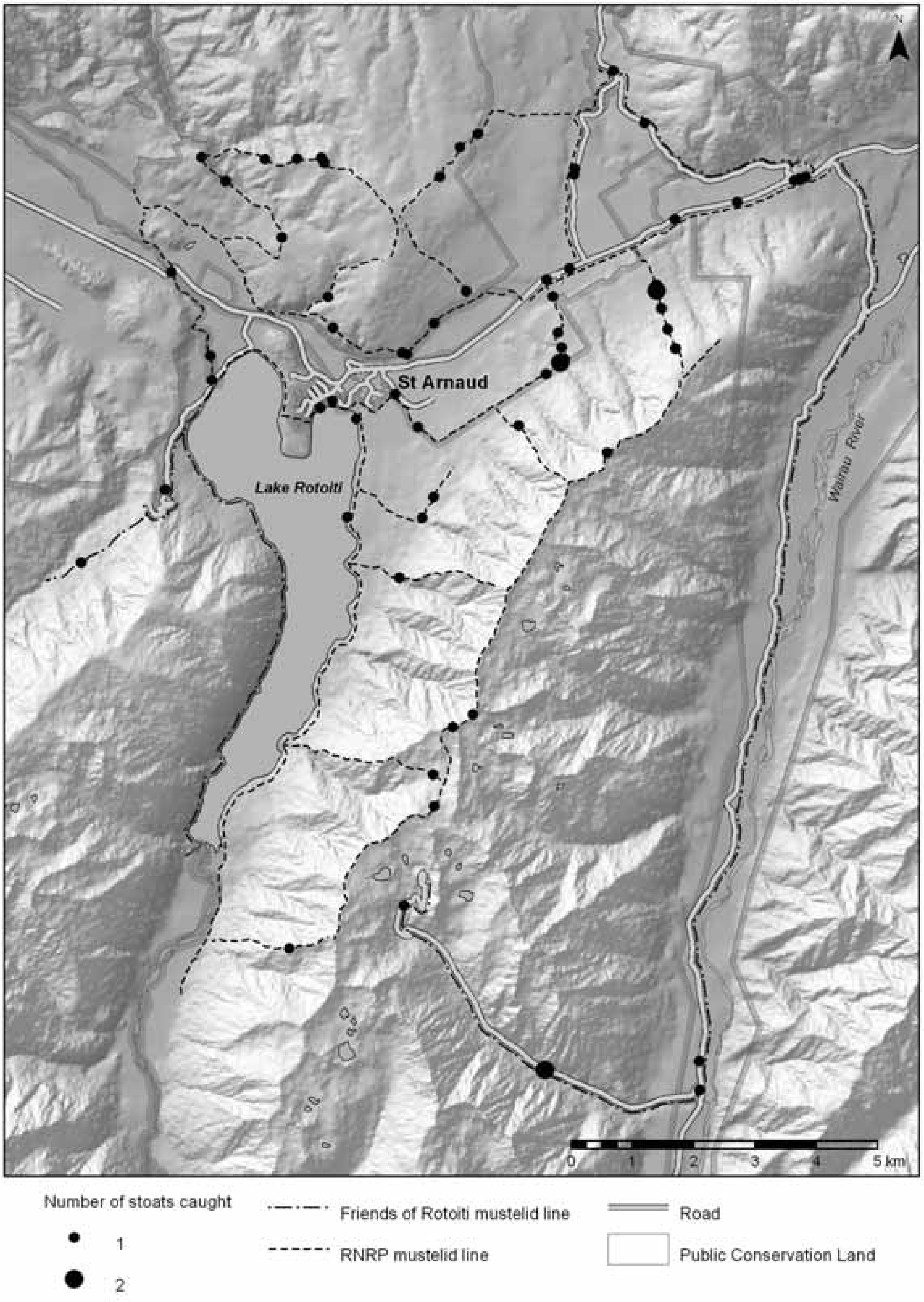

Figure 7. Map of weasel and ferret captures on RNRP trap lines for 2009-2010

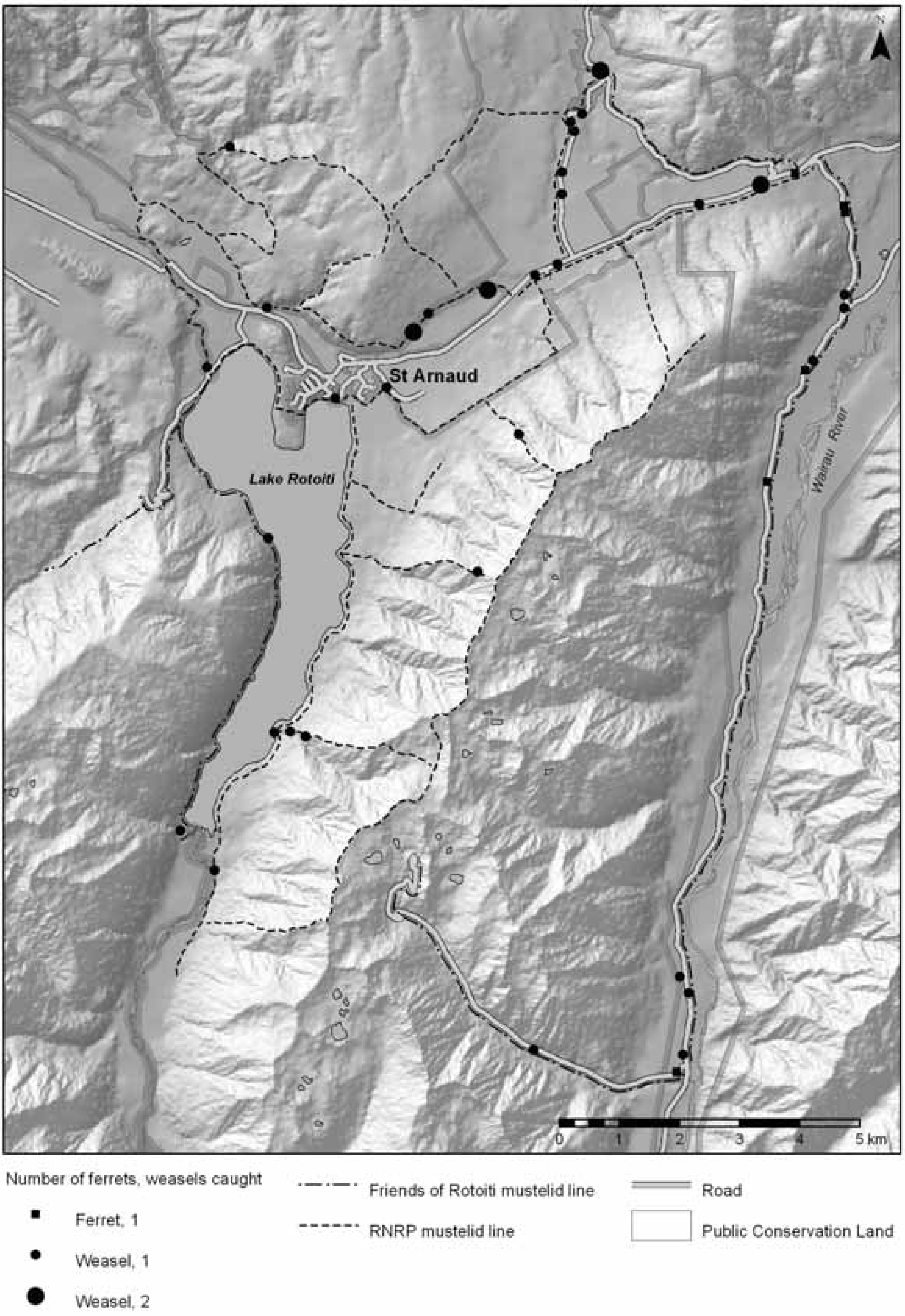

'Erayz' trial

Erayz™ is a compacted dried rabbit meat lure for carnivores. It is designed to attract mustelids to kill-traps. It is smaller, lighter and easier to handle than eggs, the current kill-trap lure that the RNRP uses, so was trialled in 2010 as a possible substitute for eggs.

Methods

Erayz was deployed on 12 traplines run by the RNRP, on each alternate trap on the lines (391 traps). Trap lines were selected for a prior history of relatively high stoat captures within the Mainland Island. The Erayz was presented on a nail, which had been nailed up through the bottom plate of the trap box. Eggs were also presented along with the Erayz, as the smell of the lure was being trialled initially. Eggs were still presented in the other remaining traps without Erayz. Checks were made at the usual intervals. The trial ran until from January 2010 until the end of July 2010.

Results

The traps with Erayz caught almost the same number of stoats as traps without (65 vs 66, Figure 8). More rats were caught in the traps with Erayz than with eggs only but the difference was not statistically significant (136 vs 117). On the lines where hedgehogs were trapped (8 traplines), traps with Erayz trapped significantly fewer hedgehogs on the lines with eggs only (17 vs 39; Fisher's Exact test:p=0.02). There was no significant difference between lines on captures of weasels (17 vs 15), cats (2 vs 2), or mice (5 vs 3).

There was a substantial amount of bait interference of Erayz by mice with 64 baits eaten entirely. Towards winter, with wetter weather, mould on Erayz also became a significant problem.

Figure 8. Captures of stoats, rats and hedgehogs in DOC200 traps with eggs or 'Erayz' as a lure.

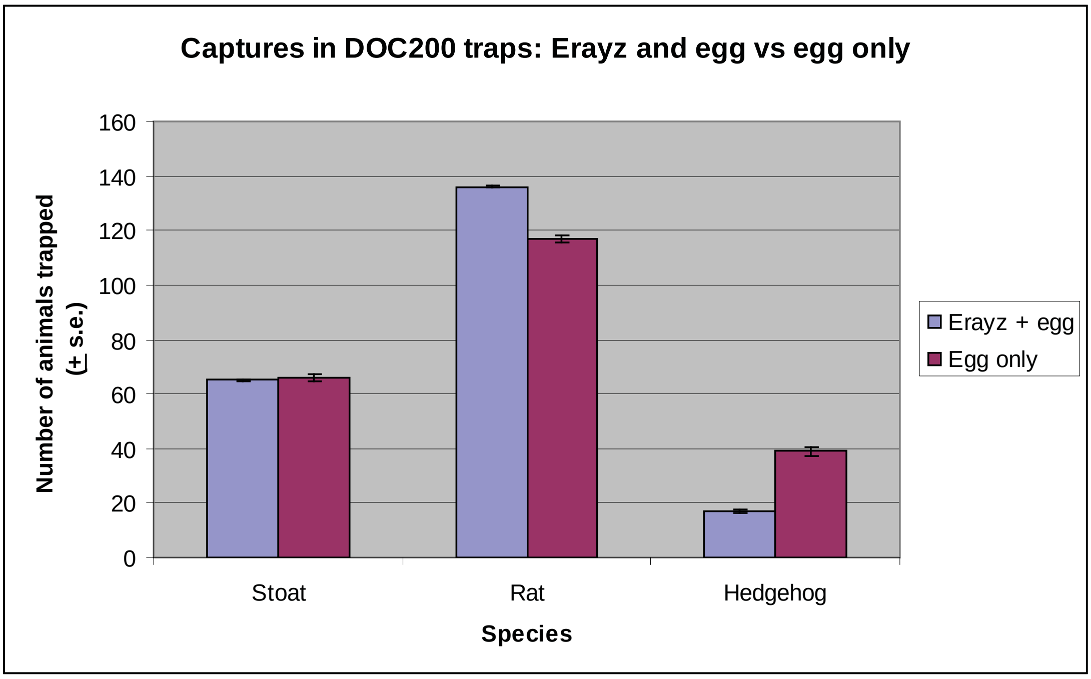

Discussion

Erayz was an easier bait to use than eggs, but there were substantial problems with bait take by mice and then mould as weather became damper toward winter. These problems eventually resulted in the trial being abandoned as fewer and fewer effective baits became available as lures. Despite the mouse population during the year of the trial being high,

mouse interference is always likely to be problematic as mice are ever present. One possible way of circumventing the problem would be to suspend the bait from the ‘ceiling’ of the trap box. Mould during the wetter months is unlikely to be easily overcome and these results suggest that Erayz would be better suited to sites with drier climates.

Hedgehogs appeared to avoid traps with an egg *and* Erayz, which was a surprise. This result should probably be investigated further and may have some application in nest protection for ground-nesting birds.

In summary , the lack of significant differences in numbers of captures of virtually all the trapped animals suggests that the problems with Erayz (mould, mice) precludes it being used instead of eggs in its present formulation.

Friends of Rotoiti mustelid control methods

Mustelid trapping lines have been maintained as a buffer to the Mainland Island, with an additional line set up this year (July 2010), with a total of 384 traps in operation.

Rainbow Valley Line: 153 DOC200s and DOC250s. Most of the odd numbers on this line are DOC250s, and the even numbers are all DOC200 traps.

Seasonal Rainbow Ski-field Line: 68 DOC200s and DOC250s. These traps were put out in October 2010 and removed for the winter in June 2011.

Mt Robert Line: 17 DOC200s.

Whisky Falls Line : 81 DOC200s. A bait trial was carried out over 27 months (December 2008 to February 2011) along this line to determine whether a rabbit/hare based long-life bait would attract as many stoats as fresh white hen’s eggs. The 41 odd-numbered traps had the polymer baits and the 40 even-numbered traps were baited with eggs. The field work was being carried out by FOR, under the guidance of Biodiversity Ranger, Kate Steffens. The project has been written up (DOCDM-716512) “A comparison of long-life stoat baits at Lake Rotoiti, Nelson Lakes National Park” in April 2011, with the following recommendations:

- the mustelid polymer bait (rabbit based) be used instead of eggs as a stoat lure when rodent numbers are low i.e. in non-mast years;
- a number of different mouse-proof holders be trialled to determine an effective means of excluding mice from polymer baits within trap tunnels;
- the attractiveness of the polymer baits (as a lure to stoats) be measured against length of time set in the field.

Note: at the end of this trial, the recipe of the Mustelid polymer bait changed from being rabbit based to fish based. This trial would have to be repeated to determine whether the new fish based bait is as effective as fresh eggs at attracting stoats to traps.

Tophouse Road Line: 43 DOC200s.

Speargrass Line: 22 DOC200s. This new line was set up in July 2010.

Trap check frequency differs to the RNRP schedule, with checks occurring weekly in the warmer months from December to February, fortnightly during March/April, and October/November, and monthly through the remaining colder months of the year. Bait changes occur every eight weeks, with polymer baits (from Trappers Cyanide) replacing fresh eggs as the bait used in all mustelid traps, from early 2011. Results of trapping are shown below (Table 1 and Figure 9)

Friends of Rotoiti mustelid control results

TABLE 1: FRIENDS OF ROTOITI TRAP LINES: MUSTELID CAPTURES 2010-11

MONTH	STOAT	WEASEL	FERRET
July	3	2	0
August	5	1	1
September	2	0	1
October	4	0	0
November	2	1	0
December	1	1	0
January	17	1	1
February	9	0	1
March	4	1	0
April	0	2	1
May	4	0	0
June	4	0	0
Totals	55	9	5

The following captures were non-target species caught as "by-catch" in the Friends of Rotoiti mustelid traps.

- 127 hedgehogs
- 16 possums
- 226 rats
- 19 rabbits
- 11 cats
- 3 mice
- 5 birds (1 sparrow, 2 starlings, 2 unknown)

There were no weka caught in the mustelid traps this year.

Figure 9. Friends of Rotoiti stoat capture per trap per line 2010-2011

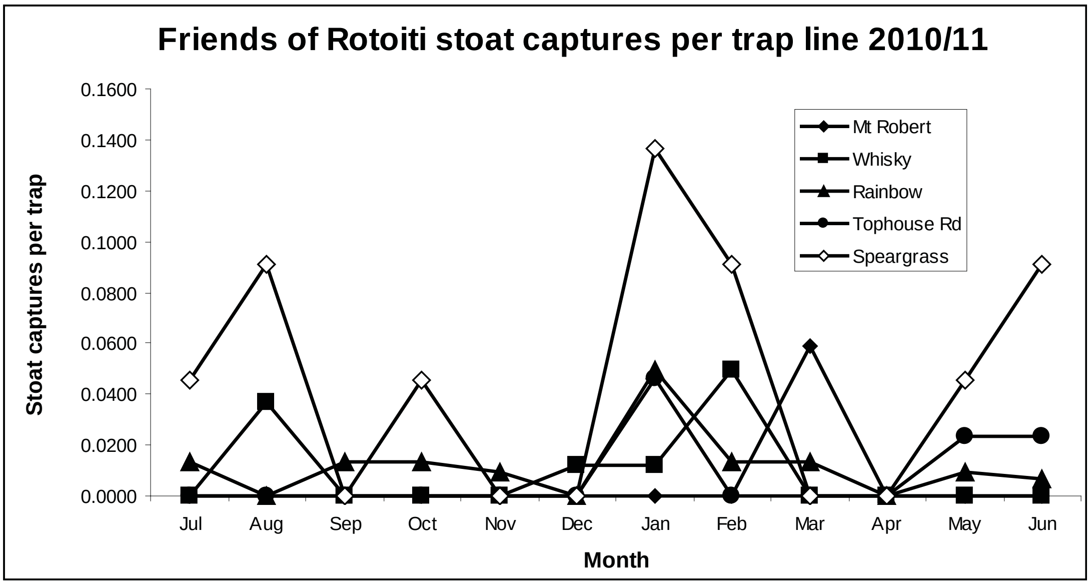

RNRP mustelid population monitoring

Introduction

Mustelid monitoring is used to compare mustelid tracking rates between the Rotoiti treatment site (trapping) and the Rotoroa non-treatment site (no trapping). The Rotoiti site includes the Core Area, Lakehead and Big Bush lines.

Methods

Mustelid monitoring is carried out using standard coreflute tracking tunnels with Trakka™ inked cards and rabbit meat bait set to the best practice method described by Gillies and Williams (2004). Refer to the 'RNRP Field Manual 09-10' for further details.

Results

Tracking Tunnels were set for mustelids in November 2010 and February 2011 (Figure 10).

Mustelid tracking at the Rotoiti site exceeded the recommended <5% mean tracking rate per line in February 2011. Maintaining stoats below the 5% threshold has been shown to be of most benefit to kaka populations (Greene et al. 2004).

Detailed tracking tunnel descriptions, results and graphs and are found in the Excel document 'Tracking Calculator 2007-08'.

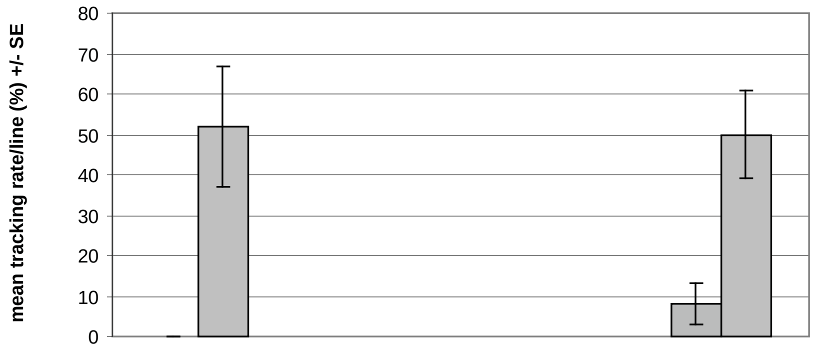

	Nov-10	Feb-11
Rotoiti (Core, Lakehead and all Big Bush lines)	0	8
Rotoroa	52	50

Figure 10. Tracking rates for mustelids recorded by the RNRP in 2010-11.

Discussion

DOC200 combination traps are in the process of being replaced with stainless steel traps as the former wear out. The stainless steel traps require very little maintenance, are easier to remove captures from and have a lower 'spring-off' weight. The reduction in the entrance hole size of the DOC250 tunnels to deter weka has largely made them comparable to the DOC200 tunnels, and therefore of less value for catching ferrets. Indeed the only ferret caught this season was caught in a DOC200 tunnel. However it is still desirable to have a trap designed to catch ferrets.

Rat by-catch this year was about average. There was also about the usual number of hedgehogs trapped. Many of the hedgehog captures were on the northern lines of 'Black Sheep Gully', 'State Highway 63', 'Borlase Boundary' and 'Dome Ridge'. The first three trap lines have substantial amounts of 'edge' in the form of pasture and forest boundaries whereas 'Dome' is entirely within Big Bush.

2.1.3 Feral cat control and monitoring

Methods

In the absence of effective weka safe cat kill traps, Havahart cage traps were used to control feral cats in and around the RNRP area. Trapping was undertaken in areas with high cat presence (sightings, scats), mainly in the spring, targeting adults before breeding commenced, and in autumn when juvenile are present. Cage traps were baited with fish frames or fresh rabbit meat. Trapped cats were shot with a .22 rifle. In addition to the targeted cage trapping, the DOC200/ 250s on the stoat trap lines continued to catch juvenile cats, though they are not designed to humanely kill feral cats

Results

In total 16 feral cats were removed from the RNRP this season (Figure 11). Ten cats were caught using cage traps, an increase from last season. Cage traps were run for 581 trap-nights, a return of 1 cat/58 trap nights. Trapping in autumn had a capture rate of 1 cat/17 trap nights. The stoat traps caught six cats, only half of what they caught last season.

Discussion

Feral cats continue to be a difficult pest to control in the Mainland Island due to the continual influx of feral cats from surrounding farmland, their apparently large home ranges and the necessity of using less effective cage traps due to the presence of weka. Cage trapping in autumn appeared the most effective time to trap as juvenile cats have been recently weaned and are hungry and trap naive. Wasp interference with bait is also declining at this time, as during summer trapping is unviable due to high wasp numbers. Cage traps are used by the public to trap feral cats in the township and assist in the control of cats near the Core Area of the Mainland Island also.

Figure 11.
Map of RNRP
cat captures
in 2010-2011

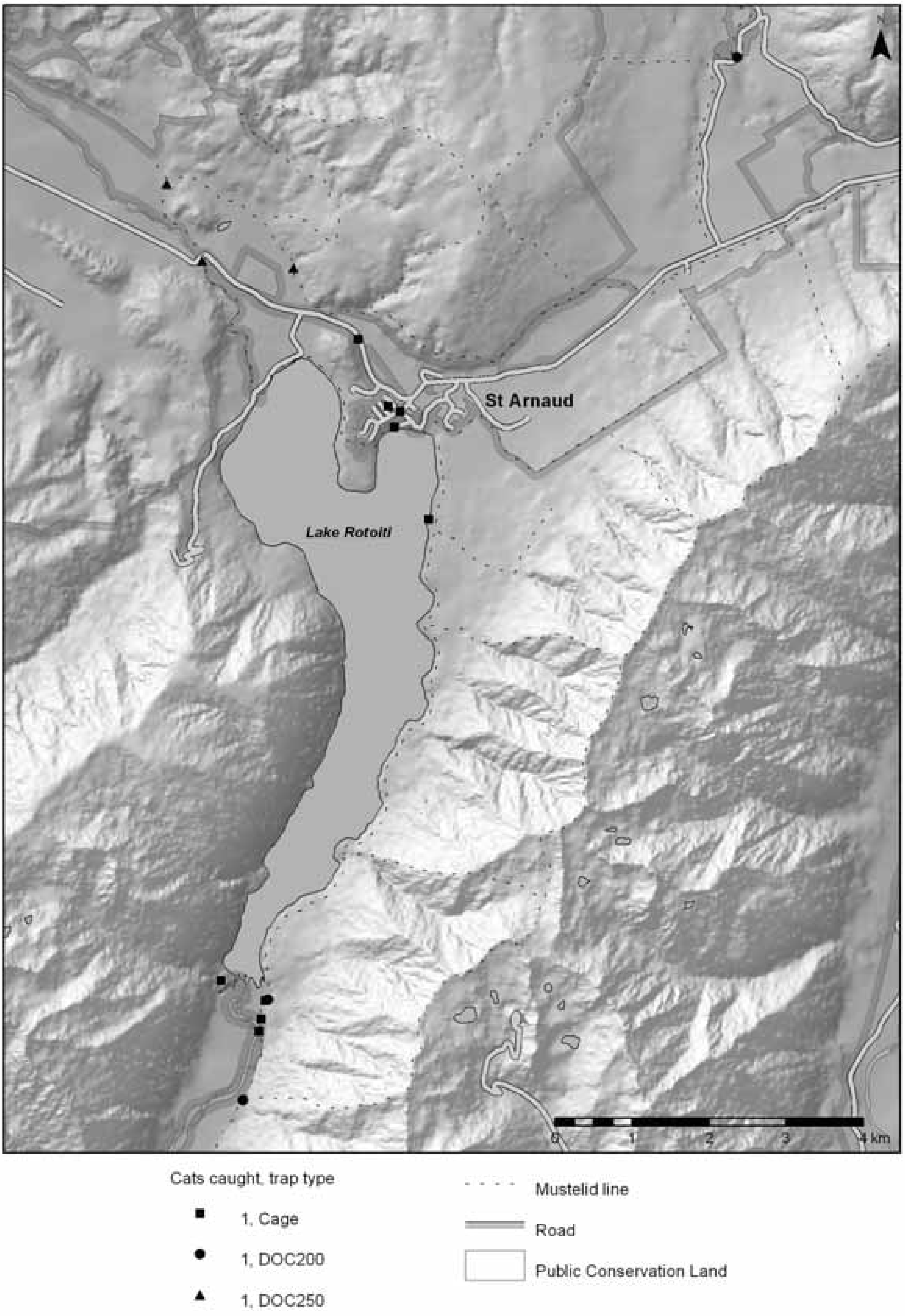

2.1.4 Possum control and monitoring

Methods

Kill trapping was maintained on the Snail, Grunt MOR and Lakehead mustelid trap lines using Sentinel kill traps baited with 'Smooth in a tube' possum lure.

The Borlase Boundary trap line which had BMI kill traps was removed due to the low possum captures and maintenance required on this type of trap. Possum control was increased to the south of the Core Area with the use of Sentinel kill traps using a rolling front method focusing on areas that had either had nil or little previous possum control.

Results

Possum captures were the highest recorded for the RNRP with 148 possums killed, in comparison to 13 in 2008-09 and 101 in 2009-10 (Figure 12). This increase in captures is due to increased effort and that some areas had either had very little or nil previous possum control, although the previous waxtag monitoring undertaken in 2008-09 did indicate a rise in possum populations within the Core Area of the Mainland Island.

The possum capture results were consistent with the mistletoe FBI results – see the 'mistletoe' section and Figure 15 for details on browse within the Core Area.

Discussion

As in previous years, trap lines situated to the south of the Core Area caught the most possums. This is due the lack of previous possum control to the south of the Mainland Island, so there is likely to be a continuing high rate of re-invasion.

The RNRP has benefitted from the adjacent recent AHB possum control operations which have kept possum numbers extremely low and minimised the number of possums moving south into the Mainland Island. However the AHB are planning to cease control in these areas after 2011 as no TB has been detected in the possum populations for some time. The consequence of this is likely to be a future increase in the local population.

Figure 12. Map of RNRP possum captures in 2010-2011

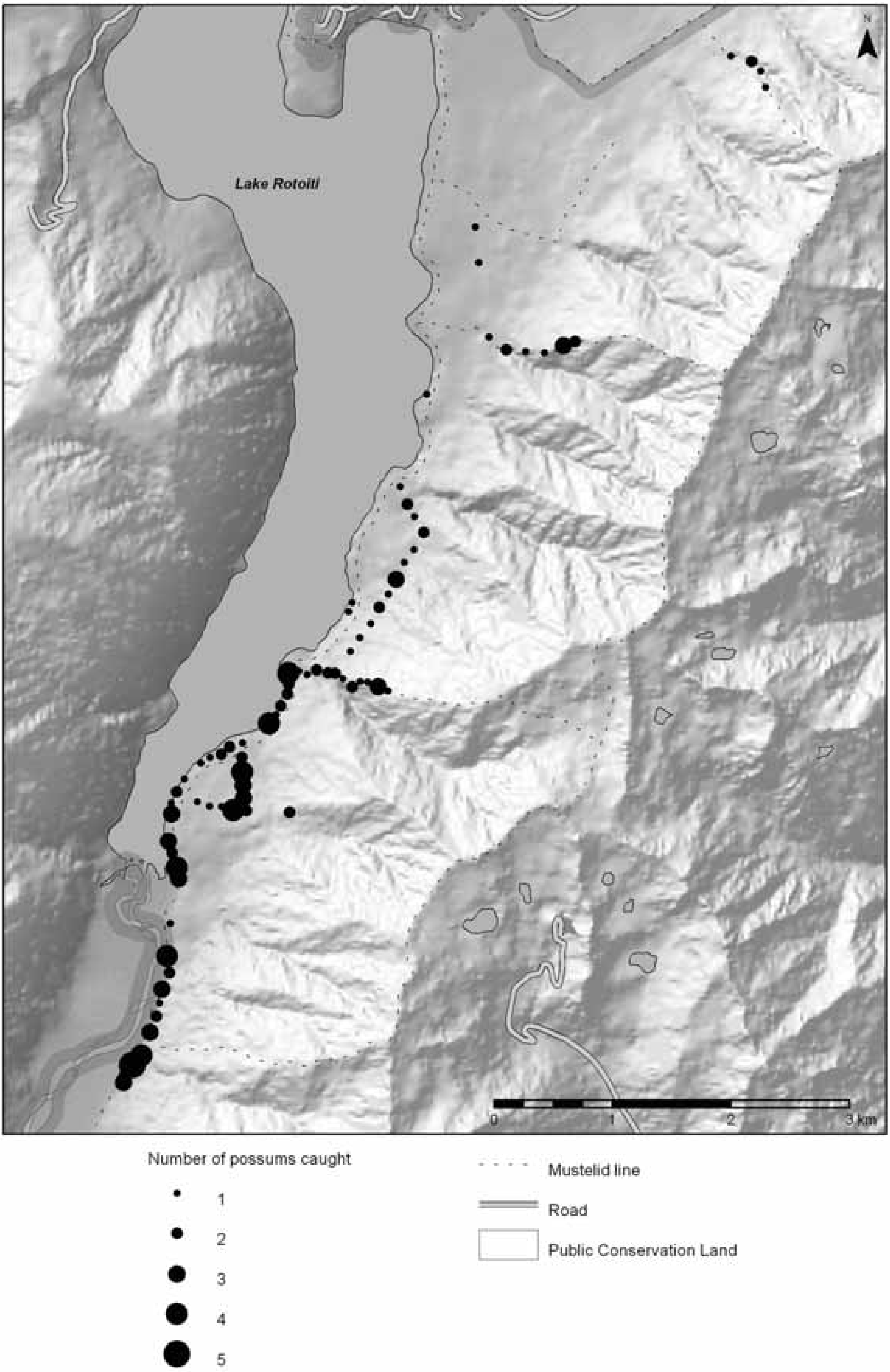

2.1.5 Deer control and monitoring

Methods

Project staff report deer sign and sightings on the St Arnaud Range while carrying out other work within the project. These signs and sightings are recorded in the Excel document 'Predator and Ungulate Sign'. Sign and sightings are only recorded for the St Arnaud Range as this is where most vulnerable plant species are present.

A system to allow principally NZ Deerstalkers local branch members access on a volunteer basis has been established. It allows hunters to book access to hunting blocks within Mainland Island.

Results

Three deer and two chamois were sighted within the Mainland Island during the season, all during the summer/autumn period.

There has been limited use of the hunting blocks since May 2010. No hunting was allowed during three months in spring due to the rat poison operation. There were 38 hunting days during the 2010-11 season and two deer and one chamois were shot.

Discussion

Although numbers of ungulates within the Mainland Island appear to be at low densities or have a very patchy distribution, their affect on native plants is likely to be biased toward particular preferred species, like *Pittosporum patulatum*. This means that numbers of browsers in the Mainland Island need to be kept low to reduce impact on rare plant species in particular.

2.1.6 Kaka (*Nestor meridionalis*) monitoring

Methods

The kaka encounter rate survey was continued this year from the beginning of October through to the end of April and run in conjunction with the mustelid trapping programme during the regular trap checks on 19 of the 24 trap lines. The other remaining mustelid trap lines are excluded as they do not traverse suitable kaka habitat.

Trapping staff record the date, the start and finish time in suitable habitat, any kaka seen or heard, the nearest trap box location and the time of any kaka encountered. No recording was done above the bush line.

Results

Ninety-two kaka were seen or heard over 339 hours with an encounter rate of 0.272 encounters/hour. The Duck Pond, Struth and the Cedar trap-lines in the Big Bush area were the lines with the highest encounter rates. No kaka were encountered on the Anglers Walk, Lakehead and the Hubcap trap lines (Table 2).

TABLE 2: KAKA ENCOUNTER RATE ON RNRP TRAP LINES (OCTOBER 2010 – APRILI 2011)

TRAP LINE	HOURS SURVEYED	KAKA SEEN	KAKA HEARD	ENCOUNTER RATE PER HOUR SEEN AND HEARD
Lake Edge	31	1	3	0.129
Lake Head	14	0	0	0.000
Hubcap	12	0	0	0.000
Snail Boundary	12	0	6	0.500
Anglers Walk	8	0	0	0.000
Peninsula Nature Walk	12	0	1	0.081
Clearwater	13	0	3	0.229
German Village	21	0	1	0.047
Borlase Boundary	17	1	2	0.176
Dogleg	11	2	3	0.442
Duck Pond Stream	11	2	8	0.877
Dome Ridge	27	4	5	0.331
Cedar	18	1	11	0.674
Struth Line	15	6	7	0.861
Black Sheep Gully	27	3	5	0.301
Grunt Boundary	19	0	9	0.474
Black Valley Stream	29	0	2	0.069
Teetotal Road	20	0	1	0.051
Middle of the Road	21	0	5	0.236
Total	339	20	72	0.272

Figure 13. Seasonal Kaka Encounter Rates for 2007-08 to 2010-11

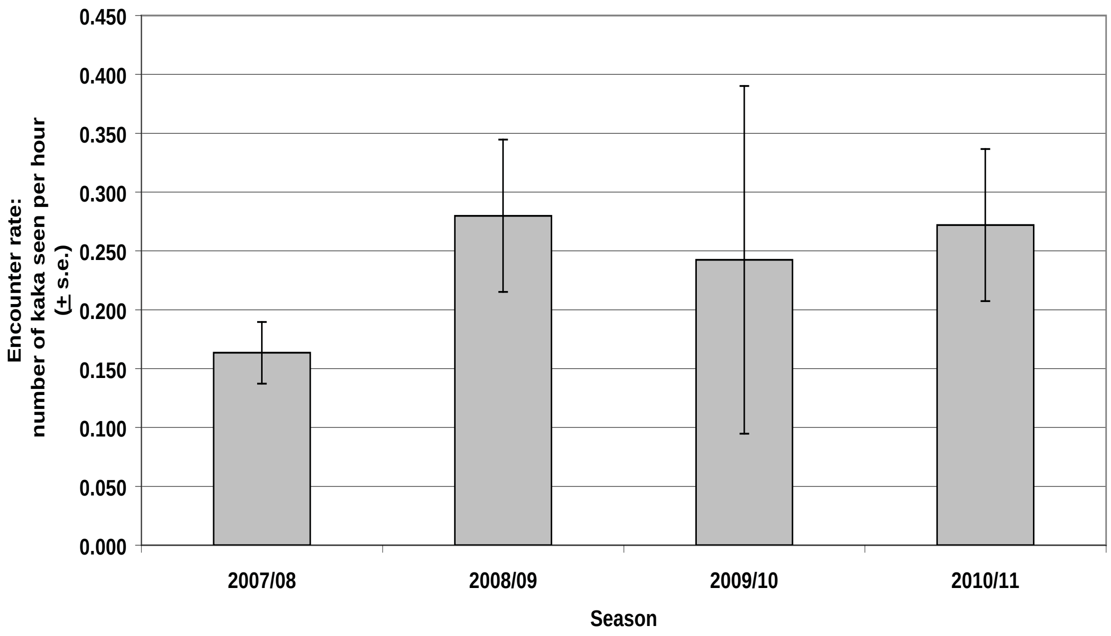

Discussion

This year a similar kaka encounter rate to the previous three seasons was recorded (Figure 13). Most kaka were recorded in lines within Big Bush, on the new Cedar line and on the two main boundary trap lines of the Core Area (Grunt and Snail), this pattern has persisted for the past three years. All these lines are also within large areas of contiguous beech forest, although this season there was no kaka encountered on the Hubcap line which traverses similar habitat. This season, and as in previous years, the fewest or no kaka are recorded on the Anglers Walk, Peninsula, Lakehead and German Village trap-lines where there is marginal kaka habitat. This current method so far appears to be suitable for measuring kaka abundance in the beech forest environment and as the results from more seasons are measured we will have more confidence in the results.

2.1.7 Kea (*Nestor notabilis*) nest protection

Introduction

Kea are present in low numbers in Nelson Lakes National Park and there is evidence for a continuing slow decline (Steffens & Gasson 2009). The Kea Conservation Trust have run kea surveys in the Lake Rotoiti/Raglan Range area in recent years and this work supports evidence of a decline (J. Kemp, pers. comm.). Kemp (1999) found little evidence of predation after monitoring 36 nests over five years but information from elsewhere suggests that possums and stoats kill kea nestlings and incubating adults fairly often. There is also evidence for lead poisoning in kea, from lead roofing nails and flashings on buildings in the alpine zone, like huts and skifield buildings.

In light of the apparently poor state of the kea population in the Nelson Lakes Area and that one of the principal agents of decline is likely to be predation on nests the RNRP has been asked to assist with nest protection in the area. The planned nest protection will be centred on known nests on the MOR ridge in the Mainland Island, one near the Rainbow Skifield Road and on one to two ridges on the eastern Raglan Range. The protection will involve use of DOC200 traps for stoats and 'kea-proof' poison bait stations for possums.

This programme will begin in the 2011-12 year.

2.1.8 Weka (*Gallirallus australis*) monitoring

Introduction

At the time of European settlement in the Rotoiti area weka (*Gallirallus australis*) were very abundant, but the population was decimated during a mass die-off in 1909. For the past century the population has fluctuated at a very low level (Steffens & Gasson 2009). The reason(s) for the lack of recovery is unknown and there is a paucity of data on habitat use, reproductive success and causes of mortality in weka in alpine beech forest. In order to better understand this species a weka monitoring programme was initiated in 2010.

Methods

Any weka sighted in or near the Mainland Island were targeted for capture, using cage traps or nets, and captured birds were fitted with backpack transmitters and a metal and colour band combination. The radio-tagged weka were checked several times a week and their location GPSd. Requests for sightings of banded birds by the public resulted in much location information also. Home ranges were mapped as weka location data accumulated. Any nests were monitored by closely approaching and noting location, number of eggs, hatch date, number of chicks and number of successful fledglings.

Figure 14. Map of RNRP Mainland Island showing locations of kaka encounters (October 2010 – April 2011)

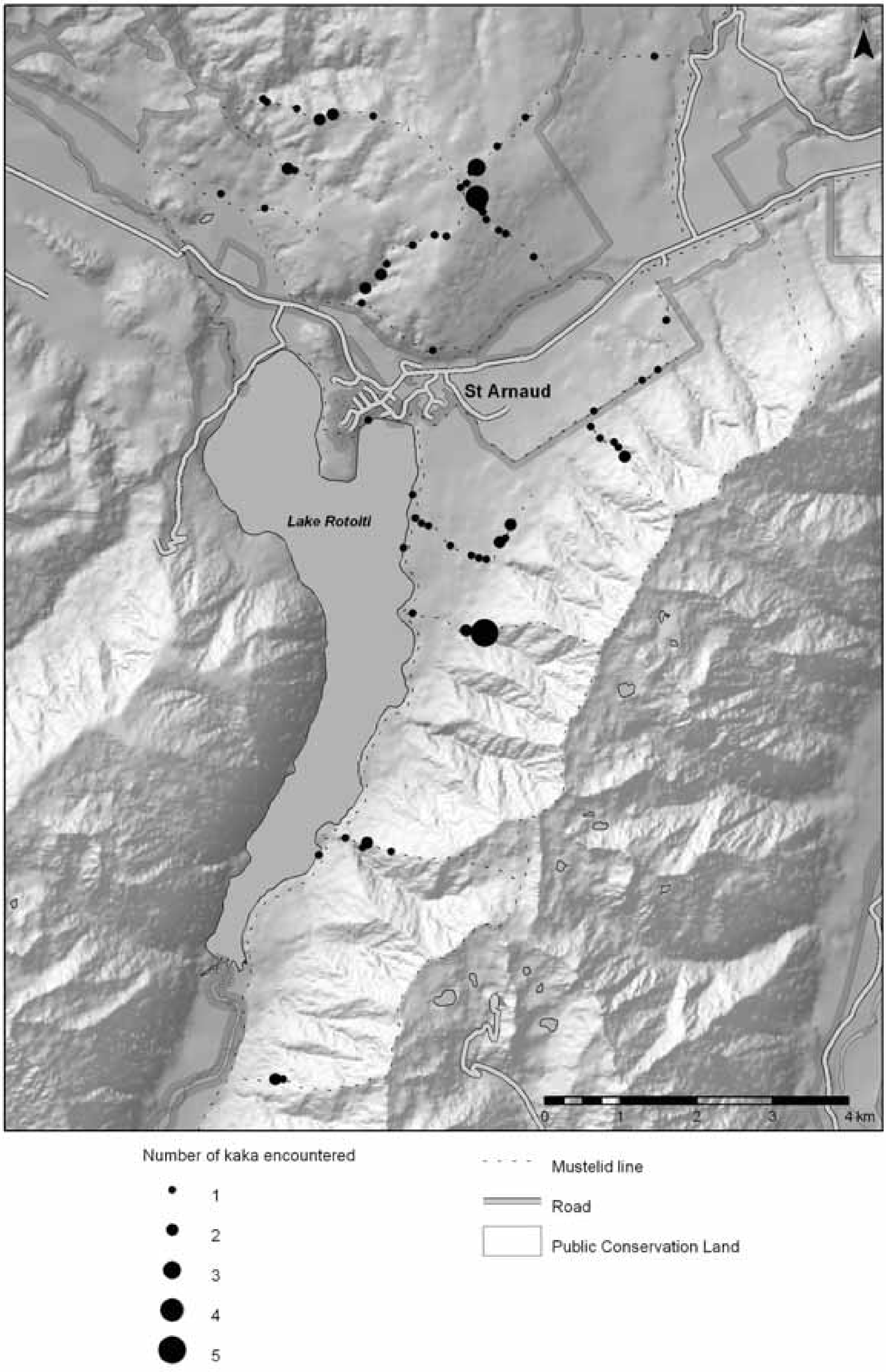

Results

Five weka (4 ♂, 1 ♀) were captured during the 2010-2011 period. One banded male subsequently died in a cage-trap set for cats by a member of the public who had departed on holiday. One male and an apparently young female established a pair bond in early 2010 and have nested twice during the period, once in April and again in June. Although eggs were incubated in both cases, neither nests were successful. The reasons for the nest failures are unknown. Two unbanded adults and two chicks which were not part of the programme were also seen by the public at Tophouse in December 2010.

The mean home range size of the five weka is about 350ha and there is a degree of overlap between home ranges (Figure 15). All the home ranges overlap in the area of the St Arnaud township. The home range of the male of the nesting pair completely overlaps the home range the female. One male has travelled 10km from its capture at Lakehead to the St Arnaud township.

Discussion

Although the weka monitoring has produced some useful interim data there are few conclusions that can be derived from it at this early stage. The RNRP will continue the current programme and plan to capture and monitor any other weka that appear in the area or indeed that are hatched here. It is likely that the information will be collated for publication in a peer-reviewed journal at some stage in future.

2.1.9 Mistletoe (*Alepis* and *Peraxilla*) monitoring

Methods

No mistletoe survey took place this year as it need only be done every 3-5 years. Re-establishment of FBI monitoring on Raukawa simplex as a biological indicator of possum damage began this year and will tie in with mistletoe surveys and wax tag monitoring to form a possum monitoring package. This was recommended by a vegetation review by Anne Brow and Mike Hawes in 2007.

2.1.10 *Pittosporum patulum* monitoring

No *Pittosporum patulum* monitoring was undertaken this season.

Discussion

At present the main problems with maintaining the *P. patulum* population within the Mainland Island is the lack of any known adult plants and the continued browsing pressure on juvenile plants. Adult plants would be dispersal points for seeds to establish within the project boundaries and would also provide an additional seed source for propagation. *P. patulum* plants can potentially remain in their juvenile form for many years until the right conditions are meet. Finding and protecting adult plants is therefore a priority. Deer appear to be the main browser on juvenile plants and the control effort for deer within the Mainland Island should be increased. A *P. patulum* enclosure to exclude deer and chamois is planned although a suitable site has yet to be selected. It is planned to erect the exclosure in 2011-2012 and plant juvenile plants into the site.

2.1.11 *Powelliphanta* sp monitoring

No *Powelliphanta* monitoring was carried out this season.

Figure 15. Home ranges of radio-tagged weka at the Rotoiti Mainland Island.

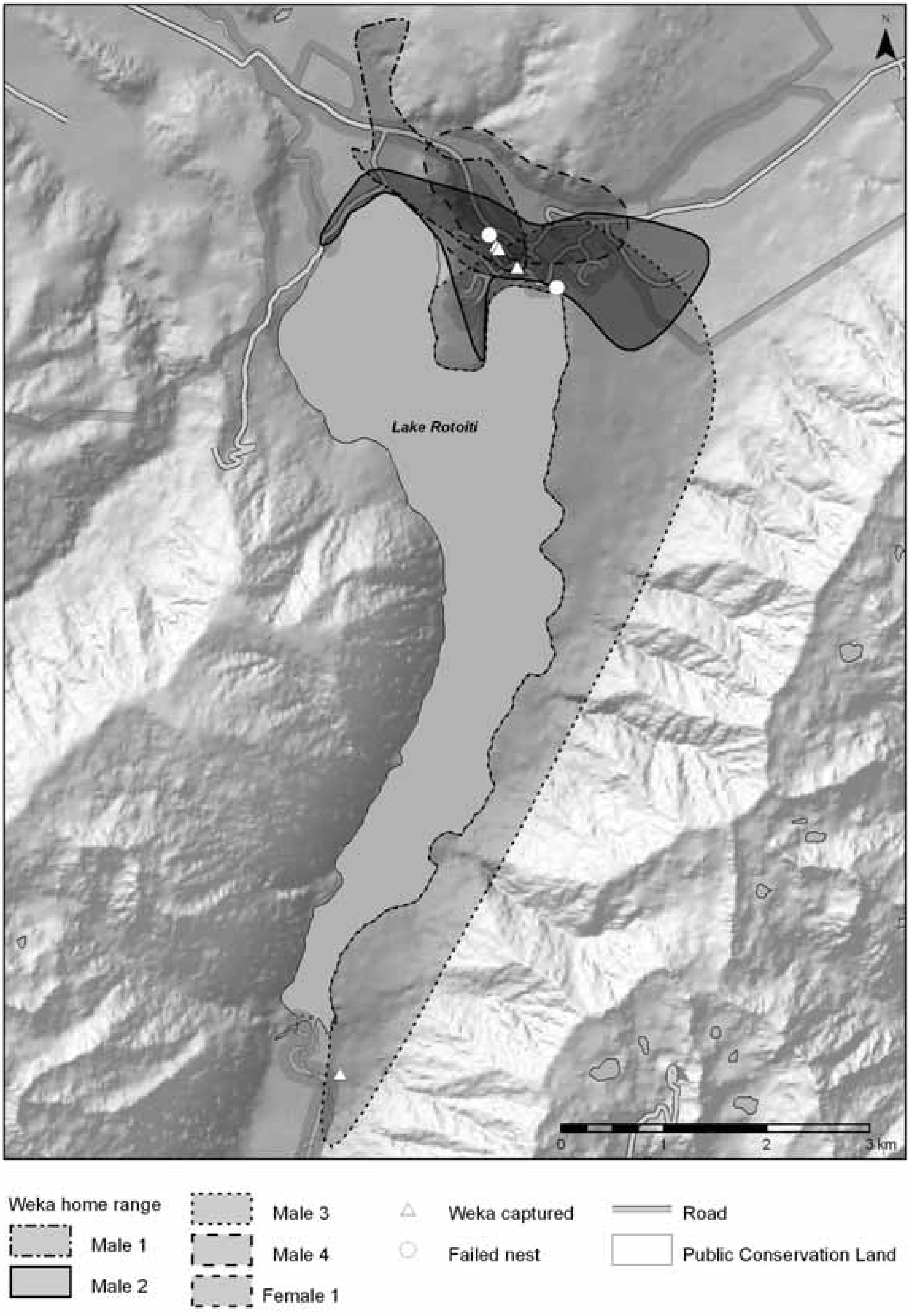

2.2 ESTABLISH AND MAINTAIN POPULATIONS OF GREAT SPOTTED KIWI AND OTHER SPECIES

2.2.1 Introduction

Great spotted kiwi (GSK) were likely present in the Nelson Lakes area early in the 20th century but have since become locally extinct (Steffens and Gasson 2009). Fourteen GSK were reintroduced to the Mainland Island in two operations in 2004 and 2006, from a population at the Gouland Downs, Kahurangi National Park. The reintroduced birds settled well and have since produced at least six chicks.

Reproductive success was not been as high as expected and a proposal to supplement the population with up to14 Operation Nest Egg (ONE) chicks, sourced as eggs from the Gouland Downs, was approved in 2008. This operation commenced in early 2009 with the radio-tagging of adults in the Gouland Downs (Brow et al. 2010). The RNRP is in its third year of ONE.

2.2.2 Great spotted kiwi (*Apteryx haastii*) population management

Methods

No close-order or direct management of the kiwi population in the Mainland Island was undertaken during 2010-09. The egg removal part of the Operation Nest Egg (ONE) programme was initiated in spring 2009 and has continued since then (refer Section 3.3.1).

Indirect management that benefited kiwi consisted of threat management, principally control of stoats which can prey on kiwi chicks. A fairly intensive effort to control cats before and shortly after ONE kiwi chicks were released removed several cats that may have depredated them. Live capture cage traps were set around the Kerr Bay-Loop Track area.

Dogs remain one of the biggest threats to kiwi nationally. Signs posted at the main entrances to the National Park are maintained to reiterate that dogs are prohibited.

Indirect management that benefited kiwi consisted of threat management, principally control of stoats which prey on kiwi chicks. Chicks were weighed and checked regularly in the first year and any transmitter in mortality mode was promptly investigated.

Results

Through BNZONE, two hard releases have taken place. One in March 2010 and another in April 2011 which has resulted in the addition of two juveniles from those releases (refer to section 3.3 for further details).

It is suspected that a male adult kiwi 'Takaka' was killed by a dog as the body was found with severe damage to the neck, although the post-mortem could not confirm the identity of the predator. There were only a few recorded minor dog offences in Kerr Bay and West Bay this past year. Most people were either unaware of the signage or were happy to remove their dog when requested.

Discussion

There has been continued trouble with transmitters failing or dropping off which entails relocating kiwi with Fen. RNRP staff have been in discussion with 'Sirtrack' to develop a better kiwi transmitter attachment to reduce the incidents of dropped transmitters and improve the fitting procedure, by reducing the time taken to attach and changing the attachment method. One promising model is being developed at present.

Dogs remain one of the biggest threats to kiwi nationally and are likely responsible for an adult death this year. New BNZ funded “Kiwi Zone” signs have been posted in Kerr Bay at both the Bellbird and Peninsula walks and also at the beginning of Mt Robert Rd in West Bay reiterating that dogs are prohibited in the National Park.

2.2.3 Great spotted kiwi (*Apteryx haastii*) population monitoring

Methods

Remote monitoring of the translocated Rotoiti population for mortality and breeding has continued this year.

Remote monitoring of radio-tagged birds for mortality and breeding attempts has continued this year. Every year the number of radio-tagged kiwi fluctuates due to transmitters failing or dropping off and individuals with no transmitter being re-located.

Kiwi Call Counting, a national community-based monitoring scheme, took place this year. It differed in scope from the 2009 call count with more permanent listening sites selected. NMIT Trainee Rangers were used for the call counts and they camped at 11 listening sites within the Mainland Island.

Results

To summarise some data from the inception of the project until June 2011:

- 15 extant adults. Seven are currently radio-tagged and eight kiwi are not radio-tagged. Two have died since release.
- Seven chicks hatched or released. Five wild-hatched and two ONE chicks.
- 32% of transmitters have either failed or dropped.
- On average two out of three monitored incubations result in a fledged chick.
- Two new pairings where a partner has left their previous partner have resulted in a healthy chick (2009 and 2011).
- Only one adult death due to predation (adult male: Takaka) recorded in 6 years within the radio-tagged population. This death appears to have been by a dog.

Annual health checks were carried out this year. All kiwi appeared healthy with no abnormalities or major weight losses.

Fen has found four individuals without transmitters in the past year (one bird twice) and located at least ten radio-tagged kiwi within the Mainland Island, some several times. Fen also located new pairs for the BNZONE programme in the Gouland Downs. There are currently two fully certified handlers for Fen with a third handler coming into the programme to fill in for the Programme Manager. RNRP dog handlers and Fen continue to be assessed by the National Dog Coordinator as Fen is the first multi-handler dog owned by the Department.

Only one nesting attempt was known to have occurred this season, between Puremahaia and Awaroa which resulted in a chick. The chick was located 14 days after it apparently hatched and a chick transmitter was fitted. The parents appeared to abandon it after this intervention and it was located by itself eight days later. This chick was killed or scavenged by a predator shortly after the last check. This chick’s weight of 190g was recorded at two weeks old, which is very light, as normal hatch weights are about 300g-350g. Therefore it is possible that this chick was in poor condition and may have eventually succumbed in any case.

Very few sub-adults have been relocated so far and only one bird found by Fen this season was thought to be a sub-adult. It was unbanded although loose/worn skin on the upper tarsus suggested a transmitter had been fitted recently. It was initially thought to be the adult male Tata as his dropped transmitter was found nearby and the bird was roosting with Wainui. However the weight and bill length was substantially heavier and longer, respectively, than Tata's measurements the previous year, suggesting that the bird was female and is possibly their offspring 'Ngahere'. Ngahere hatched about 19 January 2007 (Brow 2008), so would have been four years old.

All the wild GSK chicks hatched within the Mainland Island have been found with their parents or within their parents' home range at least a year after hatching (Table 3). Of note is the timing of nesting which have all occurred in the summer or early autumn (note: the wild chick that died in January 2011 was the earliest recorded hatch in the Mainland Island at about 9 December 2010).

TABLE 3. LENGTH OF TIME GSK CHICKS ARE KNOWN TO CLOSELY ASSOCIATE WITH PARENTS IN THE ROTOITI MAINLAND ISLAND

CHICK	EST. HATCH DATE	DATE LAST LOCATED WITH PARENT(S)	ELAPSED TIME
Miharo	1/2/2005	6/6/2008	3 years, 6 months*
Rito	1/2/2006	24/4/2007	1 year, 3 months
Ngahere	19/1/2007	31/10/2008	1 year, 6 months
Marama	11/2/2008	12/4/2009	1 year, 2 months
No. 5	6/4/2010	15/6/2010	1 year, 2 months

* Possibly longer, see previous notes.

Call counts

Approximately the same number of calls were heard as in 2009. It was clear that even though listening sites were within active territories, most birds were not heard calling.

Discussion

By the time Takaka's death in August was detected and the body located it was only clear that he had been killed. The wound, lack of consumption of the corpse and size of the bird tends to rule out predation by all likely species except a dog. There had been recent sightings of probable dog prints in the snow one spur north of the site. There is little that can be done about un-tethered dogs once they are in the Mainland Island and only ongoing education of local and visiting dog-owners can reduce the threat to kiwi.

Kiwi call count monitoring could be used in conjunction with dog surveys to monitor changes in the population if transmitters were to be removed. Kiwi call counting is not an accurate indicator of kiwi numbers in itself due to various factors, like the fact that juveniles do not call.

The loss of the latest wild-hatched chick to be hatched suggests that early management intervention threatens their well-being, despite a wild-hatched chick having been previously handled without incident at five days old ('Ngahere', on 24 January 2007). This incident has resulted in a change in management of young chicks, as parents may abandon them if disturbed shortly after hatching. Chicks may not be able to retain contact with the adults at this age and this leaves them vulnerable to predation. As GSK chicks remain with the adults for at least to 1-2 years, in future chick captures will occur about two to three months after hatching, by locating the adults and searching their burrow and/or the surrounding area for the chick.

Although information about the outcome of probable nesting activity may be lost if predation occurs, predation risk appears to be substantially lower whilst the chick shelters with their parents.

It is becoming increasingly apparent that great spotted kiwi have substantially greater parental input into chick care than other kiwi species and will closely associate with chicks for at least a year after hatching. The recent find of an unknown large kiwi roosting with Wainui, possibly their four year old offspring 'Ngahere', will be investigated and feathers taken for genetic analysis to confirm the relationship. If so, it will add to data which suggests that GSK have ongoing close relationships with their offspring for many years after hatching including sharing of the natal territory. Management of three populations of GSK in the northern South Island is producing results that suggest that GSK have substantial parental input during chick rearing and may explain why breeding apparently occurs infrequently. GSK appear to be at the 'high parental input' end of a spectrum of life history behaviour, with brown kiwi at the 'low parental input end', as this species evicts chicks from natal territories within a few days of hatching. This has far-reaching implications for population models of the species, but also improves our knowledge about how GSK should be managed. It is becoming obvious that management techniques, like ONE, developed for better researched kiwi species, are unlikely to be successfully applied to GSK without taking into account their breeding behaviour.

3. Learning objectives

3.1 TEST THE EFFECTIVENESS OF RODENT CONTROL TOOLS IN A BEECH FOREST SYSTEM

3.1.1 Introduction

Following several years of rat control using toxins (1080 and brodifacoum), the effectiveness of snap trapping (targeting ship rats, *Rattus rattus*) was trialled from July 2000 to March 2007. Throughout that period snap trapping failed to consistently achieve the performance target of sustained rat tracking index of ≤ 5%. During the 2006-07 year a 'detection and staged response' model using toxins was trialled, but failed to achieve an initial knockdown. Snap trapping was eventually abandoned in March 2007. At that stage the intention for the following year was to implement an operation using diphacinone presented in Defender bait stations, recently developed by Connovation.

During 2007-08 it became apparent that a new toxin operation would not be affordable that year and no rat control was undertaken. In 2008-09 the target rodent tracking rate in August did not reach the desired 'trigger point' of >15% and was again postponed until the next spring.

In 2010 planning for the operation focused more specifically on the reason for controlling rats within the Mainland Island. Although protection of small native passerines from predation by rats was the principal reason for the control, that actually increasing *productivity* of passerines was most the most important outcome. There were two principal reasons for this; one being that most predation of birds is the during nesting season, rather than of adult birds; and secondly, that running an operation to keep rat numbers low year-round was not required due to the reason just outlined, but also because the expense and resources required to keep rat numbers low year-round was prohibitive. Moreover, constant exposure of rats to poison runs the risk of bait-shyness developing or rats becoming immune to the poison. In essence, control of rats in spring, when birds were nesting, was all that was required to increase small passerine numbers in the Core Area of the Mainland Island.

Although no rodent control had been undertaken for several years, associated rodent population indexing and South Island robin (*Petroica australis australis*) territory occupancy monitoring, as outcome monitoring, has continued. The last beech seed-fall was in autumn 2009.

3.1.2 Ship rat (*Rattus rattus*) control

Methods

The poison operation was to take place in early spring. It was planned that 597 existing bait stations in the Core Area of the Mainland Island were to be filled with diphacinone paste (2 x 150gm of RatAbate™) in bags placed in Philproof bait stations set at 300mm off the ground. Note that the bait stations had stainless steel baffles fixed to the baseplate at the entrance to preclude bait take by non-target species like possums, kaka, weka and kea. Bait take was to be monitored and all baits were planned to be replaced in November. Tracking tunnel monitoring was to be undertaken at the usual programmed times of August and November, with one additional tracking tunnel monitoring session within the Core Area in early October. All the bait was to be removed at the end of the operation, probably in December.

Results

We undertook a single rat control operation on 1 September 2010. Fifteen DOC staff and volunteers filled the bait stations and most were finished within six hours. 179kg of bait was distributed. Monitoring of rat abundance before the operation showed high abundance of rats (50%) in both the treatment and non-treatment sites. In October, rat tracking had declined to <4% within the treatment site whereas in the non-treatment sites it was still high at 30% (refer to 'Rodent Population Monitoring'). Because of the substantial reduction in rat numbers within the Core Area the planned second poison operation was cancelled. In November tracking tunnel data revealed 1% tracking in the treatment area and 20% outside the treatment site (see Section 3.1.3). All the bait was removed from the bait stations in November. During the retrieval of the bait three dead rats were found within bait stations.

Bait was taken from 68% of bait stations and 33% (59.5kg) of the bait was consumed by weight. The modal weight class of bait taken was 75g (25% of total bait, by weight, in each bait station) at 34% of bait stations. The bait was in excellent condition when recovered, with no mould or deterioration noted. This was despite the first month being the wettest September on record with over 348mm recorded.

Some additional work was required to allow ship rats and mice access to the bait stations. Within a few weeks it was found that bait take was higher were rats had easier access to bait stations, in particular where bait stations were lower or closer to logs or trees. In light of this, all bait stations were re-visited and sticks were leant against the entrance from the ground to allow passage of rats. This appeared to result in an increased bait take.

Discussion

The bait operation was successful in that rat numbers were maintained at a rat tracking index of ≤ 5% throughout spring. In addition, outcome monitoring indicated substantially improved productivity by robins (refer to 'South Island robin monitoring').

RatAbate™ appears to very attractive to rodents, it smells strongly of peanut butter, and the bait matrix is a paste. Consequently it has several advantages, principally that it does not require a lure or pre-feeding and it is also difficult to remove from a bait station, unlike pellets. In the latter case, when the bait was retrieved three dead rats were found in the bait stations, suggesting that rats were comfortable with the bait stations and spending time in the bait stations while feeding. Of note was the excellent condition of the bait when it was recovered, despite the very wet conditions in September in particular.

The baffles appeared to work as no non-target interference was recorded, whilst still allowing access for the rats. There may have been some affect by the baffles on access and it is suggested that sticks are placed at the entrance to the bait stations to facilitate easier access, or that Philproof bait stations are attached to trees at 150mm above ground level, rather than the standard 300mm.

It should be noted that the bait operation apparently coincided with a declining rodent population, as both rat and mice tracking rates declined concurrently outside the treatment area during the spring (refer 'Rodent population monitoring', Section 3.1.3). Therefore, although the operation did significantly reduce the rat population within the Core Area, a true test of the operation will be during a beech seed-fall season with an irrupting rodent population.

The operation timing in spring appeared to be the most efficacious use of the limited funds and staff in the RNRP and has resulted in improved productivity for the outcome monitored species. In future the operation could be carried out by six-seven staff over two days, which would probably be easier to manage.

The rat treatment area only extended up to 1100m AMSL, as rat tracking had shown that rat abundance above this altitude was always extremely low so little benefit was likely to result in poisoning the sub-alpine beech forest.

Operatio`n cost

Buying pre-filled bait bags cost $2.90 per 150g bag = $3700 (for 1200 bags, ex-GST and incld. $220 for freight). We loaded our own bait bags which took 5 hours for 5 people, and this reduced the bait cost of the operation to 38% of the pre-bagged cost (pre-bagged: $3700).

Costs:	Poison	$1384.42
	Paper bags	$23.00
	**Total:**	**$1407.42**
Staff cost: 15 persons x 8 hours x ~$22/hr		$2640.00
Planning time: 1 person x 80 hrs		$2000.00
**Overall cost:**		**$6047.42**
		= $10.08/ha to lay bait

Six warning signs were purchased for $183.10, which should last up to six years.

Recommendations

It is recommend that the RNRP continue with spring operation with the proviso that this strategy will need review in mast seed-fall years when rats will likely breed earlier in the year (i.e. late autumn and winter). In these years at least two poisoning operations may be required, with one in autumn preceding the beech seed-fall.

It is also recommended that the rat control are is enlarged by an additional 400ha, to a total of approximately 900ha, to include all the eastern shore of Lake Rotoiti, up to an altitude of 900m AMSL. This control area could comprise of three blocks to allow manageable units for bait application and servicing for a small number of staff. The larger block would reduce the 'edge effect' present with the 600ha block. It is estimated that it would take six people about four days to fill all the bait stations in a 900ha control area at the present bait station density.

3.1.3 Rodent population monitoring

Methods

Rodent monitoring is based on the use of tracking tunnels to provide a relative abundance index of rodents within the Core Area compared with Lakehead and Big Bush (no rat control but mustelid trapping) and Rotoroa (no control of any species). For the past five years no rat control has been carried out in the Core Area so the tracking rate has provided a background rate for planned rat poisoning operations.

Rodent monitoring is carried out using Tracka™ cards with a peanut butter and oat mix as a lure, set in black corflute tunnels (Gillies and Williams 2004). Refer to the 'RNRP Field Manual 09-10' for further details.

Results

Mouse tracking (Figure 16) declined at all sites through spring from the very high rates in 2009-10 and has not significantly increased since then. Whether the rat control operation had much affect on mice is a moot point as their tracking rates declined more steeply at Rotoroa where no control was undertaken.

Figure 16. Tracking rates for mice at Lakes Rotoiti and Rotoroa 2010-11

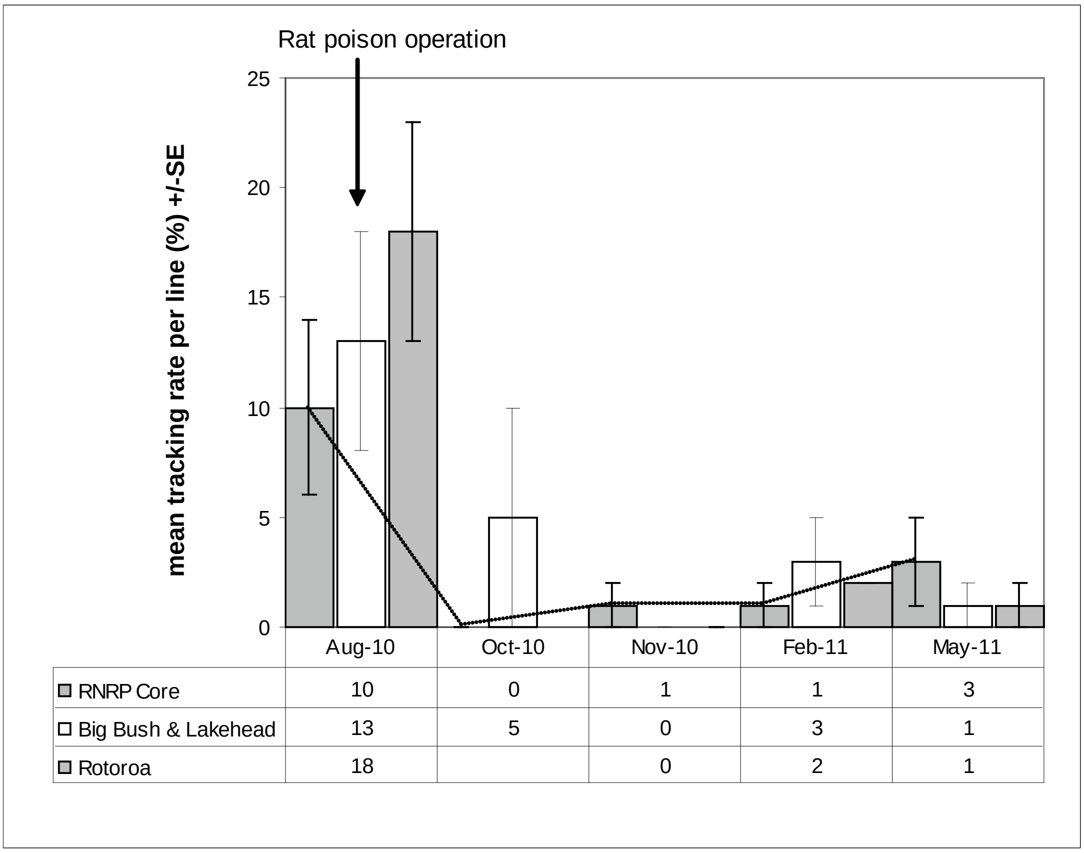

	Aug-10	Oct-10	Nov-10	Feb-11	May-11
RNRP Core	10	0	1	1	3
Big Bush & Lakehead	13	5	0	3	1
Rotoroa	18		0	2	1

Rat tracking rates declined sharply within six weeks in the Core Area after the rat control operation (Figure 17) whereas at the untreated Rotoiti sites the decline in tracking rates continued only slowly until late summer. All sites at Rotoiti had a small increase in tracking rates by autumn. Rat tracking at Rotoroa remained at 5% or less throughout the entire period.

Figure 17. Tracking rates for rats at Lakes Rotoiti and Rotoroa 2010-11

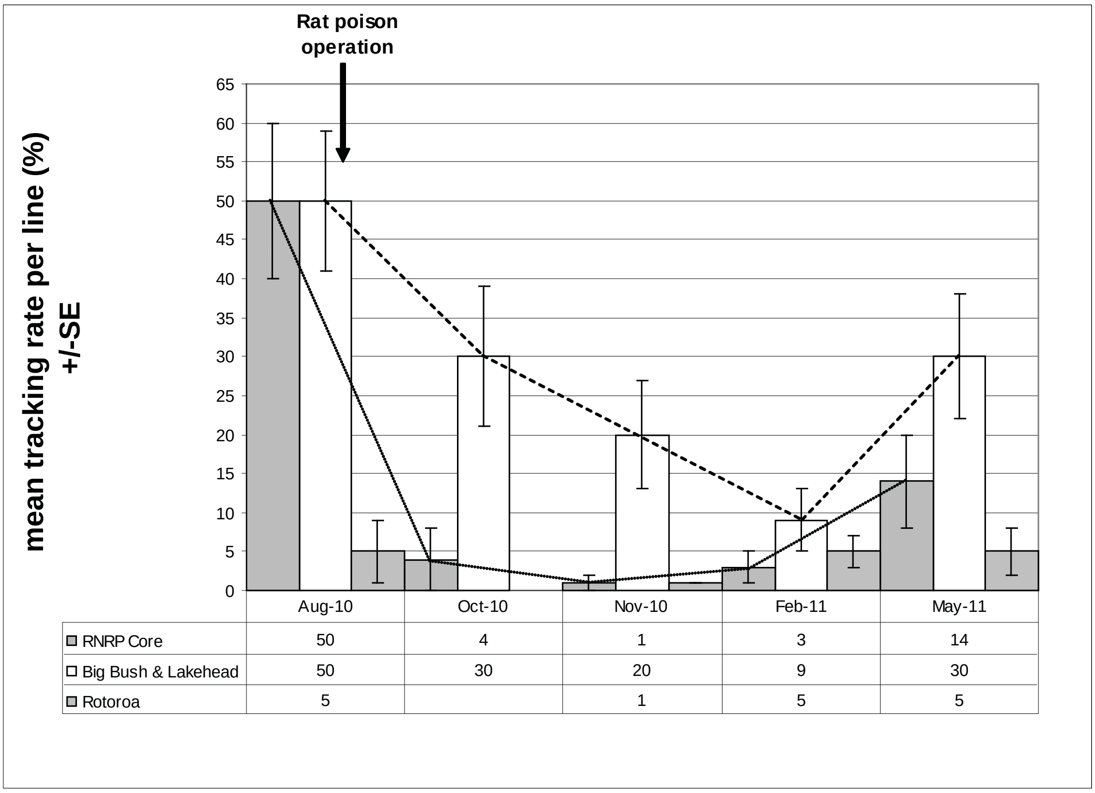

	Aug-10	Oct-10	Nov-10	Feb-11	May-11
RNRP Core	50	4	1	3	14
Big Bush & Lakehead	50	30	20	9	30
Rotoroa	5		1	5	5

Discussion

The rat control operation in early September had an obvious affect on rat tracking rates when compared with Big Bush and Lakehead and the concurrent improvement in robin nesting success confirmed the success of the operation. There was a fairly heavy snowfall in September which may have also hastened the decrease in rodent population numbers, particularly mice. It appears the operation has simply knocked down a population beginning an overall decline, although the knock-down of rats in the Core Area hastened the reduction in numbers which have barely recovered by the winter of 2011. A repeat of the rat control is now planned for every spring to protect breeding birds, but the true test of the rat control operation will be whether the RNRP can control rat numbers during a large beech seed mast event. The extension of the Rat Control Grid out to about 900ha, south of the current grid, should reduce the edge effect and slow reinvasion into the treatment site.

3.1.4 South Island robin (*Petroica australis australis*) monitoring

The South Island robin is an endemic passerine, and although the species is classified as not threatened (Miskelly et al 2008), it has declined dramatically since European settlement as a result. This is primarily due to habitat loss and mammalian predation (Bell 1986). Robins are territorial year-round and mainly breed in spring, although the robin breeding season was from August to February at Rotoiti in 1998-99 (Etheridge and Powlesland, 2001) and in 2010-2011 (G. Harper, pers. obs.)

South Island robins have been monitored within the Core Area of the Mainland Island since 1998-1999 to measure the effectiveness of rat control operations.

Methods

To determine the total number of paired robins and unpaired individuals in a defined area within the Core Area at the start of the breeding season a census was carried out. A survey is conducted four times, a week apart every September. The study area (162.1ha) is shown in Figure 18. Until 2007 the study area was approximately 120ha, but was expanded south of the Loop Track to increase the survey area, as so few robins were being located in the few years prior to this.

Pre-census: A combination of audio and 'tapping' the mealworm container was carried out within the census area in order to attract robins for colour-banding for individual recognition. An audio system was used to call the robin into the vicinity of the observer. When the robin was nearby, the audio was switched off and the mealworm container was tapped at the same time as mealworms were being fed to the robin in order to train it to come to a 'tapping' sound. We attempted to band all robins within the census area. This pre-census work was carried out during July and August. Two appropriately trained staff were required for five to six days.

Census: Three staff were required for one day a week over four weeks throughout September. The census site was split into three areas for ease of monitoring (one person per area per day of surveying). Each surveyor walked slowly along each line while tapping a mealworm container, they stopped at every second bait station for 1-2 minutes and tapped loudly to attract robins.

- If a robin was sighted, the container is tapped until the robin approached and is then fed as a reward. The following information is recorded: the band combination (or 'no bands' if none present), sex, date, whether paired or alone, observer, location and behaviour e.g. eating mealworms, caching mealworms, flying off with mealworms. These behaviours indicate whether it has a nesting partner nearby.
- If a robin was not sighted the surveyor continued walking and tapping along the line.

This data was entered into an Excel spreadsheet. If an un-banded robin was sighted during the survey, subsequent attempts are made to capture and band the robin shortly after.

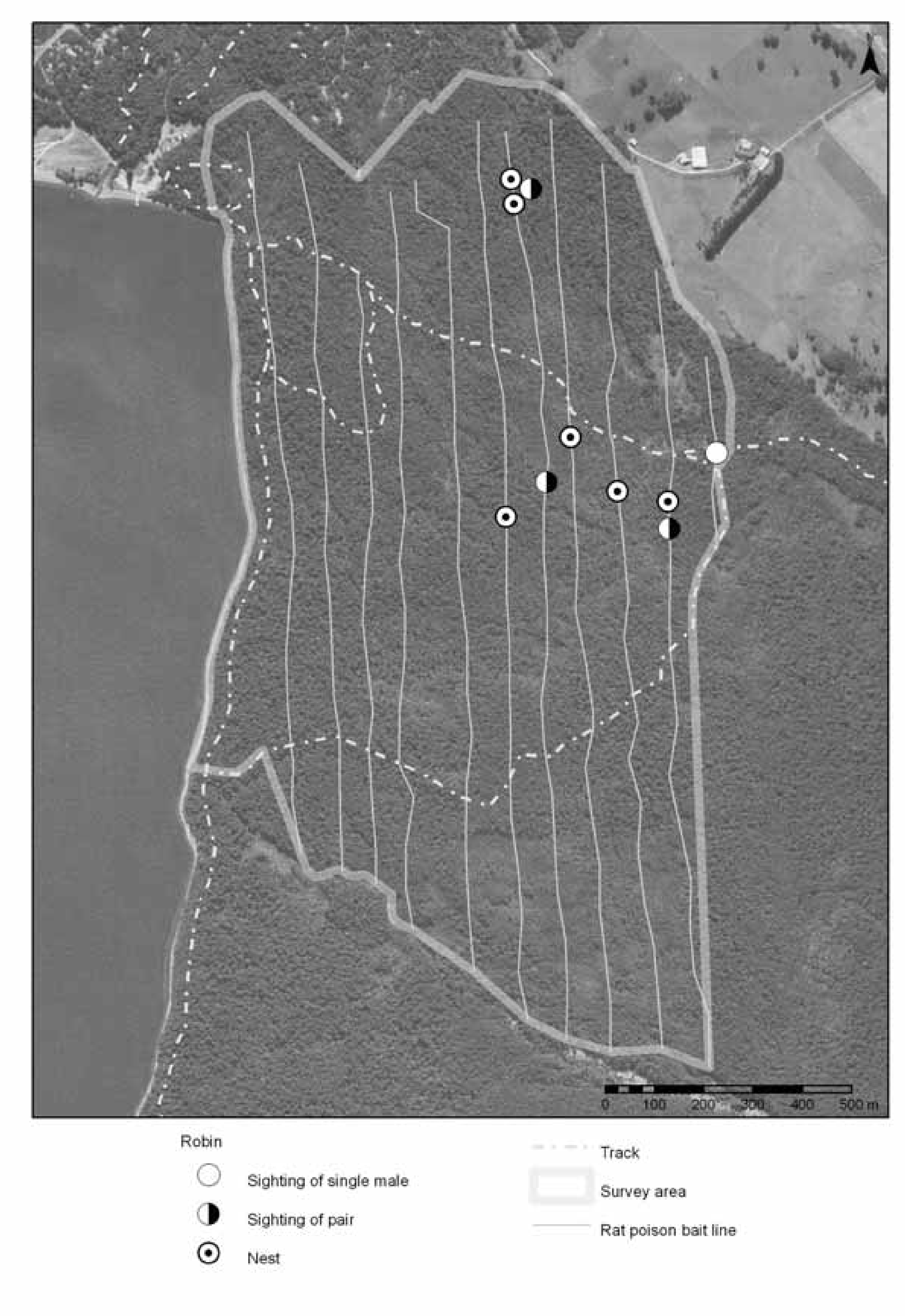

Figure 18. Map of robin survey area within the RNRP Core Area 2010

Results

In the pre-census and census period in 2010 four robins (1 ♂; 3 ♀) were banded within the study area. During the 2010 census three pairs plus one male, all banded, were counted. The three pairs (♀ = YM-DB; ♂ = M-O), (♀ = M-LB, ♂ = YM-W), (♀ = YM-LG; ♂ = M-W) were seen at bait stations WG7, LH12, and LJ7 respectively. The single male (RM/YR) was observed at bait station LA19 and this was his second year he was seen to be unpaired. The first nest was found on 10 September, but failed for unknown reasons. After that three nests were found in October, one in November, and another nest was estimated to have had eggs laid in mid-December. The three pairs each had two nests. One failed and five are known to have produced fledglings. A minimum of 11 and possibly up to 15 chicks fledged successfully. All the three adult females that nested were located in February. Several banded fledglings were seen several weeks after fledging and two have been seen some distance from the Core Area. One was seen at Lakehead, some 9km away, in February and another near the MOR trapline in March, about 5.5km from its nest.

Robin numbers within the RNRP Core Area have fluctuated since 1998 (Figure 19) but generally the number of robins counted has declined. 2004 (n = 3) and 2009 (n = 4) were the seasons with the lowest counts. In general, robin numbers tended to decline when only rodent trapping was carried out and the number of robin pairs increased following toxin operations, with a lag of about one year. It is important to note that robins were not banded in 2007 and 2008 so it is possible that some robins were double counted and density may be a little high for these two seasons.

Figure 19. Robin density and number of pairs in the Rotoiti Mainland Island Core Area during differing rodent control regimes.

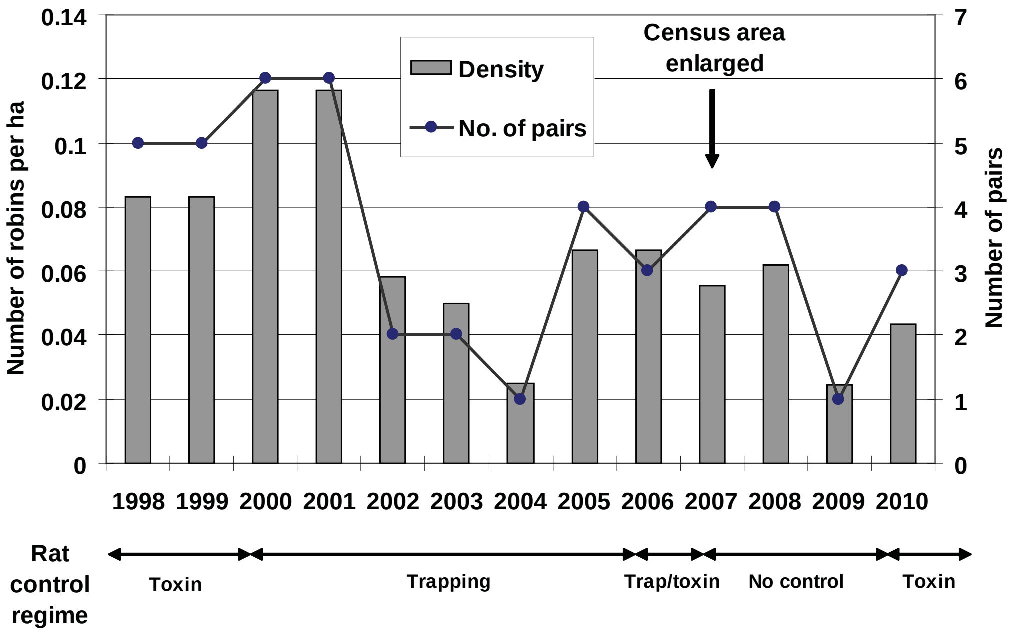

Discussion

The rat control operation in September appears to have resulted in the first recorded robin breeding within the Core Area for at least four years. Three breeding pairs produced five successful nests.

Considering the size of the area surveyed, 162.1ha, the 2010 population of seven robins, with only three breeding pairs, remains at low density. With control of rats continuing it is likely there will be an increase in robin numbers as there appears to be a lag between control using toxin being initiated and a response in the population size. However, it is highly likely that factors other than rat predation also affect robin populations, like heavy snow events or

low invertebrate numbers, so consistent increases in population size cannot be guaranteed. There also appears to be at least some dispersal of juveniles which is expected. The planned extension of the rat control grid up to 900ha should protect additional robins as well.

3.2 TEST THE EFFECTIVENESS OF WASP CONTROL TOOLS

3.2.1 Introduction

From 1998 common wasps (*Vespula vulgaris*) have been controlled in the Core Area of the Mainland Island using various protein-based baits containing mainly the toxins Finitron or Fipronil. This work was originally carried out in close association with Landcare Research and then more recently with the Nelson-based company Entecol, which is currently the only supplier of the toxic bait X-stinguish (0.1 % Fipronil). Fipronil has proven to be the more effective of the two toxins but access is currently constrained by commercial imperatives and is only available under an experimental use arrangement. Finitron has also not always been available and due to the unavailability of any suitable toxin there was no wasp control undertaken during the 2006-07 season. However the following season X-stinguish™ was again available for experimental purposes and has been used for subsequent operations since then. Experimental trials by the community group 'Friends of Rotoiti' (FOR) in the Big Bush area during the 2008-09 summer suggested that effective control of wasp nests could extend up to 350m from poison bait stations (Brow et al. 2010). As a result of this trials in the last two seasons have focused on determining the widest possible spacing between wasp bait lines while still achieving the desired reduction in wasp densities.

3.2.2 Wasp control and monitoring

Methods

To ensure that the toxic operation will be effective, pre-operation monitoring of wasp visitation on non-toxic protein-based baits is carried out. An average of one wasp per bait is considered as the trigger point for initiating the decision making process to start the toxic operation. For further detail on wasp monitoring and the decision making process refer to the 'RNRP 2010-11 Field Manual'.

In view of the FOR trial results and before the start of the 2010 operation, the spacing of the bait station grid within the Core Area was increased from 200m between lines to 400m. Lines run horizontally across the slope and bait stations are still maintained at 50m intervals along these lines. This season the same bait station array was repeated in the Core Area. In addition a new bait station grid with 400m lines apart, running vertically, with two bait stations at every 100m, was established in Big Bush. The rationale for trialling vertical lines (up and down hill) was to test the theory that wasps may be able to carry toxic bait further distances across a slope and may also potentially forage further from their nest across a slope. Within the Core Area and Big Bush wasp nests were marked along transects and flight counts taken pre-poison and repeated post-poison which was around three weeks after the wasp control operation. Two additional sites, to the south of the Core Area were re-trialled again, each with of a cluster of eight bait stations. Around these two sites wasp nests were located and marked at distances up to 325m from the bait stations cluster, flight counts were also taken pre-poison and at three weeks post-poisoning. The third cluster at head of Lake Rotoiti that was trialled last season was not re-trialled due to the different habitat found in that area and last years result not useful for comparison with the other two clusters.

X-stinguish™ bait (protein matrix with active ingredient Fipronil 0.1%) packaged in 1.5 litre plastic pails was obtained from the Entecol lab in Nelson in early January 2010 and stored frozen till the day before the wasp control operation. This season the operation was started on the 15 February 2011 with toxic bait laid in the RNRP Core, St Arnaud Village/Brunner Peninsula areas. Bait stations in the Big Bush grid, the cluster sites and the line on the western side of Lake Rotoiti (filled by the FOR group) were filled on the 16th February. All bait stations were filled with 40g of bait, with the exception of the two cluster sites where around 65g was placed into each of the eight stations. For further information regarding the bait and bait station layout, refer to the 'RNRP 2010-11 Field Manual'.

Operational performance standards specify that uneaten bait must be collected from bait stations within five days of application. All uneaten bait was retrieved and weighed to determine the amount of bait taken.

Results

The average number of wasps observed on non-toxic baits was 5.2 on 24th January 2011 but on 14 February 2010 it was only 0.975. Although this later result was below the threshold it was decided, especially considering the earlier result, it was still high enough to initiate the poison operation.

Including all of the DOC and FOR operations a total of 39.6kg toxic bait was deployed. Although the bait take was still relatively low with 24.6kg (62.2%) removed by wasps, it was higher than 47% for the 2010 operation.

After three weeks the operation only achieved a 56% overall reduction in flight counts of marked nests inside the Core Area and between 150m to 200m from bait stations there was only a 12% reduction observed (Figure 20a). This is compared to an 83% overall reduction in the Big Bush grid where nests were monitored out to around 250m from bait stations and there was still a 59% reduction observed between 150m to 200m (Figure 20b).

The two trial bait station clusters (C and D) showed mixed results, one site (C) was relatively effective out to a radius of approximately 200m, reducing overall wasp flight count numbers by 88%. Also between 150m and 200m there was still a 73% reduction, although by 200m to 250m there was only a 7% reduction. The other site (D) only reduced overall wasp counts by 76% inside the 200m radius and there was only a 49% reduction of flight counts between 150m and 200m. The combined results from both clusters show that between 100m to 150m flight counts were reduced by 81% but were only reduced by 43% between 150m to 200m (Figure 20c).

Discussion

Due to the apparently low wasp numbers the 2010-11 wasp control operation was again initiated later than in previous years and although higher than last season the reduced amount of toxic bait taken this year could have also been the result of the perceived lower number of wasps.

The toxic operation was successful in reducing the wasp nuisance around the St Arnaud Village.

Wasp activity within the Core Area was observed to fall within a few days of the operation, although there were still some active nests noted in the following months. Preliminary results inside the Core Area also indicate the operation was not particularly successful, even though monitored nests were all located within 200m of bait stations. Results also indicate that by 100m the reduction of wasp numbers probably wasn't enough to get below the threshold at which wasps are still having a detrimental impact on the ecosystem.

Figure 20a. Decline of wasp nest activity within the Mainland Island Core Area after poisoning

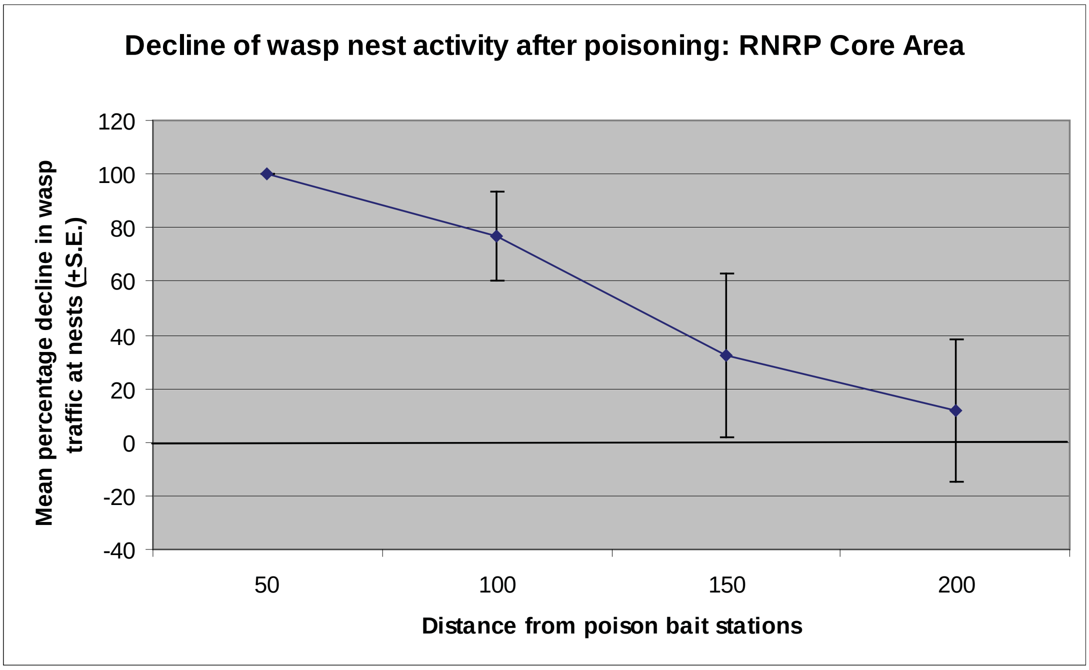

Figure 20b. Decline of wasp nest activity within the Big Bush Area after poisoning

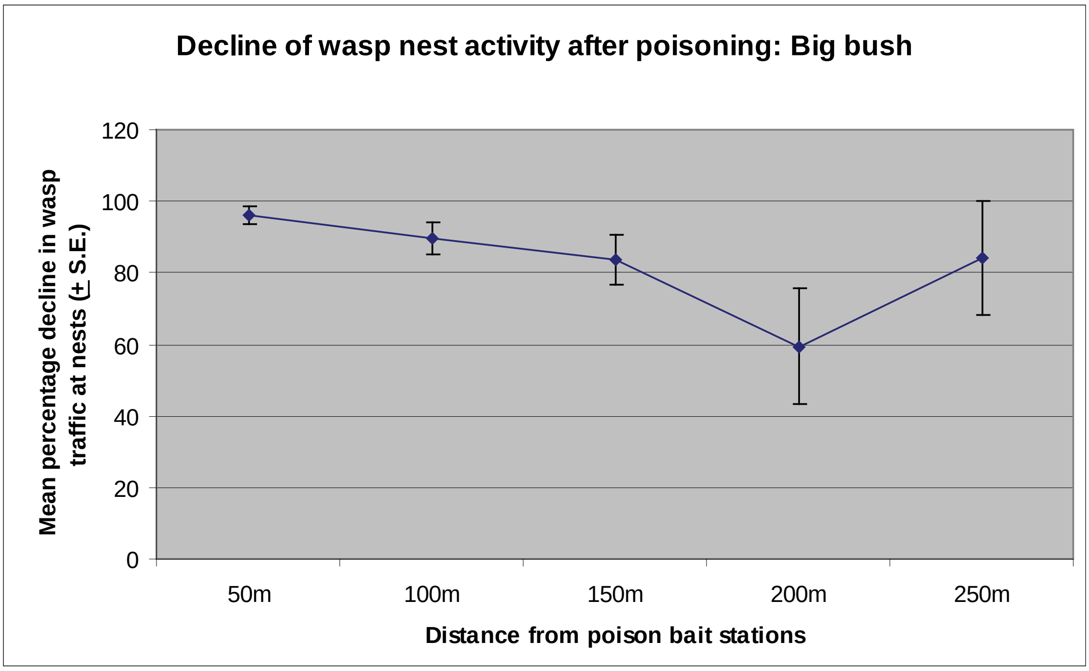

Figure 20c. Decline of wasp nest activity within the C and D bait station clusters

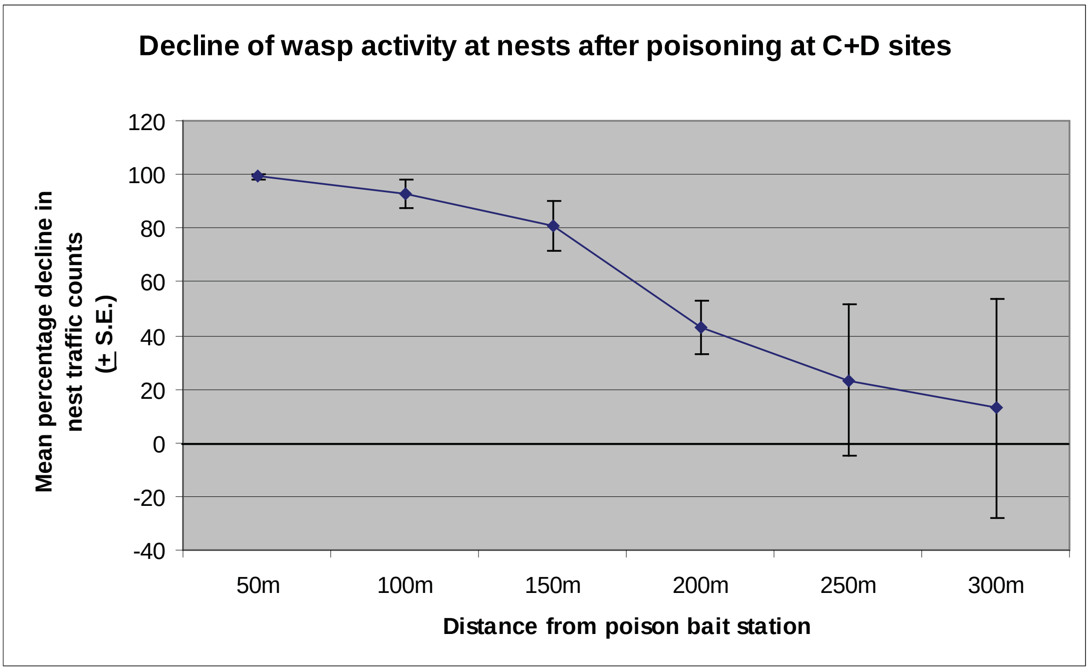

The Big Bush grid appeared to be more successful reducing flight counts of monitored nests even though some nests were monitored out to around 250m from bait stations. Having two bait stations every 100m (instead of one every 50m) has appeared to have had a positive effect, although this needs to be retested.

The combined results from the two clusters are similar to the Big Bush grid with around the same reduction observed out to the 150m mark, after which point results are marginal suggesting the most efficient gird would have 300m line spacing.

For next years operation we will be looking at re-trialling the two grid systems and the two cluster sites again.

The trial looking into potential lures to increase the amount of toxic bait take, will be delayed for a further season. Lures such as fish oil would be applied to freshly baited stations. Previous study into bait preferences found sardine cat-food as the most favoured bait for wasps (Spurr 1995).

Although the high wasp numbers predicted for this season did not eventuate, based on low wasp numbers in the prior season, models suggest that next season much higher wasp numbers are likely.

3.3 TEST THE EFFECTIVENESS OF DIFFERENT TRANSLOCATION METHODS

3.3.1 Introduction

Great spotted kiwi is the only species to have been translocated to the RNRP through wild-to-wild translocations of adults in 2004 and 2006 (Gasson 2005). No work has commenced on introducing any other species. There is more that can be done to test the effectiveness of different translocation methods for great spotted kiwi. Planning commenced in 2008 for future translocations of juvenile great spotted kiwi through an Operation Nest-Egg (ONE) project involving the collection of eggs from Gouland Downs in Kahurangi National Park. Eggs were to be incubated at Willowbank Wildlife Park in Christchurch, and subsequent chicks put on a crèche island. At a weight of >1kg the juveniles were to be released into the Mainland Island. There were two broad objectives for the ONE translocations:

- **Biodiversity objective:** to augment the existing founder population with young birds from another site, and
- **Research objective:** a pilot study to compare the success of ONE birds to the success of RNRP-hatched birds, with respect to territory establishment and breeding success in the RNRP.

A funding application submitted to the Bank of New Zealand Save the Kiwi Trust was approved. The pilot study is relevant due to increasing interest in using ONE to manage existing great spotted kiwi populations and to perhaps establish new populations. The RNRP is a site where the fate of ONE birds can be monitored and compared to wild-raised chicks protected by predator control. The project should indicate whether successful recruitment of ONE birds into an existing population will occur, and if so, whether the process (including dispersal and age of breeding) is broadly similar to wild-raised birds.

Methods

An ONE operation was carried out again this season was used this year in order to supplement the RNRP kiwi population with young individuals. Eighteen kiwi, mainly males, in the Gouland Downs, had been fitted with egg-timer transmitters by the time of the operation in November 2010. Additional transmitters had been fitted to GS kiwi in March 2010 (Harper et al. 2010). Hut wardens on the Heaphy Track monitored the kiwi through spring and passed on the activity information about once a fortnight. When it was assessed that birds or pair activity suggested incubation had begun it was planned to remove eggs after approximately 45-60 days incubation. These eggs were to be flown out to Wakefield and then driven to Willowbank in Christchurch. At Willowbank the eggs were to be incubated and successfully hatched chicks reared until release. After successful release of two chicks into the Mainland Island further releases of chicks were planned directly into the Core Area of the Mainland Island in late March at about 700g.

Results

Hut wardens on the Heaphy Track provided the RNRP team with information from activity transmitters through the spring. This information proved difficult to interpret although indications were that at least three pairs were incubating. Based on the activity information received, the RNRP team went to the Gouland Downs in mid-November.

Of the 16 possible pairs, only two were found to be incubating eggs. Both eggs were removed for ONE on 18 November 2010 (Egg ages: 72 days old and internally pipping, and 45 days old), transferred to Willowbank and subsequently hatched successfully. The resulting chicks were 'hard-released' into the Core Area of the Mainland Island on 20 March 2011 at weights of 690g and 1195g. The first chick was found dead on 4 April, after having become stuck in a small hole. The second larger chick was found dead on 3 May after having wandered over 1.5km from the release site, losing condition en route.

In March 2011, during a trip to Gouland Downs to replace transmitters, another pair were found to be incubating an egg. This egg was removed to Willowbank on 10 March at 62 days old. Unfortunately the chick was assisted in its hatch, did not thrive, and died at two months old having gained very little weight.

Thus far, only six ONE eggs have been moved to Willowbank over two years. Of these six eggs, one has died at Willowbank and of the five that have been released, two are alive in the Mainland Island.

Discussion

It is becoming apparent that the ONE programme has been of limited success for GSK at Rotoiti and mirrors similar problems with ONE and GSK elsewhere, the Paparoas for example. A picture is emerging of a species that is highly social and requires either a lot of parental input, or at least some form of socialisation with conspecifics during the chick stage. We plan to run one last year of ONE from eggs sourced at Gouland Downs this season and then terminate the programme. This is for several reasons, the first being that GSK breeding appears to be limited to every second or third year at most, so in any given year only a few pairs within a population actually breed. Secondly, releases of chicks are having limited success, possibly due to the lack of parental input or socialisation. Thirdly the operation is relatively expensive in time and money and at this stage is highly unlikely to provide the 14 chicks that were planned for in the time period allowed.

Any chicks that result from the planned operation in spring 2011 will be put into a pen within the Core Area for ease of monitoring for about the first 10-14 days and then released.

3.4 DETERMINE LONG-TERM TRENDS IN BIRD ABUNDANCE AND FOREST HEALTH IN RESPONSE TO ONGOING MANAGEMENT

3.4.1 Introduction

The RNRP continues to play an important role in monitoring bird calls and forest health as part of the Department's commitment to measuring long term biodiversity trends. Monitoring of beech seed-fall adds value to the national picture of forest seed-fall and enables the project to plan appropriate management responses.

3.4.2 Five-minute bird counts

Methods

Five-minute bird counts (5MBC) were conducted in November, February and May using the technique detailed by Dawson and Bull (1975). The counts were conducted on the St Arnaud Range Track in the Core Area, at Lakehead and along the Mt Misery Track at Rotoroa. Each site was sampled three times during each month, usually on consecutive days. A total of four different observers were used this year.

Results

The bird count data was entered onto a spreadsheet (RNRP 5MBC). Mean counts were calculated for each bird species at each location for May 2011. These were graphed against the pooled averages since 1997 in RNRP and Lakehead and 2003 in Rotoroa (Appendix 6). The bird count data will be included in the national 5MBC database.

Discussion

A Science Advisory Fund report using five minute bird count data from the RNRP was produced by Stacey Langham with Dave Kelly in 2010. It investigated the effect of wasp, rat and stoat control on the conspicuousness of birds at sites with a combination of these three treatments. There was a positive relationship between bellbird abundance and wasp control and there were also affects on the time-budgets of bellbirds, with increased time spent foraging where wasps were not controlled (i.e. wasps were competing for food). In regard to pest mammal control, the overall trend was for stoat control alone to have a negative impact

on many passerines, likely to due to a related increase in rat abundance, while many bird species benefitted from both stoat and rat control.

3.4.3 Vegetation plot monitoring

Nineteen out of twenty 20x20m vegetation plots within the RNRP have been re-measured from 2009-2011. A few corrections were made to plant identification, particularly Coprosma species and many trees tagged low were re-tagged at breast height. Most plots were measured for the third time (initial measurements took place 1997-1999). Vegetation plots are monitored using the updated field protocols for permanent plots and the RECCE method (Hurst and Allen 2007a, Hurst and Allen 2007b).

3.4.4 Beech seed fall monitoring

Methods

Beech seed fall monitoring is conducted within the Core Area of the Mainland Island and along the Mt Misery Track at the Rotoroa non-treatment site. There are 20 seed fall traps located at each site. The beech seed fall trays were fitted with seed fall collection bags in March. The bags were replaced in mid April and finally removed mid June. The seed was then counted and tested for viability. There was an error found in the calculations for viable seed counts in 2006 so the recalculations have changed that result and are now shown correctly in Figure 21.

Results

There were low beech seed counts at Lake Rotoiti and Lake Rotoroa this year. Fewer than half of all seeds collected were viable, with the exception of red beech at Rotoiti which was at 50%. Mountain beech seed had the lowest viability at both sites with only 10% of seed viable at Rotoroa. Silver beech contributed the most seed at both sites with the Rotoroa count more than double the Rotoiti count. It should be noted that one silver beech tree at one station near lake-level at Rotoroa has biased the seed count significantly over 10 of 16 annual counts. This year that one station accounted for 54% of silver beech seed collected from all twenty stations. During the most significant seed fall recorded for this one station in 2008, this particular tree produced 400 times the average seed fall for silver beech.

TABLE 4. BEECH SEED COUNTS FOR THE MAINLAND ISLAND AND LAKE ROTOROA 2010-11

SITE		NOTFUS	NOTMEN	NOTSOL
Mainland Island	Total count	287	1882	195
	Total viable seed	144	713	35
	Percentage viable	50%	38%	18%
Lake Rotoroa	Total count	502	7497	214
	Total viable seed	129	1914	22
	Percentage viable	26%	26%	10%

Figure 21. Total viable beech seed from the Rotoiti Mainland Island, Big Bush and at Mt Misery, Lake Rotoroa

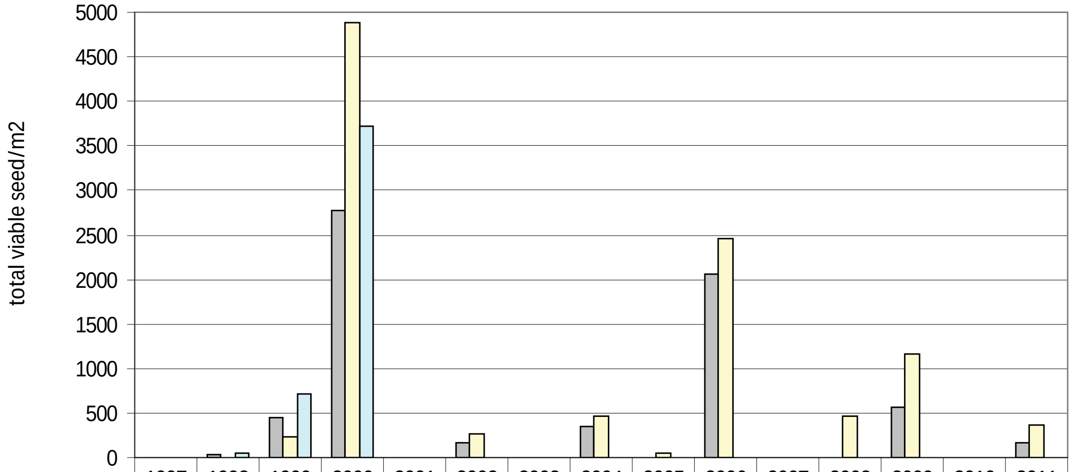

	1997	1998	1999	2000	2001	2002	2003	2004	2005	2006	2007	2008	2009	2010	2011
RNRP	0.36	35.00	447.50	2779.7	0.71	166.43	1.43	345.36	3.21	2059.2	0.36	0.00	558.44	6.79	159.30
Misery	0.00	0.00	225.89	4883.0	1.79	264.29	2.32	465.89	54.46	2461.6	0.54	460.95	1163.8	6.79	368.67
Big Bush	2.78	49.07	715.74	3722.1											

Discussion

There was no beech mast event this year and it has been five years since the last beech mast in 2006. Prior to that mast seed event, there was a six year gap to a heavy beech mast in 2000, so a beech mast event is expected within the next few years. The implications for the RNRP are improved breeding for kaka and parakeets in particular, but also a subsequent increase in rodents and consequently, stoats. Contingency planning for this event is in place, with increased trap checks for mustelid trap lines, and an extra rat control operation planned in the event of a beech mast.

3.4.5 Tussock plot monitoring

The tussock plot monitoring historically carried out at Mt Misery has been reinstated. A new plot was set up near the Misery Bivvy to record tussock flowering in *Chionochloa australis* and *Chionochloa pallens*. Following advice on methodology, a 20x2m plot was permanently marked replacing the old method used. It was recommended that a count be done using the old method in conjunction with the new method next year for comparative analysis (D. Kelly pers. comm.).

Results

There was no flowering in February 2010 before the plot was established but there was some flowering this year in both *C. australis* and *C. pallens*. This information will be added to a national databank which is used to look at tussock seed-mast events.

3.5 SYSTEMATICALLY RECORD OBSERVATIONS OF PREVIOUSLY UNREPORTED NATIVE AND NON-NATIVE ORGANISMS IN RNRP

3.5.1 Introduction

Methods

The systematic recording of previously unreported native and non-native organisms is a new objective identified in the proposed RNRP Strategic Plan 2008-13. The intention of this objective is to maximise the learning from observations of species previously unknown to be present, regardless of whether the observation is part of an organised survey or not. Increased knowledge of the native species present in the RNRP is useful. Detection of invasive plants or animals will inform management actions to protect biodiversity values.

Results

There is currently a repository for new information; 'Flora and fauna of Lake Rotoiti Recovery Project'. No new species were identified during 2010-11.

3.6 FACILITATE RESEARCH TO IMPROVE OUR UNDERSTANDING OF THE ECOLOGY AND MANAGEMENT OF BEECH FOREST AND ALPINE SYSTEMS

3.6.1 Introduction

The RNRP continues to be a place of learning for external researchers. One student review of RNRP five-minute bird counts data was completed this year (Langham 2010).

3.6.2 Research conducted during 2011

Short-tailed bat (Mystacina tuberculata) survey

Only two extant species of endemic bats occur in New Zealand, the lesser short-tailed bat (*Mystacina tuberculata*) and the long-tailed bat (*Chalinolobus tuberculata*). Both species have declined significantly since the arrival of humans, and their distribution is now discontinuous (O'Donnell and Sedgeley 1994).

A survey for short-tailed bats was conducted in late summer 2011 within the Mainland Island to determine whether they were present in the area.

Methods

Digital bat box recorders were placed within the Mainland Island for 14 nights in February 2011. The boxes recorded from 1900hrs to 0700 hrs each night. These were set within the beech forest in areas where large, old red beech trees dominated the forest (preferred short-tailed bat habitat).

Results

The weather was generally fine with warm nights. No bat passes (long-tailed or short-tailed) were detected.

Discussion

No bat passes were recorded in this survey. The survey nights were relatively fine and warm, which were ideal for bat activity. It is highly unlikely that short-tailed bats now exist in the area.

3.7 ANALYSE AND REPORT ON THE EFFECTIVENESS OF MANAGEMENT TECHNIQUES AND ENSURE THAT KNOWLEDGE GAINED IS TRANSFERRED TO THE APPROPRIATE AUDIENCES TO MAXIMISE CONSERVATION GAIN

3.7.1 Introduction

Analysing and communicating technical information about the effectiveness of management techniques is a key learning objective, linking directly to national Mainland Island Principle 2: "Results and outcomes are communicated". The RNRP transfers technical information to target groups through various documents including annual reports, field trial reports, and occasional publications, as well as through presentations to technical audiences and input to periodic workshops and hui. Technical analysis and communications need to be distinguished from advocacy work which is discussed in section 4.1.3., and includes brochures, newsletters and presentations targeted at non-technical groups. In addition there is ongoing interest from the media in work by the RNRP, mainly focusing on the kiwi project.

3.7.2 Reports generated during 2010-11

No reports were produced this year.

3.7.3 Hui, workshops, presentations and media articles

Grant Harper attended a great spotted kiwi hui in August at Arthurs Pass.

The RNRP hosted a great spotted kiwi hui in June 2011.

Several presentations were given to visiting groups.

Media articles included:

13 July 2010	*Nelson Mail:* 'Surprise chick a 'good sign' for kiwi'
13 July 2010	*Marlborough Express:* 'Chick boosts project'
13 July 2010	*Kaikoura Star:* 'Kiwi hatched without help'
13 July 2010	*Guardian (Golden Bay):* 'New kiwi chick something of a surprise'.
13 July 2010	*Stuff website:* New kiwi chick article.
20 July 2010	*Fresh Choice radio station:* Interview Nik Joice
9 August 2010	*Nelson Mail:* 'Rescue Helicopter's work stops for no MP'
28 September 2010	*Radio New Zealand:* Interview regarding Takaka's death
26 October 2010	*Nelson Mail:* 'DOC vows tough line of dogs in kiwi area'
10 January 2011	*Fresh Choice radio station:* Interview Grant Harper
8 February 2011	*Nelson Mail:* 'Few kea on way out here'
6 June 2011	*Nelson Mail:* 'Beech forest wasps causing unfolding ecological disaster'

4. Community objectives

4.1 INCREASE PUBLIC KNOWLEDGE, UNDERSTANDING AND SUPPORT FOR MAINLAND ISLANDS AND ECOLOGICAL RESTORATION NATIONALLY THROUGH EDUCATION, EXPERIENCE AND PARTICIPATION

4.1.1 Introduction

This year has continued to present more opportunities than in previous years to meet the project's community objectives, focussing on getting students and visitors out into the RNRP for guided walks and talks within the project, rather than a powerpoint presentation indoors. Most walks and talks are provided as part of the educational programme for the Rotoiti Lodge Outdoor Education Centre. This year saw a small decrease in the total number of participants, with 1593 gaining knowledge about mainland islands and specifically the RNRP.

The local conservation volunteer group, Friends of Rotoiti (FOR), have again expanded their trapping range with another stoat line. Additional assistance has been provided to the project by visiting individual volunteers and groups, who have gained valuable restoration experience (trapping, monitoring etc). Promotion of the RNRP continues through two editions of the *Revive Rotoiti* newsletter published this year. New interpretive displays have been installed in the Nelson Lakes National Park Visitor Centre, highlighting the project. Both the Murchison and Lake Rotoiti Community newsletters have had articles about work being done in the RNRP.

4.1.2 Friends of Rotoiti

In response to the RNRP being formed in the late 1990s, members of the local community came forward, wanting to assist with the efforts of the project. A partnership with this community group was formed in 2001. This dedicated group maintains trapping lines for pest control (mustelids, rodents, possums and wasps) and have the opportunity to get involved in species monitoring and re-introductions. During the year, training meetings are held to train members in best practice techniques.

This is the second year of the Supporters Group, providing financial assistance for FOR conservation work and the RNRP, with donations and sponsorships. Supporter funds received were $3,330 (to June 2011), compared to $3,000 for the same period last year.

A number of options were provided to supporters this year, as follows:

- $20 membership (37 memberships = $740)
- $55 Feed 'Fen' the kiwi dog ($165 received from 3 members)
- $75 Peanut butter and rolled oats for rat trapping
- $100 Eggs for stoat trapping ($200 received from 2 members)
- $200 Meat baits for stoat trapping ($400 received from 2 members)
- $400 Great spotted kiwi transmitter ($400 received from 1 member)
- General donation ($1,425 received)

There are 51 active volunteers, who look after the mustelid, rodent, possum trapping and wasp poisoning, and trap maintenance. Training is an important aspect for the involved

volunteers, who are expected to stay up-to-date with the groups activities and to attend at least two training meetings a year. The group contributed 248 workday equivalents (one work day equals six hours) this year.

FOR wasp control

A small number of the group operate 'Wasp Busters' to help locals control wasp nests around their homes. This service is done each summer on a donation basis, using the contact insecticide *Permex*.

Wasp control is also carried out in January/February along the Whisky Falls line along the western shore of Lake Rotoiti.

FOR rat control

Since December 2001, the FOR have been operating a rat control programme over 250 hectares adjacent to the St Arnaud township (Brunner Peninsula, Black Hill and Black Valley stream areas). Corflute tunnels house Victor Professional rat traps, baited with a peanut butter and rolled oats mix. Fortnightly checks were carried out throughout the year. A total of 671 rodents were trapped (Table 5). The 'by-catch' was one stoat, two weasels and seven introduced birds (one chaffinch, six sparrows). The additional 41 traps on the Peninsula Nature Walk have remained set for the whole year, just in case the mouse numbers were as high again as 2009/10.

TABLE 5. FRIENDS OF ROTOITI RAT TRAPPING RESULTS FOR THE PAST FOUR YEARS

	RODENT CAPTURES	
YEAR	RATS	MICE
2007/08	112	422
2008/09	113	446
2009/10	116	4343
2010/11	148	523

FOR possum control

Due to possum interference with stoat traps, FOR started possum trapping in 2004, setting up warrior traps adjacent to the affected stoat boxes. All warrior traps have now been changed to sentinels on the FOR lines. This reflects the change made by the RNRP from warrior traps to sentinels last year. A total of 30 sentinel traps are now operating and trapped 53 possums this year (Figure 22).

FOR lizard monitoring

From the summer of 2002-03 to 2008-09, FOR operated a lizard pitfall trapping programme for identifying lizard species present in the FOR rat control area. This programme was set up for FOR volunteers to gain experience with species monitoring and to identify population trends. This monitoring has been put on hold this summer to allow a student to use the pitfall traps as part of their research into the effect of introduced mammalian predators on indigenous skink populations.

Figure 22. Friends of Rotoiti possum trapping results May 2004 – June 2011. (Figures in brackets are the number of traps on each trap line.)

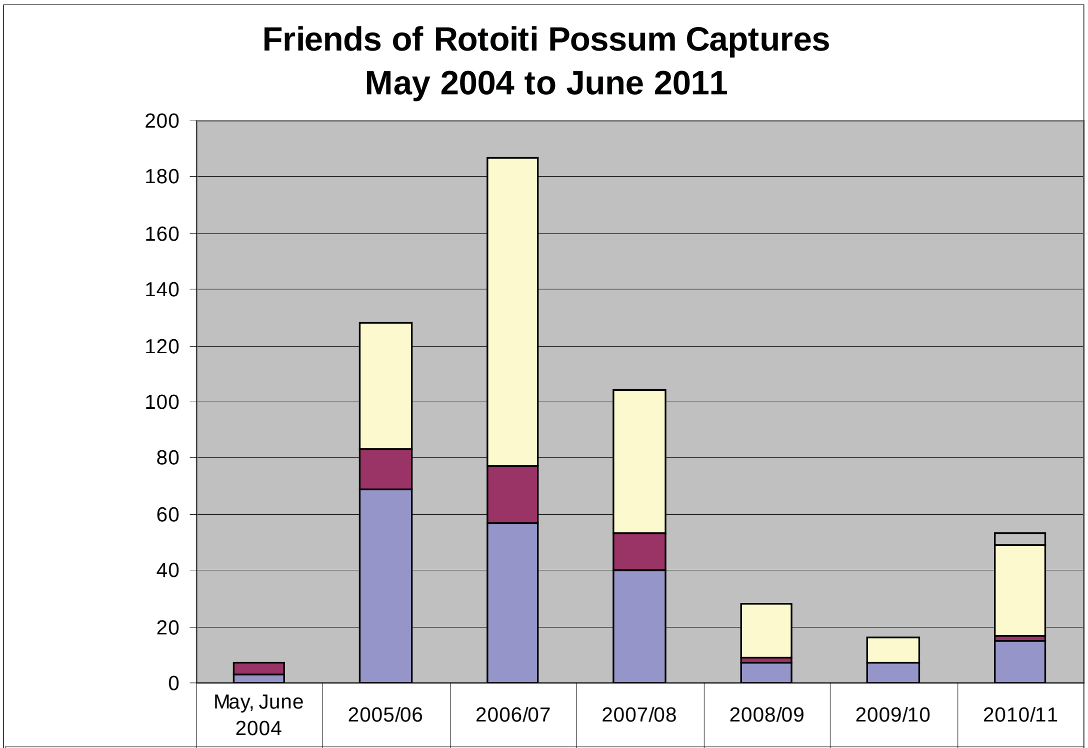

	May, June 2004	2005/06	2006/07	2007/08	2008/09	2009/10	2010/11
Speargrass (2)							4
Whisky Falls (16)		45	110	51	19	9	32
Mt Robert (2)	4	14	20	13	2	0	2
Rainbow (10)	3	69	57	40	7	7	15

4.1.3 Volunteers

The RNRP continues to provide opportunities for volunteers to learn about restoration work by hosting two general RNRP volunteers (one international, one New Zealander) and 12 trainee rangers, working the equivalent of 49 days. The trainee rangers (from Nelson Marlborough Institute of Technology) participated in the RNRP kiwi call monitoring programme in March 2011. This programme is run as part of the BNZ Save the Kiwi partnership. Note that these volunteer hours do not include those contributed by the Friends of Rotoiti.

4.1.4 Advocacy and education

Revive Rotoiti

Two publications of this six monthly newsletter *Revive Rotoiti* were published in Spring 2010 (Issue 23) and Autumn 2011 (Issue 24). The latest edition features a completely different template, in line with the Department's new identity guidelines for publications. All editions are available online on the DOC website. A hard copy mailout was done to main stakeholders, who were asked if they would prefer to go on the email list, to reduce costs and environmental impacts. Copies of these newsletters are also available at the Nelson Lakes Visitor Centre and the Nelson Regional Visitor Centre.

Media releases and other advocacy work

Media Releases

- 13 July 2010: New kiwi chick found in the RNRP by our kiwi dog Fen.
- 28 September 2010: Adult male kiwi, Takaka, found dead due to predation.
- 12 Jan 2011: Sixth kiwi chick born in the RNRP, found dead.

Both the Lake Rototiti Community Newsletter and Murchison Newsletter have had articles submitted. The DOC webpage has several RNRP pages, and these are linked to the project's annual reports, strategic plan and newsletters.

Promotion continued this year at the Murchison A & P Show (February 2011) and the Antique and Classic Boat Show (March 2011). The Power Boat Show, usually held in February was cancelled this year due to lack of entries. Updates were given at the Rotoiti District Community Council meetings and Nelson Community forums.

Visitor Services

The new back-lit interpretive displays, featuring the RNRP, welcome visitors to the Nelson Lakes National Park Visitor Centre. These displays were opened in September 2010. For those wishing to explore the project, the Bellbird and Honeydew Walks provide interpretation panels as educational tools and to broaden the experience for visitors.

Rotoiti Lodge Outdoor Education Centre – RNRP presentations

Reflecting the change made last year from inside presentations to outdoor walks, this year the RNRP PowerPoint was presented 20 times to 770 students staying at Rotoiti Lodge. These students included Year 12 and 13 students who were learning about conservation and resource management as part of their National Certificate Education Achievement unit standard.

RNRP guided walks and other presentations

The number of RNRP guided walks this year remained high, with 680 students and visitors undertaking 45 guided RNRP walks. The focus of the walks provides a more personal 'hands on' experience for the students to see and hear about the bird life, traps and bait stations in situ. In addition, there were another six RNRP/Nelson Lakes National Park PowerPoint presentations to other groups not staying at Rotoiti Lodge (to 143 participants).

5. Discussion

DEMONSTARTING THE VALUE OF THE RNRP

The 'new' RNRP team has now been present for a year or more and are now well versed with the programme requirements and are beginning to put their own stamp on it. The normal day to day or seasonal activities like trapping and monitoring are now a little more mundane, but recent publications have shown the value in this work. The RNRP is the repository for some very valuable long-term data sets, in particular the 5-minute bird counts and beech seed fall counts. These data sets are now starting to being used to provide insights into the long-term trends and changes occurring in New Zealand ecosystems and the drivers for these changes, be it climate change or predator pressure. Other data collected for the past decade is also likely to provide observations that will increase our understanding of montane beech forests. As one of the principal reasons for the establishment of the RNRP is to learn and then disseminate the finding of the research there is a need to publish the results of the past 10 years work.

Most of the work the RNRP does has an experimental component in an effort to improve the way we manage beech forest ecosystems. The recent rat control operation provided us with a beginning, using diphacinone on a relatively large scale to knock-down a rat population. This was successful and the operation has been expanded along the lake shore. The real test will be managing rats through a mast-seed year which should occur within a year or two. If rats can be kept at low numbers through this event then we can begin planning for the 'restoration' part of the RNRP, and look to reintroductions of species that have been absent for some 50-100 years.

Other work that is providing information for other practitioners is the current work on weka, investigating why the population is not thriving here as yet, wasp control work to investigate how best to control them in the most efficacious manner and measure the benefits for native fauna, and building up a picture about great spotted kiwi and gathering information on a species about which surprisingly little is known.

In addition to this work, the 'learning' part of the RNRP work has rubbed off on the local community group, the Friends of Rotoiti (FOR), has investigated wasp control and better lures. As they come to the end of their 10th year working with the RNRP they are continuing this theme and will be doing further trials on mustelid baits in future. The RNRP is also due to host a portion of a nationwide trial by DOC on self-resetting traps to control mustelids. The reason that the Mainland Island was chosen was the long-term data we hold on mustelid numbers at Rotoiti and Rotoroa, the monitoring that is done and the infrastructure to support the project. This is another indication that the work the RNRP does is valued not just by the local community but by conservation and restoration practitioners, managers and scientists nationwide.

6. Recommendations

- Discontinue use of Erayz until the bait has been reformulated to remove mice/mould problem.
- That FOR trial a mustelid polymer fish-based bait as a stoat lure in consultation with RNRP staff.
- Trial different mouse-proof holders to determine an effective means of excluding mice from polymer baits within trap tunnels.
- Investigate the attractiveness of the polymer baits over time in relation to mould and insect damage.
- Investigate methods to trap ferrets without trapping weka.
- Investigate effective cat traps that also exclude weka.
- Continue with weka monitoring in present form.
- Continue to monitor kiwi nesting attempts and take feather samples for DNA matching with possible related individuals, especially parents.
- Continue with the spring rat poison operation, and finish expanding the rat control grid to about 900ha.
- Trial fish-oil lures for wasp bait stations.
- Further investigate effects of distance to wasp bait stations on wasp control and their impacts on native fauna.
- Finish the GSK ONE programme in Gouland Downs but continue monitoring of nesting attempts in the RNRP.

7. Acknowledgements

The Rotoiti Nature Recovery Project relies on support from fieldworkers, volunteers, technical staff and experts.

Thanks to the seasonal fieldworkers, namely Ruth Garland and Katrina Hale. Other staff at Nelson Lakes Area Office also assisted the programme on occasions with shared logistics and costs. Several Nelson Conservancy staff assisted with putting out bait for the rat control operation.

The Trainee Rangers assisted with Kiwi Call Counts and Pacific Discovery Volunteers assisted with work around the Mainland Island.

The Royal New Zealand Airforce, No. 6 Squadron, got the old bivvy off Parachute Rocks. Matt Gibb of Helicharter Nelson also got us and kiwi eggs in and out of Gouland Downs.

Richard Toft of Entecol provided us with valuable advice and assistance relating to wasp control yet again.

Members of the Technical Advisory Group and external advisors provided advice at various times during the year (membership in Appendix 5).

Geraldine Moore provided a lot of assistance with mapping requests, and Trish Grant and Charmayne King helped with getting this report to print and with publicity for the project.

8. References cited

Bell, B.D. 1986. The conservation status of New Zealand Wildlife. Occasional Publication No. 12. New Zealand Wildlife Service, Department of Internal Affairs.

Brow, A. K., Bruce, T. A., Chisnall, D., Gasson, P. A., Leggett, S. A., Paton, B. R., Hawes, M. 2008. *Rotoiti Nature Recovery Project Annual Report 2006-07*. Department of Conservation, Nelson. Occasional Publication No. 73.

Brow, A., Bruce, T, Forder, S., Carter, P., Chisnall, D., Rees, D., Harper, G. 2010. *Rotoiti Nature Recovery Project Annual Report 2008-09*. Department of Conservation, Nelson. Occasional Publication No. 83.

Brown, K. P. & Gasson, P. A. 2008. *Rotoiti Nature Recovery Project. Strategic Plan 2008-2013*. Department of Conservation, Nelson.

Dawson, D. G. & Bull, P. C. 1975. Counting birds in New Zealand forests. Notornis 22: 101-109.

Etheridge, N. & R.G. Powlesland. 2001. High productivity and nesting success of South Island robins (*Petroica australis australis*) following predator control at St Arnaud. Notornis 48: 179-180.

Gasson, P. A. 2005. Translocation of great spotted kiwi/roa (*Apteryx haasti*) to Rotoiti Nature Recovery Project. Occasional Publication No. 67.

Gillies, C. and Williams D. 2004. Using tracking tunnels to monitor rodents and other small mammals. Unpublished report, Northern Regional Office, Department of Conservation, Hamilton.

Greene, T. C., Powlesland, R. G., Dilks, P. J., Moran, L. 1994. Research summary and options for conservation of kaka (*Nestor meridionalis*). DOC Science Internal Series 178.

Harper, G., Forder, S., Henderson, J., Joice, N., Carter, P., Chisnall, D., Steffens, K., Rees, D. 2010. *Rotoiti Nature RecoveryProject Annual Report 2009-10*. Department of Conservation, Nelson. Occasional Publication No. 86.

Hurst, J. M & Allen R. B. 2007a. A permanent plot method for monitoring indigenous forests. Landcare Research, Lincoln

Hurst, J. M & Allen R. B. 2007b. The RECCE method for describing New Zealand vegetation. Landcare Research, Lincoln

Elliott, G. & Kemp, J. 1999. Conservation ecology of kea (*Nestor notabilis*) . WWF-NZ, Final Report.

Langham, S.A. 2010. Effect of wasp control on bird populations at Nelson Lakes National Park, New Zealand. Unpublished Honours Dissertation. University of Canterbury, Christchurch.

Miskelly, C. M, Dowding J. E., Elliott G. P., Hitchmough, R. A., Powlesland R. G., Robertson H. A., Sagar P. M., Scofield R. P., Taylor, G. A. 2008 Conservation status of New Zealand birds, 2008. *Notornis 55:* 117-135

Moorhouse, R. Greene, T., Dilks, P., Powlesland, R., Moran, L., Taylor, G., Jones, A., Knegtmans, J., Wills, D., Pryde, M., Fraser, I., August, A., August, C. 2003. Control of introduced predators improves kaka *Nestor meridionalis* breeding success: reversing the decline of a threatened New Zealand parrot. *Biological Conservation 110:* 33-44.

O'Donnell, C. & Sedgely, J. 1994. An automatic monitoring system for recording bat activity. Department of Conservation Technical Series No. 5. Wellington.

Spurr, E.B. 1995. Protein bait preferences of wasps (Vespula vulgaris and V. germanica) at Mt Thomas, Canterbury, New Zealand. *New Zealand Journal of Zoology 22:* 281-289.

Steffens, K., Gasson, P. 2009. A history of threatened fauna in Nelson Lakes Area. Department of Conservation, Nelson. Occasional Publication No. 81.

Appendix 1

RNRP DATASETS

Data sets referred to in the report, and others that were maintained during the 2009-2010 year are listed below:

INTRODUCED SPECIES

DATA SET DESCRIPTION	FILE NAME AND LOCATION	CONTACT PERSON
Wasp bait stations	dme://docdm-612441/	Nik Joice
Possum captures	dme://docdm-516760/	Dan Chisnall
Possum monitoring results	dme://docdm-458218/	Dan Chisnall
Rodent tracking tunnel results	dme://docdm-621366/	Nik Joice
Mustelid captures	RNRP stoat database 09/10, docdm-788735	John Henderson
Mustelid tracking tunnel results	dme://docdm-621366/	John Henderson
Ungulate sightings	dme://docdm-148952/	Grant Harper

NATIVE SPECIES

DATA SET DESCRIPTION	FILE NAME AND LOCATION	CONTACT PERSON
20 x 20 vegetation plots	Vegetation Plot Ring binder in RNRP office	Nik Joice
Beech seed fall monitoring	dme://docdm-60998/	Nik Joice
Mistletoe monitoring results	dme://docdm-72306/	Sarah Forder
Pittosporum patulum monitoring results	dme://docdm-199798/	Nik Joice
Powelliphanta monitoring results	dme://docdm-546239/	Nik Joice
Great spotted kiwi monitoring	dme://docdm-156428/	Sarah Forder
Robin (*Petroica australis*) monitoring	dme://docdm-459805/	Grant Harper
Kaka (*Nestor meridionalis*) monitoring	dme://docdm-171970/	Nik Joice
5-minute bird counts	5 minute bird count data, docdm-769826	John Henderson

Appendix 2

RNRP REPORTS GENERATED

Nil

Appendix 3

PROJECT REVIEWS

REVIEW DATE	REVIEW TITLE	FILE NAME AND LOCATION
NIL		

Appendix 4

RESEARCH REPORTS RECEIVED

Langham, S.A. 2010. Effect of wasp control on bird populations at Nelson Lakes National Park, New Zealand. Unpublished Honours Dissertation. University of Canterbury, Christchurch.

Appendix 5

PROJECT MANAGEMENT

Budget

Staff (Salary & wages):	$195,751 [excluding volunteer hours]
Operating:	$33,450
BNZ Save the Kiwi	$12,100
**Total**	**$241,301**

Staffing

Grant Harper, Nik Joice, John Henderson, Sarah Forder, Ruth Garland, Katrina Hale, Dave Rees, Dan Chisnall, Akira Doura.

Technical Advisory Group (TAG)

Kerry Brown, Peter Gaze, Craig Gillies, Grant Harper, Mike Hawes, Martin Heine, Dave Rees, Alison Rothschild.

RNRP Advisors

Mick Clout, Graeme Elliott, Dave Kelly.

Appendix 6

BIRD COUNT GRAPHS

Bellbird

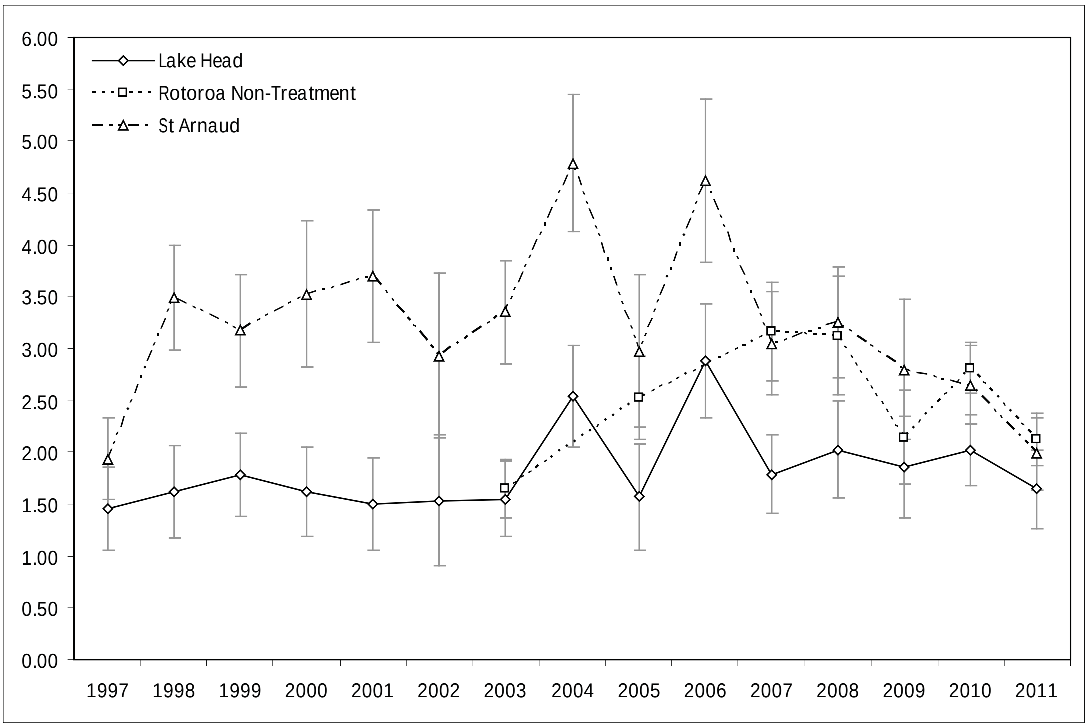

Tui

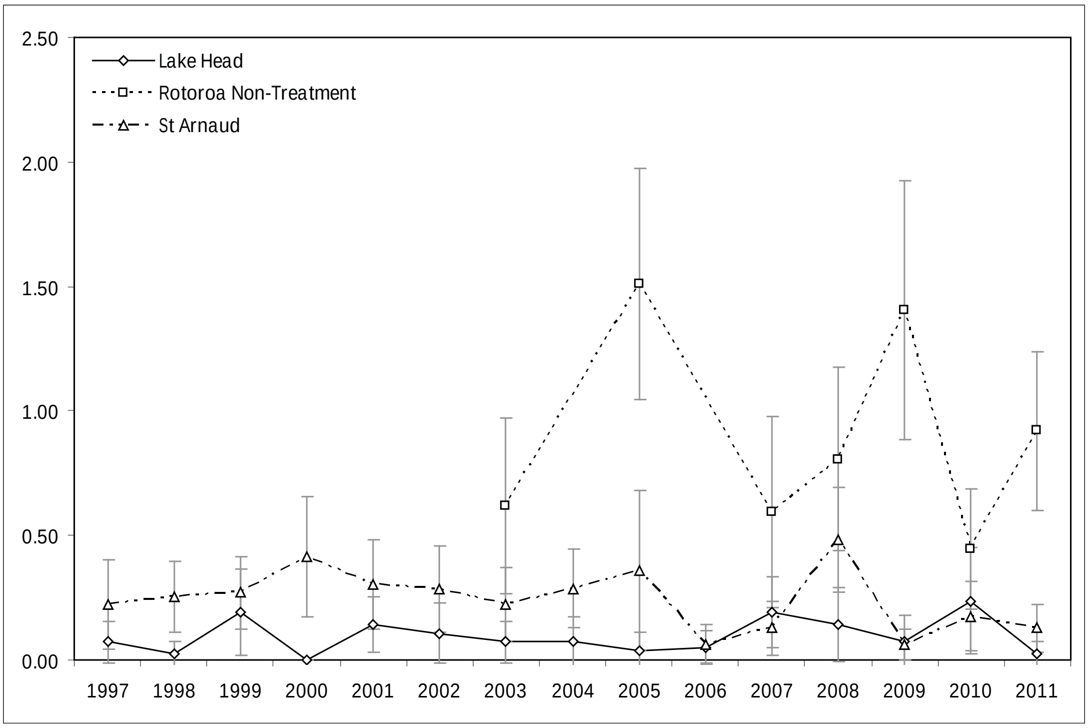

Pied tit

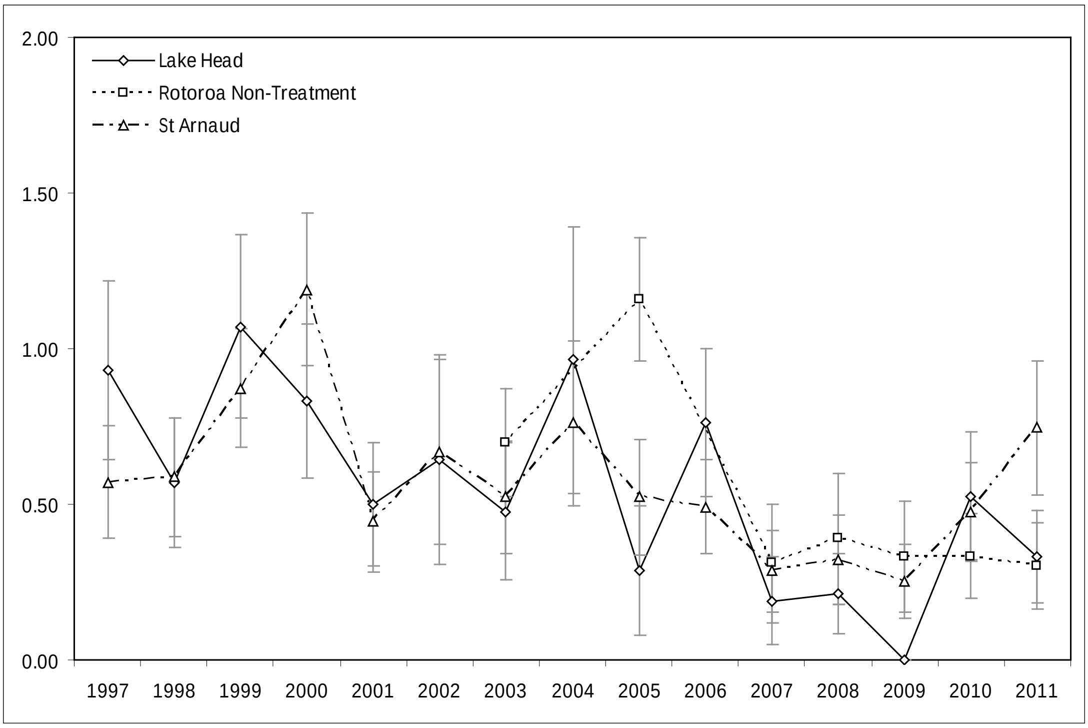

South Island
Robin

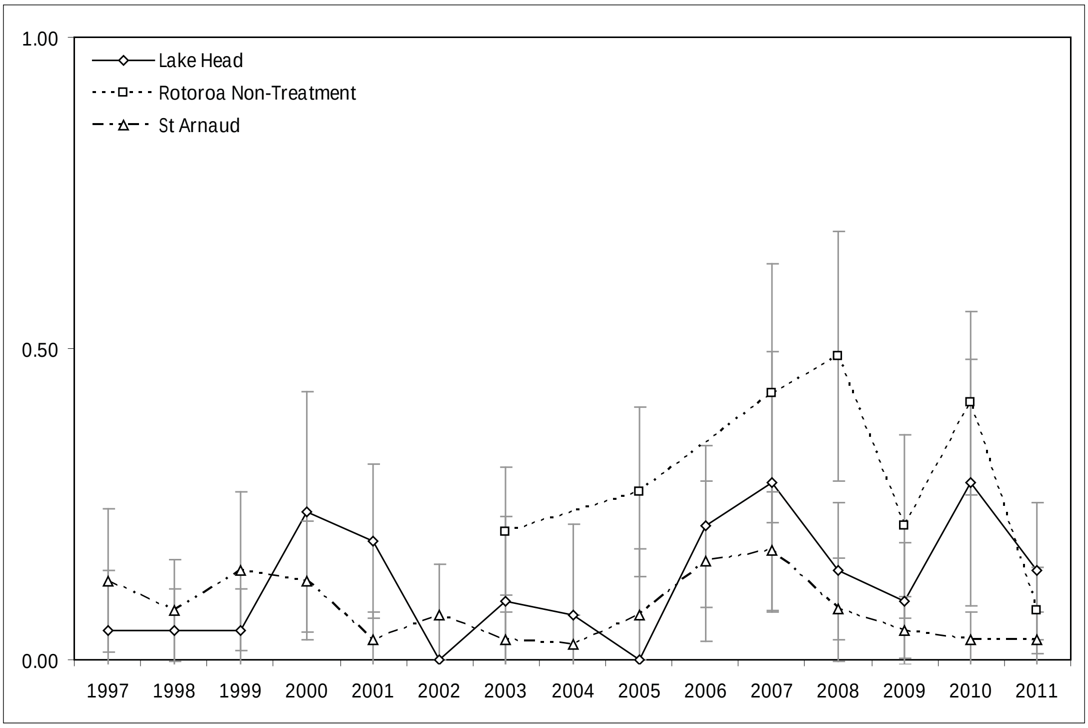

Brown creeper

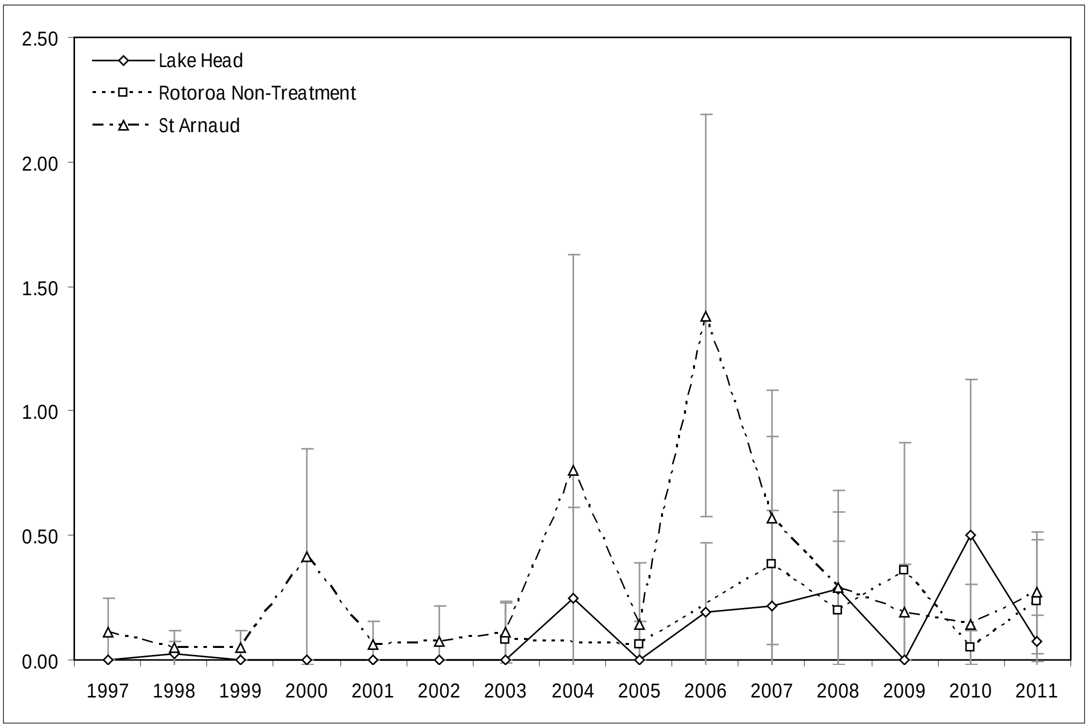

Grey warbler

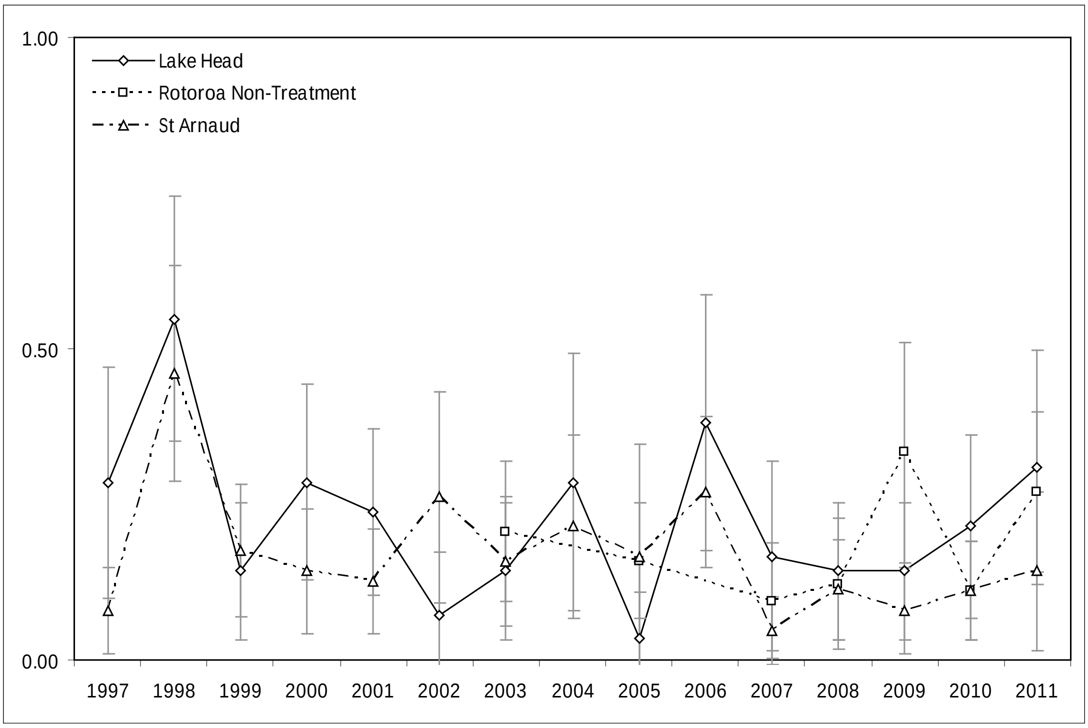

Rifleman

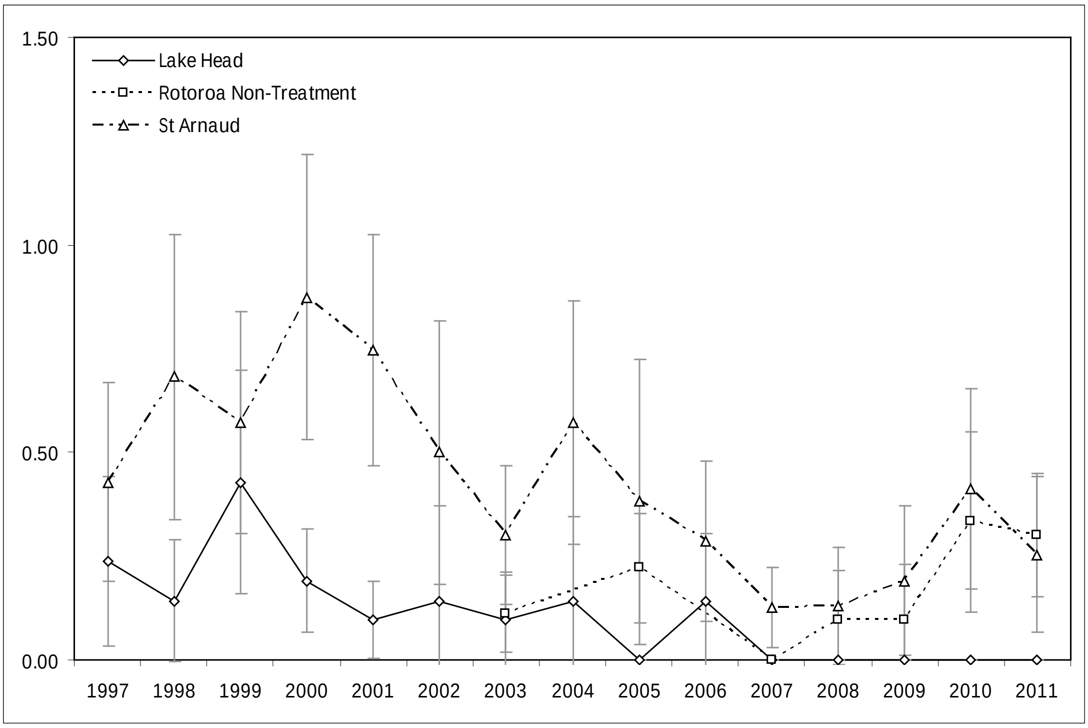

Fantail

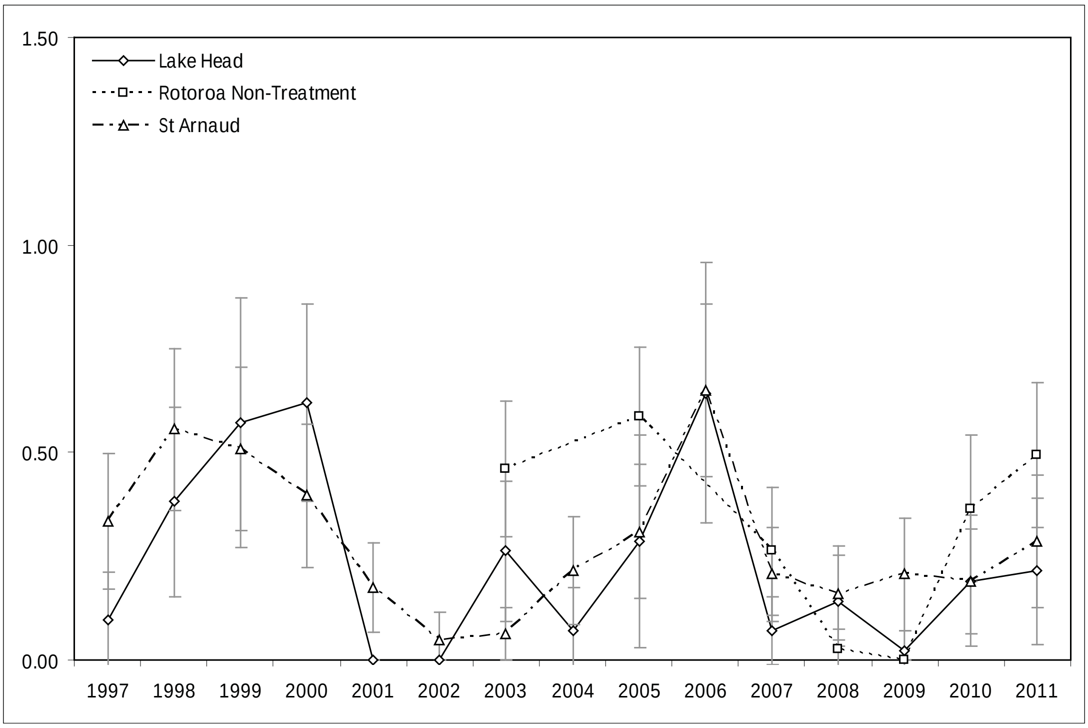

Kaka

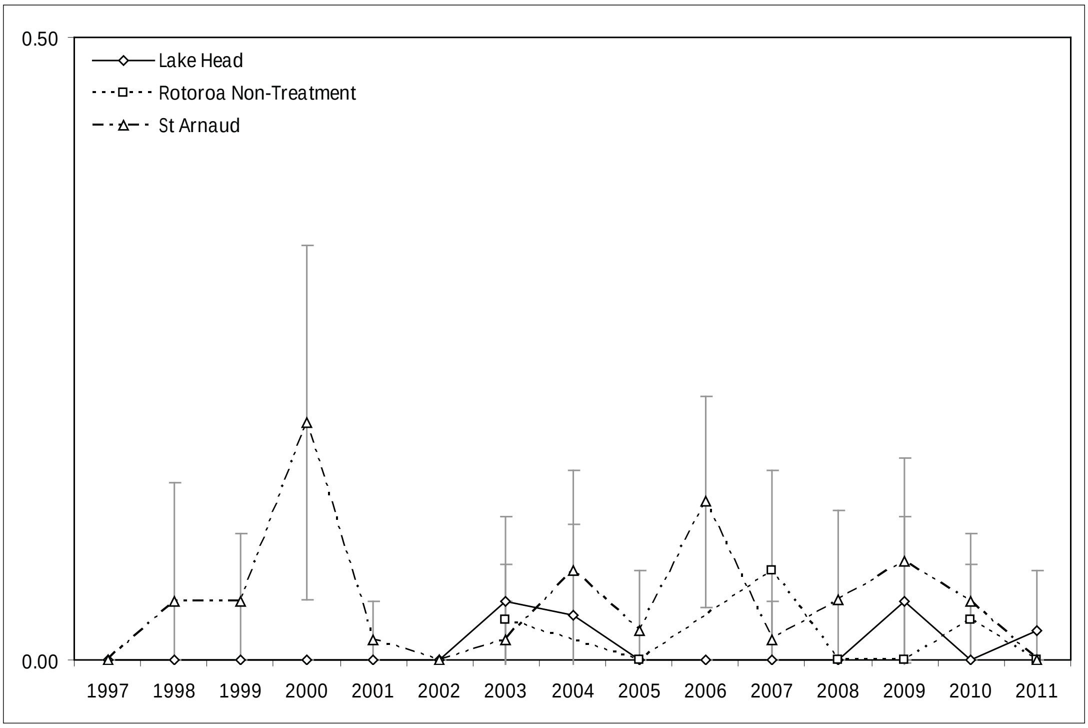

Yellow-crowned parakeet

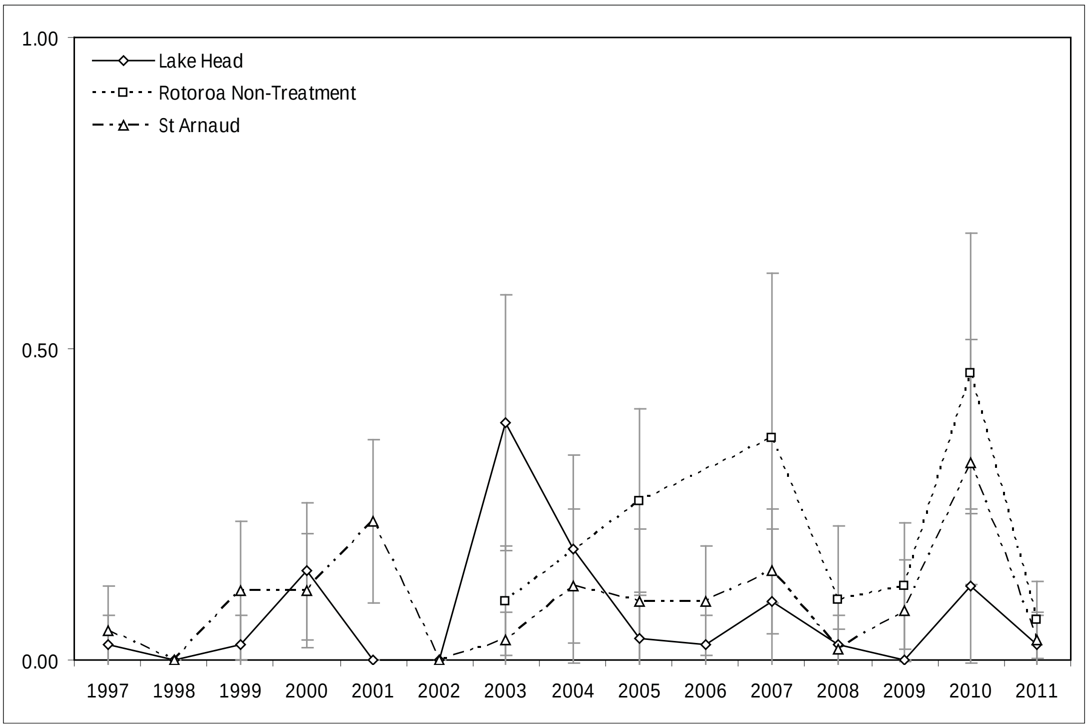

Blackbird

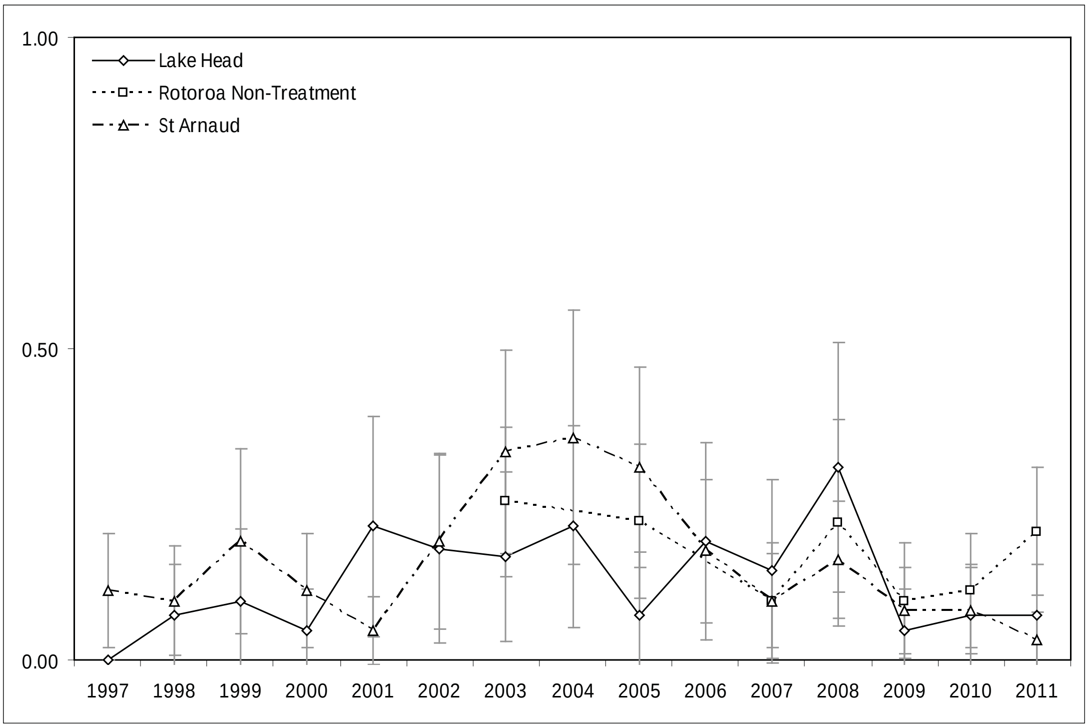

Chaffinch

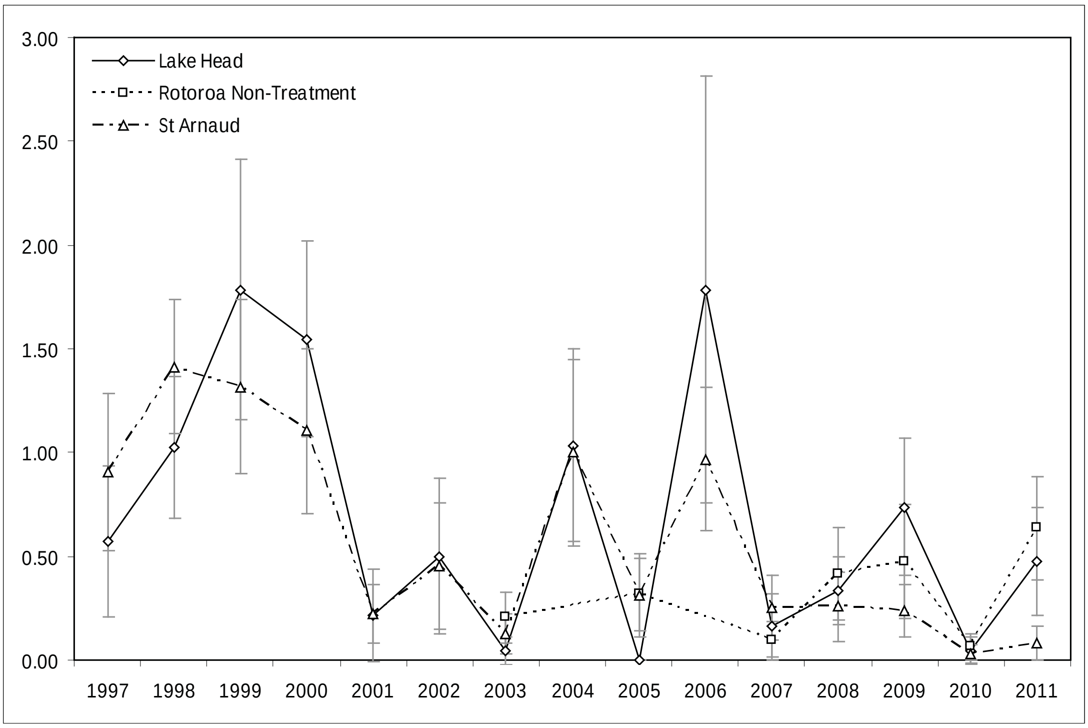

Supplement: Supplementary file 6 — Supporting Material [file COBI-35-35-s005.pdf]
