# Supplementary material for: Responses of New Zealand forest birds to management of introduced mammals
Source: Conserv Biol. 2020 Mar 23;35(1):35–49. doi: 10.1111/cobi.13456 (PMC7984369; doi:10.1111/cobi.13456)
Supplement: Supplementary file 7 — Supporting Material [file COBI-35-35-s006.pdf]

Mt Pihanga – Rotopounamu Restoration Project

Annual Report
Year 9
2011-2012

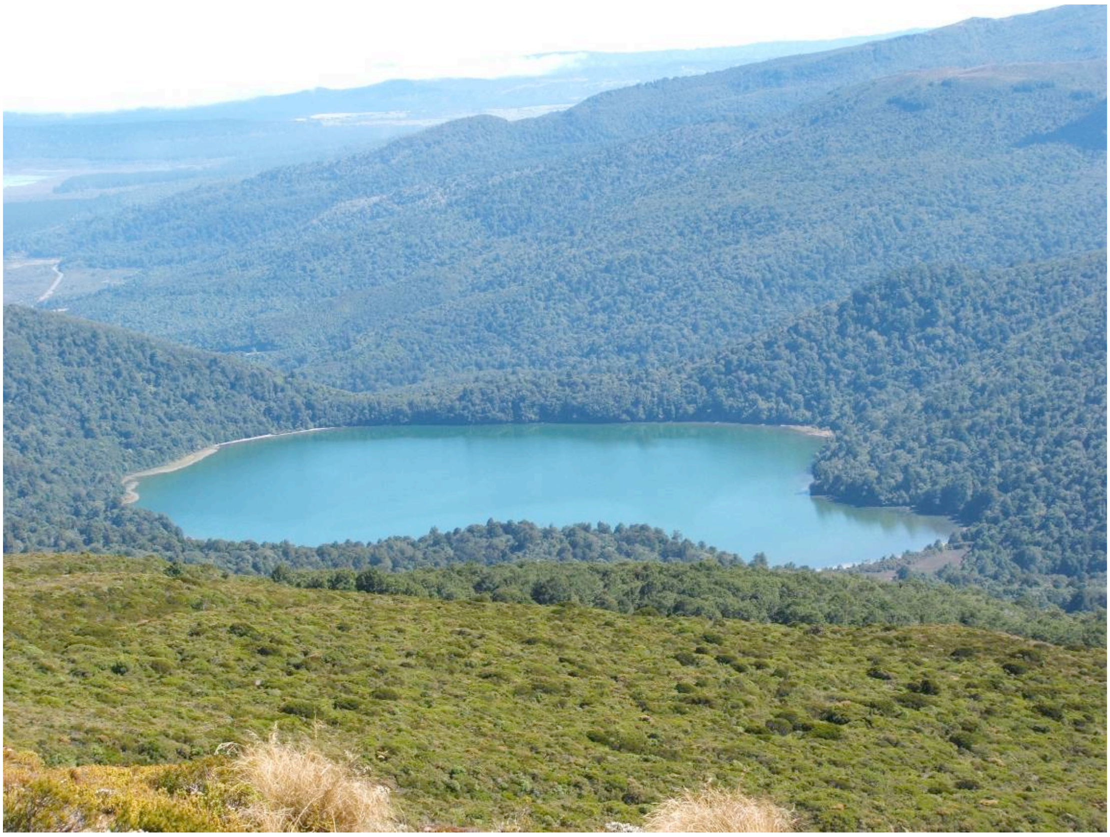

PROJECT TONGARIRO
TONGARIRO NATURAL HISTORY SOCIETY

Prepared by:
Ian McNickle
Biodiversity Ranger,
Taupo-nui-a-Tia Area office
Department of Conservation

Cover photo: Rotopounamu. Photo: I McNickle

Table of Contents

1.0	Summary	5
2.0	Introduction	6-7
3.0	Report against objectives	8-17
3.1	Objective 1	8
3.1.1	Management Recommendations - Objective 1	8
3.2	Objective 2	8-9
3.2.1	Method	9-10
3.2.2	Results/Discussion	10-13
3.2.3	Management Recommendations - Objective 2	13
3.3	Objective 3	14
3.3.1	Management Recommendations - Objective 3	14
3.4	Objective 4	14
3.4.1	Method	15
3.4.2	Results/Discussion	15
3.4.3	Management Recommendations - Objective 4	15
3.5	Objective 5	15-16
3.5.1	Management Recommendations - Objective 5	16
3.6	Objective 6	16
3.6.1	Method	16-17
3.6.2	Management Recommendations - Objective 6	17
3.7	Objectives 7, 8 and 9	17
4.0	Community Relations, Education and Awareness	18-21
4.1	Adopt a Hectare	18
4.1.2	Visitor Numbers	18-19
4.1.3	Predator Traps	19-21
4.1.4	Management Recommendations for Community Relations	21
5.0	Staff and Financial Contribution	22
6.0	Summary of Recommendations	22-25
6.1	General	22
6.2	Objective 1	22
6.3	Objective 2	23
6.4	Objective 3	23
6.5	Objective 4	23-24

6.6	Objective 5		24
6.7	Objective 6		24
6.8	Community Awareness and Education		25
7.0	Acknowledgements		26
8.0	References		26
9.0	Appendices		27-37
	Appendix 1	Contractor Details and Costs	27
	Appendix 2	Pest Bait Details	28
	Appendix 3	Bait Station Layout	29
	Appendix 4	Pest Plant Locations	30
	Appendix 5	Tracking Tunnel Line Locations	31
	Appendix 6	Wax Tag Line Locations	32
	Appendix 7	Five Minute Bird Count Locations	33
	Appendix 8	Summary of Expenses Incurred 2011/12	34
	Appendix 9	Five Minute Bird Count Recording Sheet	35-36
	Appendix 10	Project Timeline	37

1.0 Summary

The Mt Pihanga/ Rotopounamu project was established in 2003 and is community focused with integrated pest management and advocacy at its core. The project is managed according to the Rotopounamu Management Plan (2008), which aims to maintain, enhance (or re-establish) vulnerable habitats and ecosystems and their component fauna and flora; and to prevent the establishment of new threats. To achieve this, it lists nine biodiversity objectives. The purpose of this document is to report progress made against these objectives and make recommendations for the future.

Diphacinone, 0.05g/kg, Pest Off cereal pellets was the chosen bait again this year for the rodent control operation having proven very successful in the 2010/11 season. The bait has once again controlled rats to target levels through most of the bird breeding season this year. The rodent control programme had some challenges with a number of trees coming down and pigs deciding the bait in the bait stations was a good source of food. Five minute bird counts were undertaken again by DOC staff and Project Tongariro Interns, with a total of 248 counts completed. Significant increases in rifleman, silvereye and whitehead numbers were seen, showing that they have experienced another good breeding season. The kakariki population continues to steadily increase and Rotopounamu is proving to be a stronghold for this species.

Wax tags were used to measure possum abundance within the 530ha management block, and the equivalent of less than 2% RTCI was recorded. Therefore no possum management was undertaken this year. Project Tongariro volunteers continued to check the 50 traps set around the walking track, killing 19 stoats and 79 rats.

Due to a reduction in staff numbers for the year, no threatened species monitoring was undertaken, although these surveys were identified as ideal Project Tongariro intern projects. Similarly, no weed control was undertaken along the lake margins, although heather was targeted on the Pihanga summit.

Project Tongariro members and interns put a lot of effort in to the Rotopounamu project volunteering a lot of time running tracking tunnels, checking and clearing traps and anything else that was asked of them. Their skill bases are expanding every year and their friendly faces are great advocates for Project Tongariro and conservation in general while carrying out the work.

The “Adopt a Hectare” initiative is steadily building in momentum with 60 hectares adopted by the community so far. The Rotopounamu walk is a great attraction to travelers and the local community with more than 11,000 visitors per annum. The Rotopounamu project also provides a great advocacy tool for demonstrating predator control techniques to the public.

Overall the 2011/12 season was a great success and the future looks positive for the Rotopounamu Project. The 2012/13 season will see another great year of Project Tongariro working with the Department of Conservation to restore the ecosystem of the MT Pihanga – Rotopounamu area.

2.0 Introduction

Rotopounamu, on the slopes of Mt Pihanga in Tongariro National Park, is the focal point of a long term project to protect the native biodiversity of this area. The site has high conservation value, as a part of Tongariro National Park. Tongariro National Park is amongst a few sites that have dual World Heritage status for both their natural and cultural values. The natural landscape was acknowledged as a world heritage site in 1990 and its cultural values recognised in 1993. The area is the only site in New Zealand to be recognised for its cultural heritage, which acknowledges a people's cultural connection to the land.

Mt Pihanga is situated at the northern most point of Tongariro National Park. Rotopounamu is nestled into the flank of Mt Pihanga and is reached by a short 15 minute drive from Turangi. The high ecological values of Mt. Pihanga and Rotopounamu were identified by the Tongariro Natural History Society, now known as Project Tongariro (PT), and the Department of Conservation (DOC), as the site within the National Park best suited to restoration by a community based group. The Mt Pihanga/ Rotopounamu project was established in 2003 and is community focused with integrated pest management and advocacy at its core. Its overall vision is:

"Restoring the mauri of Mt Pihanga".

The project is managed according to the Rotopounamu Strategic Management Plan (2008), which aims to maintain, enhance (or re-establish) vulnerable habitats and ecosystems and their component fauna and flora; and to prevent the establishment of new threats. To achieve this, it lists nine biodiversity objectives. They are:

Objective 1:
To maintain sensitive canopy and sub canopy flora in a healthy condition and to protect vulnerable fauna by maintaining possum abundance at less than 5% RTCI[1] (or equivalent index) over the Mount Pihanga Restoration Area.

Objective 2:
To allow populations of small, vulnerable forest birds to increase in abundance by maintaining ship rat populations below a 5% footprint tracking index during the period October to February annually, over a minimum 530ha management area in the immediate vicinity of Rotopounamu.

Objective 3:
To allow kaka and kakariki populations to increase in abundance by maintaining stoat tracking indices at less than 5% footprint tracking index, over a minimum 530 ha management area in the immediate vicinity of Rotopounamu.

Objective 4:
To protect sensitive flora and fauna by maintaining possum populations at less than 2% RTCI, (or equivalent index), over a minimum 530 ha management area in the immediate vicinity of Rotopounamu.

[1] Residual Trap Catch Index – a standard possum control industry protocol for assessing relative possum density

Objective 5:
To enhance populations of threatened species within the Mount Pihanga – Rotopounamu Restoration Project Area by:

- Threatened species survey/inventory;
- Targeted species-specific management; and
- Re-introduction of suitable species as key pest control targets are achieved.

Objective 6:
To undertake site-led weed control of all exotic plant species found above the tree line on Mount Pihanga and on the margins of Rotopounamu.

Objective 7:
To sustain an increased level of pig control through targeted recreational hunting effort, and measure pig impacts on ground vegetation cover over the podocarp-hardwood strata of the Mount Pihanga Restoration Area.

Objective 8:
To maintain Rotopounamu free of introduced aquatic weeds and new aquatic pests, and investigate options and feasibility of smelt control for koaro enhancement.

Objective 9:
To minimise deer impacts over the Mount Pihanga Restoration Area so as to allow highly palatable flora to regenerate in the under-story.

The purpose of this document is to report progress made towards achieving these objectives and provide recommendations for ongoing management. This report focuses primarily on the key objectives currently being implemented, which are objectives 2, 4 and 6 but also provides an opportunity to review the remaining objectives and community relations initiatives / work implemented during the season.

3.0 Report against objectives

3.1. Objective 1:

To maintain sensitive canopy and sub canopy flora in a healthy condition and to protect vulnerable fauna by maintaining possum abundance at less than 5% RTCI[2] (or equivalent index) over the Mount Pihanga Restoration Area.

Mount Pihanga's flora, and some of its key fauna are highly sensitive to the browsing, competitive and predatory impacts of possums. For example, possums have already had significant impact through causing large-scale dieback of the Hall's totara belt which was once a dominant feature. Northern rata has also been virtually eliminated from the eastern slopes. Without control possums have the potential to cause further significant canopy losses to species such as kamahi and mahoe.

Mount Pihanga falls within the Animal Health Board's (AHB) Bovine Tuberculosis (Tb) Vector Area, Turangi Sector 5A. This area includes some 6,000 ha of bush habitat where possums, as a significant vector for the transmission of Tb to livestock, have been controlled under a national Tb Management Strategy using the aerial application of 1080 baits. The area has been subject to four aerial bait applications since the early 1990's based on a four to six year treatment cycle.

The next AHB aerial application treatment is due in winter 2013.

3.1.1. Management Recommendations - Objective 1

The Governance group should be talking to the AHB from early 2012/2013 season onwards about how the aerial 1080 operation will take place and how this is going to best work in with the Rotopounamu Restoration projects goals and objectives listed in the Rotopounamu Restoration Project Strategic Management Plan.

3.2. Objective 2:

To allow populations of small, vulnerable forest birds to increase in abundance by maintaining ship rat populations below a 5% footprint tracking index during the period October to February annually, over a minimum 530ha management area in the immediate vicinity of Rotopounamu.

Of all the threats to the suite of smaller forest birds present within the Mount Pihanga – Rotopounamu Restoration Project Area, ship rats have the greatest impact. Through both direct predation of nests (i.e., eggs, chicks and females) and competition for food (fruit, seeds and invertebrates), ship rats actively suppress and reduce the abundance of many of the smaller forest bird species (e.g., tomtit, fantail and robin). Control of ship rats has been clearly demonstrated to significantly increase general forest bird abundance at a wide range of forest restoration sites around New Zealand. Therefore to achieve

[2] Residual Trap Catch Index – a standard possum control industry protocol for assessing relative possum density

Objective 2, a bait station network has been implemented to control rats to below 5% footprint tracking index, and five minute bird counts are used to measure the outcome of the management.

3.2.1 Method

Bait stations

There are 1038 bait stations covering 530ha around Rotopounamu (see Appendix 3). These bait stations are filled with bait at the beginning of the breeding season for most birds (usually September), to help ensure successful recruitment of offspring into the population. The bait stations continue to be refilled with fresh bait until all bait is removed in March for the winter.

Diphacinone, 0.05g/kg, Pest Off cereal pellets was the rodent bait used this year due to the success obtained from this product in the 2010/11 season, with the footprint tracking index rate being the lowest recorded yet at the Rotopounamu Restoration project.

Bushworks contractors were employed to undertake the baiting operation this season. Ideally the first bait station fill would take place at the beginning of September but due to uncertainty of funding, the first fill was not completed until 10th October 2011. The second round of baiting started a fortnight later on the 25th October, followed by the third baiting round taking place a month later on the 23rd November. Based on last year's findings which showed the bait would last up to two months in the bait stations, the next bait replacement was left until 3rd January 2012 when a full replacement of bait took place. The bait was again left for a further two months before being removed in March 2012.

Spacing the bait station refills out to two monthly once rat numbers were low resulted in significant savings made this year in both contractor costs and cost of bait. Diphacinone, 0.05g/kg, Pest Off cereal pellets was pre-bagged into zip lock bags with 300g of bait per bag. The pre-packed bags were transported by DOC rangers to Rotopounamu and locked in large water tight boxes. Contractors then picked up the appropriate number of bags from the boxes to complete the number of bait station lines required. The contractors were asked to fill out a sheet to show how much bait had been taken at each station (i.e. 100%, 75%, 50%, 25%, 0%) and record bait stations that needed to be upgraded. On average, contractors completed 112 bait stations per day per person.

Bushworks contractors were unable to return to remove the bait at the end of the season so two local contractors, Asher Inc and Manawhenua Solutions, were employed on a trial basis.

Tracking tunnels

In the 2011/12 season the tracking tunnels were largely run by Project Tongariro volunteers. The tracking tunnel lines, which are numbered 1, 2, 3, 10, 11, 12, 13, are 500m long with 10 tracking tunnels on each line at 50m spacing's (Appendix 5). This system is principally used as an index of rat abundance in the 530ha of bush. Rat numbers were monitored using peanut butter and pre-inked cards placed in the tracking tunnel overnight and collected the following morning. Tracking tunnels were run October, December, January and March to monitor the success of the rodent baiting programme. Unfortunately due to weather and staff/volunteer availability at the time, the tracking tunnels were not run until 2 weeks after the baiting programme had started. This means that there was no

baseline tracking tunnel data prior to the operation taking place. The percentage of the rats and mice recorded on each line was used to give the abundance of rodents throughout the season. No tracking tunnels were run to monitor mustelids this season.

Bird counts

A bird count transect was established on every second bait station line, and each count station spaced 200 m apart (Appendix 7) with 129 stations in total. Three days of training was undertaken to ensure all observers were of equal skill in observing and identifying birds.

The counts were undertaken in early February, with five minutes spent at each station recording each different bird seen or heard. No bird was knowingly counted twice. In addition, the distance was estimated between the observer and any grey warbler, kereru and kakariki. Distances were estimated as 0-5m, 5-10m, 10-20m, 20-30m, 30-50m, 50-70m and 70-100m. This made distances easier to estimate and therefore reduce variability between observers. Distance sampling is used to estimate the density of birds around the observer, and is used as an additional monitoring method to bird counts.

With no control site in place, a comparison of birds call rates was made with Tongariro Forest to help determine whether trends seen at Rotopounamu were also being seen elsewhere. The full comparison can be seen at DOCDM – 948786.

3.2.2 Results / Discussion

Bait stations

In general, more bait was taken from the edges of the bait station network and from the southern side of the lake, which is only protected by a small number of bait stations (see Appendix 3). This is likely a reflection of the invasion pressure along the boundaries as rats first encounter the bait.

Pig interference became an issue this year with a large number of bait stations being tampered with. Bait stations were tipped up so the bait would fall out of the bait station then eaten and if the bait stations were secured top and bottom the pigs would pull the base plate out. The pigs seemed to move through the block over the period of just over a month before losing interest or moving outside the block.

Tracking tunnels

Rats were successfully maintained below 5% tracking rates for the duration of the season (Figure 1).

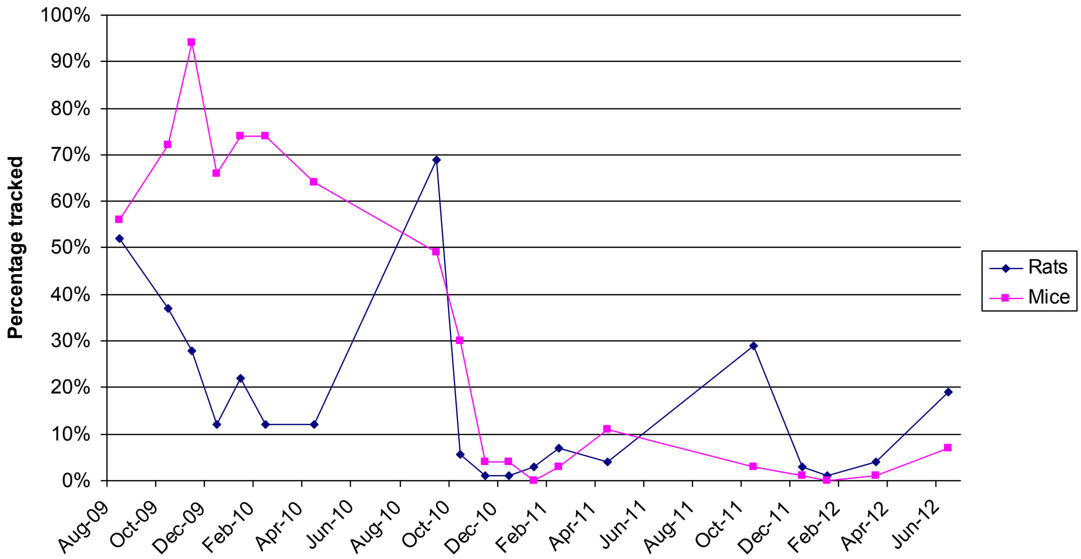

Figure 1. Rat and mice tracking tunnel footprint index from 2009 to2012 at Rotopounamu.

It is clear that when the Diphacinone, 0.05g/kg, Pest Off cereal pellets is used it is very effective at getting both rat and mice numbers down to low levels. The other notable factor is the short timeframe at which Diphacinone, 0.05g/kg, Pest Off cereal pellets reduced the rodent numbers down to below 5%. Once rodents were controlled to low levels they were maintained at fewer than 5% for the remainder of the bird breeding season. If baiting had started at the preferred time of the year (i.e. beginning of September) it is possible objective 2 (reducing rat tracking rates to below 5% from October to February) would have been achieved for the first time since the Rotopounamu project started.

Bird counts

This season has seen another good result for most passerine species with rifleman, silvereye and whitehead showing significant increases (Figure 2).

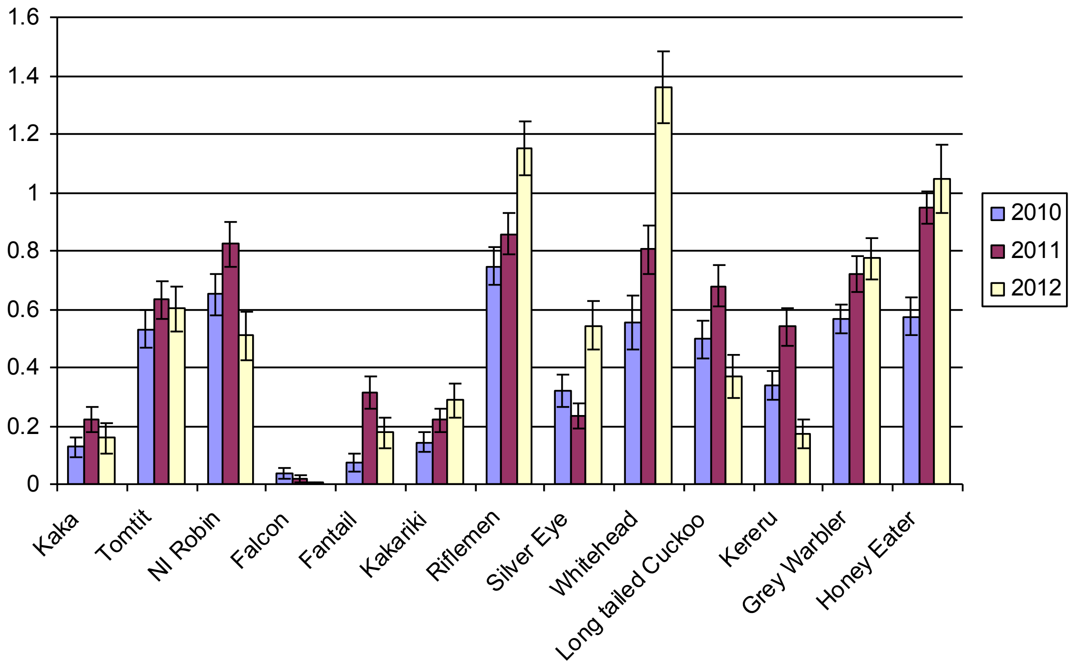

Figure 2: Average number of calls per 5 minute bird count for native bird species recorded at Rotopounamu.

This suggests that these birds have again had a successful breeding season. This is likely to be linked with the rats being controlled to below 5% tracking rate during the breeding season. This is especially encouraging for rifleman, who are classified as Declining (Miskelly *et al.* 2008). No significant change in call rates were observed for most species, such as kaka, tomtit, kakariki, grey warbler and honeyeaters (bellbird and tui).

Although robins decreased in call rates, this was thought be behavioural as robins were still frequently seen, but appeared to be quiet compared to previous years. Therefore robins would not have been detected as easily when carrying out 5 minute bird counts. Fantails declined significantly too, although this decline was mirrored in call rates in Tongariro Forest (DOCDM-948786). The species is known to fluctuate in numbers from year to year, and since the decline occurred at both sites, it might be part of their normal variation in numbers. Similarly long-tailed cuckoo call rates vary from year to year, therefore inter-season variation is considered normal.

The biggest decrease in bird numbers was seen by kereru. Kereru are mobile species that will fly large distances to where resources are available. It is likely the significant decrease in numbers is due to a lack of food available. This suggests that the poor weather season meant trees have not fruited as well as they have in previous years. A similar decline occurred in Tongariro Forest this season, probably for similar reasons. It is likely that kereru numbers will recover if the trees at Rotopounamu are more productive in the coming summer.

In all, most bird call rates remained relatively stable, or are thought to have declined due to behavioural or resource issues. It does suggest however, that this season was not as good a breeding season as the year before. Natural variation is to be expected, as bird numbers cannot continue to increase each year. However, the bait stations were not filled until October, which means that some first clutch broods may have been lost.

The successful reduction of rat numbers since then would have ensured that second clutches would have had a higher success rate. Next season's monitoring will determine whether starting in September will result in increases in bird call rates.

Due to staffing difficulties, only 248 counts were obtained. The smaller sample size compared to previous years reduces our ability to observe small changes in populations, but will allow for significant changes to be detected. To ensure that the distance sampling data can be used, at least 60 independent encounters of each bird species is required. Due to the smaller sample size, this was not achieved and distance sampling data could not be analysed this year.

3.2.3 Management Recommendations - Objective 2

- It is important to carry out tracking tunnels prior to the rodent control season commencing to get a clear picture of rodent population pre operation.

- Diphacinone, 0.05g/kg, Pest Off cereal pellets should be used next season as it is efficient and cheap. Bait should be delivered to bait stations (300g per bait station) by contractors starting in late August to the start of September 2012.

- It is important to ensure funding for the project is in place at the beginning of the season in order to achieve the conservation objectives identified in the Rotopounamu Restoration Strategic Management Plan.

- The first maintenance check should be completed after a fortnight, then out to monthly intervals. Once rodent levels are down to target levels (below 5% tracking tunnel indices) maintenance checks should be pushed out to two monthly intervals until the bait is removed in March. Bait stations should always have at least 150g of good condition bait available to rodents at any one time.

- Pre-bagging the bait into 300g zip lock bags makes delivering the bait easier for contractors and should continue in the 2012/13 season.

- When ordering poison warning signs, it needs to be specified that they will be outside and subjected to sunlight as the signs used this year deteriorated rapidly.

- If possible, enough bird counts should be undertaken to ensure at least 60 independent encounters with grey warbler, honeyeaters and kakariki to ensure that distance sampling can be used.

- Continue to compare data between Rotopounamu and Tongariro Forest as this helps to clarify trends seen each year.

3.3 Objective 3:

To allow kaka and kakariki populations to increase in abundance by maintaining stoat tracking indices at less than 5% footprint tracking index over a minimum 530 ha management area in the immediate vicinity of Rotopounamu

Stoats are significant forest predators in the Mount Pihanga–Rotopounamu Restoration Project Area. While their primary food source is rodents, they also incidentally prey on a wide range of native species including invertebrates, lizards, waterfowl and forest birds. As hole-nesting birds, kaka and kakariki are particularly sensitive to stoat predation as the majority of nests only have one entrance, which reduces the ability of the female to escape predation.

The best management approach to reducing stoat abundance to less than 5% footprint tracking index is sustained control using ongoing, intensive management through the placement of DOC 200 traps at a density of one trap set per two hectares within the rat control bait station grid.

Due to funding constraints this objective has not yet been fully implemented.

3.3.1 Management Recommendations - Objective 3

- A rodent control operation will not be necessary in the 2013/2014 season as the AHB will be carrying out an aerial 1080 operation winter 2013. This will create cost savings for the Rotopounamu project in this season. Project Tongariro should look to implement a stoat control infrastructure in the 2013/2014 season with savings made from the AHB aerial 1080 operation.

- Since the Rotopounamu Strategic Management Plan was written in 2009 there has been significant advances in knowledge around best practice for mustelid control. There has also been various new technologies developed like the Henry self setting trap. This year (2012/13) the governance group should review the prescription (7.3.3) of the Rotopounamu Strategic Management Plan and come up with a new prescription based around current best practice and new technologies.

3.4 Objective 4:

To protect sensitive flora and fauna by maintaining possum populations at less than 2% RTCI over a minimum 530ha management area in the immediate vicinity of Rotopounamu.

Ongoing management of possum density to very low levels within the Core Area provides an important opportunity to better manage more vulnerable plant species such as mistletoe and *Dactylanthus taylorii*, and negate the predatory impacts of possums on sensitive fauna such as kaka and kereru. Maintaining possum density at low levels in the Core Area also reduces potential possum interference on the rat control bait station network, which could severely reduce the effectiveness of this regime in protecting forest birds.

3.4.1 Method

Wax Tag

Ten wax tag lines were run this year at Rotopounamu. Each line is 200m long with wax tags placed every 10m nailed on to a tree 30cm off the ground. Four people laid the lines out, two people doing two lines and two people doing three lines. The same lines were used last season and the GPS locations are on record (see appendix 6). The tags were left out and collected after seven nights. The Bite Mark Index (BMI) was calculated by recording how many wax tags showed signs of possum bite marks.

3.4.2 Results/ Discussion

This year nine out of 200 wax tags had been chewed by a possum. This resulted in a BMI of 4.50 which is equivalent to a RTC of <1%. The majority of lines with possum bite marks were on lines that were close to the operational area boundary. Numbers were so low when the possum monitoring was carried out that a possum control operation did not take place this season in the 2011/12 season.

3.4.3 Management Recommendations - Objective 4

- With the AHB Aerial 1080 operation coming up winter 2013 no possum control should be carried out as unless the possum population has reached a level where they are likely too significantly interfere with the rodent bait stations. If possum numbers are less than 10% RTC then a control operation should not take place. If a possum control operation is required then Potassium Cyanide 475g/kg in Feratox encapsulated pellets (Feratox Strikers) should be placed at 25 meter intervals along the bait station lines prior to the rodent baiting programme starting. This job should be put out to contract as it is difficult to find enough approved handlers to carry out the work within DOC staff. Bushworks Contractors will be suitable to do this work. Feratox Strikers can be ordered from Connovations at approximately $1 each.

- If a possum control operation takes place then post monitoring should be run to assess the outcome of the possum operation against the target of objective 4, which is less than 2% RTCI

3.5 Objective 5:

To enhance populations of threatened species within the Mount Pihanga – Rotopounamu Restoration Project Area by:

- ***Threatened species survey/inventory;***
- ***Targeted species-specific management; and***
- ***Re-introduction of suitable species as key pest control targets are achieved.***

There are a number of threatened species present at Rotopounamu that have been identified as priority. Usually these are monitored on an annual basis, although specific work was not undertaken on the following species this year, predominantly due to resources and weather:

- Weta monitoring

- *Dactylanthus taylorii* monitoring
- White mistletoe monitoring
- Red and Yellow mistletoe survey
- Bat Survey

3.5.1 Management Recommendations - Objective 5

- *Dactylanthus taylorii*: The cages need to be changed to stainless steel cages as the current cages are made of galvanised steel which, over time, may leach into the soil and poison the plants. If stainless steel cages are available this should be carried out in the 2012/2013 season. There may be a possibility that DOC will have enough caging to carry out this work.

- White mistletoe: Project Tongariro Intern Chris Taipeti monitored white mistletoe at Rotopounamu in the 2010/2011 season for his placement report. The report is located in the DOC file system - Rotopounamu White Mistletoe survey 2010-2011 (DOCDM -758865). This is an excellent project for any keen Project Tongariro volunteers to get involved in. Advice on monitoring and locations of white mistletoe is available from DOC staff.

- Red and Yellow mistletoe: This is also an excellent project for any keen Project Tongariro volunteers to get involved in. Advice on monitoring and locations of red and yellow mistletoe is available from DOC staff.

- Bat Survey: Bats have been monitored in previous seasons and there is no further need for more information.

3.6. Objective 6:

To undertake site-led weed control of all exotic plant species found above the tree line on Mount Pihanga and on the margins of Rotopounamu.

There are three major weed species that is the focus of weed management as part of the Mount Pihanga–Rotopounamu Restoration Project. These include *Pinus contorta*, Ling heather (*Caluna vulgaris*) and grey willow (*Salix cinerea*). *Pinus contorta* and heather (*Caluna vulgaris*) threaten the alpine vegetation associations above bush line on Mount Pihanga, while heather and willow also establish around the shoreline of Rotopounamu. While a range of other weed threats exist, the intrinsic nature of mature podocarp-hardwood forest makes it reasonably resilient to most invasive weed species. The exceptions are bird-dispersed climbers such as Japanese honey-suckle (*Lonicera japonica*), Ivy (*Hedera helix*) and Old Man's Beard (*Clematis vitalba*) which are all present in adjoining areas. The threat posed by these species will be managed by surveillance activity with follow-up control as required.

The management strategy to best achieve this biodiversity objective is site-led management aimed at maintaining zero density at both the critical sites within the project area.

3.6.1 Method:

Site - led Weed Management

There are currently two weed species that are controlled in the Pihanga-Rotopounamu area. Ling Heather (*Calluna vulgaris*) is controlled on the summit

of Mt Pihanga and in two separate locations around the shores of Rotopounamu. Grey willow (*Salix cinerea*) is controlled on a tidal area at the first beach reached when walking the track in a clockwise direction (See map in Appendix 4).

Heather (*Calluna vulgaris*) control was undertaken on the summit of Pihanga on 28th February 2012. Heather was controlled using Metsulfuron at a rate of 5g/10 litres, but by lunch time the weather had deteriorated and the crew had to return early.

Due to DOC staff shortages the heather (*Caluna vulgaris*) and willow (*Salix cinerea*) sites were not controlled around Rotopounamu this season.

3.6.2 Management Recommendations - Objective 6

- Known heather (*Calluna vulgaris*) and grey willow (*Salix cinerea*) around the lake should be visited and controlled where necessary. Lake edges will be checked for any new incursions of pest plants, mapped and controlled where found.

- Project Tongariro members should notify DOC rangers of any weed sightings when they are carrying out trap checks. A practical weed identification and control/growsafe training could be organised for Project Tongariro volunteers and the interns to help with the surveillance and control of weed species on Pïhanga and around Rotopounamu. This should include knowledge of aquatic weed species to assist in the implementation of objective 8 (see below 3.7).

- Surveying and control of Heather (*Calluna vulgaris*) on the summit of Pihanga will be continued next season. Project Tongariro members will be offered the opportunity to join DOC staff on the annual weed control operation on the Pihanga summit.

3.7. Objectives 7, 8 and 9

These objectives are not addressed in this report as no actions were undertaken to achieve them. A general recommendation from this report (see section 6.1) is to review all the objectives in the Rotopounamu Strategic Management Plan including 7, 8 and 9, that when reviewing objective 8: To maintain Rotopounamu free of introduced aquatic weeds and new aquatic pests, and investigate options and feasibility of smelt control for koaro enhancement, it is important to note Rotopounamu is now the only aquatic weed free lake in the Taupo catchment.

4.0 Community Relations, Education and Awareness

4.1 Adopt a Hectare

In October 2011 Project Tongariro launched an initiative "Adopt a Hectare" which has received a positive response from the public. It takes roughly $100.00 per annum to maintain 1 hectare of native bush at Rotopounamu. The benefits that will come out of this initiative are:

- Generate income to fund the Rotopounamu Project
- Give supporters the opportunity to make a tangible donation so that they can directly see and feel the rewards of their contribution
- Raise the profile of the good work being done at Rotopounamu and Project Tongariro in general
- Extend the protection of this New Zealand jewel.

As at 30 June 2012, 60 hectares have been adopted by the community including individuals and local businesses that are committed to conservation in the local region.

4.1.2 Visitor Numbers

A track counter is present between the car park and the junction at the top of the hill, and records number of visitations each year (Figure 3 and 4). Totals shown in the graphs should be halved as the counter would count a person both entering the track and exiting the track.

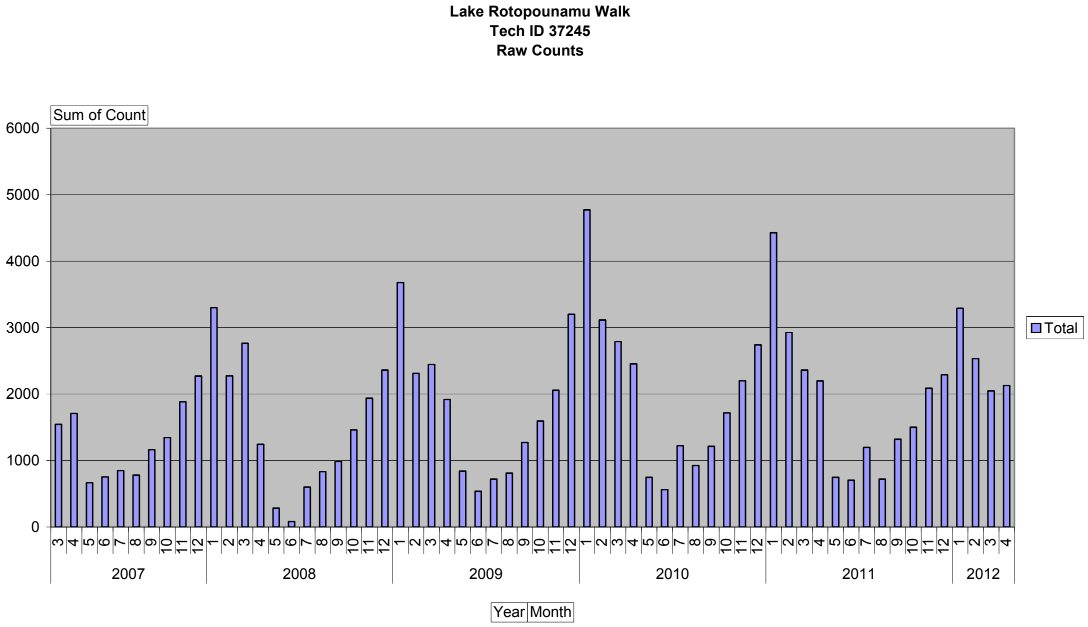

Figure 3: Number of visitors to Lake Rotopounamu broken down by month from 2007 to 2012.

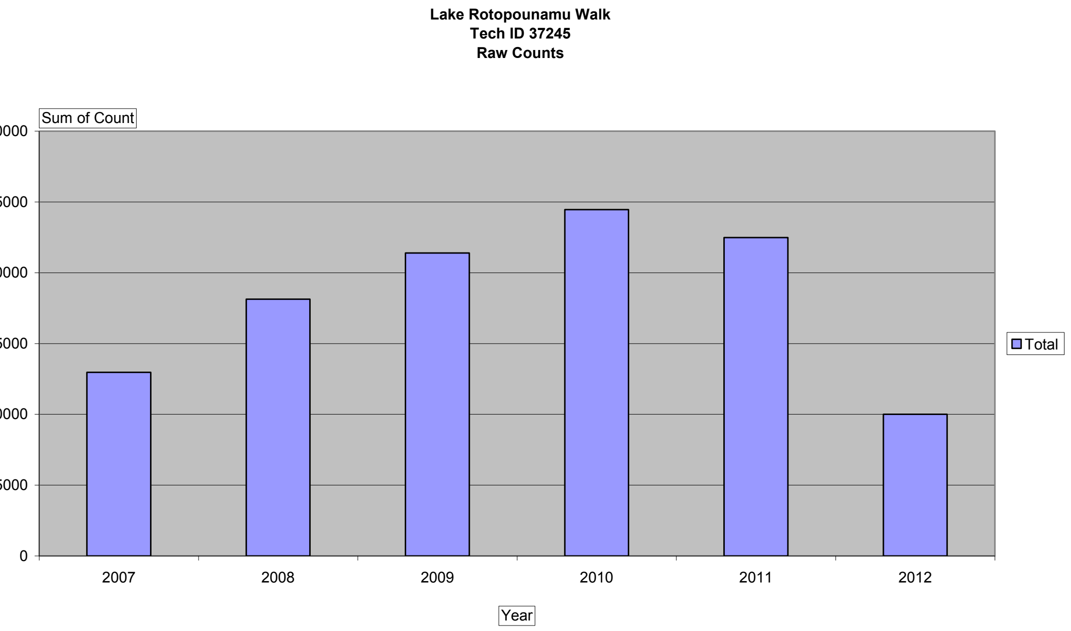

Figure 4: Number of visitors to Lake Rotopounamu annually from 2007 to 2012.

There were a total of 11,243 visitors for the calendar year 2011, down 988 visits in comparison to the 2010 year visitor figures. This is likely to be because of the poor weather conditions we have had over the last summer. So far for the 2012 season January – April, there have been 5,000 visitors to the site.

4.1.3 Predator traps

Rotopounamu has a network of 50 DOC 200 traps located around the track placed at 100 metre spacing. These traps are designed to target rats and mustelids and act as an advocacy tool for demonstrating pest control methods. Project Tongariro members run this programme and check the traps fortnightly.

Fresh eggs are used in the traps all year round and occasionally volunteers add salted rabbit meat for variety. In the 2011/12 season the Project Tongariro volunteers have trialled using golf balls instead of eggs and have had very good success. They have also fitted all of the traps with a flag system showing when a trap has been set off. A lot of the traps can now be checked without having to leave the track.

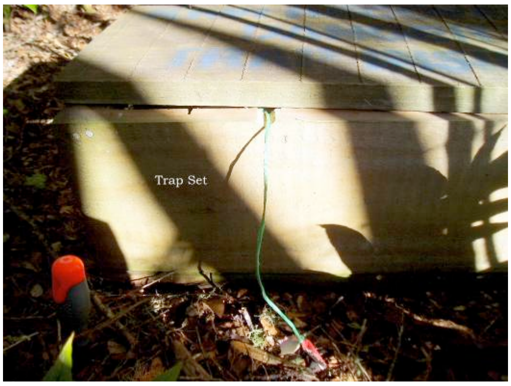

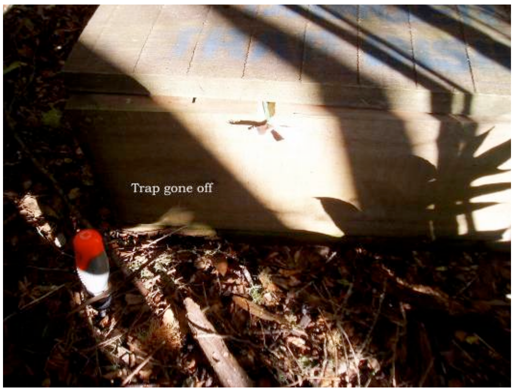

New flag system on the DOC 200 traps. Photo: K. Griffith.

Up until 2010 the traps had been shut down over the winter months. Last winter the Project Tongariro volunteers took the initiative to run the traps throughout the year including the winter months. This explains the higher numbers of rats caught in the last two seasons even though there has been a greater success with the baiting programme. Rats are more likely to be caught over the winter months as there is less food source and they are actively searching for alternative food sources. Trapping results have been collected since 2003. Each of the seasons shown in Figure 5 show results for the months October – September. The data shown for 2011/12 shows results from October 2011 – 2nd July 2012 so has three months left in the DOC 200 trapping season.

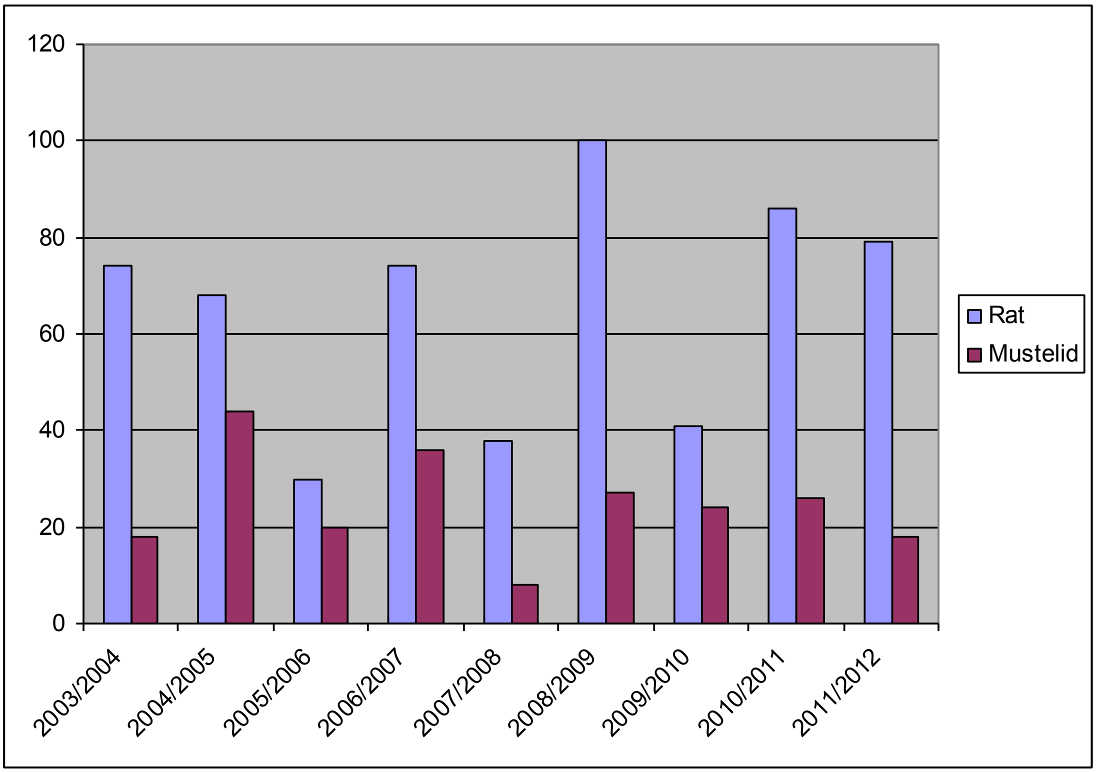

Figure 5: Number of rats and mustelids caught over the past 8 seasons in the DOC 200 traps.

Note that the 2007/08 season had low numbers due to the 1080 drop that year and the 2005/06 season had a small data set, only showing catch rates over 3 months. Figure 6 shows the comparison of catch rate between months this season 2011/12.

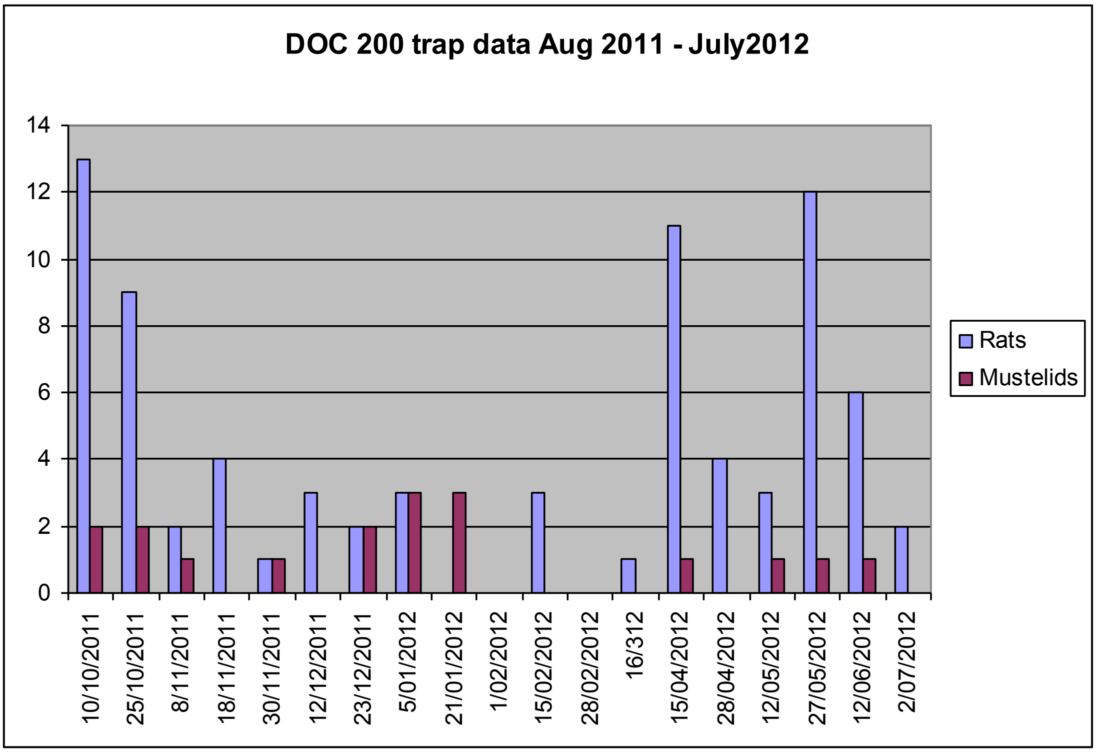

Figure 6: Number of rats and mustelids caught over the period August 2011-July 2012

4.1.4 Management Recommendations - Community Relations, Education and Awareness:

All tree plaques need to be concreted into place at Rotopounamu. This would be a good winter/spring job for a DOC ranger and PT volunteers before the busy summer season starts.

Trapping should continue around the lake throughout the winter and on into next season. Other Project Tongariro members should be encouraged to take part in checking the traps. Experienced volunteers can teach new volunteers. Designated volunteer to continue to collate data for this programme as Lyn Thomas has done over the 2011/12 season.

It is the 10th anniversary of the Rotopounamu project and therefore an opportunity to celebrate the work that has been achieved. Over the years Rotopounamu has given many volunteers and interns the opportunity to learn new skills and get involved in conservation. Focusing on the volunteers/interns over the years and where they are now could be a good angle for the PT website / newsletter etc.

5.0 Staff and Financial Contribution

In the 2011/12 season volunteers contributed a huge amount of time to the Rotopounamu project carrying out a number of tasks such as preparing bait, running tracking tunnels, clearing and checking the DOC 200 traps and carrying out five minute bird counts. These volunteers not only provide contributions directly but are also great advocates for the Rotopounamu project and conservation in general.

In addition to this, a total of 921hrs (115 person days) were put into the project from Department of Conservation staff (July to June).

In the 2011/12 season the Mt Pihanga-Rotopounamu restoration project received funding from:

- Huckleberry's Sports and Charitable Society Inc
- Sir John Logan Campbell Residuary Estate
- W N Pharazyn Trust
- Craters of the Moon Charitable Trust

6.0 Summary of Recommendations

It is important to note these recommendations are based on the same amount of funding being available as previously experienced in the 2011/12 year.

6.1 General

- The Rotopounamu Strategic Management Plan is a 5 year plan and was first written in 2008. It was then reviewed and signed off by DOC and PT in July 2009. However the sections in the plan which outline tasks do not identify work beyond 2012. Review all the objectives of the Rotopounamu Strategic Management Plan during the 2012/13 business year.

- The DOC internal computer DOCDM filing system has a specific file path set up for Rotopounamu. (DOCDM/Natural Heritage/Ecosystems/Mainland Islands/ Rotopounamu Restoration) which is not currently being used. It is important that all DOC reports/data that are saved to DOCDM are saved here to enable DOC staff, and therefore other community groups, to access this information.

- Set up a single excel Rotopounamu metadata spreadsheet so that everyone knows where all the information/data/reports/plans and GIS files are kept. O Tu Wharekai (national wetland restoration project) has a good example of this type of spreadsheet.

6.2 Objective 1

- The Governance group should be talking to the AHB from early 2012/2013 season onwards about how the aerial 1080 operation will take place and how this is going to best work in with the Rotopounamu Restoration projects goals and objectives listed in the Rotopounamu Restoration Project Strategic Management Plan.

6.3 Objective 2

- Diphacinone, 0.05g/kg, Pest Off cereal pellets should be used next season as it is efficient and cheap. Bait should be delivered to bait stations (300g per bait station) by contractors starting in late August to the start of September 2012.

- It is important to ensure funding for the project is in place at the beginning of the season in order to achieve the conservation objectives identified in the Rotopounamu Restoration Strategic Management Plan.

- The first maintenance check should be completed after a fortnight then out to monthly intervals. Once rodent levels are down to target levels (below 5% tracking tunnel indices) maintenance checks should be pushed out to two monthly intervals until the bait is removed in March. Bait stations should always have at least 150g of good condition bait available to rodents at any one time.

- Pre-bagging the bait into 300g zip lock bags makes delivering the bait easier for contractors and should continue in the 2012/13 season.

- When ordering poison warning signs, it needs to be specified that they will be outside and subjected to sunlight as the signs used this year deteriorated rapidly.

- If possible, enough bird counts should be undertaken to ensure at least 60 independent encounters with grey warbler, honeyeaters and kakariki to ensure that distance sampling can be used.

- Continue to compare data between Rotopounamu and Tongariro Forest as this helps to clarify trends seen each year.

6.4. Objective 3

- A rodent control operation will not be necessary in the 2013/2014 season as the AHB will be carrying out an aerial 1080 operation winter 2013. This will create cost savings for the Rotopounamu project in this season. Project Tongariro should look to implement a stoat control infrastructure in the 2013/2014 season with savings made from the AHB aerial 1080 operation.

- Since the Rotopounamu Strategic Management Plan was written in 2009 there has been significant advances in knowledge around best practice for mustelid control. There has also been various new technologies developed like the Henry self setting trap. This year (2012/13) the governance group should review the prescription (7.3.3) of the Rotopounamu Strategic Management Plan and come up with a new prescription based around current best practice and new technologies.

6.5. Objective 4

- With the AHB Aerial 1080 operation coming up winter 2013 no possum control should be carried out as unless possums are at a level where they are likely too significantly interfere with the rodent bait stations. If possum numbers are less than 10% RTC then a control operation should not take place. If a possum control operation is required then Potassium Cyanide

475g/kg in Feratox encapsulated pellets (Feratox Strikers) should be placed at 25 meter intervals along the bait station lines prior to the rodent baiting programme starting. This job should be put out to contract as it is difficult to find enough approved handlers to carry out the work within DOC staff. Bushworks Contractors will be suitable to do this work. Feratox Strikers can be ordered from Connovations at approximately $1 each.

- If a possum control operation takes place then post monitoring should be run to assess the outcome of the possum operation against the target of objective 4, which is less than 2% RTCI

6.6. Objective 5

- Wood Rose (*Dactylanthus taylorii*). The cages need to be changed to stainless steel cages as the current cages are made of galvanised steel which, over time, may leech into the soil and poison the plants. If stainless steel cages are available this should be carried out in the 2012/2013 season. There may be a possibility that DOC will have enough caging to carry out this work.

- White Mistletoe (*Tupeia antarctica*): Project Tongariro Intern Chris Taipeti monitored white mistletoe at Rotopounamu in the 2010/2011 season for his placement report. The report is located in the DOC file system - Rotopounamu White Mistletoe (*Tupeia antarctica*) survey 2010-2011 (DOCDM -758865). This is an excellent project for any keen Project Tongariro volunteers to get involved in. Advice on monitoring and locations of white mistletoe is available from DOC staff.

- Red Mistletoe (*Peraxilla tetrapetala*) and Yellow Mistletoe (*Alepis flavida*): This is also an excellent project for any keen Project Tongariro volunteers to get involved in. Advice on monitoring and locations of red and yellow mistletoe is available from DOC staff.

- Bat Survey*:* Bats have been monitored in previous seasons and there is no further need for more information.

6.7. Objective 6

- Known heather (*Calluna vulgaris*) and grey willow (*Salix cinerea*) infestations around the lake should be visited and controlled where necessary. Lake edges will be checked for any new incursions of pest plants, mapped and controlled where found.

- Project Tongariro members should notify DOC rangers of any weed sightings when they are carrying out trap checks. A practical weed identification and control/Growsafe training could be organised for Project Tongariro volunteers and the interns to help with the surveillance and control of weed species on Pïhanga and around Rotopounamu. This should include knowledge of aquatic weed species to assist in the implementation of objective 8 (see below 3.7).

- Surveying and control of Heather (*Calluna vulgaris*) on the summit of Pihanga will be continued next season. Project Tongariro members will be offered the opportunity to join DOC staff on the annual weed control operation on the Pihanga summit.

6.8. Community Awareness and Education:

- All tree plaques need to be concreted into place at Rotopounamu. This would be a good winter/spring job for a DOC ranger and PT volunteers before the busy summer season starts.

  Trapping should continue around the lake throughout the winter and on into next season. Other Project Tongariro members should be encouraged to take part in checking the traps. Experienced volunteers can teach new volunteers. Designated volunteer to continue to collate data for this programme as Lyn Thomas has done over the 2011/12 season.

- It is the 10th anniversary of the Rotopounamu project and therefore an opportunity to celebrate the work that has been achieved. Over the years Rotopounamu has given many volunteers and interns the opportunity to learn new skills and get involved in conservation. Focusing on the volunteers / interns over the years and where they are now could be a good angle for the PT website / newsletter etc.

7.0 Acknowledgements

I would like to thank everyone who contributed to the success of the Rotopounamu project this year. Kiri Te-Wano has done a great job in the first season in her new role, along with Nina Manning. Kevin and Beth Griffiths, Shirley Potter, Karen Arden and Noel and Lyn Thomas the team of core Project Tongariro volunteers running the DOC 200 trap network, running tracking tunnels and providing great feedback to DOC about current track conditions, windfalls etc. Project Tongariro Interns, Kelly and Jenny who helped with a variety of PT projects and a number of DOC projects. We would also like to thank and acknowledge the help received from DOC staff Jessica Scrimgeour, Leith Rhynd, Nathaniel Mellon and Theo Wylie.

8.0 References

Department of Conservation (2009). *Rotopounamu strategic management plan.* Department of Conservation, Turangi.

Lloyd, B. (1991). *Conservation of kaka in New Zealand.* Science and Research Internal Report No.101. Department of Conservation, Wellington.

Mander, C; Hay, R; Powlesland, R. (1998). *Monitoring and management of Kereru (Hemiphaga novaeseelandiae).* Department of Conservation Technical Series No15. Department of Conservation, Wellington.

O'Donnell, C.F.J; Dilks, P, J. (1986). *Forest birds in South Westland – status, distribution and habitat use*. New Zealand Wildlife Service Occasional Publication No10. Department of Internal Affairs, Wellington.

O'Donnell, C.F.J; Rasch, G. (1991). *Conservation of kaka in New Zealand.* Science and Research Internal Report No.101. Department of Conservation, Wellington.

Location of Rotopounamu Reports:

Pre 2009 reports – Tongariro Natural History Society	
2009-2010 Report – DOC, Turangi	DOCDM -590663
2010-2011 Report – DOC, Turangi	DOCDM-750543
Rotopounamu Strategic Management Plan – DOC, Turangi	DOCDM- 463822

9.0 Appendices

Appendix 1: Contractors Details and Costs

Bushwork Contracting – Chris Brausch
139 Works Rd
RD2
Katikati
PH: 021 039 3540
Email: chris.brausch

Asher Inc
110 Taupo View Road
Taupo
New Zealand
EMAIL: jude.asher@xtra.co.nz

Manawhenua Solutions
6 Parerohi Grove
Turangi
3381
PH: 022 098 0493

Summary of Contractors Costs*:

Baiting Round	Date	Cost excluding gst	Contractor
Round 1 – Initial fill	10/10/2011	$5,000.00	Chris Brausch
Round 2 - Maintenance	25/10/2011	$4,393.00	Chris Brausch
Round 3 – Maintenance	23/11/2011	$4,393.00	Chris Brausch
Round 4 – Complete replacement	03/01/2012	$5,400.00	Chris Brausch
Bait removal	26/03/2012	$4,400.00	Split between Manuwhenua solutions and Asher Inc
**Total cost of baiting**		**$23,586.00**	

*Accommodation costs are on top of these prices.

Appendix 2: Details of Pest Bait

PestOff50D
Animal Control Products Ltd
Whanganui
Ph 06 344 5302

Cost per 10kg bag Diphacinone, 0.05g/kg, Pest Off cereal pellets: $41.00 + Freight (excluding gst)

Bait costs incurred for the 2011/12season:
100 x 10kg bags of Diphacinone, 0.05g/kg, Pest Off cereal pellets = $4,100.00 (excluding gst)

Chemstock NZ Ltd – Waste Bait Disposal
Ed Hills
PO Box 80
Fielding
Ph 0800 243 678
Chemstock will deliver bait new bait from Whanganui and return empty pales (pales used to dispose bait).

Cost of bait delivery and disposal of old bait at the end of the season
$1,100.00

Connovation Limited
PO Box 58613
Manukau
Ph 021 273 4880
www.connovation.co.nz

Approximately $1/ striker +GST
2000 strikers $2000
(Feratox strikers for possum control operation if required)

Appendix 3: Rotopounamu Bait Station Layout

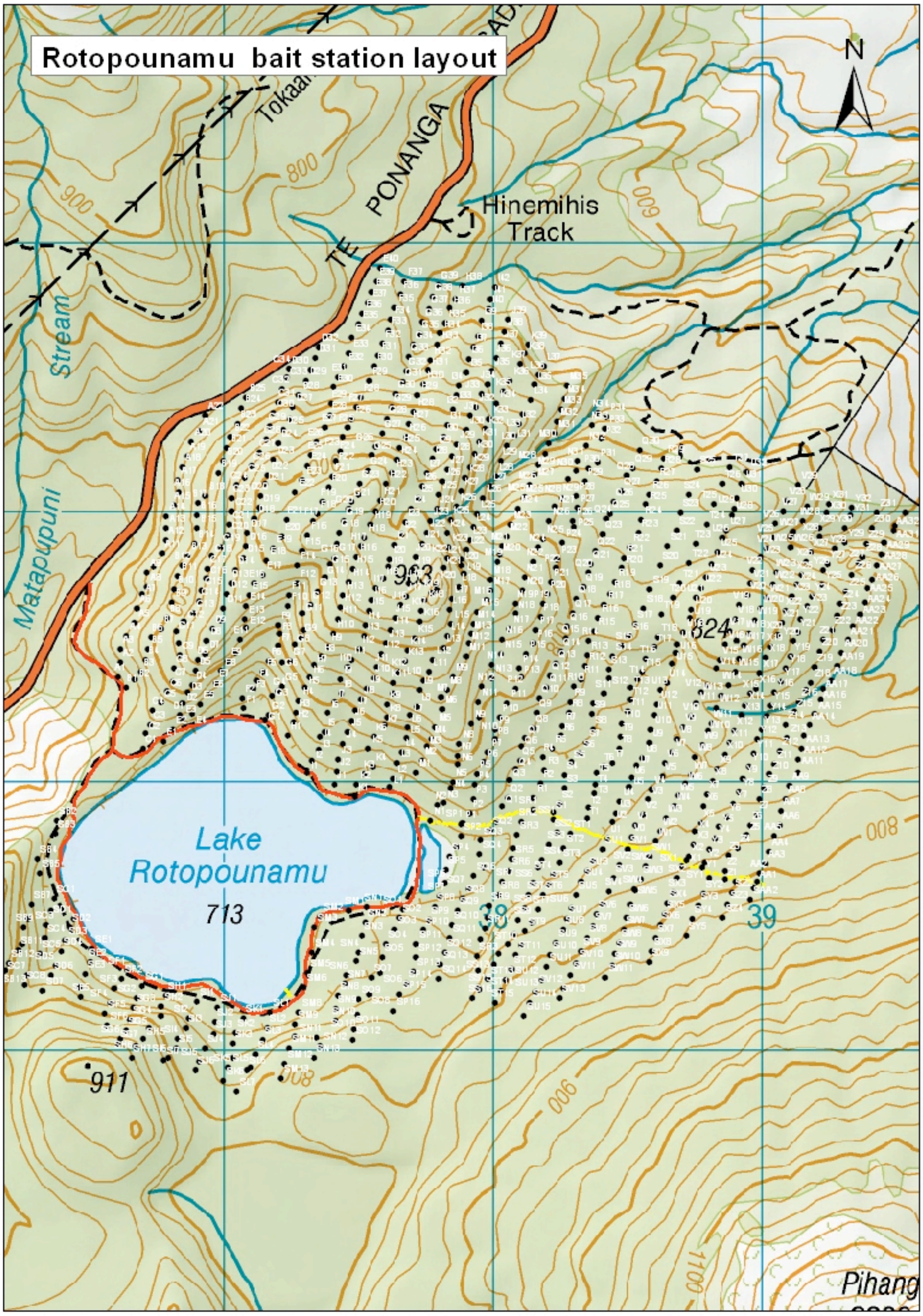

Appendix 4: Pest Plant Locations at Rotopounamu

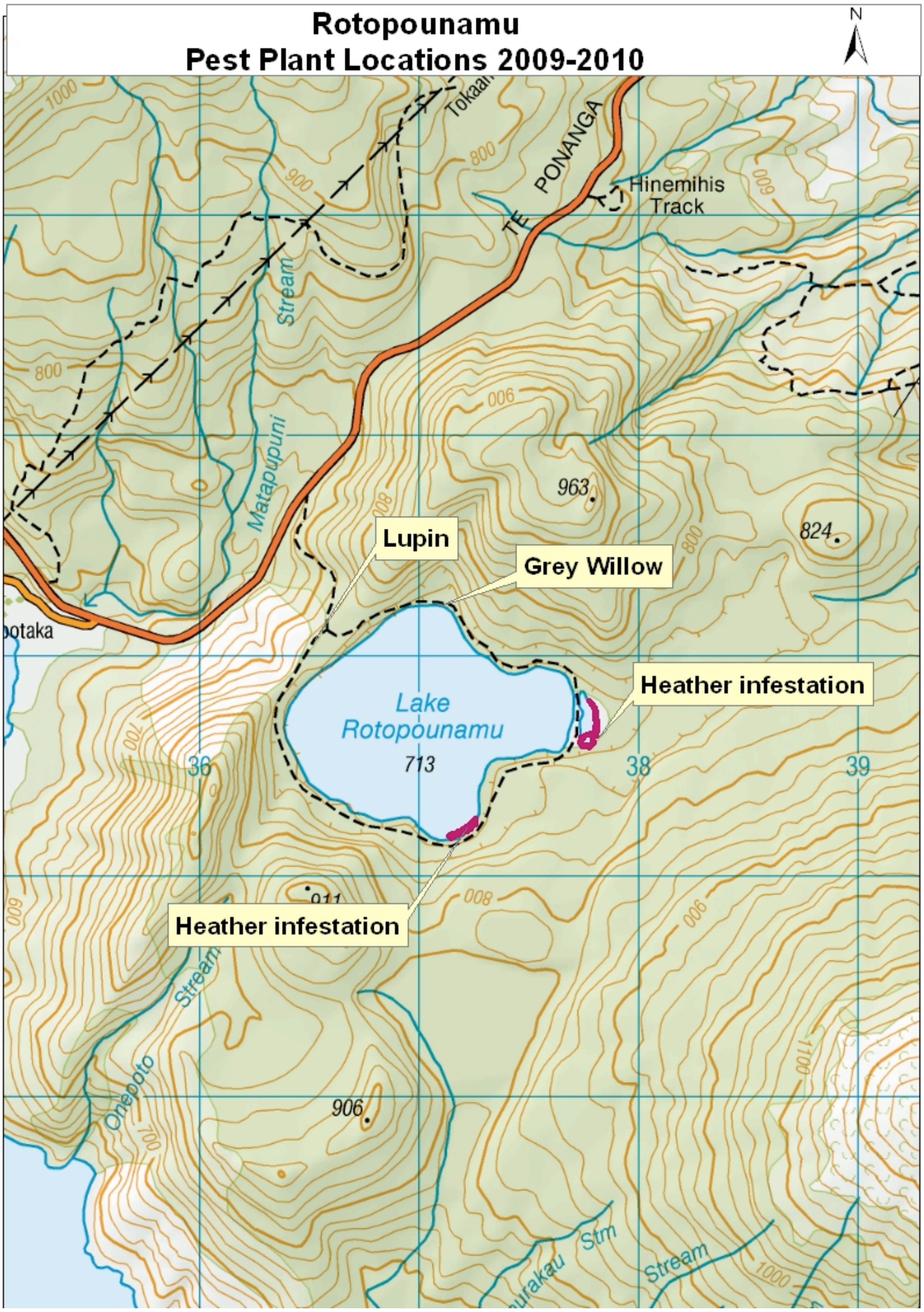

Appendix 5: Tracking Tunnel Line Locations at Rotopounamu

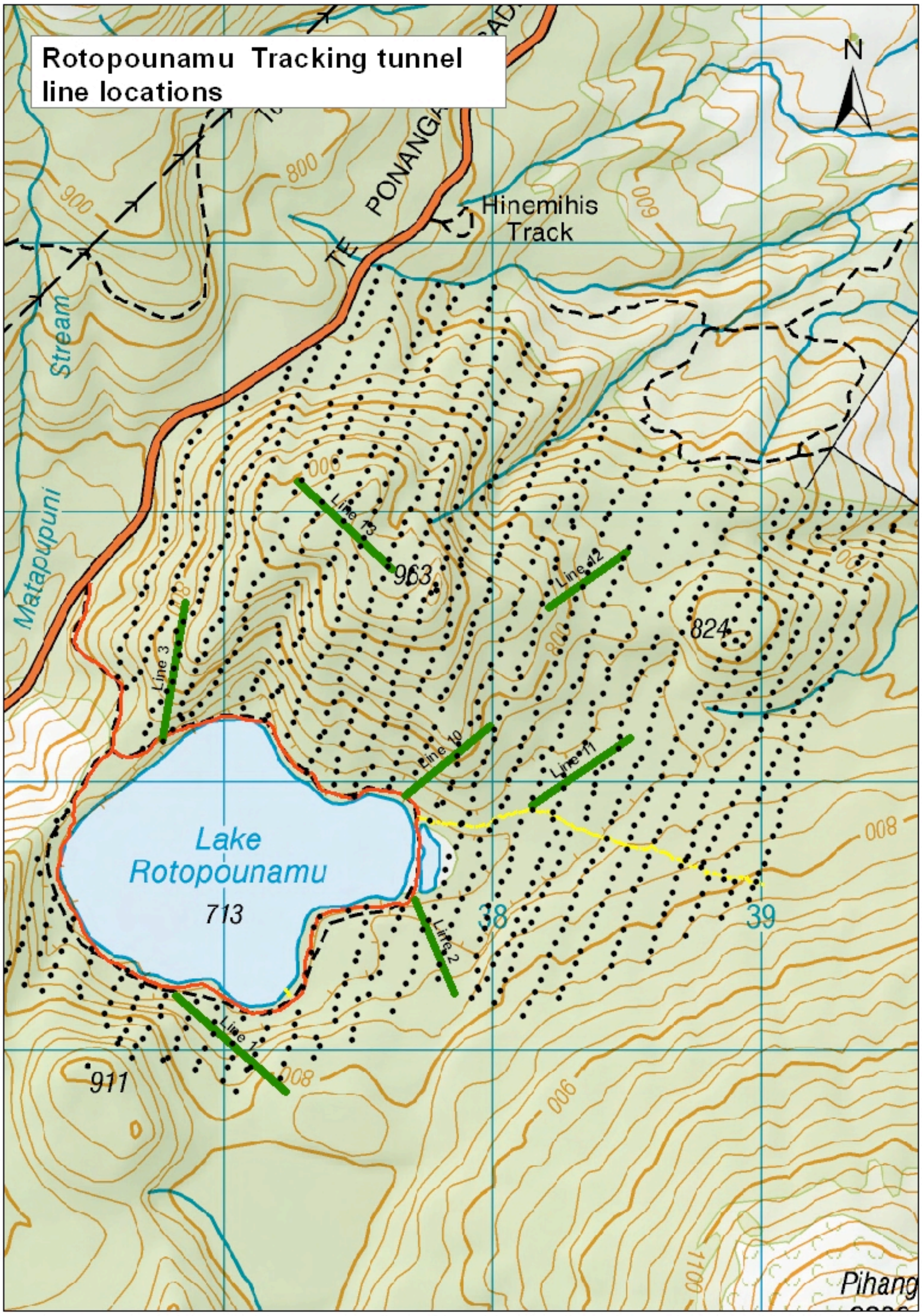

Appendix 6: Wax Tag Line Locations at Rotopounamu

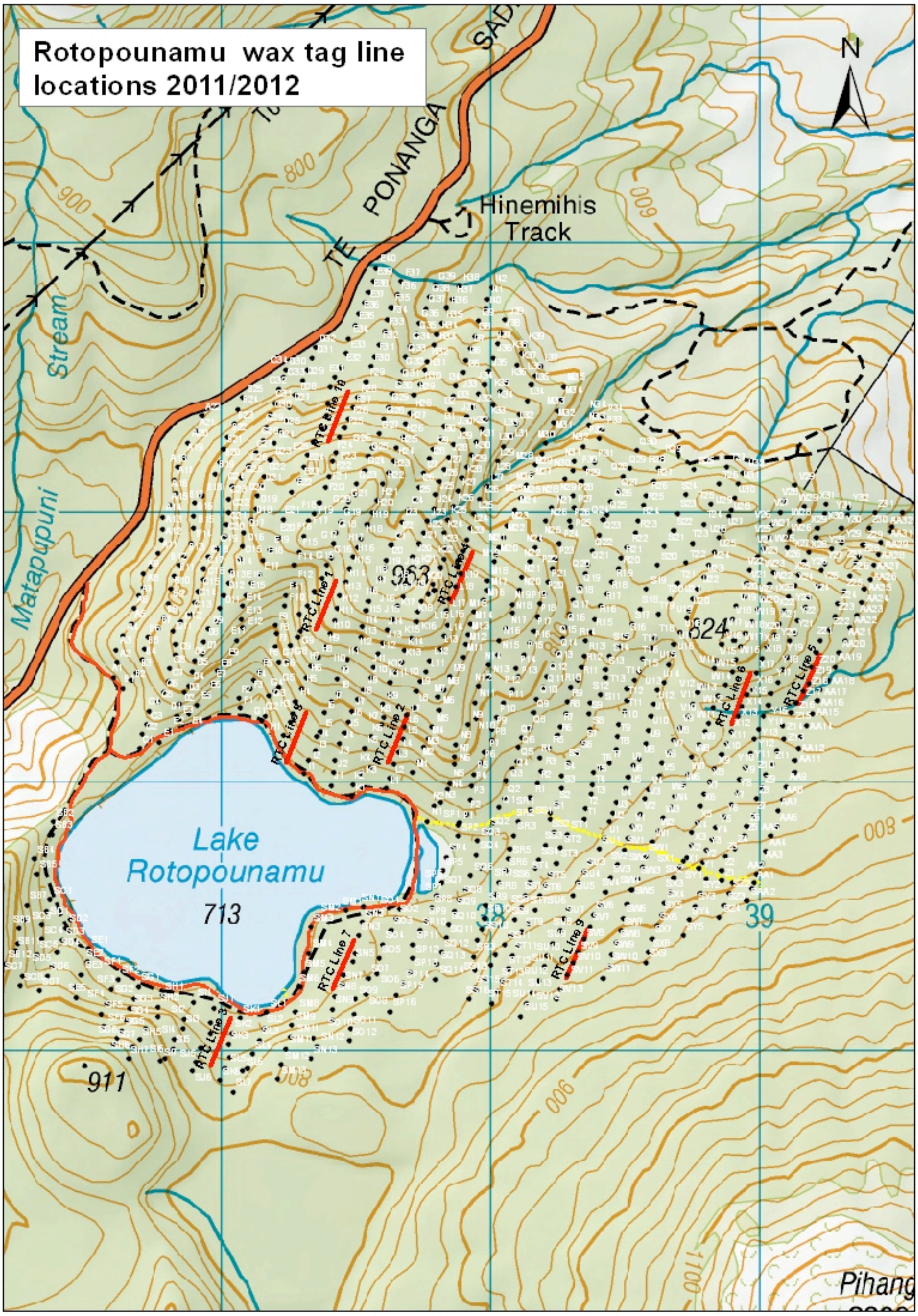

Appendix 7: Five Minute Bird Count Locations.

Line	Bait station number	# of counts per line	
Line C	1 5 9 13 17 21 25 29 33	(9)	
Line E	1 5 9 13 17 21 25 29 33 37	(10)	
Line G	1 5 9 13 17 21 25 29 33 37	(10)	
Line I	1 5 9 13 17 21 25 29 33 37	(10)	
Line K	1 5 9 13 17 21 25 29 33 37	(10)	
Line M	1 5 9 13 17 21 25 29 33 37	(10)	
Line P	1 5 9 13 17 21 25 29 33	(9)	
Line R	1 5 9 13 17 21 25 29	(8)	
Line T	1 5 9 13 17 21 25	(7)	
Line V	1 5 9 13 17 21 25	(7)	
Line X	1 5 9 13 17 21 25	(7)	= 97 counts
Line SB	1 5 9 13	(4)	
Line SD	1 5	(2)	
Line SF	1 5	(2)	
Line SH	1 5	(2)	
Line SJ	1 5	(2)	
Line SL	1 5	(2)	
Line SN	1 5 9 13	(2)	= 18 counts
Line SP	4 8 12	(3)	
Line SR	4 8 12	(3)	
Line ST	4 8 12	(3)	
Line SV	4 8 12	(3)	
Line SX	4 8	(2)	= 14 counts

Total counts per round =129 counts

4 complete rounds = 516 counts
