# Supplementary material for: Responses of New Zealand forest birds to management of introduced mammals
Source: Conserv Biol. 2020 Mar 23;35(1):35–49. doi: 10.1111/cobi.13456 (PMC7984369; doi:10.1111/cobi.13456)
Supplement: Supplementary file 8 — Supporting Material [file COBI-35-35-s007.docx]

**Appendix S13.** From personal communication with Greg Moorcroft (08-06-2017). Kererū is the Māori name for the New Zealand Pigeon.

| Table 5.10.1. Total density (individuals/count), richness (s=number of species/count), diversity (*H’*=Shannon-Wiener Index) and equitability (H/In*s*) of bird communities from data recorded during five-minute bird counts in Otamatuna Core Area, Te Urewera Mainland Island, 1996/97 - 2010/11 (In DM 157165). | | | | | | | | | | | | | | |
| --- | --- | --- | --- | --- | --- | --- | --- | --- | --- | --- | --- | --- | --- | --- |
|  | 1997 Winter (*n*=89) | | 1998 Winter (*n*=206) | | 1999 Winter  (*n*=210) | | 2000 Winter  (*n*=155) | | 2001 Winter  (*n*=131) | | 2007 Winter  (*n*=200) | | **2011 Winter**  **(*n*=200)** | |
| Species | Mean | SE | Mean | SE | Mean | SE | Mean | SE | Mean | SE | Mean | SE | **Mean** | **SE** |
| Rearea, bellbird | 3.079 | 0.019 | 3.028 | 0.008 | 5.043 | 0.148 | 5.517 | 0.456 | 4.107 | 0.016 | 2.125 | 0.888 | **4.055** | **0.154** |
| Blackbird | 0.112 | 0.004 | 0.393 | 0.003 | 0.171 | 0.028 | 0.117 | 0.024 | 0.298 | 0.005 | 0.17 | 0.030 | **0.06** | **0.0168** |
| Chaffinch | 0.022 | 0.002 | 0.000 | - | 0.010 | 0.007 | 0.000 | - | 0.023 | 0.001 | 0.000 | - | **0.045** | **0.0163** |
| Dunnock | 0.011 | 0.001 | 0.015 | 0.001 | 0.019 | 0.009 | 0.006 | 0.006 | 0.031 | 0.001 | 0.000 | - | **-** | **-** |
| Kaeaea falcon | 0.000 | - | 0.000 | - | 0.014 | 0.008 | 0.000 | - | 0.000 | - | 0.005 | 0.005 | **-** | **-** |
| Piwakawaka, fantail | 0.607 | 0.009 | 0.490 | 0.004 | 0.710 | 0.051 | 0.428 | 0.062 | 0.427 | 0.005 | 0.36 | 0.043 | **0.395** | **0.045** |
| Greenfinch | 0.000 | - | 0.005 | 0.000 | 0.000 | - | 0.000 | - | 0.000 | - | 0.000 | - | **-** | **-** |
| Kahu, harrier hawk | 0.000 | - | 0.000 | - | 0.005 | 0.005 | 0.000 | - | 0.000 | - | 0.01 | 0.007 | **0.005** | **0.005** |
| Kaka | 0.000 | - | 0.005 | 0.000 | 0.000 | - | 0.012 | 0.007 | 0.214 | 0.004 | 0.035 | 0.013 | **0.1** | **0.027** |
| Kakariki | 0.090 | 0.004 | 0.155 | 0.002 | 0.119 | 0.028 | 0.094 | 0.028 | 0.168 | 0.004 | 0.095 | 0.025 | **0.13** | **0.033** |
| Kereru | 0.360 | 0.009 | 0.641 | 0.005 | 0.833 | 0.066 | 0.634 | 0.077 | 0.954 | 0.007 | 0.505 | 0.052 | **0.715** | **0.066** |
| Kotare, kingfisher | 0.000 | - | 0.005 | 0.000 | 0.000 | - | 0.000 | - | 0.000 | - | 0.000 | - | **-** | **-** |
| Kokako | 0.045 | 0.003 | 0.112 | 0.002 | 0.138 | 0.037 | 0.252 | 0.049 | 0.328 | 0.006 | 0.495 | 0.069 | **0.46** | **0.059** |
| Magpie | 0.022 | 0.002 | 0.078 | 0.002 | 0.010 | 0.010 | 0.013 | 0.009 | 0.000 | - | 0.000 | - | **-** | **-** |
| Pheasant | 0.000 | - | 0.005 | 0.000 | 0.000 | - | 0.000 | - | 0.000 | - | 0.000 | - | **-** | **-** |
| Rifleman | 0.742 | 0.012 | 0.772 | 0.004 | 0.757 | 0.049 | 0.958 | 0.128 | 0.817 | 0.007 | 0.675 | 0.057 | **0.32** | **0.0495** |
| North Island Robin | 0.517 | 0.009 | 1.141 | 0.005 | 1.295 | 0.067 | 2.294 | 0.232 | 1.695 | 0.008 | 1.26 | 0.066 | **1.4** | **0.069** |
| Pihipihi, silvereye | 1.404 | 0.024 | 5.461 | 0.032 | 6.795 | 0.640 | 10.331 | 1.030 | 7.099 | 0.061 | 7.845 | 0.612 | **2.26** | **0.155** |
| Spur-winged Plover | 0.011 | 0.001 | 0.000 | - | 0.000 | - | 0.000 | - | 0.000 | - | 0.000 | - | **-** | **-** |
| Mimiro, tomtit | 0.483 | 0.008 | 0.553 | 0.003 | 0.567 | 0.048 | 1.122 | 0.111 | 0.550 | 0.005 | 0.47 | .0447 | **0.34** | **0.038** |
| Koko, tui | 1.045 | 0.015 | 0.505 | 0.003 | 1.062 | 0.080 | 1.471 | 0.134 | 2.206 | 0.012 | 0.9 | 0.0653 | **0.815** | **0.0705** |
| Grey Warbler | 0.966 | 0.011 | 0.859 | 0.005 | 0.710 | 0.053 | 1.677 | 0.148 | 0.863 | 0.006 | 0.87 | 0.055 | **1.05** | **0.0725** |
| Whitehead | 0.382 | 0.010 | 1.039 | 0.010 | 1.381 | 0.207 | 3.031 | 0.391 | 2.115 | 0.036 | 1.685 | 0.240 | **0.87** | **0.115** |
| Richness | 4.640 | 0.017 | 5.898 | 0.009 | 6.400 | 0.129 | 7.445 | 0.156 | 7.015 | 0.012 | 5.95 | 0.128 | **5.85** | **0.118** |
| Diversity | 0.392 | 0.002 | 0.417 | 0.001 | 0.425 | 0.018 | 0.360 | 0.016 | 0.325 | 0.001 | 1.406 | 0.025 | **1.56** | **0.021** |
| Equitability | 0.322 | 0.003 | 0.282 | 0.001 | 0.266 | 0.018 | 0.195 | 0.012 | 0.178 | 0.001 | 0.815 | 0.010 | **0.904** | **0.037** |
| Total Density | 9.899 | 0.051 | 15.003 | 0.036 | 19.638 | 0.774 | 27.958 | 2.251 | 21.893 | 0.080 | 17.50 | 1.477 | **13.025** | **0.99** |

**Appendix S14.**

| Mean call rates (and standard errors) for eleven bird species in the Aorangi Forest Park (Wairarapa, New Zealand) by Nyree Fea collected for her doctoral thesis (acquired June 2017). Call rates for the treatment site (Aorangi Forest Park) and non-treatment site (Remutaka Range)) are annual summaries calculated from data collected in Spring and the following Summer. Mammal control, through aerial application of 1080, occurred in August 2014 in the Aorangi Forest Park (“Aorangi”) and is delineated by a vertical dashed line. Monitoring at a reference site occurred in the Remutaka Range (“Remutaka”). NA - counts not conducted that year. Count sample sizes, means and standard errors for each forest bird species are calculated from proportions of 30, ten-second subsamples within continuous five-minute bird counts that were collected using automated recorders. | | | | | | | | | | | |
| --- | --- | --- | --- | --- | --- | --- | --- | --- | --- | --- | --- |
|  | **Site** | 2012-13 |  | 2013-14 |  | 2014-15 |  | 2015-16 |  | 2016-17 |  |
|  |  | mean | ± SE | mean | ± SE | mean | ± SE | mean | ± SE | mean | ± SE |
| Sample sizes | Aorangi | 54 |  | 45 |  | 46 |  | 48 |  | 116 |  |
|  | Remutaka | NA |  | 30 |  | 51 |  | 42 |  | 107 |  |
| Pigeon | Aorangi | 0.020 | 0.012 | 0.005 | 0.003 | 0.006 | 0.003 | 0.032 | 0.012 | 0.018 | 0.005 |
|  | Remutaka | NA | NA | 0.010 | 0.005 | 0.020 | 0.016 | 0.015 | 0.006 | 0.007 | 0.002 |
| Tui | Aorangi | 0.125 | 0.029 | 0.135 | 0.038 | 0.067 | 0.029 | 0.199 | 0.043 | 0.155 | 0.022 |
|  | Remutaka | NA | NA | 0.277 | 0.066 | 0.309 | 0.041 | 0.246 | 0.051 | 0.158 | 0.023 |
| Bellbird | Aorangi | 0.132 | 0.036 | 0.110 | 0.037 | 0.213 | 0.045 | 0.344 | 0.061 | 0.228 | 0.027 |
|  | Remutaka | NA | NA | 0.133 | 0.049 | 0.076 | 0.029 | 0.082 | 0.021 | 0.035 | 0.012 |
| Whitehead | Aorangi | 0.160 | 0.044 | 0.183 | 0.052 | 0.323 | 0.054 | 0.269 | 0.057 | 0.358 | 0.036 |
|  | Remutaka | NA | NA | 0.086 | 0.045 | 0.042 | 0.025 | 0.041 | 0.020 | 0.064 | 0.019 |
| Silvereye | Aorangi | 0.452 | 0.047 | 0.184 | 0.038 | 0.288 | 0.044 | 0.079 | 0.023 | 0.279 | 0.027 |
|  | Remutaka | NA | NA | 0.262 | 0.064 | 0.205 | 0.035 | 0.173 | 0.046 | 0.214 | 0.023 |
| Tomtit | Aorangi | 0.146 | 0.033 | 0.170 | 0.043 | 0.303 | 0.053 | 0.233 | 0.040 | 0.191 | 0.024 |
|  | Remutaka | NA | NA | 0.414 | 0.065 | 0.246 | 0.046 | 0.210 | 0.046 | 0.296 | 0.033 |
| Fantail | Aorangi | 0.143 | 0.036 | 0.039 | 0.017 | 0.101 | 0.031 | 0.006 | 0.006 | 0.047 | 0.013 |
|  | Remutaka | NA | NA | 0.180 | 0.053 | 0.212 | 0.046 | 0.094 | 0.031 | 0.173 | 0.026 |
| Rifleman | Aorangi | 0.040 | 0.023 | 0.036 | 0.021 | 0.037 | 0.021 | 0.045 | 0.022 | 0.026 | 0.011 |
|  | Remutaka | NA | NA | 0.098 | 0.048 | 0.042 | 0.024 | 0.008 | 0.006 | 0.035 | 0.010 |
| Grey warbler | Aorangi | 0.110 | 0.020 | 0.118 | 0.023 | 0.051 | 0.012 | 0.082 | 0.024 | 0.115 | 0.014 |
|  | Remutaka | NA | NA | 0.128 | 0.027 | 0.132 | 0.026 | 0.121 | 0.028 | 0.079 | 0.011 |
| Blackbird | Aorangi | 0.357 | 0.054 | 0.227 | 0.053 | 0.205 | 0.053 | 0.075 | 0.026 | 0.214 | 0.031 |
|  | Remutaka | NA | NA | 0.063 | 0.029 | 0.222 | 0.053 | 0.159 | 0.047 | 0.105 | 0.022 |
| Chaffinch | Aorangi | 0.132 | 0.033 | 0.138 | 0.039 | 0.114 | 0.033 | 0.059 | 0.024 | 0.156 | 0.027 |
|  | Remutaka | NA | NA | 0.096 | 0.047 | 0.148 | 0.044 | 0.006 | 0.006 | 0.038 | 0.013 |


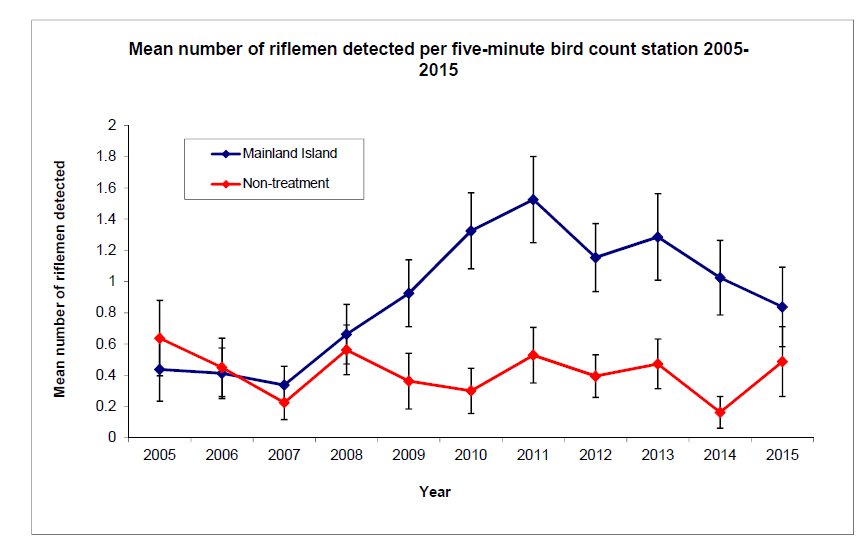


**Appendix S15a.**


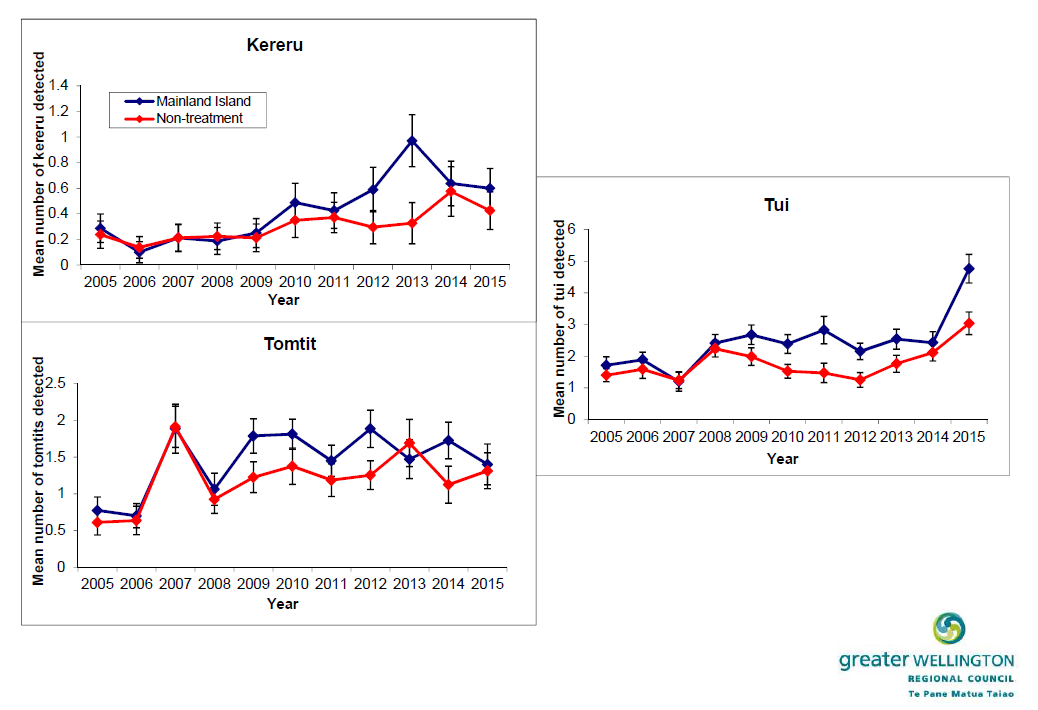


**Appendix S15b.**


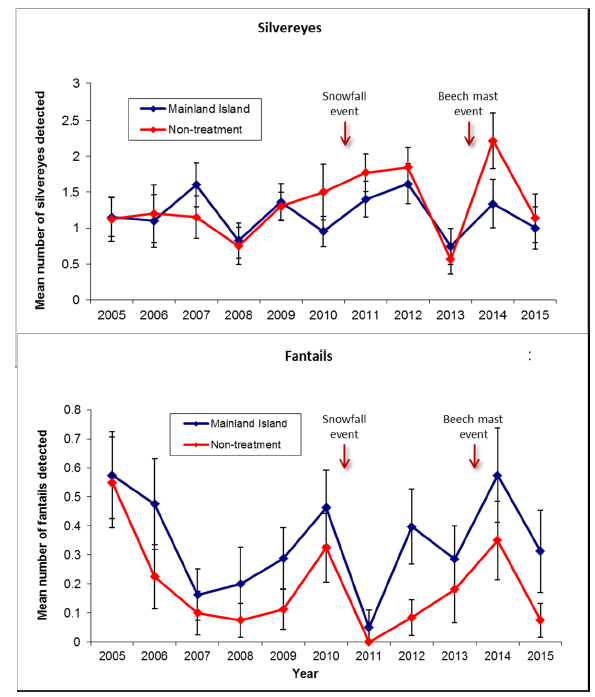


**Appendix S15c.**


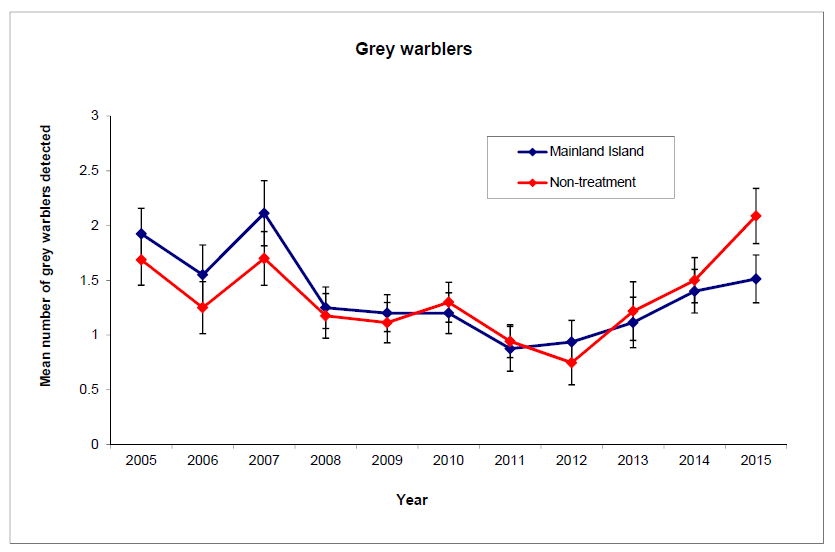


**Appendix S15d.**


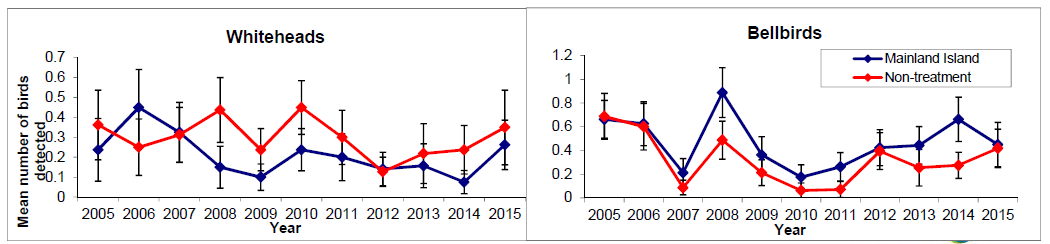


**Appendix S15f.**

**Appendix S15.** Presentation of the mean responses (and standard errors) for nine native bird species in the Wainuiomata Mainland Island (Wellington, New Zealand) project managed by Philippa Crisp (Greater Wellington Regional Council). These figures were presented at the Ecological Society of New Zealand conference, Hamilton, New Zealand, November 2016. Summaries acquired through personal communication 19-12-2016. Kererū is the Māori name for the New Zealand Pigeon.
