# Supplementary material for: Responses of New Zealand forest birds to management of introduced mammals
Source: Conserv Biol. 2020 Mar 23;35(1):35–49. doi: 10.1111/cobi.13456 (PMC7984369; doi:10.1111/cobi.13456)
Supplement: Supplementary file 9 — Supporting Material [file COBI-35-35-s008.pdf]

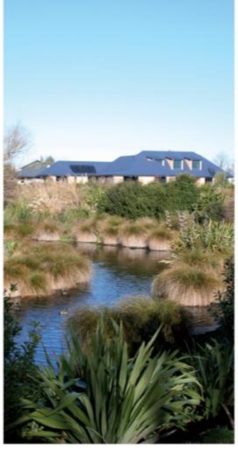
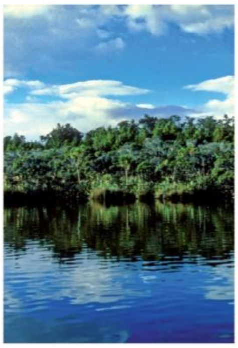
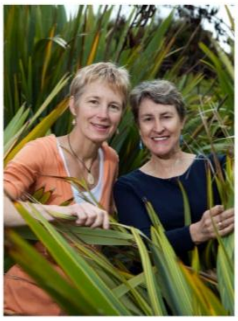
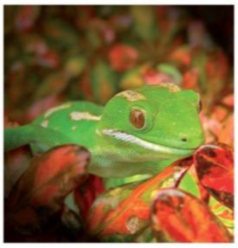

Hamilton City biennial bird counts: 2004–2012

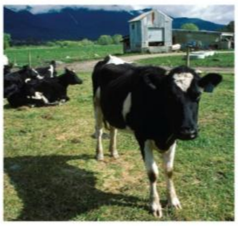
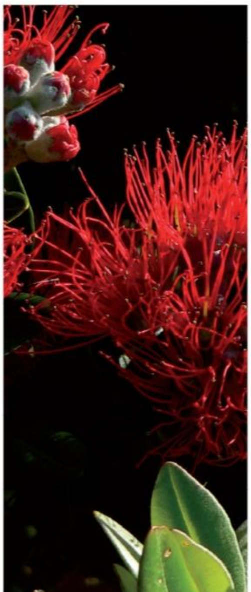
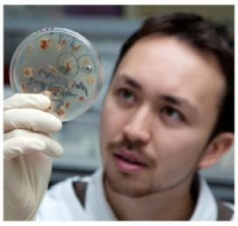
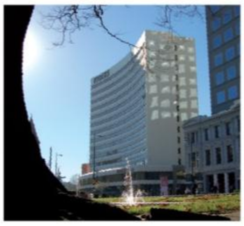
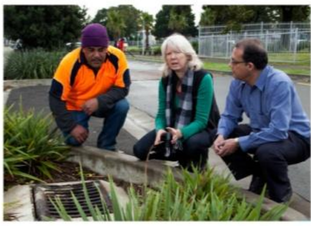

Landcare Research
Manaaki Whenua

Hamilton City biennial bird counts: 2004–2012

Neil Fitzgerald, John Innes

Landcare Research

Prepared for:

Hamilton City Council

Private Bag 3010
Hamilton 3240
New Zealand

June 2013

Landcare Research, Gate 10 Silverdale Road, University of Waikato Campus, Private Bag 3127, Hamilton 3240, New Zealand, Ph +64 7 859 3700, Fax +64 7 859 3701, www.landcareresearch.co.nz

Reviewed by:	*Approved for release by:*
Mark Smale<br>Ecologist<br>Landcare Research	Bill Lee<br>Portfolio Leader<br>Managing Biodiversity
Landcare Research Contract Report:	LC 1484

Disclaimer

This report has been prepared by Landcare Research for Hamilton City Council. If used by other parties, no warranty or representation is given as to its accuracy and no liability is accepted for loss or damage arising directly or indirectly from reliance on the information in it.

TELARC SAI registered

ISO 14001

© Landcare Research New Zealand Ltd 2013

No part of this work covered by copyright may be reproduced or copied in any form or by any means (graphic, electronic or mechanical, including photocopying, recording, taping, information retrieval systems, or otherwise) without the written permission of the publisher.

Contents

Summary v

1 Introduction 1

2 Background 1

3 Objective 3

4 Methods 3

4.1 Five-minute counts 3

4.2 Slow-walk transects 4

5 Results 6

5.1 Five-minute counts 6

5.2 Slow-walk transects 12

6 Discussion and conclusions 13

7 Recommendations 15

8 Acknowledgements 15

9 References 15

Appendix 1 – Scientific names and classification of birds referred to in text 17

Appendix 2 – Comparative photographs of Waiwhakareke transects in 2006 and 2012 18

Summary

Project and Client

- This report describes the fifth set of biennial bird counts, initiated in 2004, in green (parkland and vegetated gullies) and residential areas of Hamilton City, undertaken in August (winter) and November (spring) 2012. These counts enable ongoing assessment of biodiversity that can be used by Hamilton City Council (HCC) as an environmental sustainability indicator. This work is funded jointly by HCC and Landcare Research.

Objective

- To determine changes in bird abundance in green and residential parts of Hamilton City since 2004.

Methods

- Five-minute counts of terrestrial birds were undertaken at 101 stations green areas in August and November 2012, and at 106 stations in residential areas in Hamilton in November 2012.
- Terrestrial birds were counted in ten slow-walk transects in five green areas in Hamilton in November 2012.
- The same count stations and transects have been counted since 2004.

Results

- More bird species were recorded in green areas (27) than residential areas (17) in 2012.
- Twice as many native bird species were recorded in green areas (10) than residential areas (5) in 2012.
- The abundance of tūī in Hamilton increased significantly between 2004 and 2012 in all areas and seasons and with both survey techniques. Mean abundance in 5-minute counts in green areas increased from 0.03 to 0.49 from 2004 to 2012 in August, and from 0 to 0.48 in November. Mean abundance in 5-minute counts in residential areas in November increased from 0 to 0.09 from 2004 to 2012. Mean abundance in slow-walk transects in green areas in November increased from 0 to 0.3 birds per hectare from 2004 to 2012.
- Blackbirds decreased significantly in green areas between 2004 and 2012 in both winter and spring.
- Two native species – kākā and pied stilt – were recorded for the first time in counts in 2012.

Conclusions

- The general patterns of bird abundance and distribution in green (urban parks and gullies) and residential areas of Hamilton remain the same as previously recorded in 2004–2010. Forty-seven percent more species and twice as many native species were found in green areas than in residential areas.
- Since 2004 tūī have increased in abundance and distribution in Hamilton in both the breeding and non-breeding seasons. Detailed research of tūī movement and nesting behaviour suggests that the increases are primarily as a result of pest control in remnant forests surrounding the city since 2007.
- Tūī populations are likely to continue to increase if predator control is maintained in surrounding forests and the current pest control in the city increased to protect the tūī now breeding there.

Recommendations

- These biennial bird counts should be continued (next in 2014) to monitor changes in bird abundance in various parts of Hamilton, in particular to monitor the response of tūī, bellbirds, and New Zealand pigeon to ongoing pest control in and near the city.
- The current effective control of possums and ship rats by Waikato Regional Council in forest areas surrounding Hamilton should continue to increase tūī abundance in the city, and an emphasis on areas known to contain bellbirds and New Zealand pigeon should similarly increase the numbers of these species in Hamilton.
- To protect nesting tūī in the city, effective control of possums and ship rats should be continued and expanded by Hamilton City Council in urban parks such as Donny Park, Hamilton Gardens, Hamilton East Cemetery, Jubilee Park, Hammond Park, and other riverside reserves.
- Plant species that are beneficial to native birds should continue to be planted in urban parks by Hamilton City Council.
- Information on best-practice pest control and advice on planting to encourage native birds should continue to be made available to private landowners wishing to improve their properties for birds.

1 Introduction

This report describes bird counts undertaken in green (parkland and vegetated gullies) and residential areas of Hamilton City, in August and November 2012. Results are compared with earlier surveys carried out biennially since 2004, particularly as regards changes in the distribution and observed abundance of tūī (common and scientific names of birds are given in Appendix 1).

2 Background

Innes and Burns (2004) suggested that Hamilton City Council (HCC) monitor birds as a biodiversity indicator of progress towards environmental sustainability in Hamilton (Hamilton City Council 2002). This is one of many measures that enable HCC to meet its vision for city sustainability, originating from 'Agenda 21' and the 1992 Rio de Janeiro Earth Summit (United Nations Conference on Environment and Development, 3–14 June 1992).

Bird counts undertaken in 2004 showed that Hamilton had a diverse terrestrial avifauna (23 species), but it comprised mainly exotic and widespread common native birds (Innes et al. 2005a). Key iconic native birds such as New Zealand pigeon, tūī and bellbird, although widespread and relatively abundant in large (>1000 ha) native forests in the Waikato (Robertson et al. 2007), were rare or absent in the city. At that time tūī were of special interest in the Waikato because they are uncommon there compared with many other parts of New Zealand (Robertson et al. 2007), and their abundance, diet, movements, and nesting success in the region were studied by Landcare Research between 2002 and 2007. This work showed that tūī typically visited Hamilton to feed on nectar, mainly from exotic winter flowering plants such as coastal banksia (*Banksia integrifolia*), and in spring moved to forested areas 7–20 km away, where they breed. In the absence of intensive pest control, nesting success is poor (Innes et al. 2005b). Subsequently, Waikato Regional Council began Hamilton Halo, a programme of intensive control of possums (*Trichosurus vulpecula*) and ship rats (*Rattus rattus*) to low abundance in areas of native forest surrounding Hamilton (Figure 1), with the aim of improving breeding success of tūī that would subsequently visit the city.

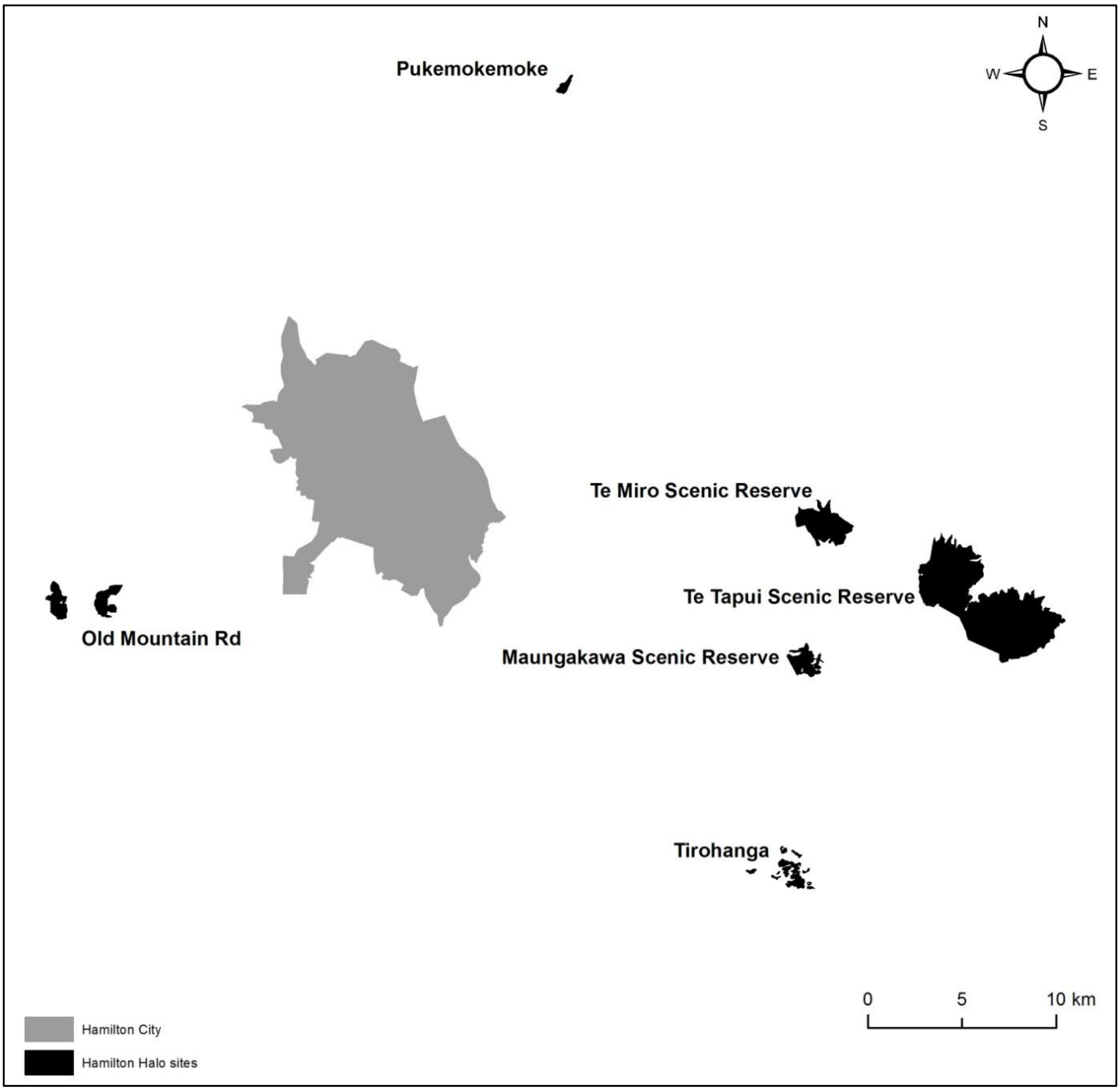

Figure 1 Location of Hamilton Halo sites were ship rats and possums are controlled by Waikato Regional Council.

Sampling involved bird counts in August (late winter) to record winter visitors, and again in November (late spring) to record birds breeding in urban areas.

3 Objective

To determine changes in bird abundance in green and residential parts of Hamilton City since 2004.

4 Methods

Locations used in earlier counts were resampled using the same methodology (Innes et al. 2005a, 2008; Fitzgerald & Innes 2010, 2012). All counts were undertaken in two distinct urban environments: 'green' areas comprising amenity parks and vegetated gullies (Morgan et al. 2009), and 'residential' areas of mostly private gardens and streets (Figure 2). Residential counts were made from public footpaths and located by address and GPS. Green count stations were located by GPS.

Two different bird count techniques were used: 5-minute counts and slow-walk transects. Counts were conducted by Neil Fitzgerald and Scott Bartlam of Landcare Research, Hamilton.

4.1 Five-minute counts

The 5-minute bird count technique (Dawson & Bull 1975) was selected to allow comparisons with many other counts made around the Waikato and New Zealand. During each 5-minute count, terrestrial birds seen or heard within 100 m of the stationary observer are recorded. The technique does not determine absolute density of birds, but provides repeatable indices of abundance, provided that counts are made by experienced observers at the same time of year in conditions of little or no wind or rain (Innes & Burns 2004; Hartley 2012).

In 2004 we established count stations at least 200 m apart along lines through green and residential areas. These locations were selected to be representative of these zones in the city. Main roads were avoided when the stations were established to minimise traffic noise, and we avoided counting during moderate or strong winds or rain.

All green stations were counted in late winter (6–23 August), and all green and residential stations were counted in late spring (7–27 November) 2012. Resource limitations meant we have not undertaken counts residential areas in winter. Birds were counted between 0800 and 1700 h in August and between 0800 and 1830 h in November to avoid significant changes in conspicuousness that are likely to occur near sunrise and sunset. The number of stations counted differed slightly in some years due to flooding and other issues (Table 1).

Table 1 Number of 5-minute bird count stations counted in each area, season and year

	Green		Residential
Year	August	November	November
2004	101	101	106
2006	99	99	106
2008	96	99	106
2010	101	101	106
2012	101	101	106

Bird count data were log10-transformed and compared between years using the Linear Mixed Effects models procedure in the R statistical computing environment (R Core team 2013). Time (year) was taken as a fixed effect and observation station was considered a random effect. Three models (random intercepts, random intercepts with auto-correlation, and random slopes and intercepts) were tested for each species and the Akaike Information Criterion (AIC) was used to determine which model was best supported by the data. Model slopes were back-transformed onto the natural scale to give an annual percentage change in bird abundance. No compensation was made for multiple comparisons. We tested for differences between years in the proportion of count stations where tūī were recorded using chi-squared tests for equal proportions.

4.2 Slow-walk transects

For slow-walk transect counts, all terrestrial birds within 10 m of the observer's path are recorded while slowly walking a 500 m-long line (Lovegrove 1988). Two transects were counted at each of five green sites during November only. Eight of the ten transects are located in areas where 5-minute counts are also conducted, allowing comparisons of the methods to be made. The transects give an estimate of absolute density of birds in some small areas (unlike 5-minute counts) but describe bird distribution poorly because there are few transects. Each transect was counted three times (no more than once per day) between 7 and 30 November 2012, and the mean of these replicates calculated for each species. Assuming all birds within 10 m of the transect line are detected, the count result is effectively the number of birds counted per hectare.

Mean transect counts were log10-transformed and compared between years using the Linear Mixed Effects models procedure in the R statistical computing environment (version 2.13.0). Time (year) was taken as a fixed effect and transect was considered a random effect. Two models (random intercepts, and random intercepts with auto-correlation) were tested for each species and the AIC used to determine which model was best supported by the data. Model slopes were back-transformed onto the natural scale to give an annual percentage change in bird density.

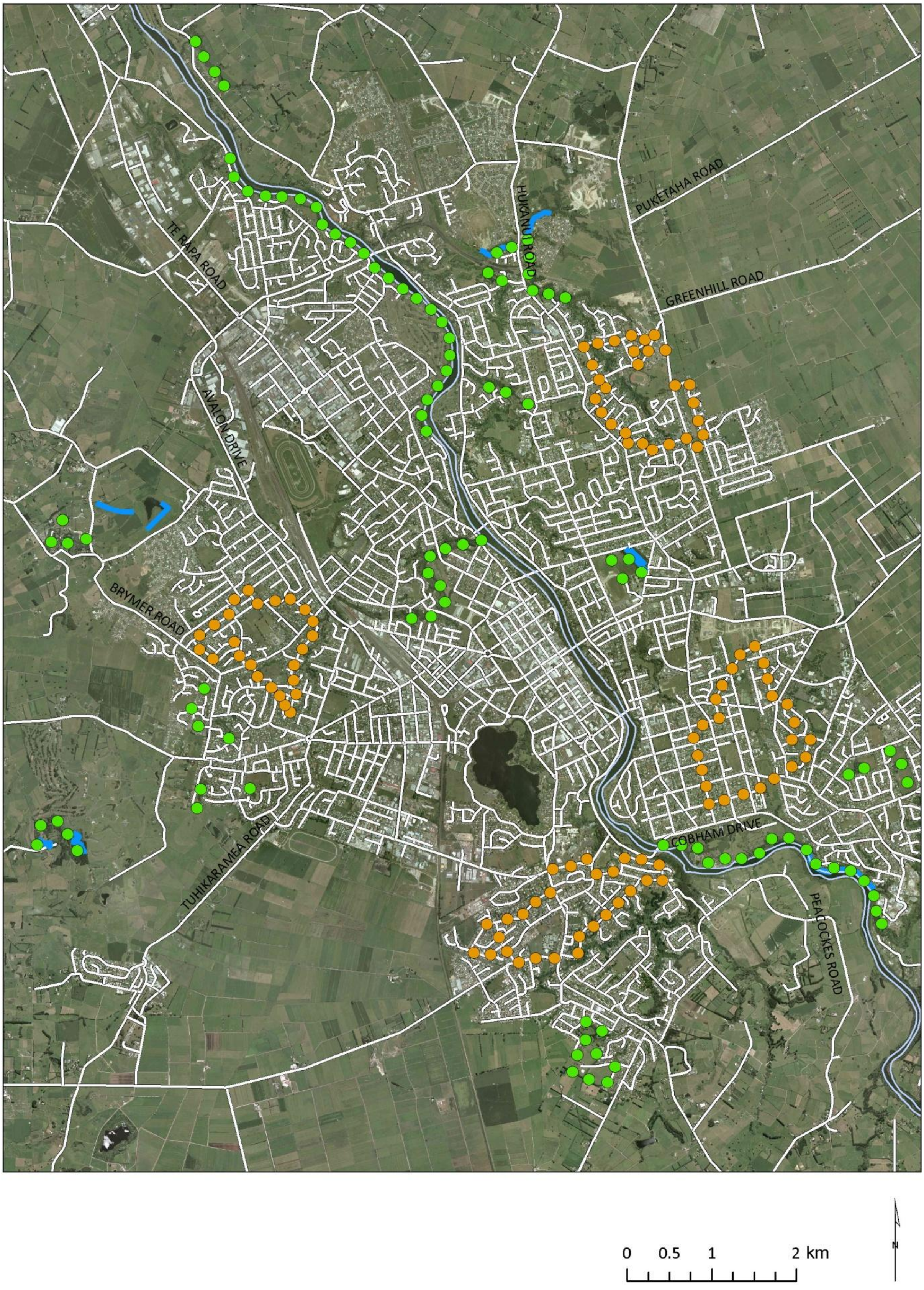

Figure 2 Location of residential (●) and green (●) 5-minute bird count stations and slow walk transects (▬) in Hamilton City.

5 Results

5.1 Five-minute counts

Twenty-nine terrestrial bird species were recorded in 5-minute counts in 2012, including one species, kākā, recorded for the first time. In total, 32 species were recorded in one or more of the survey years and in 2012, as in previous years, more species were recorded in green areas (27 species, Table 2) than in residential areas (17 species, Table 3).

The six most abundant bird species in green areas in November were the same across years, and only one of these (silvereye) is native. The three most abundant species in these counts were all introduced species and in the same (decreasing) order: house sparrow, blackbird, and goldfinch.

Four species were consistently the most abundant in green areas in August, and in 2012 these species were recorded in the same order of decreasing abundance as in November 2012 counts in green areas, i.e. house sparrow, blackbird, goldfinch, and silvereye.

From 2006 to 2012, six species (house sparrow, common starling, blackbird, common myna, silvereye, and goldfinch) were consistently the most abundant in residential areas. The top three of these were in the same order of decreasing abundance in all years.

Tūī was the 11th most abundant species in green areas in both August and November 2012, which is the highest ranking for tūī in either month since these counts began in 2004. Tūī were also recorded for the first time in residential areas in November 2012.

There were statistically significant ($p < 0.05$) trends in counts of 14 different species in one or more of the areas or seasons, meaning the counts are likely to reflect real changes in abundance during the study period. One of these species, blackbird, decreased significantly in both areas and seasons, while tūī increased significantly in both areas and seasons. The count decline in blackbirds if expressed as an annual change was –1.8 %.y^{-1} in green areas in August, –2.9 %.y^{-1} in green areas in November, and –1.9 %.y^{-1} in residential areas. On the same basis tūī increased at 2.8 %.y^{-1} in green counts in August, and 2.9 %.y^{-1} in green counts in November. The increase in tūī in residential areas reflects the first appearance of tūī in these November counts.

Another measure of the trend of increasing numbers of tūī in Hamilton is the increase in number and distribution of stations at which they were recorded (Table 4, Figure 3 & 4). The proportion of count stations at which tūī were recorded was similar in green areas in August 2012 and 2010, but was significantly higher in November counts in green and residential areas than in any previous year ($\chi^2 = 99.99$, d.f. = 4, $p < 0.001$). The mean number of tūī per station was similar in August (0.49) and November (0.48) in 2012, but tūī were at fewer ($\chi^2 = 4.18$, d.f. = 1, p = 0.04) stations in August (21/101) than in November (35/101), indicating greater aggregation in winter when tūī were probably forraging in family groups (Bergquist 1985).

Table 2 Mean numbers of terrestrial birds counted per 5-minute count station in Hamilton green areas in August and November. Figures in bold indicate a significant change (↑ = increase, ↓ = decrease) in mean count over the 2004–2012 period ($p < 0.05$). Common names follow Gill et al. (2010). Native species are italicised

	**August**						**November**					
	**2004**	**2006**	**2008**	**2010**	**2012**		**2004**	**2006**	**2008**	**2010**	**2012**	
Australian magpie	0.08 (0.04)	0.04 (0.02)	0.15 (0.05)	0.07 (0.03)	0.06 (0.03)		0.08 (0.04)	0.11 (0.06)	0.15 (0.06)	0.08 (0.05)	0.10 (0.05)	
Barbary dove	0 (0)	0 (0)	0 (0)	0 (0)	0 (0)		0 (0)	0 (0)	0.01 (0.01)	0 (0)	0 (0)	
Blackbird	**3.1 (0.27)**	**2.81 (0.26)**	**3.01 (0.35)**	**2.81 (0.37)**	**2.55 (0.26)**	↓	**3.54 (0.32)**	**2.86 (0.23)**	**3.1 (0.3)**	**3.1 (0.29)**	**2.13 (0.18)**	↓
California quail	0 (0)	0 (0)	0.01 (0.01)	0.01 (0.01)	0 (0)		0 (0)	0 (0)	0.01 (0.01)	0.01 (0.01)	0 (0)	
Chaffinch	1.00 (0.13)	0.66 (0.09)	0.85 (0.13)	0.69 (0.09)	0.66 (0.08)		0.78 (0.1)	0.86 (0.1)	1.03 (0.1)	0.81 (0.09)	0.91 (0.09)	
Common myna	0.63 (0.09)	0.34 (0.09)	0.51 (0.1)	0.44 (0.07)	0.52 (0.11)		0.49 (0.08)	0.44 (0.08)	0.55 (0.1)	0.51 (0.08)	0.54 (0.08)	
Common pheasant	**0 (0)**	**0 (0)**	**0 (0)**	**0.01 (0.01)**	**0.05 (0.02)**	↑	0.06 (0.02)	0.07 (0.03)	0.06 (0.02)	0.08 (0.03)	0.07 (0.03)	
Common redpoll	0 (0)	0.01 (0.01)	0 (0)	0 (0)	0 (0)		0 (0)	0 (0)	0 (0)	0 (0)	0 (0)	
Common starling	0.71 (0.19)	0.75 (0.19)	1.04 (0.16)	1.01 (0.16)	0.52 (0.1)		**1.72 (0.2)**	**1.79 (0.23)**	**1.44 (0.17)**	**1.35 (0.18)**	**1.01 (0.1)**	↓
Dunnock	0.03 (0.02)	0.01 (0.01)	0.02 (0.01)	0 (0)	0 (0)		0 (0)	0.01 (0.01)	0 (0)	0.01 (0.01)	0 (0)	
Eastern rosella	0.06 (0.03)	0.13 (0.04)	0.12 (0.05)	0.1 (0.04)	0.05 (0.02)		**0.01 (0.01)**	**0.07 (0.04)**	**0.15 (0.04)**	**0.1 (0.04)**	**0.1 (0.03)**	↑
Goldfinch	3.45 (1.6)	3.61 (0.84)	1.45 (0.28)	2.05 (0.68)	1.86 (0.48)		**2.55 (0.27)**	**2.22 (0.32)**	**2.12 (0.2)**	**1.45 (0.17)**	**1.5 (0.14)**	↓
Greenfinch	0.3 (0.08)	0.21 (0.07)	0.19 (0.05)	0.29 (0.17)	0.35 (0.07)		**0.47 (0.07)**	**0.51 (0.08)**	**0.54 (0.09)**	**0.74 (0.11)**	**0.72 (0.08)**	↑
Grey warbler	*0.48 (0.06)*	*0.52 (0.07)*	*0.48 (0.07)*	*0.49 (0.07)*	*0.68 (0.07)*		***0.25 (0.06)***	***0.23 (0.06)***	***0.3 (0.05)***	***0.44 (0.09)***	***0.42 (0.06)***	↑
House sparrow	3.51 (0.41)	3.13 (0.38)	4.18 (0.57)	3.19 (0.46)	2.78 (0.33)		3.61 (0.38)	4.02 (0.44)	5.15 (0.47)	4.74 (0.4)	3.6 (0.38)	
Kākā	*0 (0)*	*0 (0)*	*0 (0)*	*0 (0)*	*0.03 (0.03)*		*0 (0)*	*0 (0)*	*0 (0)*	*0 (0)*	*0 (0)*	
NZ fantail	*0.99 (0.1)*	*0.77 (0.1)*	*0.67 (0.08)*	*0.77 (0.08)*	*1.02 (0.08)*		*0.55 (0.09)*	*0.62 (0.08)*	*0.74 (0.08)*	*0.53 (0.08)*	*0.79 (0.08)*	
NZ kingfisher	*0.22 (0.05)*	*0.16 (0.04)*	*0.18 (0.05)*	*0.19 (0.05)*	*0.3 (0.06)*		*0.59 (0.09)*	*0.35 (0.06)*	*0.34 (0.06)*	*0.37 (0.07)*	*0.55 (0.07)*	
NZ pigeon	*0 (0)*	*0 (0)*	*0.01 (0.01)*	*0 (0)*	*0 (0)*		*0 (0)*	*0 (0)*	*0.01 (0.01)*	*0 (0)*	*0.01 (0.01)*	
Pukeko	*0.46 (0.12)*	*0.22 (0.06)*	*0.35 (0.11)*	*0.47 (0.11)*	*0.25 (0.06)*		*0.11 (0.03)*	*0.17 (0.06)*	*0.09 (0.04)*	*0.19 (0.05)*	*0.2 (0.06)*	

	August						November					
	**2004**	**2006**	**2008**	**2010**	**2012**		**2004**	**2006**	**2008**	**2010**	**2012**	
Rock pigeon	**0.08 (0.07)**	**0.06 (0.04)**	**0.08 (0.06)**	**0.16 (0.07)**	**0.51 (0.36)**	↑	0.01 (0.01)	0.09 (0.06)	0.06 (0.04)	0.18 (0.14)	0.11 (0.04)	
Shining cuckoo	*0 (0)*	*0 (0)*	*0 (0)*	*0 (0)*	*0 (0)*		*0.05 (0.02)*	*0.01 (0.01)*	*0.09 (0.03)*	*0.15 (0.04)*	*0.06 (0.02)*	
Silvereye	***1.97 (0.18)***	***3.15 (0.72)***	***2.17 (0.28)***	***1.5 (0.21)***	***1.64 (0.27)***	↓	*1.6 (0.16)*	*1.42 (0.13)*	*1.34 (0.15)*	*1.16 (0.13)*	*1.38 (0.12)*	
Skylark	0.02 (0.01)	0.01 (0.01)	0.01 (0.01)	0.01 (0.01)	0.02 (0.01)		0 (0)	0 (0)	0.01 (0.01)	0.01 (0.01)	0.01 (0.01)	
Song thrush	0.42 (0.09)	0.55 (0.1)	0.49 (0.09)	0.42 (0.09)	0.39 (0.08)		0.65 (0.11)	0.69 (0.1)	0.8 (0.11)	0.51 (0.11)	0.47 (0.08)	
Spotted dove	**0 (0)**	**0.01 (0.01)**	**0.03 (0.02)**	**0.03 (0.02)**	**0.05 (0.03)**	↑	0.03 (0.02)	0.01 (0.01)	0.05 (0.03)	0.03 (0.02)	0.08 (0.03)	
Spur-winged plover	*0.08 (0.04)*	*0.05 (0.03)*	*0.06 (0.04)*	*0.07 (0.03)*	*0.03 (0.02)*		*0.05 (0.04)*	*0.02 (0.02)*	*0.02 (0.02)*	*0.02 (0.01)*	*0.06 (0.04)*	
Swamp harrier	*0.01 (0.01)*	*0.03 (0.02)*	*0.03 (0.03)*	*0.02 (0.01)*	*0.04 (0.02)*		***0.03 (0.02)***	***0.04 (0.02)***	***0.03 (0.02)***	***0.01 (0.01)***	***0 (0)***	↓
Tūī	***0.03 (0.02)***	***0.06 (0.03)***	***0.11 (0.04)***	***0.38 (0.09)***	***0.49 (0.17)***	↑	***0 (0)***	***0.04 (0.02)***	***0.05 (0.02)***	***0.03 (0.02)***	***0.48 (0.07)***	↑
Welcome swallow	*0.42 (0.09)*	*0.37 (0.08)*	*0.14 (0.05)*	*0.26 (0.06)*	*0.33 (0.07)*		*0.41 (0.08)*	*0.53 (0.11)*	*0.49 (0.09)*	*0.35 (0.08)*	*0.46 (0.13)*	
White-faced heron	*0 (0)*	*0.01 (0.01)*	*0.01 (0.01)*	*0.02 (0.01)*	*0 (0)*		*0.03 (0.02)*	*0 (0)*	*0 (0)*	*0 (0)*	*0 (0)*	
Yellowhammer	0.11 (0.09)	0.02 (0.02)	0.06 (0.05)	0.06 (0.04)	0.08 (0.04)		0.01 (0.01)	0.07 (0.03)	0.03 (0.02)	0.03 (0.02)	0.02 (0.01)	
Total number of species recorded	23	26	27	26	25		24	25	28	27	25	
Number of introduced species	14	16	16	16	15		14	15	17	17	15	
Number of native species	9	10	11	10	10		10	10	11	10	10	

Table 3 Mean numbers of terrestrial birds counted per 5-minute count station in Hamilton residential areas in November. Figures in bold indicate a significant change (↑ = increase, ↓ = decrease) in mean count over the 2004–2012 period ($p < 0.05$). Common names follow Gill et al. (2010). Native species are italicised

	**November**					
	**2004**	**2006**	**2008**	**2010**	**2012**	
Australian magpie	0 (0)	0.08 (0.05)	0.03 (0.02)	0.03 (0.02)	0.03 (0.02)	
Barbary dove	0 (0)	0 (0)	0 (0)	0 (0)	0 (0)	
Blackbird	**2.4 (0.18)**	**2.29 (0.17)**	**2.43 (0.15)**	**2.3 (0.17)**	**1.72 (0.13)**	↓
California quail	0 (0)	0 (0)	0 (0)	0 (0)	0 (0)	
Chaffinch	**0.23 (0.06)**	**0.14 (0.04)**	**0.18 (0.04)**	**0.13 (0.04)**	**0.03 (0.02)**	↓
Common myna	1.22 (0.11)	1.31 (0.12)	1.21 (0.11)	1.44 (0.13)	1.19 (0.12)	
Common pheasant	0 (0)	0 (0)	0 (0)	0 (0)	0 (0)	
Common redpoll	0 (0)	0 (0)	0 (0)	0 (0)	0 (0)	
Common starling	**3.1 (0.2)**	**3.16 (0.2)**	**2.66 (0.28)**	**3.61 (0.22)**	**2.37 (0.2)**	↓
Dunnock	0 (0)	0 (0)	0 (0)	0 (0)	0 (0)	
Eastern rosella	0 (0)	0 (0)	0.08 (0.05)	0 (0)	0.04 (0.02)	
Goldfinch	0.8 (0.1)	0.96 (0.12)	1.01 (0.13)	0.79 (0.12)	0.83 (0.11)	
Greenfinch	**0.1 (0.03)**	**0.1 (0.04)**	**0.19 (0.05)**	**0.25 (0.06)**	**0.24 (0.05)**	↑
Grey warbler	*0 (0)*	*0.02 (0.01)*	*0.01 (0.01)*	*0 (0)*	*0 (0)*	
House sparrow	8.84 (0.48)	9.32 (0.53)	9.33 (0.38)	10.59 (0.47)	7.45 (0.29)	
Kākā	*0 (0)*	*0 (0)*	*0 (0)*	*0 (0)*	*0 (0)*	
NZ fantail	*0.07 (0.03)*	*0.04 (0.02)*	*0.03 (0.02)*	*0.02 (0.01)*	*0.11 (0.03)*	
NZ kingfisher	*0.05 (0.02)*	*0 (0)*	*0.05 (0.02)*	*0.01 (0.01)*	*0 (0)*	
NZ pigeon	*0 (0)*	*0 (0)*	*0 (0)*	*0 (0)*	*0 (0)*	
Pukeko	*0 (0)*	*0.03 (0.03)*	*0 (0)*	*0 (0)*	*0 (0)*	
Rock pigeon	**0.15 (0.08)**	**0.27 (0.11)**	**0.7 (0.23)**	**0.77 (0.27)**	**0.77 (0.25)**	↑
Shining cuckoo	*0 (0)*	*0 (0)*	*0 (0)*	*0 (0)*	*0.02 (0.01)*	
Silvereye	*1.34 (0.18)*	*1.27 (0.14)*	*1.19 (0.14)*	*1.16 (0.14)*	*1.07 (0.11)*	
Skylark	0 (0)	0 (0)	0 (0)	0 (0)	0 (0)	
Song thrush	**0.35 (0.06)**	**0.19 (0.04)**	**0.2 (0.05)**	**0.21 (0.05)**	**0.15 (0.03)**	↓
Spotted dove	0 (0)	0 (0)	0 (0)	0 (0)	0.02 (0.01)	
Spur-winged plover	*0.02 (0.02)*	*0 (0)*	*0.05 (0.05)*	*0 (0)*	*0 (0)*	
Swamp harrier	*0 (0)*	*0 (0)*	*0 (0)*	*0 (0)*	*0 (0)*	
Tūī	***0 (0)***	***0 (0)***	***0 (0)***	***0 (0)***	***0.09 (0.03)***	↑
Welcome swallow	*0.12 (0.04)*	*0.27 (0.07)*	*0.15 (0.04)*	*0.19 (0.06)*	*0.17 (0.05)*	
White-faced heron	*0 (0)*	*0 (0)*	*0 (0)*	*0 (0)*	*0 (0)*	
Yellowhammer	0 (0)	0.01 (0.01)	0.01 (0.01)	0 (0)	0 (0)	
Total number of species recorded	14	16	18	14	17	
Number of introduced species	9	11	12	10	12	
Number of native species	5	5	6	4	5	

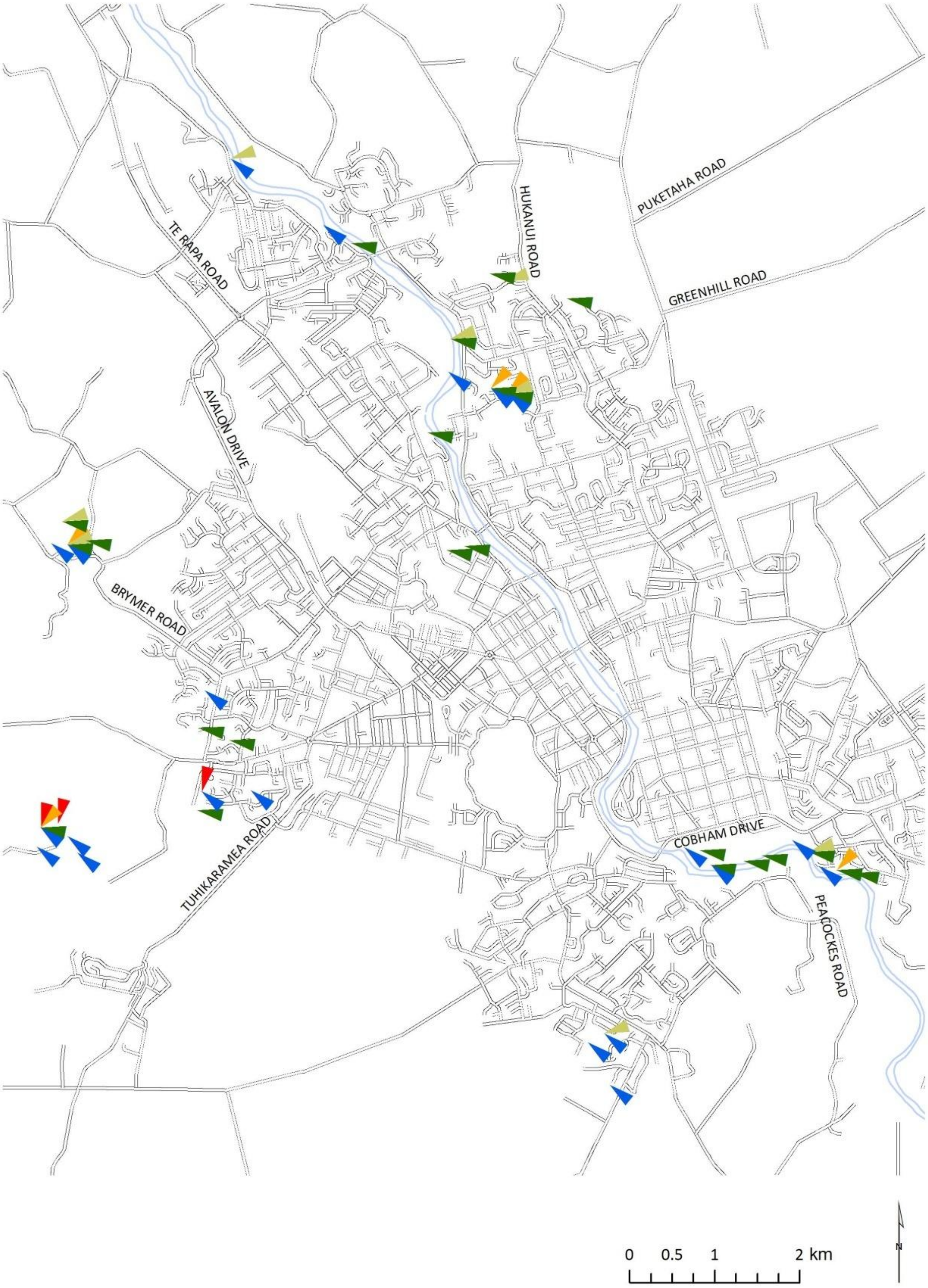

Figure 3 Locations of 5-minute bird count stations in Hamilton City at which tūī were recorded in August 2004 ( ), 2006 ( ), 2008 ( ), 2010 ( ), and 2012 ( ).

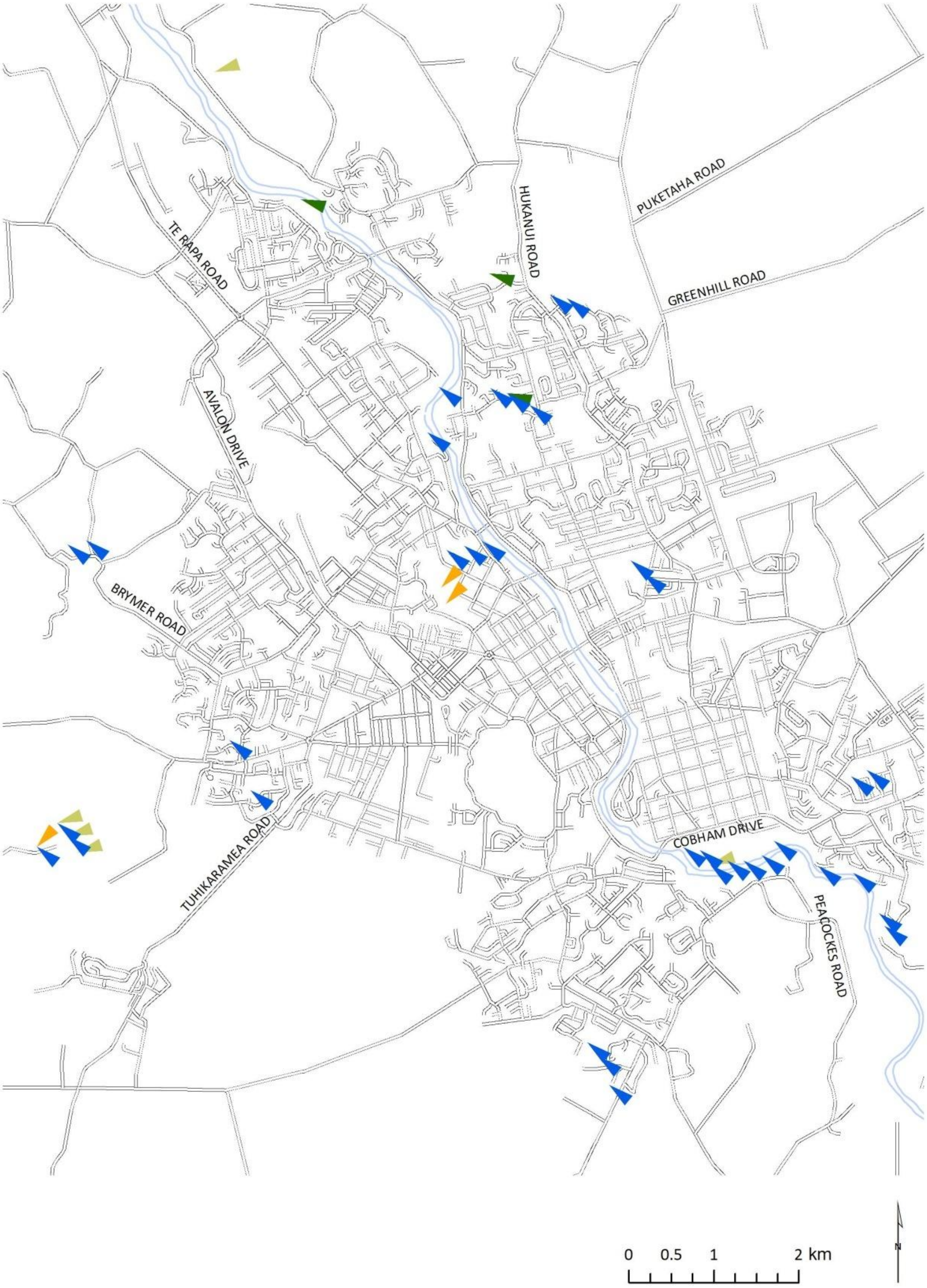

Figure 4 Locations of 5-minute bird count stations in Hamilton City at which tūī were recorded in November 2006 (▲), 2008 (▲), 2010 (▲), and 2012 (▲).

Table 4 Proportion of 5-minute bird count stations at which tūī were recorded

	2004	2006	2008	2010	2012
Green, August	0.03	0.05	0.08	0.23	0.21
Green, November	0.00	0.03	0.05	0.03	0.35
Residential, November	0.00	0.00	0.00	0.00	0.08

5.2 Slow-walk transects

Seventeen species of terrestrial birds were recorded in slow-walk transects in 2012, including one species, pied stilt, recorded for the first time (Table 5). This is 8 species less than were recorded in 5-minute counts in green areas during the same month. In total, 22 species were recorded in slow-walk transects in one or more of the survey years.

Table 5 Mean numbers of terrestrial birds (birds per hectare) counted per slow-walk transect in Hamilton green areas in November. Figures in bold indicate a significant change (↑ = increase, ↓ = decrease) in mean count over the 2004–2012 period ($p<0.05$). Common names follow Gill et al. (2010). Native species are italicised

	2004	2006	2008	2010	2012	
Australian magpie	0.1 (0.1)	0.1 (0.1)	0	0	0	
Blackbird	5.1 (0.5)	4.9 (0.6)	5.2 (0.7)	5 (0.4)	3.7 (0.5)	
Chaffinch	1.8 (0.4)	2.1 (0.3)	2.1 (0.3)	2 (0.3)	1.4 (0.2)	
Common myna	0.4 (0.2)	0.3 (0.1)	1.1 (0.3)	0.1 (0)	0.3 (0.1)	
Common starling	1.2 (0.3)	2.1 (0.9)	3.5 (1.5)	0.8 (0.3)	0.6 (0.3)	
Eastern rosella	**0.3 (0.1)**	**0.3 (0.1)**	**0.2 (0.1)**	**0.1 (0.1)**	**0.1 (0.1)**	↓
Goldfinch	4.1 (1.1)	3.7 (0.9)	3.1 (0.6)	2.1 (0.3)	2.2 (0.5)	
Greenfinch	**0.7 (0.2)**	**0.7 (0.2)**	**0.6 (0.2)**	**0.4 (0.1)**	**0.3 (0.2)**	↓
Grey warbler	***0.7 (0.2)***	***0.9 (0.2)***	***0.6 (0.2)***	***0.9 (0.2)***	***0.3 (0.1)***	↓
House sparrow	**5.4 (1)**	**5.6 (1.2)**	**6.5 (1)**	**5.4 (1.3)**	**3.6 (0.7)**	↓
NZ fantail	***2.5 (0.4)***	***1.3 (0.3)***	***1.4 (0.2)***	***1.4 (0.3)***	***0.7 (0.2)***	↓
NZ kingfisher	***0.5 (0.1)***	***0.3 (0.1)***	***0.6 (0.2)***	***0.1 (0.1)***	***0.2 (0.1)***	↓
Pied stilt	*0*	*0*	*0*	*0*	*0.1 (0.1)*	
Pukeko	*0.8 (0.3)*	*0.5 (0.2)*	*0.6 (0.3)*	*0.5 (0.2)*	*0.7 (0.2)*	
Rock pigeon	0	0.1 (0.1)	0.1 (0.1)	0	0	
Silvereye	***7 (1.1)***	***5.5 (0.9)***	***6.5 (1)***	***3 (0.6)***	***2.3 (0.4)***	↓
Skylark	0.1 (0.1)	0.1 (0.1)	0 (0)	0	0	
Song thrush	**1.3 (0.3)**	**2.4 (0.4)**	**1.7 (0.3)**	**0.9 (0.2)**	**1 (0.3)**	↓
Swamp harrier	*0*	*0*	*0.1 (0)*	*0*	*0*	
Tūī	***0***	***0 (0)***	***0.1 (0.1)***	***0.1 (0)***	***0.3 (0.1)***	↑
Welcome swallow	*0.3 (0.1)*	*0.2 (0.1)*	*0.4 (0.1)*	*0.1 (0.1)*	*0.1 (0)*	
Yellowhammer	0	0.1 (0)	0.1 (0.1)	0	0	

There were significant trends of declining density of 8 species ($p < 0.05$) between 2004 and 2012. However, 3 of these species (Eastern rosella, greenfinch, and grey warbler) increased significantly in 5-minute counts during the same period.

There was an apparent near-significant ($p = 0.1$) decrease in blackbirds between 2004 and 2012.

The only species which increased in slow-walk transects between 2004 and 2012 was tūī ($p = 0.04$).

6 Discussion and conclusions

In the latest survey 47% more species and twice as many native species were found in green areas than residential. This pattern is apparently consistent over time and highlights the importance of green areas with a range of woody plants for native birds in Hamilton.

There were changes in the abundance of several bird species between 2004 and 2012 in one or more seasons (in winter or in spring), environments (in residential or in green areas), or survey methods (in 5-minute counts or in slow-walk transects). Although differences in the trends in counts of some species in winter and spring may be due to real changes in seasonal abundance, the cause or biological significance of disparate seasonal changes requires detailed information on the phenology of these species in Hamilton that is generally poorly known. For example, seasonal movement of New Zealand urban birds move in relation to local food supplies is poorly understood. Some habitats we sample have changed significantly since 2004, and this will affect the bird communities present there. For example, Waiwhakareke is the focus of a major restoration project involving considerable change to the vegetation of the sampled areas, particularly the removal of mature grey willow (*Salix cinerea*) trees and the replacement of pasture with native trees and shrubs (Appendix 2). Furthermore, although mortality at nests has been studied (Morgan et al. 2009; van Heezik et al. 2008), little is known about the rates and causes of mortality of subadult and adult birds. We limit further discussion here to species that changed consistently across different survey methods and seasons.

Tūī distribution and abundance increased consistently across all seasons and areas and with both survey techniques. Mean abundance in 5-minute counts in green areas increased from 0.03 to 0.49 from 2004 to 2012 in August, and from 0 to 0.48 in November. Mean abundance in 5-minute counts in residential areas in November increased from 0 to 0.09 from 2004 to 2012. Mean abundance in slow-walk transects in green areas in November increased from 0 to 0.3 birds per hectare from 2004 to 2012. These increases verify the widespread anecdotal perception that tūī are now more common in Hamilton than before 2008. Furthermore, the clear increase in tūī distribution and abundance that we measured in November shows that more tūī are now breeding in Hamilton. During the non-breeding season tūī forage over many kilometres and may congregate at sources of abundant nectar, but in spring they disperse and pairs defend smaller territories around nest sites with a foraging range of approximately 0.5 km (Bergquist 1985). The greater distribution of a smaller number of tūī in November counts compared with August reflects seasonal differences in behaviour, and has meant that tūī are now seen in a wider range of urban environments, including residential gardens, than in previous years. The magnitude of increase in tūī is consistent with what is known about their movements and nesting success in and around Hamilton (Innes et al. 2005b), the

targeted pest control carried out under Waikato Regional Council's Hamilton Halo project, and the general increase in this species in the North Island (Spurr 2012) which is probably due to the widespread application of possum and rat control.

Despite the recent increases, the abundance of tūī in Hamilton is still lower than in some other North Island cities, e.g. New Plymouth (mean of 1.56 tūī per green area 5-minute count in December 2011; Landcare Research unpublished data), suggesting there is potential for tūī abundance to continue to increase. New Plymouth has a greater proportion of native vegetation cover inside the city boundary than Hamilton (Clarkson et al. 2007), and there is more intensive pest control undertaken in new Plymouth. We suggest that habitat for tūī is not limiting in either city and that they higher numbers in New Plymouth are due to pest control, although it is difficult to explore habitat and food supply as limiting factors (Innes et al. 2010). Tūī readily utilise habitat with exotic vegetation (Robertson 2013) and do not require native forest to live in.

Some tūī (especially females) exhibit strong natal philopatry, meaning they typically breed close to their birthplace (Stewart & Craig 1985; Bergquist 1985), so continuation of Hamilton Halo pest control will primarily increase the number of winter visiting tūī, and control of mammalian nest predators in Hamilton will accelerate the recovery of tūī resident in the city throughout the year. The key predators of tūī in native forest (ship rats and possums) are less abundant and more unevenly distributed in Hamilton (Morgan et al. 2009), but expanding control of these pests in Hamilton to protect resident breeding birds is now prudent.

Kākā, a large iconic native parrot, was recorded for the first time in counts in August 2012: three birds at one count station at Taitua Arboretum. Juvenile kākā are known to disperse widely in winter (Higgins 1999) and kākā are sparse but regular visitors to some rural and urban parts of lowland Waikato including Hamilton (Moorhouse 2013), but the source or age of these birds is not known.

Blackbirds decreased across all 5-minute counts but their decline in slow-walk transects was not as statistically significant. It seems likely that there has been a real decline in blackbirds in Hamilton. We cannot explain this decrease but blackbirds are known to be preyed on by introduced mammalian predators, including possums, mustelids, rats, and cats (Higgins et al. 2006, Morgan et al. 2011).

Native birds play important ecological roles in pollination and seed dispersal so are valuable indicators of ecosystem health and sustainability, and they are generally appreciated by the public. Tūī are important pollinators of kowhai (*Sophora microphylla*) and flax (*Phormium tenax*), two native species that grow throughout the Waikato and are often planted in residential gardens and amenity parks in Hamilton. The presence of tūī in Hamilton during the summer flowering period of flax means an important plant/pollinator relationship has now been restored there.

Apart from tūī, New Zealand's only honeyeater (Meliphagidae) is the bellbird. Like tūī, bellbirds play important roles in native ecosystems, including pollination and seed dispersal of native plants, and face similar threats such as nest predation. Bellbirds were known to occur in Hamilton until about 1965–70 (C. Tanner, pers. comm.) and have been seen occasionally on the outskirts of the city in recent years (pers. obs.). In May 2010 bellbirds were translocated from Tiritiri Matangi Island and Tawharanui Regional Park to Hamilton in an effort to help their reestablishment in the city. No bellbirds were detected during the

course of this work, so it appears the translocation failed. Bellbirds are among the most difficult New Zealand birds to translocate (Miskelly & Powlesland 2013) so their failure to re-establish in this way is not unusual, and it remains likely that bellbirds and other endemic species such as New Zealand pigeon will return to Hamilton as their numbers increase in Hamilton Halo areas.

7 Recommendations

- These biennial counts should be continued (next in 2014) to monitor changes in bird abundance in various parts of Hamilton, in particular to monitor the response of tūī, bellbirds and New Zealand pigeon to ongoing pest control within and surrounding the city.
- The current effective control of possums and ship rats by Waikato Regional Council in forest areas surrounding Hamilton should continue, to increase abundance of tūī bellbird and New zealand pigeon in the city.
- Effective control of possums and ship rats to protect nesting native birds should be continued and expanded by Hamilton City Council in urban parks such as Donny Park, Hamilton Gardens, Hamilton East Cemetery, Jubilee Park, Hammond Park and other riverside reserves.
- Plant species beneficial to native birds should continue to be planted in urban parks by Hamilton City Council.
- Information on best-practice pest control and advice on planting to encourage native birds should continue to be made available to private landowners wishing to improve their property for birds.

8 Acknowledgements

Scott Bartlam helped greatly with bird count fieldwork. Guy Forrester and Norman Mason gave invaluable assistance with statistical analysis. An earlier draft was improved thanks to comments from Bill Lee and Mark Smale, and editing by Anne Austin.

9 References

Bergquist CAL 1985. Movements of groups of tūī (*Prosthemadera novaeseelandiae*) in winter and settlement of juvenile tūī in summer. New Zealand Journal of Zoology 12: 569–571.

Clarkson BD, Wehi PM, Brabyn LK 2007. A spatial analysis of indigenous cover patterns and implications for ecological restoration in urban centres, New Zealand. Urban Ecology 10: 441–457.

Fitzgerald N, Innes J 2010. Hamilton City biennial bird counts: 2004–2008. Landcare Research Contract Report 0910/099.

Fitzgerald N, Innes J 2012. Hamilton City biennial bird counts: 2004–2010. Landcare Research Contract Report LC892.

Gill BJ, Bell BD, Chambers GK, Medway DG, Palma RL, Scofield RP, Tennyson AJD, Worthy TH 2010. Checklist of the Birds of New Zealand, Norfolk and Macquarie Island, and the Ross Dependency, Antarctica. 4th edn. Wellington, NZ, Ornithological Society of New Zealand Inc. & Te Papa Press.

Hartley L 2012. Five-minute bird counts in New Zealand. New Zealand Journal of Ecology 36: 268–278.

Higgins PJ ed. 1999. Handbook of Australian, New Zealand and Antarctic birds. Volume 4: parrots to dollarbird. Melbourne, Oxford University Press.

Higgins PJ, Peter JM, Cowling SJ eds. 2006. Handbook of Australian, New Zealand and Antarctic birds. Volume 7: boatbill to starlings. Melbourne, Oxford University Press.

Innes J, Fitzgerald N, Thornburrow D, Burns B 2005a. Initial bird counts in Hamilton City, 2004. Landcare Research Contract Report 0405/130.

Innes J, Fitzgerald N, Watts C, Thornburrow D, Blackwell H, Landcaster E, Burns B 2005b. Distribution, movements and nesting success of Waikato tūī. Notornis 52(3): 173–178.

Innes J, Fitzgerald N, Thornburrow D, Burns B 2008. Second biennial bird counts in Hamilton City, 2006. Landcare Research Contract Report 0708/163.

Innes J, Kelly D, Overton JM, Gillies 2010. Predation and other factors currently limiting New Zealand forest birds. New Zealand Journal of Ecology 34: 86–114.

Miskelly CM, Powlesland RG 2013. Conservation translocations of New Zealand birds, 1863–2012. Notornis 60: 3–28.

Moorhouse RJ 2013. Kākā. In: Miskelly CM ed. New Zealand Birds Online. www.nzbirdsonline.org.nz (accessed 4 June 2013).

Morgan DKJ, Waas JR, Innes J 2009. An inventory of mammalian pests in a New Zealand city. New Zealand Journal of Zoology 36: 23–33.

Morgan DKJ, Waas JR, Innes J, Fitzgerald N 2011. Identification of nest predators using continuous time-lapse recording in a New Zealand city. New Zealand Journal of Zoology 38: 343–347.

R Core Team 2013. R: A language and environment for statistical computing. Vienna, Austria, R Foundation for Statistical Computing. http://www.R-project.org/

Robertson CJR, Hyvönen P, Fraser MJ, Pickard CR 2007. Atlas of bird distribution in New Zealand 1999–2004. The Ornithological Society of New Zealand, Inc, Wellington.

Robertson HA 2013. Tūī. In: Miskelly CM ed. New Zealand Birds Online. www.nzbirdsonline.org.nz (accessed 10 June 2013).

Stewart AM, Craig JL 1985. Movements, status, access to nectar, and spatial organisation of the tūī. New Zealand Journal of Zoology 12: 649–666.

Van Heezik Y, Ludwig K, Whitwell S, McLean IG 2008. Nest survival of birds in an urban environment in New Zealand. New Zealand Journal of Ecology 32: 155–165.

Appendix 1 – Scientific names and classification of birds referred to in text

Table 6 Names, taxonomic order and origin of birds recorded in Hamilton. Common names adapted from and scientific names follow Gill et al. (2010)

Common name	Scientific name	Order	Status
Australian magpie	*Gymnorhina tibicen*	Passerine (perching) birds	Introduced
Barbary dove	*Streptopelia risoria*	Pigeons and doves	Introduced
Bellbird	*Anthornis melanura*	Passerine (perching) birds	Native
Blackbird	*Turdus merula*	Passerine (perching) birds	Introduced
California quail	*Callipepla californica*	Game birds and allies	Introduced
Chaffinch	*Fringilla coelebs*	Passerine (perching) birds	Introduced
Common myna	*Acridotheres tristis*	Passerine (perching) birds	Introduced
Common pheasant	*Phasianus colchicus*	Game birds and allies	Introduced
Common redpoll	*Carduelis flammea*	Passerine (perching) birds	Introduced
Common starling	*Sturnus vulgaris*	Passerine (perching) birds	Introduced
Dunnock	*Prunella modularis*	Passerine (perching) birds	Introduced
Eastern rosella	*Platycercus eximius*	Cockatoos, parrots and parakeets	Introduced
Goldfinch	*Carduelis carduelis*	Passerine (perching) birds	Introduced
Greenfinch	*Carduelis chloris*	Passerine (perching) birds	Introduced
Grey warbler	*Gerygone igata*	Passerine (perching) birds	Native
House sparrow	*Passer domesticus*	Passerine (perching) birds	Introduced
Kākā	*Nestor meridionalis*	Cockatoos, parrots and parakeets	Native
New Zealand fantail	*Rhipidura fuliginosa*	Passerine (perching) birds	Native
New Zealand kingfisher	*Todiramphus sanctus*	Kingfishers and allies	Native
New Zealand pigeon	*Hemiphaga novaeseelandiae*	Pigeons and doves	Native
Pied stilt	*Himantopus himantopus*	Waders and gulls	Native
Pukeko	*Porphyrio melanotus*	Rails and allies	Native
Rock pigeon	*Columba livia*	Pigeons and doves	Introduced
Shining cuckoo	*Chrysococcyx lucidus*	Cuckoos	Native
Silvereye	*Zosterops lateralis*	Passerine (perching) birds	Native
Skylark	*Eurasian skylark*	Passerine (perching) birds	Introduced
Song thrush	*Turdus philomelos*	Passerine (perching) birds	Introduced
Spotted dove	*Streptopelia chinensis*	Pigeons and doves	Introduced
Spur-winged pover	*Vanellus miles*	Waders and gulls	Native
Swamp harrier	*Circus approximans*	Hawks and allies	Native
Tūī	*Prosthemadera novaeseelandiae*	Passerine (perching) birds	Native
Welcome swallow	*Hirundo neoxena*	Passerine (perching) birds	Native
White-faced heron	*Egretta novaehollandiae*	Herons, ibises and allies	Native
Yellowhammer	*Emberiza citrinella*	Passerine (perching) birds	Introduced

Appendix 2 – Comparative photographs of Waiwhakareke transects in 2006 and 2012

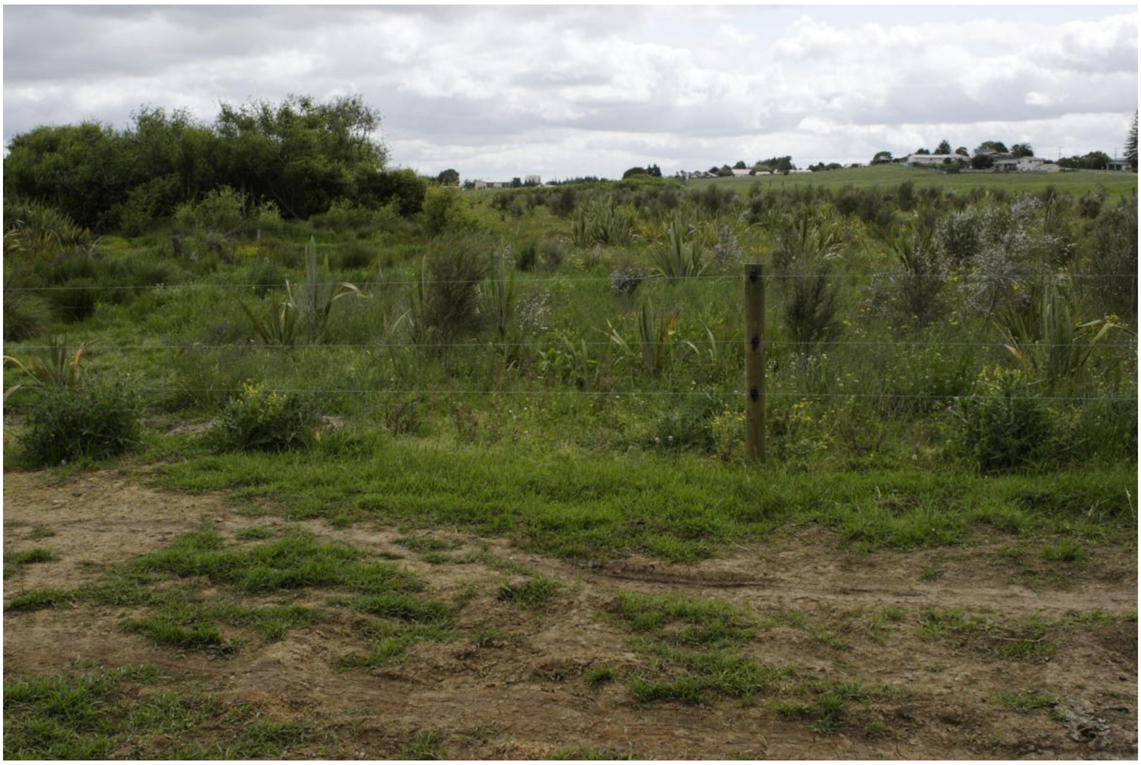

Figure 5 View along Transect 3 from start point in 1996.

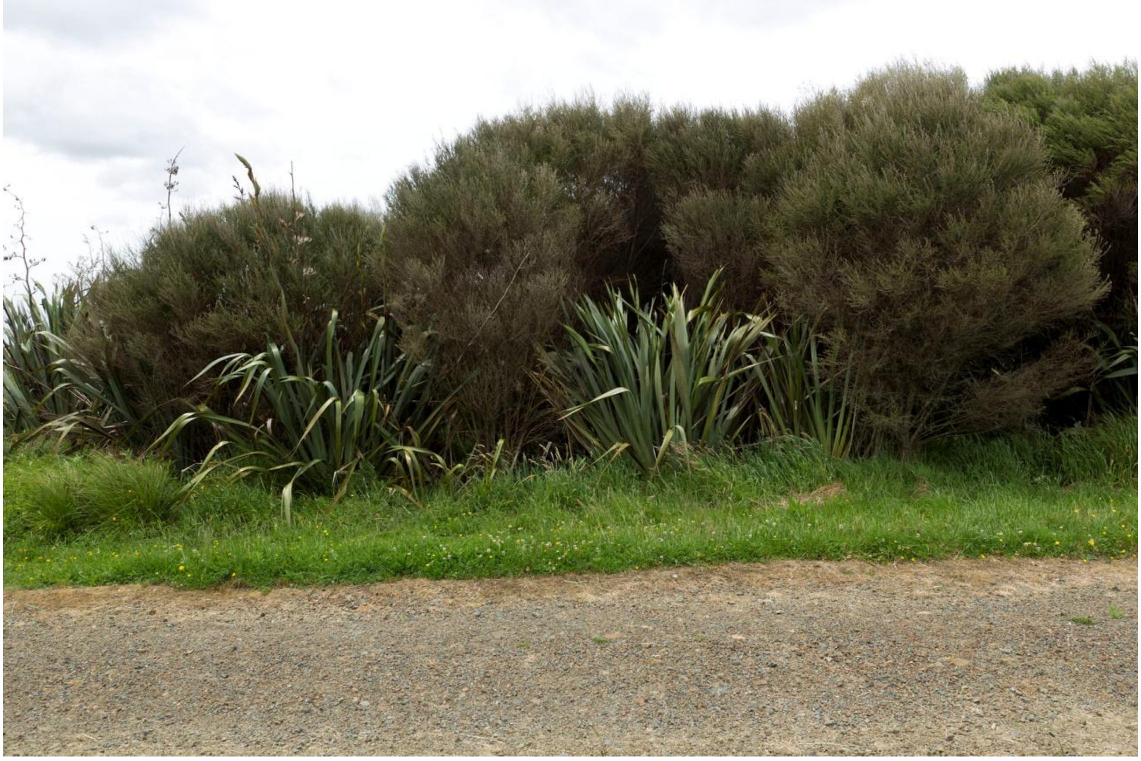

Figure 6 View along Transect 3 from start point in 2012.

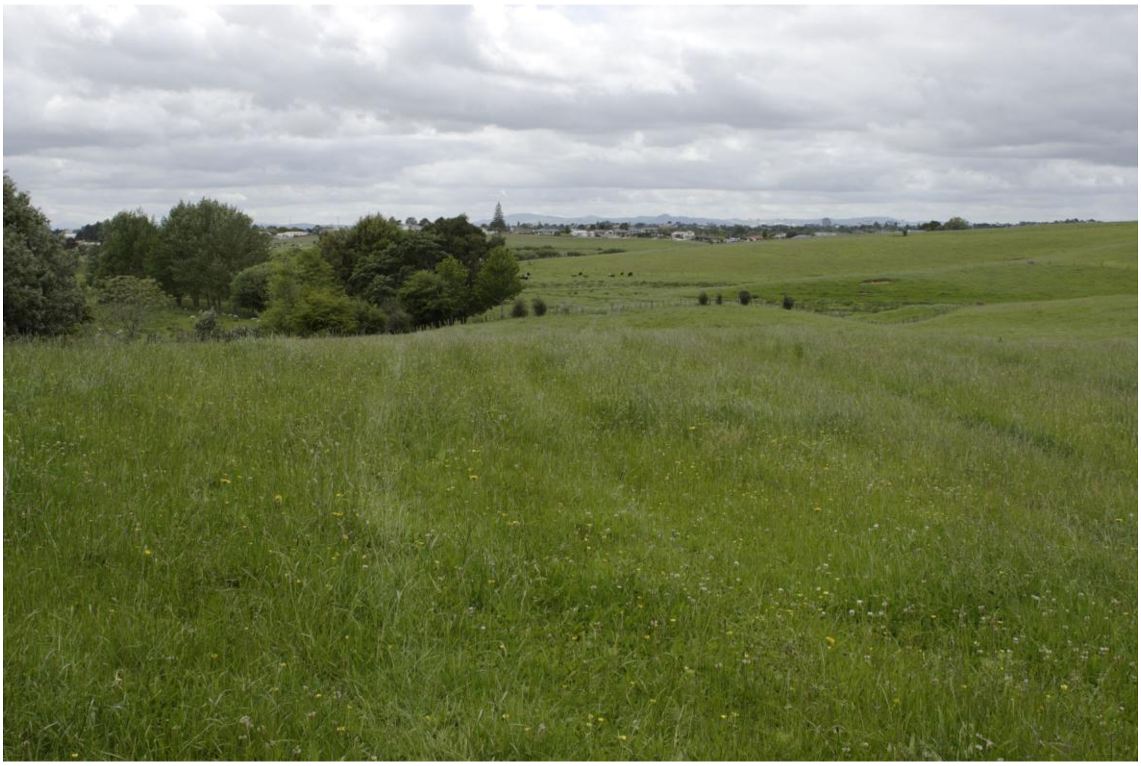

Figure 7 View along Transect 4 from end point in 2006.

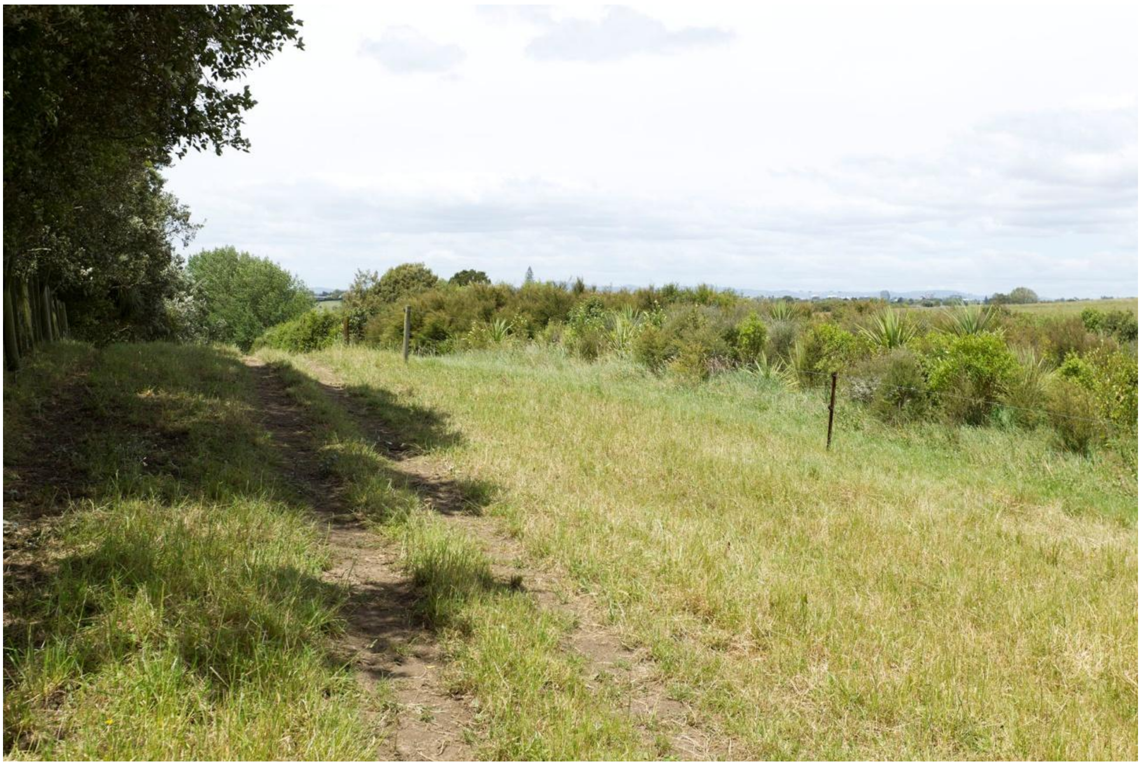

Figure 8 View along Transect 4 from end point in 2012.
