# Supplementary material for: Responses of New Zealand forest birds to management of introduced mammals
Source: Conserv Biol. 2020 Mar 23;35(1):35–49. doi: 10.1111/cobi.13456 (PMC7984369; doi:10.1111/cobi.13456)
Supplement: Supplementary file 10 — Supporting Material [file COBI-35-35-s009.pdf]

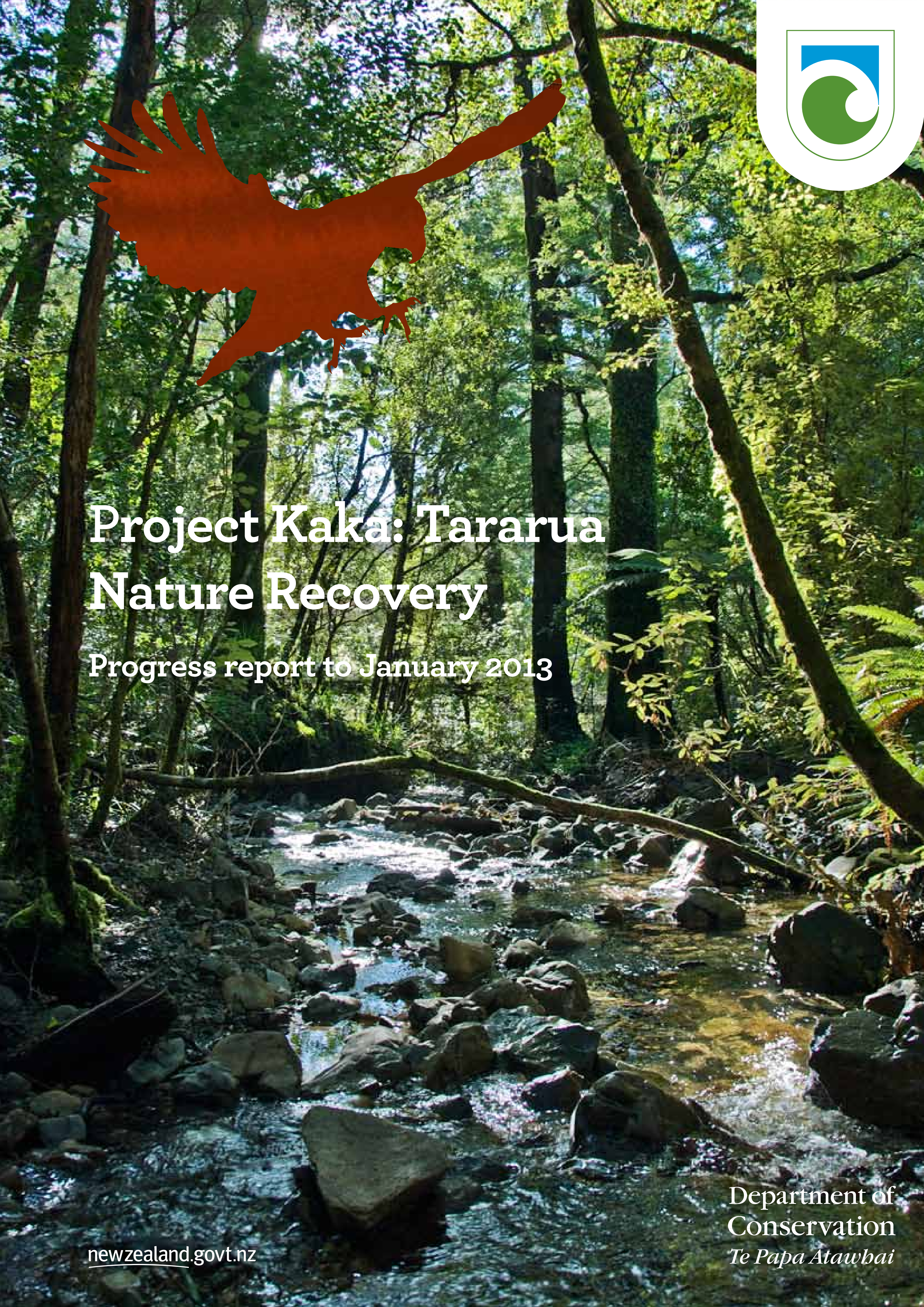

Project Kaka: Tararua Nature Recovery

Progress report to January 2013

Department of Conservation
Te Papa Atawbai

newzealand.govt.nz

Cover: Smith Creek, Tararua Ranges. *Photo: Jeremy Rolfe.*

© Copyright February 2014 New Zealand Department of Conservation

ISBN 978–0–478–15005–6 (printed copy)
ISBN 978–0–478–15006–3 (web PDF)

Published by The Department of Conservation, PO Box 10420, The Terrace, Wellington 6143, New Zealand.

CONTENTS

Project Kākā: Tararua Nature Recovery 1

Progress report to January 2013 1

Summary 1

1. Background 2

2. Monitoring results 2009 to January 2013 4

    2.1 Changes in possum indices 4

    2.2 Changes in rodent indices 5

    2.3 Changes in stoat indices 6

    2.4 Changes in deer and goat indices 7

    2.5 Changes in bird abundance 7

    2.6 Landcare Research update 10

3. Other research 12

4. Public engagement 12

5. Future developments 13

6. References 13

Project Kākā: Tararua Nature Recovery Progress report to January 2013

Summary

An earlier report (DOC 2012) described in detail the rationale behind Project Kākā: Tararua Nature Recovery, a 10-year forest restoration initiative in the Tararua Forest Park. It also covered the initial impacts of applying biodegradable 1080 baits on animal pests and some bird species over the 22 000-ha Project Kākā area. This report summarises the results of Project Kākā pest animal and bird monitoring programmes between 2009 and 2013. Monitoring of the forest response continues and will be reported in later years.

The possum Bite Mark Index has risen slowly in the Project Kākā area over the past 2 years after being reduced to near zero (from approximately 40% pre-control) following the initial pest control operation in 2010. This is likely to have reduced the browse and predation pressure exerted by possums. Rat tracking rates remained low for a full year after 1080 treatment before returning to pre-control levels by the end of the second summer after treatment (2012/13). The rapid rise in tracking rates during the 2012/13 summer was probably due to rat numbers increasing because of a moderate beech mast event in 2012. Mouse tracking rates were very low across all areas irrespective of treatment for 2 years after control, before also rising in summer 2012/13. Stoat tracking rates have shown little change and remained at low levels before, and after, control. There have been small changes in the deer and goat indices, but there is no evidence that deer and goats were affected by 1080 treatment.

There was a significant increase in the call counts of several bird species (bellbird + tūī, kākāriki, rifleman and whitehead) in the Project Kākā area 1 year after 1080 application. Tomtit counts had also increased by 2012. When comparisons were made between 2009 and 2012 (2 years after treatment), the number of species that still showed increases in call counts (now just bellbird + tūī, and tomtit) had fallen. This most likely reflected a recovery in rodent numbers and associated increases in predation two years after 1080 treatment. These results are consistent with the expectation outlined in the first Project Kākā report (DOC 2012); namely, that an effective knockdown of the three main predators (rats, stoats, possums) would benefit birds through at least one and possibly two breeding seasons of a 3-year treatment cycle.

The second of the 3-yearly aerial 1080 treatments occurred in spring 2013. The intensive monitoring programme will continue as planned.

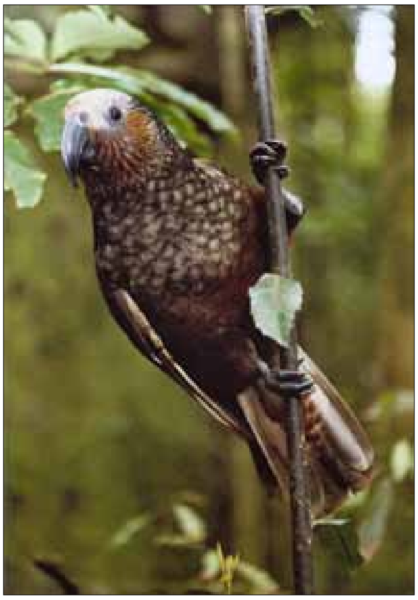

North Island kākā. *Photo: Ross Henderson*.

1. Background

In 2010, the Department of Conservation's (DOC's) Wellington Hawkes Bay Conservancy started an ambitious 10-year ecological restoration initiative in the Tararua Forest Park, calling it Project Kākā: Tararua Nature Recovery. As the name suggests, the goal is to restore forest and wildlife values in the Tararua Forest Park using kākā—New Zealand's endangered forest parrot and a rangatira of forest birds for Māori—as the flagship species. Kākā populations are declining on both the North and South Islands because of predation by stoats, rats and possums. Yet with protection from predators, wild kākā are thriving at several locations close to the Tararua Forest Park: on Kapiti Island, at Zealandia in Wellington city and at Pukaha, Mt Bruce, in the Wairarapa. Control of rodents, stoats and possums should also benefit other birds, lizards, invertebrates and forest plants that are heavily browsed by possums.

The management regime relies on recent advances in pest control; in particular, pre-feeding with non-toxic baits followed by application of biodegradable 1080 baits, both delivered aerially. This treatment can consistently reduce populations of the three major pest species (rats, stoats and possums) to very low numbers for at least a year (or longer in the case of possums). A single treatment of 1080 provides a 'triple hit' on possums, rats and stoats over large areas that cannot be achieved by any other single control method[1]. Hence repeated control on a 3-year cycle prior to the nesting season aims to give birds a safer 'breeding window' for at least one in every three years. The expectation is that this will be sufficient to enable bird populations to increase over the long term, other factors being equal. The first aerial pest control operation was carried out in November 2010 over the Project Kākā area—a 22 000-ha east to west 'slice' of the Tararua Forest Park (Fig. 1).

Biodiversity conservation is the first of three important objectives that collectively distinguish Project Kākā from other conservation projects. The second major objective is to improve our understanding of the value, safety and efficacy of three 3-year cycles of aerial 1080 for large-scale pest control through a comprehensive research and monitoring programme. The third objective is to increase the public's engagement with, and awareness of, conservation issues and the benefits and costs of different approaches to conservation management. As Project Kākā has evolved, a further important objective has been to build synergies with other management and research agencies. This has been successfully achieved by: (1) synchronising with TBfree (formerly Animal Health Board (AHB)) control operations along the eastern margins of the Tararua Range; (2) collaborating with the Greater Wellington Regional Council (GWRC) by using identical monitoring methods to track pest and bird responses; (3) linking the monitoring programme with larger research initiatives by DOC and Landcare Research scientists.

The Greater Wellington Regional council (GWRC) has been controlling possums to protect Wellington's water supply catchments and the ecological values of those areas. Possums in the Hutt Water Collection Area have been controlled using a 5–6-year cycle of aerial application of 1080 baits. The monitoring information obtained from Project Kākā will provide valuable information that can be used to compare the effectiveness of 3-year and 5–6 year aerial pest control operations. The commitment by DOC to a 10-year, three-control-cycle programme encouraged Landcare Research to include Project Kākā in its large Invasive Mammal Impacts research programme. The point of all the monitoring and research activities is to provide information that improves the effectiveness and efficiency of pest management practices for Project Kākā and elsewhere in New Zealand. More background information on the rationale for Project Kākā, details of the monitoring programme, the initial pest control operation and the first monitoring results are provided in DOC (2012), available online at

http://www.doc.govt.nz/publications/conservation/land-and-freshwater/land/project-kaka-tararua-nature-recovery-2010-2011-report/

---

1 Mice numbers can also be reduced at the same time, but with less consistency of results.

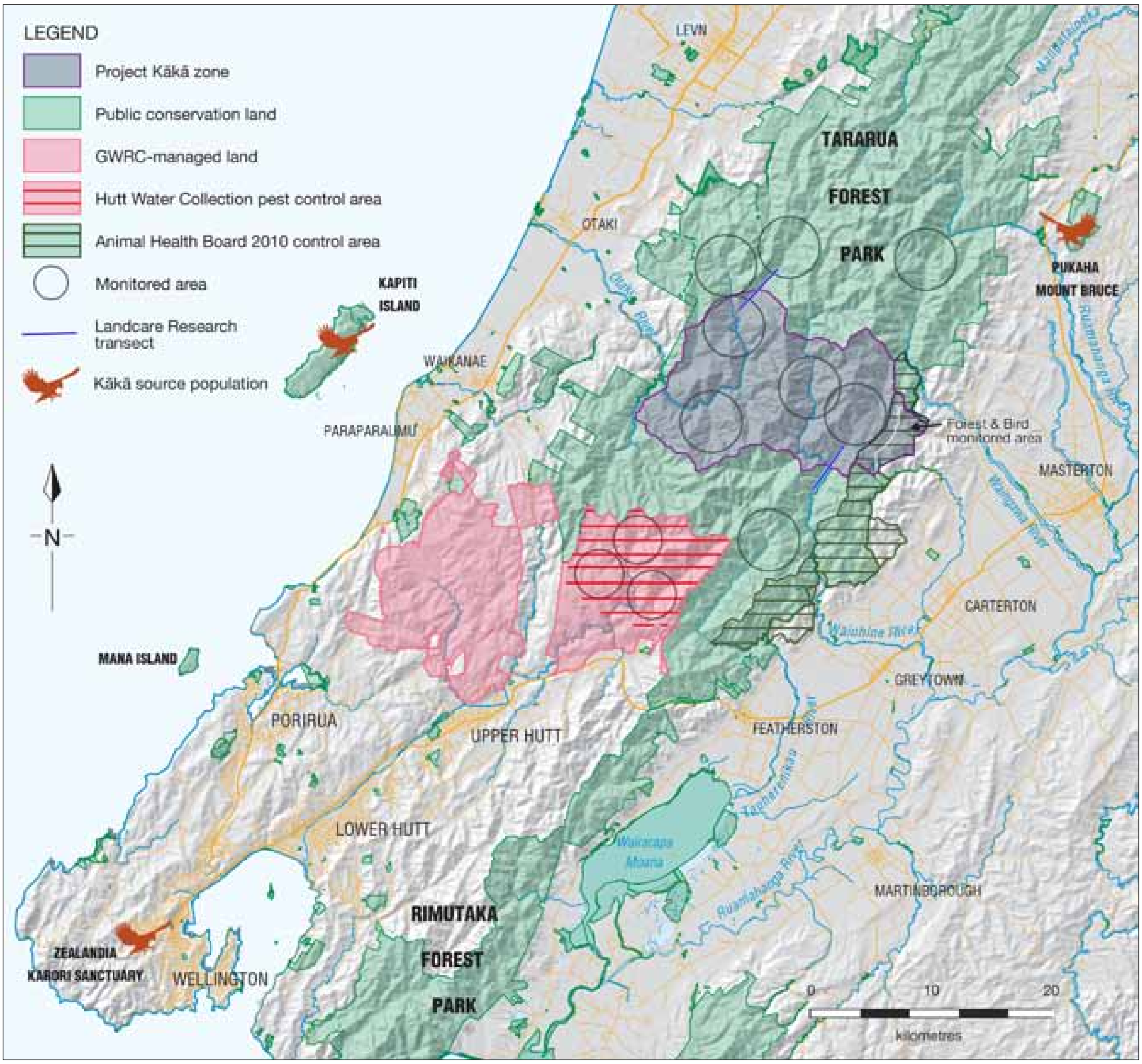

Figure 1. Map showing the location of the Project Kākā area within the Tararua Forest Park and other sites mentioned in the text.

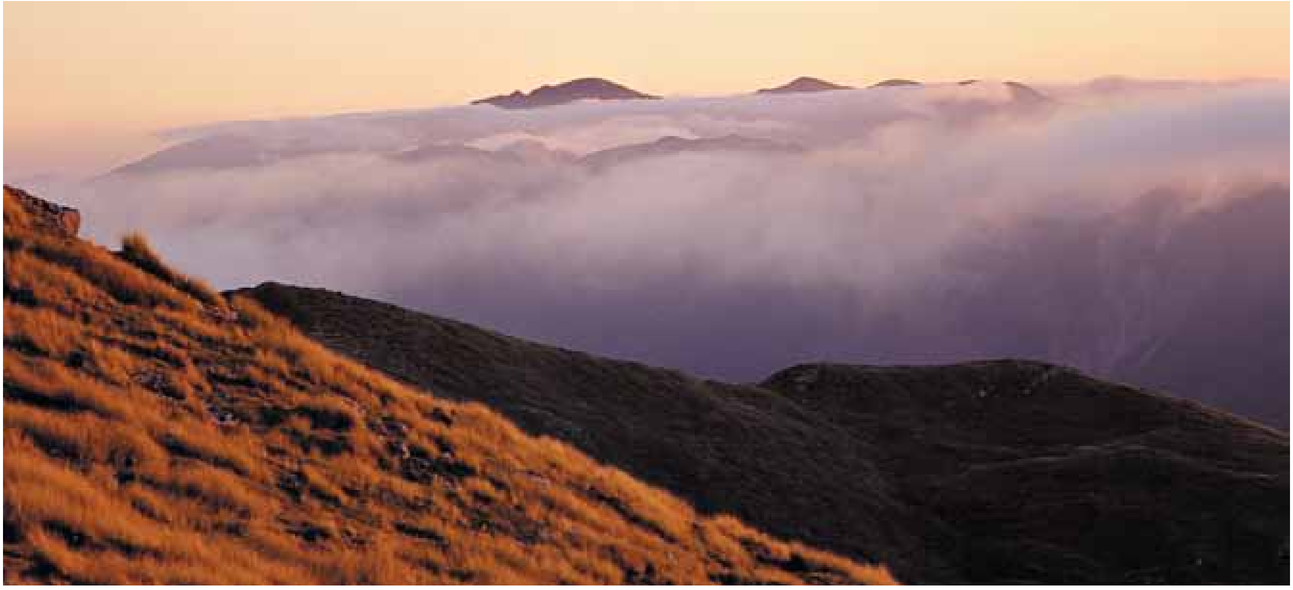

Angle Knob, Tararua Range, at sunset. *Photo: Jeremy Rolfe.*

2. Monitoring results 2009 to January 2013

This section covers:

- Changes in indices of pest animal abundance, showing the impacts of the 2010 aerial operation on possum, rat, mouse, stoat and deer/goat populations and their recovery through the summer of 2013
- Changes in indices of bird abundance from 2009 baseline through to the summer of 2012/13 (January 2013)
- Changes in indices of weta and beetle abundance

The methodology for the extensive plant and animal monitoring programme is detailed in the Annex of DOC (2012). Since the pre- and first post-treatment monitoring, further monitoring of pest, bird and invertebrate abundance indices was carried out in 2012 and 2013.

2.1 Changes in possum indices

In 2010, the operational control target for possums was to reduce the Bite Mark Index (bites made by possums on wax blocks) to less than 10%. In the Project Kākā treatment area the Bite Index fell from approximately 40% before control to near zero after, but there was no reduction in the non-treatment area (Fig. 2). Two years later, the Bite Index in the treatment area had risen slowly from near zero immediately post-control to close to 25%. This is less than half the index that was recorded in the non-treatment area (approximately 60%) at the same time (February 2013). Hence it is likely that predation and browse pressure from possums was considerably reduced in the Project Kākā treatment area for the two summers following control.

In the Hutt Water Collection Area ('Hutt Catchment' in graphs), possum densities remained lower for longer following aerial and ground control in 2009. Only in the last year (2012) was there a rise from near zero, some 4 years after control.

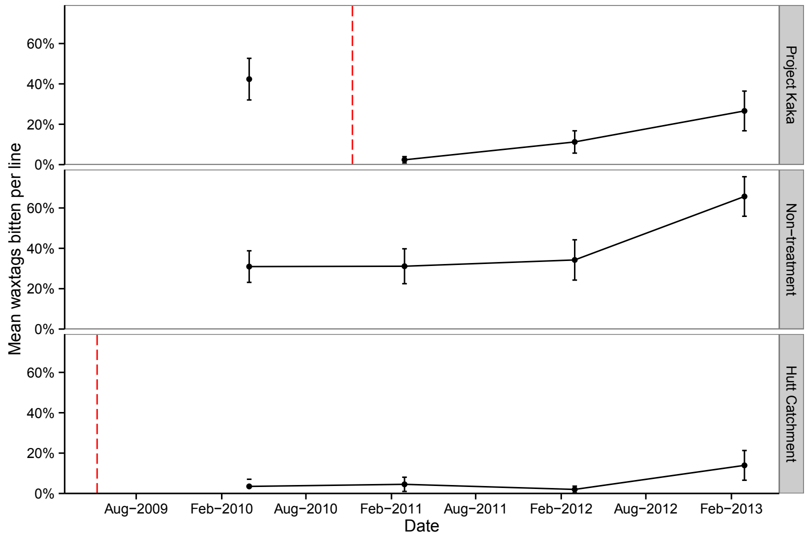

Figure 2. Average proportion of waxtags bitten in the Project Kākā treatment area and adjacent non-treatment areas before and after aerial 1080 application in November 2010, and in the Hutt Water Collection Area after aerial control in 2009. Red dashed lines indicate aerial 1080 operations. Error bars are 95% confidence limits around the mean.

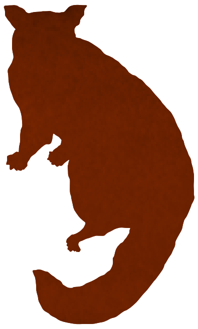

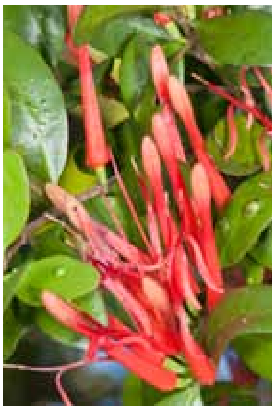

Mistletoe, *Peraxilla tetrapetala*. *Photo: Jeremy Rolfe*.

2.2 Changes in rodent indices

The control target for the rodent tracking index was to reduce the tracking rate (rat or mouse tracks recorded on paper in tracking tunnels) to less than 10%. The results for rats (Fig. 3) and mice (Fig. 4) showed that the tracking index for both species dropped to zero after the 2010 1080 treatment. The reduction was more marked for rats (which started at a higher tracking rate than mice). The tracking rate for rats stayed low for two summers (2010/11, 2011/12) before rising quickly in the spring and summer of 2012/13. A similarly rapid rise in rat tracking rates was recorded in the non-treatment area as well as in the Hutt Catchment area in the 2012/13 summer. Beech mast years (when large amounts of beech seed are produced) provide increased food for rodents in New Zealand beech forests. Mast years occurred in all three study areas in 2009 and 2012 and are represented by dashed black lines in Figs 3–5. Hence the rapid rise in rat numbers during the 2012/13 summer was most likely due to the 2012 mast year. The Project Kākā model aims to reduce rodent pressure, especially on breeding birds, for one (but preferably two) breeding seasons, so the rise in rodent numbers after two low years was to be expected. The lower rat tracking rates for two summers following the application of 1080 was in line with the objectives of the project.

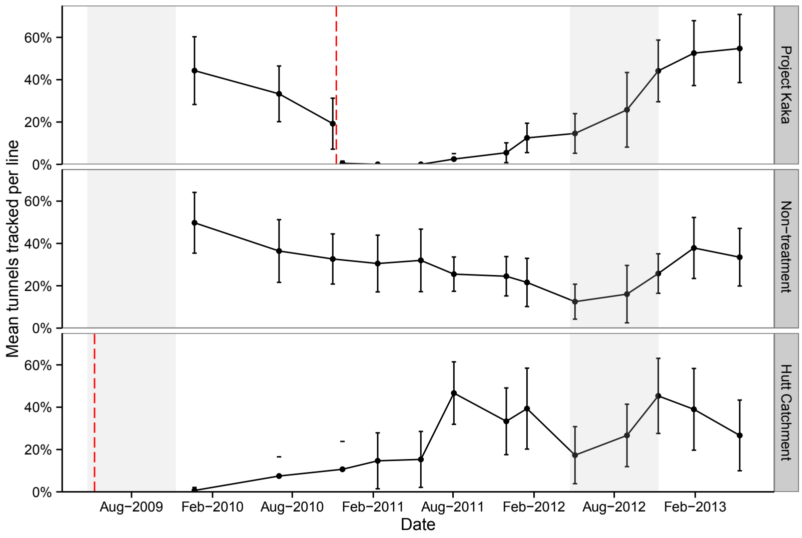

Figure 3. Rat tracking rates for the Project Kākā treatment and non-treatment areas before and after aerial 1080 application in November 2010, and for the Hutt Water Collection Area after aerial control in 2009. Grey shaded bands indicate beech mast periods. Red dashed lines indicate aerial 1080 operations. Error bars are 95% confidence limits around the mean.

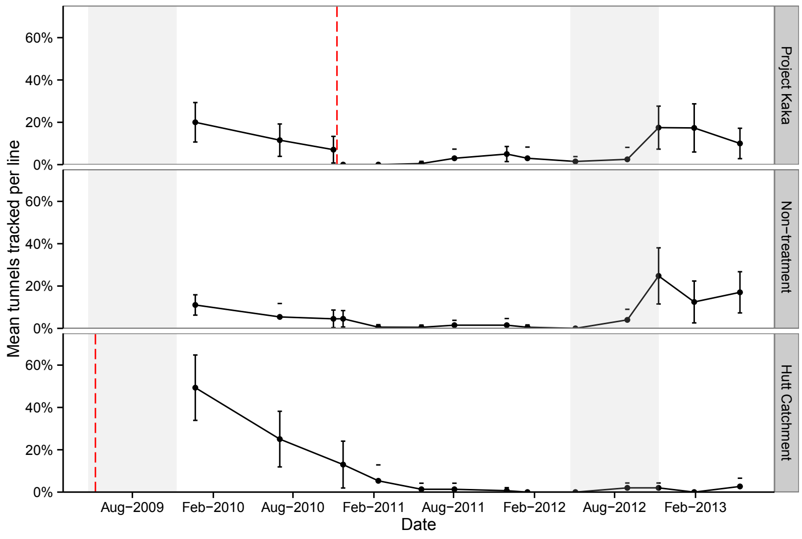

Figure 4. Mouse tracking rates for the Project Kākā treatment and non-treatment areas before and after aerial 1080 application in November 2010 and for the Hutt Water Collection Area after aerial control in 2009. Grey shaded bands indicate beech mast periods. Red dashed lines indicate aerial 1080 operations. Error bars are 95% confidence limits around the mean.

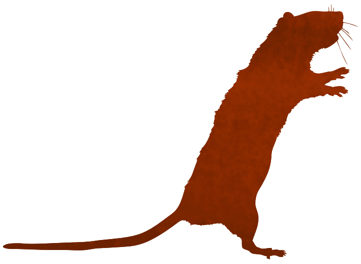

There are interesting differences in the rat and mouse response patterns between Project Kākā and the Hutt Catchment area. Rats will eat mice and can compete with them for food. Mouse tracking indices were high in the Hutt Catchment when rat indices were low (c.f. Figs 3 and 4) and were low in Project Kākā areas when rat indices were high (pre-2010). Whether or not this can be attributed to depredation or competition is an open question since some research suggests rats may inhibit the use of tunnels by mice. If this is the case, then mouse numbers might be higher than the index suggests. Based purely on the tracking index results it appears that, compared with rats, mice did not respond to the 2012 beech mast in the Hutt Catchment, while in the Project Kākā treatment and non-treatment areas, mice increased to pre-control levels in the 2011/12 summer. Perhaps the high stoat numbers in the Hutt Catchment were keeping mice numbers down. The further research planned should help with our understanding of the complex relationships between rats and mice in forests.

2.3 Changes in stoat indices

Given low stoat tracking rates in Project Kākā areas before control, no control targets were set. In the treatment area, tracking rates were zero after the 1080 operation and remained low until a small rise in 2013; more than 2 years after treatment. By this time the stoat indices were similar in both the treatment and non-treatment areas, but still significantly below the stoat indices for the Hutt Catchment at the same time (Fig. 5).

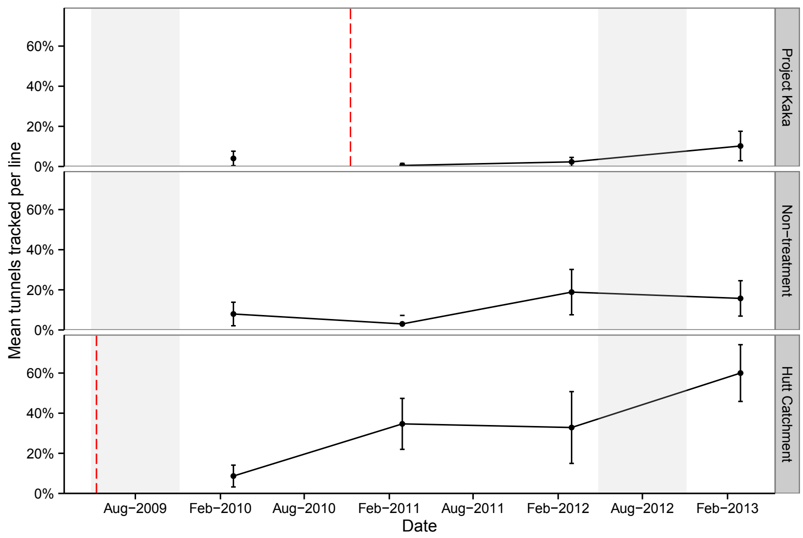

Figure 5. Stoat tracking rates for the Project Kākā treatment and non-treatment areas before and after aerial 1080 application in November 2010, and for the Hutt Water Collection Area after aerial control in 2009. Grey shaded bands indicate beech mast periods. Red dashed lines indicate aerial 1080 operations. Error bars are 95% confidence limits around the mean.

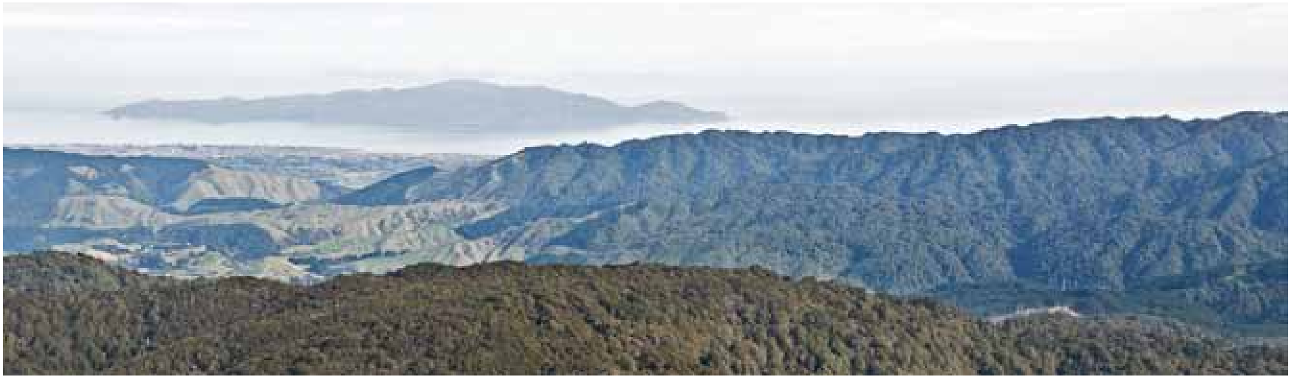

Kapiti Island. View looking west from Pukeatua Track, Tararua Range. *Photo: Jeremy Rolfe*.

2.4 Changes in deer and goat indices

Deer and goat faecal pellets were not differentiated for recording purposes; the analysis therefore assesses combined changes in deer and goat abundance. Pellet counts remained low in both treatment and non-treatment areas—as they had been before and immediately after the 1080 operation. (Fig. 6). There is no evidence that 1080 treatment has affected deer and goat numbers in the Project Kākā area, either immediately after the operation or over the following 2 years.

Figure 6. Deer and goat faecal pellet index for the Project Kākā treatment and non-treatment areas before and after aerial 1080 operation in November 2010. Red dashed line indicates the aerial 1080 operation. Error bars are 95% confidence limits around the mean.

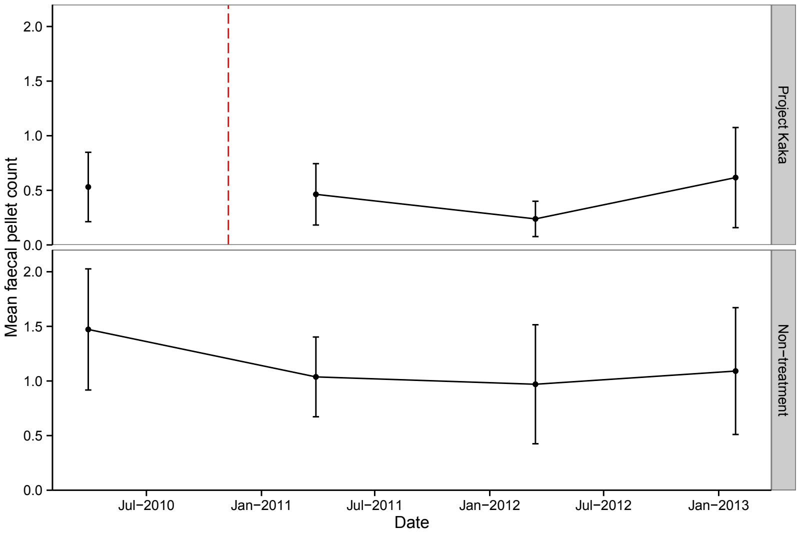

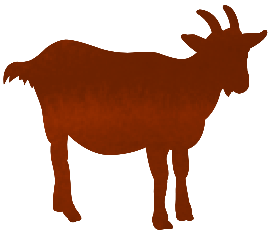

2.5 Changes in bird abundance

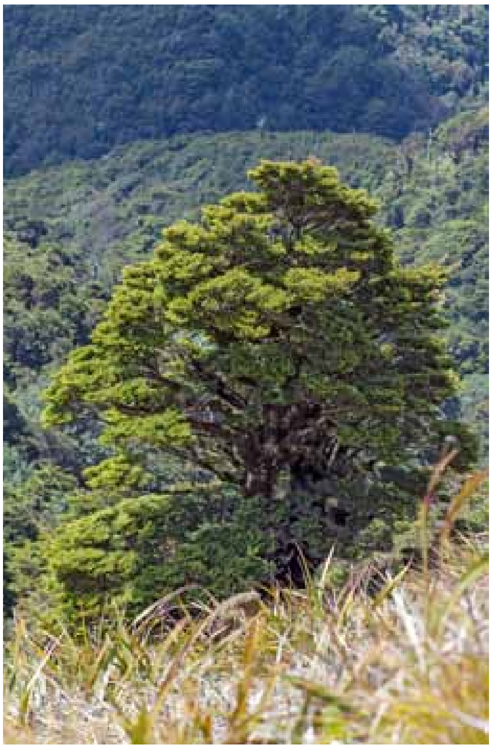

Silver beech, *Lophozonia menziesii*.
Photo: Jeremy Rolfe.

The addition of two further five minute bird counts[2] over November/December in 2011 and 2012 has provided a longer timeframe for assessing the effects—positive or negative—of the first 1080 treatment on different bird species in the project Kākā area. The first Project Kākā report (DOC 2010) indicated that no negative treatment effect was detected for any of the 16 monitored bird species, i.e. the aerial 1080 operation did not adversely affect bird counts. The difficulties of accurately measuring changes in bird abundance were also described. The bird count results for five species (kākā, kākāriki, rifleman, tomtit and bellbird) before and after treatment were included as representative of the 16 monitored species.

With two further monitoring results we can make some preliminary observations about the effect that lower numbers of predators may have had on bird survival and abundance over the 2 years following the application of 1080 in the Project Kākā area. These results can be compared with the model of how it is hoped 1080 treatment will help to increase bird numbers (refer to Fig. 11 in DOC 2012).

Prior to doing so it is important to remember that many factors influence bird survival from year to year. Food supply and weather are two important factors aside from predation. We know from information gathered in the Orongorongo Valley and the Tararua

[2] The analysis of the five minute bird count data used the mean of the mean counts for each count station (location). Data from all treatment and non-treatment sites was analysed the same way. This approach was used to remove an upward bias that can arise from using the maximum counts for each count station as had been done to calculate the results given in the first Project Kākā report (DOC 2012).

Ranges that there were beech mast events in 2009 and 2012. Also, there was severe weather in 2011 that may have affected bird survival. On 25 July 2011, New Zealand experienced its coldest winter snap in 15 years. Snow fell to low levels in both islands. Another major storm struck less than 3 weeks later (14 August); snow fell extensively down to sea level in Wellington for the first time since 1970. Heavy snow also fell on both sides of the Tararua Ranges and remained for days. If bird abundance had decreased markedly in 2011–2012, what role might these severe storms have played? If abundance increased, might it have been even greater under normal winter conditions?

These questions underscore the importance of having a non-treatment area for comparative purposes and the importance of continuing the in-depth monitoring and related research programme over three treatment cycles of aerial control using 1080. The latter will provide essential long-term trend data and test the value of the treatment beyond any specific impacts such as the winter storms of 2011.

The call count results for kākā, kākāriki, riflemen, tomtits, bellbirds + tūī and whiteheads are shown in Figs 7A–F. The tūī and bellbird data from the five minute bird counts were combined in one graph because of the difficulty of distinguishing the calls of these species. Kākā numbers remain low in all three monitored areas (Fig. 7A). Since kākā breeding success is closely related to food supply it will be instructive to look at the kākā response when more information on food supply is available. A confounding factor with kākā is that they are very strong fliers. If kākā are benefitting from lower predator numbers within the treatment area, their probable dispersal to other parts of the Tararua Forest Park may mask an increase in breeding success.

Kākāriki (Fig. 7B) and rifleman (Fig. 7C) counts increased significantly in the Project Kaka area 1 year after 1080 application when compared with the non-treatment area. But by 2012, counts in the treatment and non-treatment areas were similar.

The results for the other three species also showed relative increases in bird counts 1–2 years after 1080 application. Both tomtit (Fig. 7D) and bellbird + tui (Fig. 7E) counts increased over the 2 years following 1080 application and were significantly higher than in the non-treatment area. Whitehead (7F) counts were higher in the treatment area in 2011, but there was no difference between treatment and non-treatment area counts by 2012. Counts for these species also increased in the Hutt Catchment after 1080 application.

Lower numbers of possums, rats, mice and stoats in treatment areas after 1080 application has been followed by statistically significant increases in the counts for several bird species that are vulnerable to mammalian predation. This increase in bird counts is consistent with the prediction outlined in the first Project Kākā report (as described in DOC 2012); that is, an effective knockdown of rats, stoats, and possums would benefit birds for one and possibly two breeding seasons. Losses in the second and third seasons are expected as predator numbers increase, but the hope is that productivity exceeds mortality over the 3-year period.

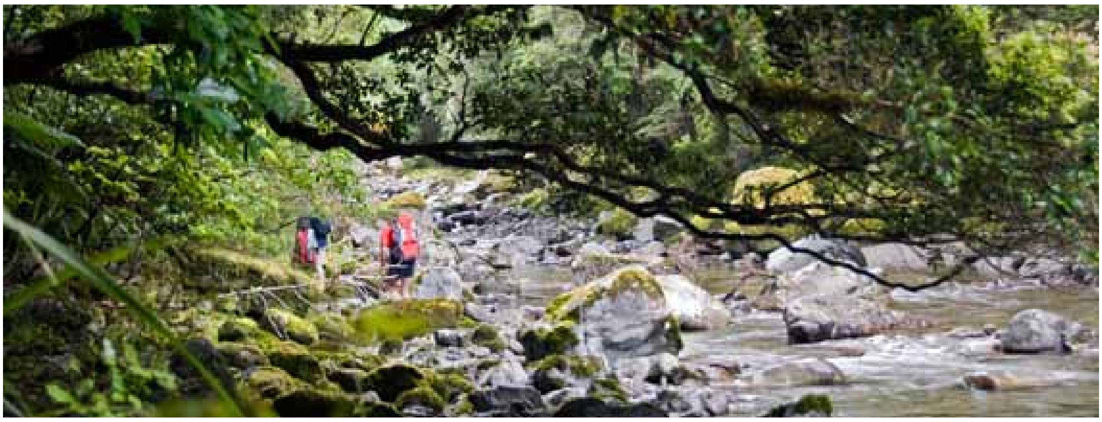

Trampers beside the Atiwhakatu Stream, near Donnelly Flat. *Photo: Jeremy Rolfe*.

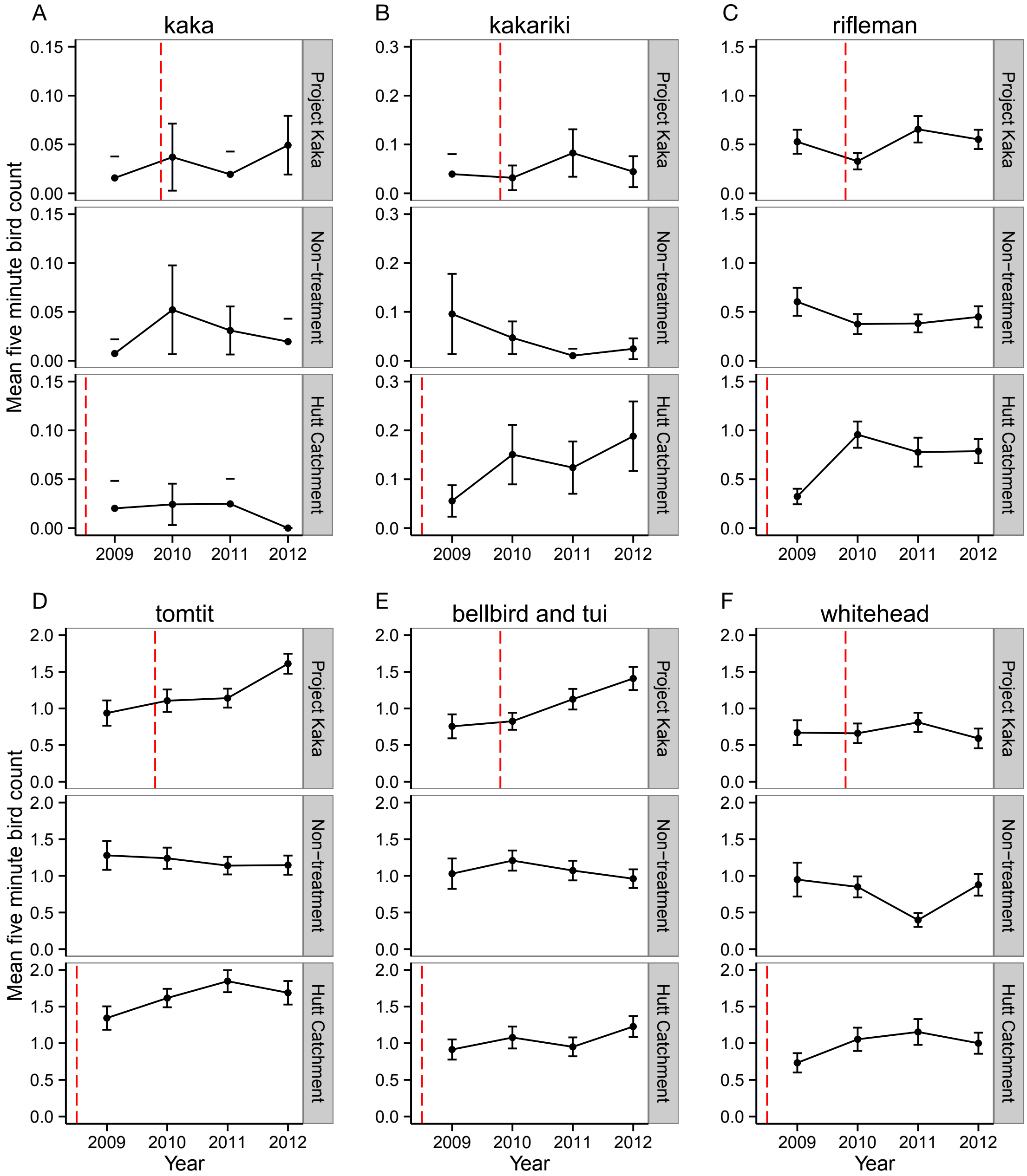

Figure 7. Five minute bird count results for Project Kākā treatment and non-treatment areas before and after aerial 1080 application in November 2010, and for the Hutt Water Collection Area after aerial control in 2009. Red dashed lines indicate aerial 1080 operations. Error bars are 95% confidence limits around the mean. A = kākā, B = kākāriki, C = rifleman, D = tomtit, E = bellbird + tui and F = whitehead.

In contrast with the species described above, changes in fantail and silvereye call counts were similar across treatment and non-treatment areas (Fig. 8A, B). Fantails and silvereyes are found throughout New Zealand and do not appear to be as vulnerable to predation pressure from rats, stoats or possums as many other native birds. It is therefore not surprising that these species did not respond positively to pest control. Concurrent changes in fantail and silvereye counts for treatment and non-treatment areas may instead relate to other factors, such as severe weather events or food supply. Fantails, for example, feed on insects on the wing and the effects of the harsh winter of 2011 may have contributed to reduced bird call counts in 2011.

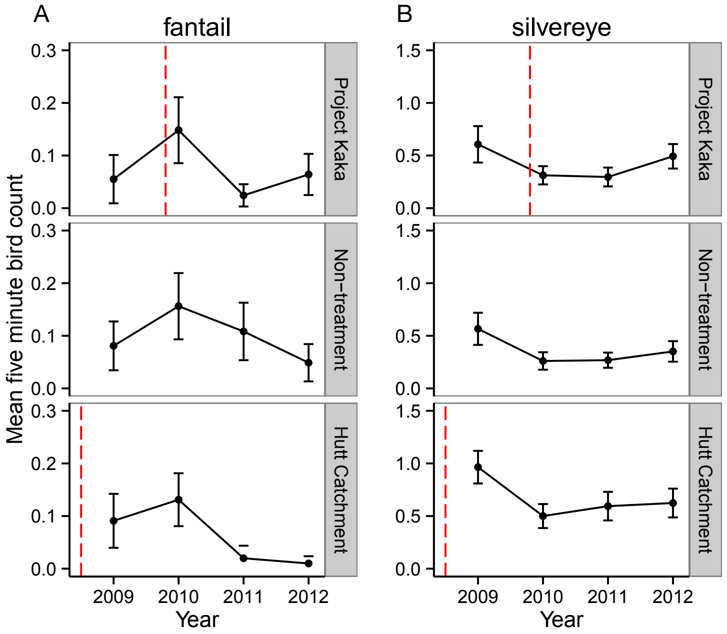

Figure 8. Five minute bird count results for Project Kākā treatment and non-treatment areas before and after aerial 1080 application in November 2010, and for the Hutt Water Collection Area after aerial control in 2009. Red dashed lines indicate aerial 1080 operations. Error bars are 95% confidence limits around the mean. A = fantail, B = silvereye.

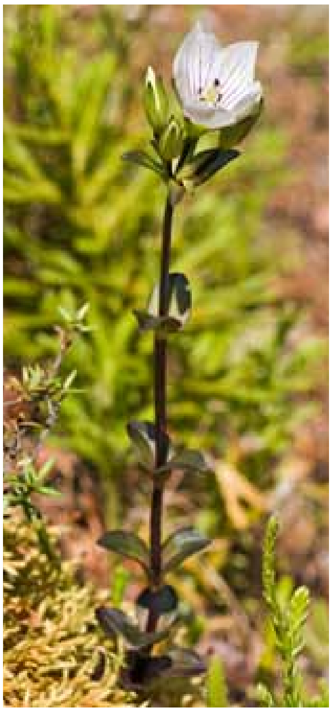

Gentianella montana.
Photo: Jeremy Rolfe.

Statistical tests comparing bird monitoring data from treatment and non-treatment areas over the period 2009–2012 are summarised in Table 1. Statistical tests were carried out to answer the following key questions:

1. Did bird counts drop in treatment areas immediately after 1080 application in 2010? This would show if 1080 caused significant direct bird mortality.
2. Did bird counts increase in treatment areas a year after 1080 application (2011) compared with 2009 counts?
3. Did bird counts increase in treatment areas 2 years after 1080 application (2012) compared with 2009 counts?

Results show statistically significant ($P < 0.05$) increases for four species a year after 1080 treatment. The expectation of some loss of gains by Year 2 was supported by the results.

Table 1. Statistically significant changes in bird call counts in Project Kākā treatment area compared with the non-treatment areas. 2009–2010 tests were for significant decreases; 2009–2011 and 2009–2012 periods indicate significant increases in Project Kākā counts compared with non-treatment areas.

2009–2010	2009–2011	2009–2012
No significant decreases	Bellbird + tui	Bellbird + tui
	Kākāriki	Tomtit
	Rifleman	
	Whitehead	

2.6 Landcare Research update

Landcare Research has continued to be involved with Project Kākā through supplementary monitoring of pest and native species on the boundaries of the Project Kākā pest control area and the surrounding non-treatment areas (see Fig. 1). Rat tracking rates at the LCR monitoring sites on the boundaries of the treatment area returned to high levels within 9 months of 1080 application compared with DOC's monitoring sites within the treatment area which took around 18 months to recover. This may indicate that re-invasion was occurring at the boundary. Alternatively (or additionally) it might also indicate that LCR's monitoring sites were more productive for rats than DOC's. Further, rat tracking rates at the LCR monitoring sites ended up higher inside the Project Kākā treatment area than outside it, perhaps due to reduced rat competition with possums in the treatment area (where possum bite mark indices remained lower relative to outside the treatment area). Changes in rat abundance were potentially reflected in the number of key invertebrate prey caught in pitfall traps. Beetles and (to a lesser extent) wētā showed higher abundance at the treatment sites over the 9 months following control but then became indistinguishable from non-treatment sites after this time—coincident with the recovery of rat numbers (Fig. 9). Landcare Research is also measuring relative abundance of tree wētā using 'wētā hotels'—wooden artificial refuges nailed to trees that wētā use for shelter. Changes in the mean number of wētā per hotel with rat abundance were somewhat confounded with increasing occupancy over time as the hotels became more weathered and accepted by the wētā. However, there did appear to be a lower number of wētā in the treated monitoring sites relative to the untreated sites post-August 2011 (Fig. 10), corresponding to the increase in rat tracking rates at this time.

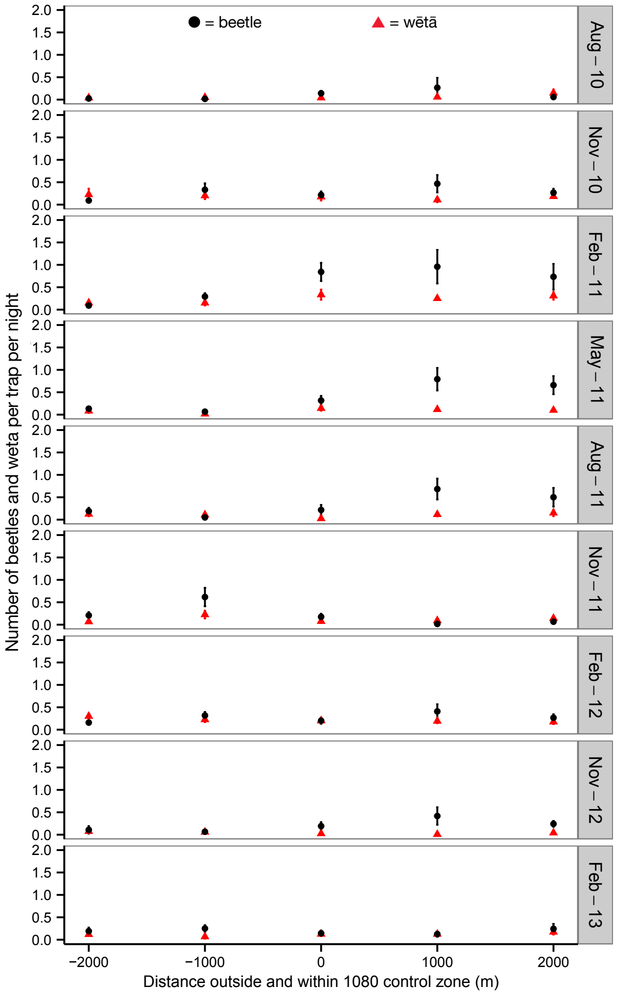

Figure 9. Results of pitfall trap monitoring of wētā and beetles outside and within the Project Kākā pest control area from August 2010 (1080 applied) to February 2013.

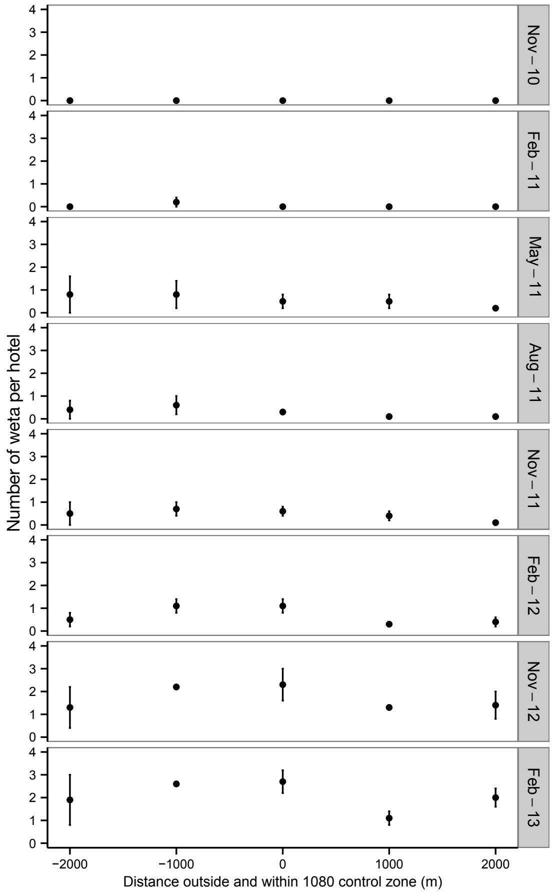

Figure 10. Results of wētā hotel monitoring outside and within the Project Kākā pest control area from August 2010 (1080 applied) to February 2013.

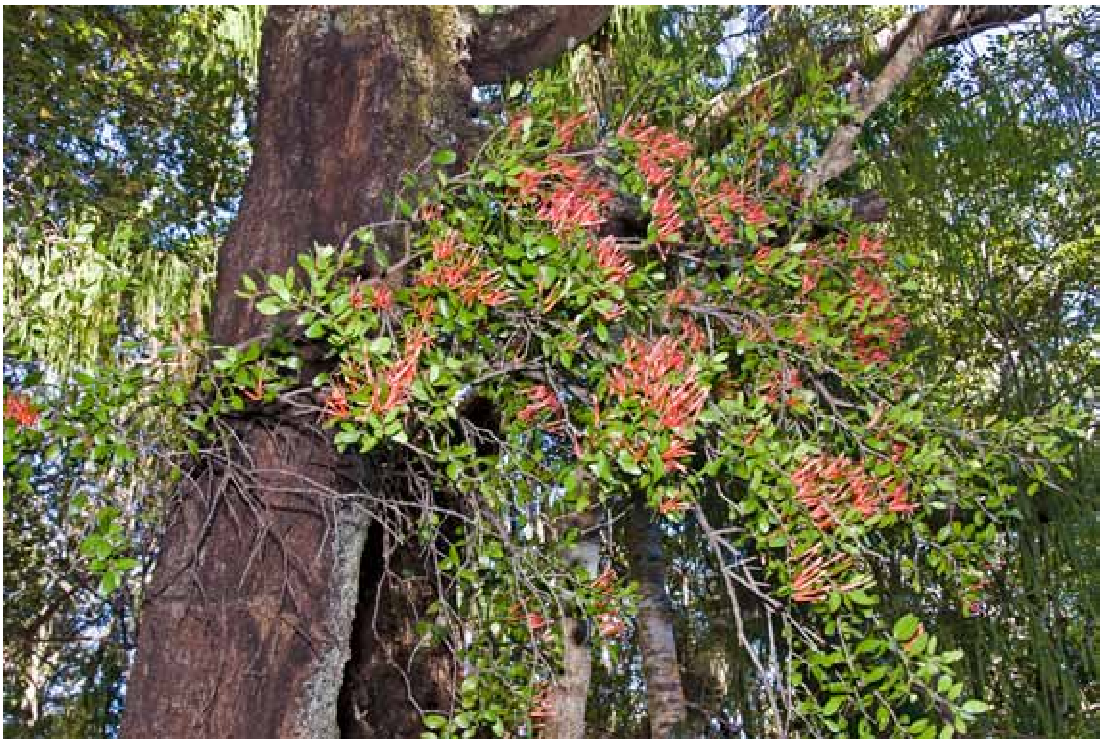

Mistletoe, *Peraxilla tetrapetala*, in Tararua Range. *Photo: Jeremy Rolfe*.

Landcare Research is committed to working with DOC on Project Kākā via on-going monitoring plus analysis and publication of combined monitoring data. By pooling knowledge and resources, progress can be made on both organisations' mutual goals of identifying better ways of managing multiple invasive species and maximising the benefits to native species.

3. Other research

Project Kākā is one of three sites where 1080 is being used and where the effects of this use on forest birds is being closely monitored. The other sites are in the Marlborough Sounds and South Westland. At each site, bird call counts are carried out regularly during summer. As well, a few key species at these locations are monitored in greater detail by measuring nesting success and survival. In Project Kākā, the rifleman is the species that is being intensively monitored to detect declines that might be caused by incidental by-kill immediately after applying 1080 as well as increases in abundance and productivity that might result from 1080 reducing stoat, rat and possum numbers. It is particularly important to replicate this work at as many sites as possible to provide confidence in any conclusions about the effects of 1080 on forest birds.

So far, seventy rifleman nests have been located (by following individual birds) in the Project Kākā area and surrounding non-treatment areas. Nests were visited regularly or had automatic cameras installed to take photographs of birds and predators going in and out of the nests. Nesting success has been significantly higher in the treatment area, which is consistent with the big drop in rat numbers recorded (Fig. 3) after 1080 treatment. At the time of writing, only rats have been recorded by the automatic cameras, although stoats are also known to be significant predators of riflemen elsewhere.

4. Public engagement

A group of local volunteers has set up a successful stoat, rat and possum control operation at Donnelly Flat, the eastern entrance to the Project Kākā area. Donnelly Flat is an area of fertile river flats dominated by podocarps, and provides high-value foods for many native birds. It is also the area of highest recreational use by visitors to the Tararua Forest Park and for this reason it was excluded from the Project Kākā aerial 1080 operation. The community pest control effort at Donnelly Flat therefore forms a vital part of the overall Project Kākā restoration effort.

The volunteer trapping project at Donnelly Flat is about to start its fourth year. Since 2010, the volunteers have trapped over 1700 pest animals. This figure includes approximately 200 possums, more than 650 rats, almost 800 mice and 50 mustelids. Removing this large number of pest animals from the popular Donnelly Flat area is benefitting the forest and the visitors that frequent it. Over the past 3 years, volunteers have taken on more responsibilities—organising trapping teams, working with school groups, and undertaking the trapping, maintenance work and monitoring of the pests. The Department of Conservation warmly acknowledges the ongoing commitment these volunteers have made to this successful project. Potential volunteers are invited to check out the 'Get involved' page on the DOC website (www.doc.govt.nz) or should phone DOC's Masterton Field Base (+64 6 377 0700).

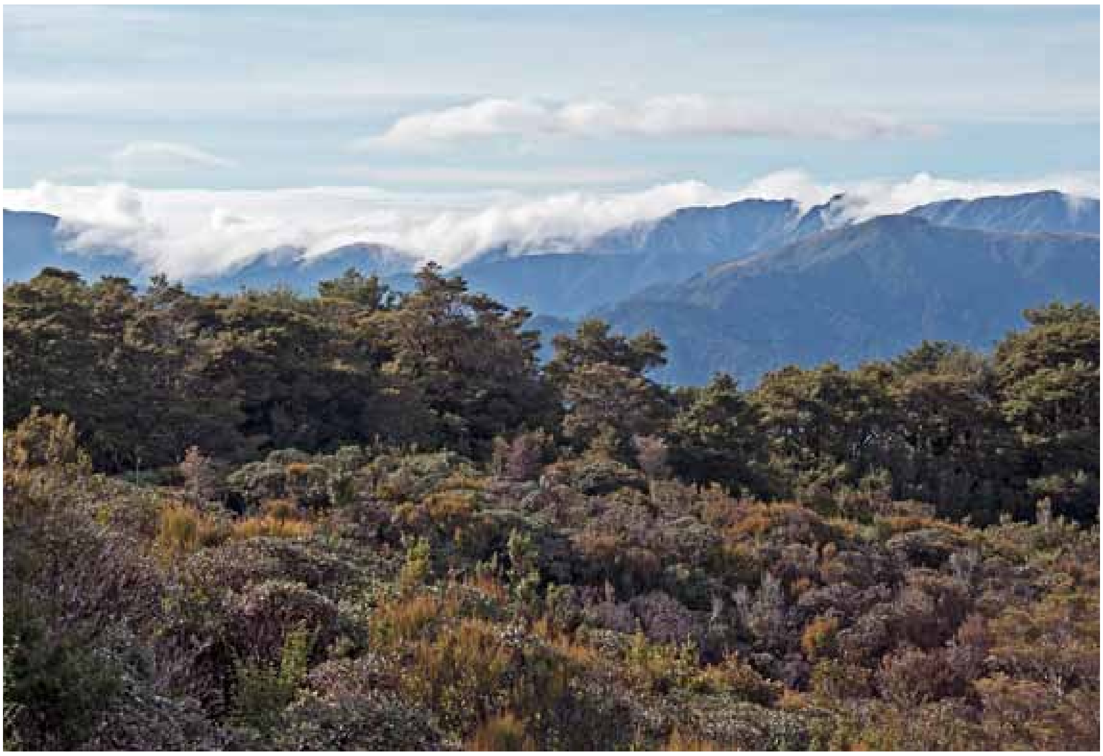

View from Pukeatua Track, Tararua Range. *Photo: Jeremy Rolfe*.

5. Future developments

The second of the 3-yearly aerial 1080 treatments over the 22 000 ha Project Kākā area was carried out in spring 2013. The Greater Wellington Regional Council has scheduled the next aerial control for the Hutt Catchment for late winter of 2014, a year after the DOC treatment.

Modelling by researchers predicts that there will be another beech mast in 2014 in the Tararua Ranges. If 2014 is a mast year, then the production of fruits and seeds in the late summer of 2014 would mean an abundant food supply on the ground during the 2014 winter. This would benefit rats and mice throughout the mast area, which is likely to include the Hutt Catchment and Project Kākā treatment and non-treatment areas. If the mast occurs, we have a unique research and management opportunity to compare the efficacy of rodent control in the two treatment areas in relation to the timing of beech mast events.

In addition to the 1080 treatment in 2013, the intensive monitoring and research programmes will continue. The first 3-year cycle has been completed and the intensive monitoring programme has shown positive results, with an upswing in the numbers of some important bird species following the very successful reduction in possum, rat, mice and stoat numbers in 2013. However, while the early results are promising, there is still much to find out about how to manage for conservation gains that can be investigated through Project Kaka.

6. References

DOC (Department of Conservation) 2012: Project Kākā: Tararua Nature Recovery. Project background and progress report covering July 2010 to June 2011.

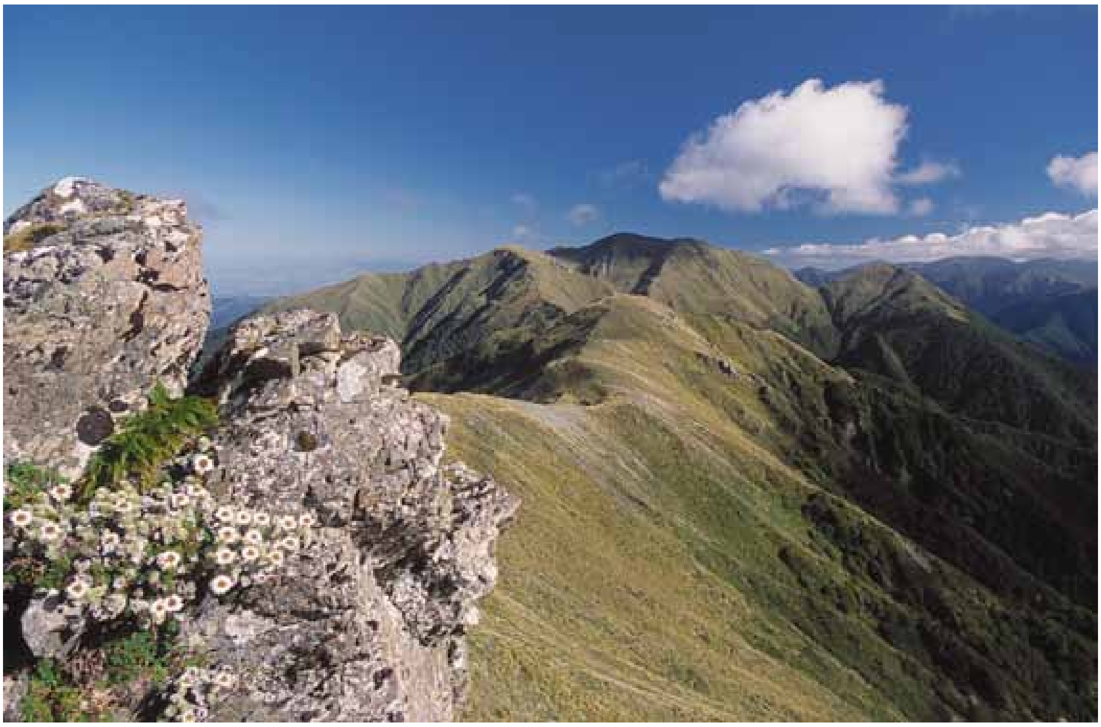

Isabelle Peak from Jumbo Ridge, Tararua Range. *Photo: Jeremy Rolfe*.
