# Supplementary material for: Responses of New Zealand forest birds to management of introduced mammals
Source: Conserv Biol. 2020 Mar 23;35(1):35–49. doi: 10.1111/cobi.13456 (PMC7984369; doi:10.1111/cobi.13456)
Supplement: Supplementary file 11 — Supporting Material [file COBI-35-35-s011.pdf]

Long term benefits of aerial 1080 operations on South Island tomtits (*Petroica macrocephala macrocephala*)

Billy Hamilton

Ecological Networks Ltd.,

10, Coughtrey Street,

Dunedin, New Zealand.

Email: econetworks@xtra.co.nz

Ecological Networks Report EN9-2

AHB Project Number R-80572-01

December 2009

1 Summary

1.1 Project and Client

This study (Project R-80572) was to ascertain the long term benefits of 1080 possum control operations on South Island tomtits (*Petroica macrocephala macrocephala*). The work was undertaken by Ecological Networks Limited and Dr. B. Hamilton during February 2005 and October 2009 and was funded by the Animal Health Board (AHB).

1.2 Objectives

To determine if there are any long term benefits of aerial 1080 possum control operations on tomtits by:

- Comparing seasonal counts of birds using transect line distance sampling.

1.3 Methods

- Tomtit density was compared at two sites within the Otago region
- Sites included non-treatment (Dunedin area where no 1080 had been used) and treatment (Hampden area where aerial 1080 operation had occurred in 2002 and 2008) sites.
- The line transect methodology of distance sampling was used for density estimation.
- 45 transects 250 metres long were set up at each site
- Sampling occurred summer and winter from summer 2005 to summer 2009.

1.4 Results

- Tomtit density decreased in treatment site following aerial 1080 operation, this decrease was not permanent.
- Tomtit density significantly increased at the treatment site during the breeding season immediately following aerial 1080 operation.
- Similar density increases were obtained following the 2002 aerial 1080 operation at the treatment site.
- Tomtit density is higher in treatment area than non-treatment area.
- Tomtits appear attracted to observers during line transect distance sampling methodology.

1.5 Discussion

- While there is an initial decrease in tomtit density following aerial 1080 operations this is not permanent and numbers increase significantly during the breeding season immediately following the operation.
- The greater density of tomtits within the treatment area may stem from a greater carrying capacity caused by the removal of mammalian pests through AHB control operations.
- No large tomtit kills were observed along and between transect lines in the sampling weeks following the 2008 aerial 1080 operation within the Hampden treatment site.

1.6 Recommendations

- Permanent line transects are set up in proposed aerial 1080 control zones to monitor susceptible bird species density trends over time.
- Comparisons to be made with similar areas where AHB control operations have ceased.
- Data from these studies could be used to measure other variables including sowing rates, types of baits, repellents, effect of season on non-target species susceptibility.

2 Introduction

Research project R 80572 monitored tomtit numbers in the Otago region from February 2005 to August 2008. Monitoring occurred during the summer and winter seasons of these years in both study areas. During the winter of 2008 an aerial 1080 possum control operation was undertaken within the treatment area of the research project areas.

This report is the analysis of the monitoring data during the study periods. All research was contracted to the Animal Health Board.

3 Background

Recently it has been calculated that there are approximately 30 million brushtail possums (*Trichosurus vulpecula*) within New Zealand. Allowing for previous estimation of their foraging consumption (Brockie 1992), this would equate with over 9,000 tonnes of forest being eaten each night. In addition, to their adverse effects on native flora values, possums within New Zealand also carry bovine tuberculosis (TB), a contagious disease caused by the bacterium *Mycobacterium bovis*. TB can be passed on from possums to other mammalian species where there is direct contact between species. In New Zealand this contagion risk poses a serious economic threat to our cattle and deer farming industries (Livingstone, 1994; Cowan, 2001, Coleman and Caley 2000).

The Animal Health Board (AHB) is responsible for controlling bovine TB within New Zealand. To achieve this, the AHB contracts outside agencies to control TB vectors, such as possums, ferrets and pigs.

These pest control contractors use a variety of control methods including dogs, traps and toxins. Of the latter there are six main toxins used in possums control, these being 1080 (sodium monofluoroacetate), phosphorus, cholecalciferol, pindone, cyanide and brodifacoum. Of these, ~~aerially delivered~~ 1080 is the only bait licensed for aerial delivery and is one of the most highly effective methods of possum control and can reduce possum

population by 90% or greater (Eason *et al*., 1994; Morgan *et al*., 1997; Veltman and Pinder, 2001).

Generally aerial 1080 is broadcast in carrot or cereal bait form and while it is highly effective in targeting possums there is an inherent risk to non-target native species such as birds. One of these bird species at risk from aerial 1080 control operations is the native tomtit (*Petroica macrocephala*), (Spurr 1991, Spurr and Powlesland 1997; Powlesland *et al.* 2000; Westbrooke *et al*. 2003; Westbrooke and Powlesland 2005).

Tomtits are a small passerine that forages on the ground, feeding mainly on invertebrates. Invertebrates can feed on baits containing 1080 and subsequently retain the residue within their body. Therefore, insectivores such as tomtits are susceptible to being poisoned through their food source. This South Island species can be found throughout the South Island (Bull *et al*., 1985) in a variety of forest habitat types, including mature native forests, beech (*Nothofagus* spp.), manuka (*Leptospermum scoparium*), kanuka (*Kunzea ericoides*), and exotic plantations (Heather and Robertson, 1996). They can also be found around bush pasture margins and scattered arboreal habitat within pasture land close to forested areas (B. Hamilton pers obs.).

The poisoning of non-target species, is of concern not only to the Department of Conservation, and the Animal Health Board but, also to the general public.

While any risks to non-targets should be avoided, there is some evidence suggesting that aerial 1080 operations provide benefits to native biodiversity through their reduction in numbers of introduced mammalian pests such as possums, feral cats (*Felis catus*), stoats (*Mustela erminea*), and rats (*Rattus spp.*). Such a reduction in introduced mammal numbers as caused by control operations can result in increased recruitment into the population in seasons following the poison operation for those native species that compete for resources with, or are predated upon by these mammalian pest species. This resultant increased recruitment was shown by Powlesland *et al*. (2000) in North Island tomtits (*Petroica macrocephala toitoi*) in the breeding season following a 1080 aerial operation; Powlesland *et al*. (1999) also found this effect in North Island robins (*P. australis longipes*).

But, studies of the impact on North Island and South Island tomtits of aerial 1080 operations using cereal baits have had varied results. Following a 1080 aerial operation within the Otago region of the South Island did not show a significant increase in recruitment for South Island tomtits (*P. macrocephala macrocephala*) (Hamilton 2005). Therefore, at present it is not known whether such a bounce back effect after 1080 aerial poisoning operation occurs throughout indigenous bird species, including the tomtit.

While not a conservation concern, ~~As~~ the tomtit is known to be an at risk non-target species from aerial 1080 operations (Powlesland *et al*., 2000). Monitoring ~~, while it is not of conservation concern they may provide an indication of~~ the effects of aerial 1080 poison operations on tomtits may provide an indication of the likely effects on other susceptible non-target bird species.

To ascertain if there were long term benefits of 1080 possum control operations on South Island tomtits, monitoring was undertaken in two forested areas in Otago of which one received aerial 1080 in the winter of 2008. This treatment area has in the past been part of a~~n~~ study of the benefits of 1080 possum control operations on the South Island tomtit (Hamilton 2005). Previously, tomtits were monitored for 2 years following an aerial 1080 operation, while this study continued the monitoring at the Hampden site from February 2005 to February 2009. During this time a further aerial 1080 possum control operation occurred in the winter of 2008.

Comment [p1]: This paragraph is not well written and should be part of the methodology anyway.

4 Study Sites

Initial monitoring commenced during February 2005 in two forested areas in Otago. The treatment area, i.e. where aerial 1080 possum control occurswas undertaken, is situated inland from the township of Hampden. This area was poisoned during July 2002, and August 2008. The non-treatment site was located on the northern boundary of Dunedin City, east of State Highway 1. This siteAs this area is located within the part Dunedin City's water catchment area and it has not been part of the AHB's possum poison control programmeoperation in the past.

The Hampden (treatment) site comprising (?) ha is set within coastal hill country in a landscape of plantation forests, indigenous broad-leaved forest and podocarp-mixed broad-leaved forest and pastoral farmland. The plantation forests are mainly Douglas fir (*Pseudotsuga menziesii*) with a mix of radiata pine (*Pinus radiata*). The indigenous forested areas are dominated by large lemonwood (*Pittosporum eugenioides*), broadleaf (*Griselinia littoralis*), three finger (*Pseudopanax colensoi*), marble leaf (*Carpodetus serratus*), pepper tree, mahoe (*Melicytus ramiflorus*), kowhai (*Sophora microphylla*) and lancewood (*Pseudopanax crassifolius*). There are remnants of totara (*Podocarpus totara*), matai (*Prumnopitys taxifolia*) and rimu (*Dacrydium cupressinum*) remaining within these areas. Hampden also has scrubland comprising of kanuka (*Kunzea ericoides*) and manuka (*Leptospermum scoparium*).

The Dunedin (non-treatment) site comprising (?) ha consists of coastal hill country with a similar in composition of habitat aswith the Hampden site. T with the main difference between the sites being that the non-treatmentformer area is not comprised of one large contiguous areas of forest or scrubland. Again the non-treatment site comprised of plantation forest blocks, (radiata pine and Douglas fir), intermixed with indigenous remnants and reserves. The indigenous forest areas are a mixture of broadleaf (Mount Cargill), kaikawaka (*Libocedrus bidwillii*), three finger, mahoe, rimu (*Dacrydium cupressinum*) (Leith Saddle), miro (*Prumnopitys ferruginea*) matai and totara.

Comment [p2]: Isn't there a better way of saying this? For example "This area received aerial 1080 treatment in…"

Comment [p3]: Also state how far away, i.e. "Approximately *y*km to the South of the treatment area."

As for the Hampden area kanuka and manuka scrubland was part of the indigenous mix of this area.

5 Methodology

~~Monitoring involved Distance Sampling (DS) methodology and followed that of Westbrooke *et al*. (2003) and Hamilton (2005).~~

5.1 Possum Control

~~5.1~~5.2 Distance Sampling

Monitoring involved Distance Sampling (DS) methodology and followed that of Westbrooke *et al*. (2003) and Hamilton (2005).

DS is a method of estimating the absolute density of a population. For detailed descriptions of the methodology and DS theory see Buckland *et al.* (1993, 2001); Barraclough (2000); Moffat & Minot (1994); Powlesland & Barraclough (2001).

In D.S. either a transect or point sampling, data collection methodology is used. For the purposes of this study the line transect method was selected.

To obtain the required minimum of 50 tomtit detections (birds seen or heard) per sampling effort (Buckland *et al*., 2001), 45 transects were established at each study site during February 2005.

Transects were 250 metres long. Transect along the same compass bearing were separated from each other by at least 100 metres. This separation distance represented twice the estimated radius of a tomtit's territory, thus bird registrations at each transect are assumed to be independent of each other. To ensure that tomtit territories were not crossed by more than one transect a parallel distance of at least 200 metres was left between transects not on the same compass bearing. Transect length was initially measured using a hip chain and transects start and finish marked using plastic tie markers. Transects positions were also recorded using a hand held GPS. Markers were removed following the study.

Comment [p4]: Need a section here on the possum control, i.e. "Aerial 1080 at a rate of xkg/ha of cereal pellets was applied at the Hampden treatment site on…" Also, was any ground control undertaken at the non-treatment site? If so, need to specify.

Formatted: Bullets and Numbering

Comment [p5]: Also need to include frequency with which lines were counted and the date periods when counting occurred.

Accurate distance measurement of individuals from a perpendicular distance from a transect line are essential. While walking along a transect, any tomtit seen or heard within 50 m of the transect, was recorded ~~with its~~together with the perpendicular distance to the line. Prior to and during a pilot study, the observers who did the field observations learnt to recognise tomtit calls, and to accurately estimate the distance of birds from the line by measuring distances using a hip chain and laser finder.

As for other bird sampling methods weather and noise conditions can affect audio and visual cues important in distance estimates. Therefore counts could not be performed during moderate to heavy rainfall, strong winds and/or during forestry operations. Additionally no count data was collected within an hour of dawn or dusk as these are peak bird calling periods and counting during these times could bias results.

~~5.2~~5.3 Analyses

Formatted: Bullets and Numbering

Using the established transects at each site, *t*-test were used to analyse the changes in abundance between seasons.

Data analysis of tomtit density was undertaken using the ‘Distance’ software (Thomas *et al.*, 2009). Here the model that best fitted the data was selected according to a low Akaike Information Criterion (AIC) (Akaike, 1985) and low variance (see “Bird Density” below for further description of the methods).

6 Results

6.1 Mean counts between poison operations

For the survey periods pre-winter 2008 poisoning there was on average 1.74 (se = 0.08) bird detections per transect within the Hampden treatment area during summer, 1.64 (se = 0.08) in winter, and 1.20 (se = 0.06) bird

detections per transect within the Dunedin non-treatment area during summer and 1.24 (se = 0.08) during winter (Figure 1).

Treatment Area

	Summer	Winter
Pre-treatment	1.74 (±0.08)	1.64 (±0.08)
Post-treatment	?	1.27 (±0.16)

The post poisoning results for the treatment area showed a marked decrease in numbers during the winter directly following the operation with 1.27 (se = 0.16) bird detections per transect. While there was no comparable difference seen between the summer season following the 2008 winter poisoning and the previous averaged summer data with 1.58 (se = 0.15) bird detections per transect at Hampden. Compared with 1.74 (se = 0.~~.~~08) bird detections per transect within the Hampden treatment area during summer.

There was a significant difference in the number of birds/transect during winter at Hampden between pre and post poisoning (178 df; p = 0.01). In ~~but~~ comparison, ~~of~~ the summer values at the Hampen ~~is~~ site ~~during pre and post poisoning~~ showed no differences (223 df; p = 0.17) between pre and post poisoning~~)~~. There were no differences between comparable seasons pre~~post~~ and post~~pre~~ poisoning at the Dunedin non-treatment site with winter comparisons being (178 df; p = 0.30) and ~~the~~ summer ~~values showing no differences~~being (223 df; p = 0.05).

Comment [p6]: This is confusing to read and maybe should be shown in a 2x2 table format? See example for treatment area. Would need to do another one for the non-treatment area.

Comment [p7]: Isn't this explained further down and therefore unnecessary duplication?

Comment [p8]: Again, this doesn't read properly.

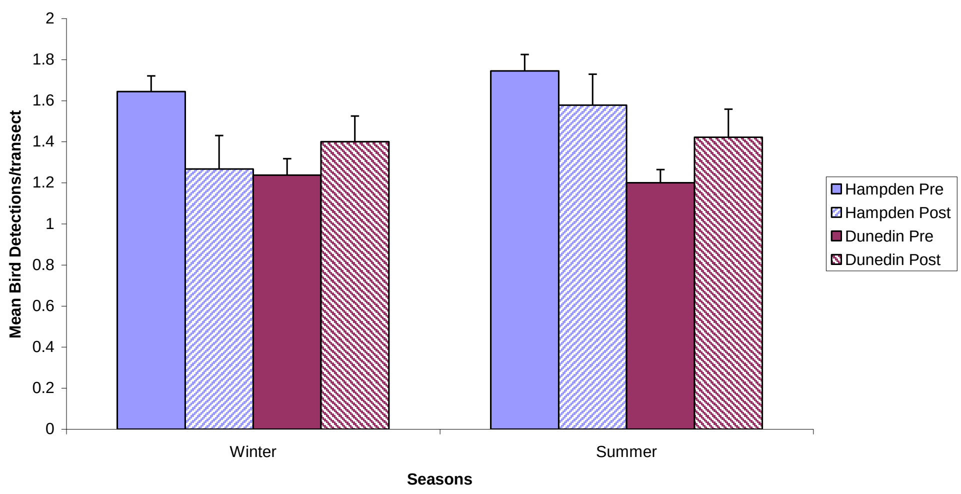

Figure 1: Mean number of bird detections/transect during winter and summer pre- and post- poisoning at both sites. Note that the non-treatment pre- and post-poison category is for comparison only and no poisoning occurred

For all survey periods except the winter immediately following the aerial 1080 control operation more birds were registered at the treatment (Hampden) site than at the non-treatment (Dunedin) site. This difference was significant during summer 06, 07 and 08 and winter 07 with a higher number of registrations at the treatment site (Figure 2).

Significant reduction in bird counts at the treatment site occurred between the summer 07 and winter 07 and between summer 08 and winter 08. This latter reduction in bird numbers occurred after the 1080 aerial poison operation.

Comment [p9]: Is this really true? What was the p-value? From the CI's it doesn't look as though it was statistically significant.

Comment [p10]: Again need to include p-value.

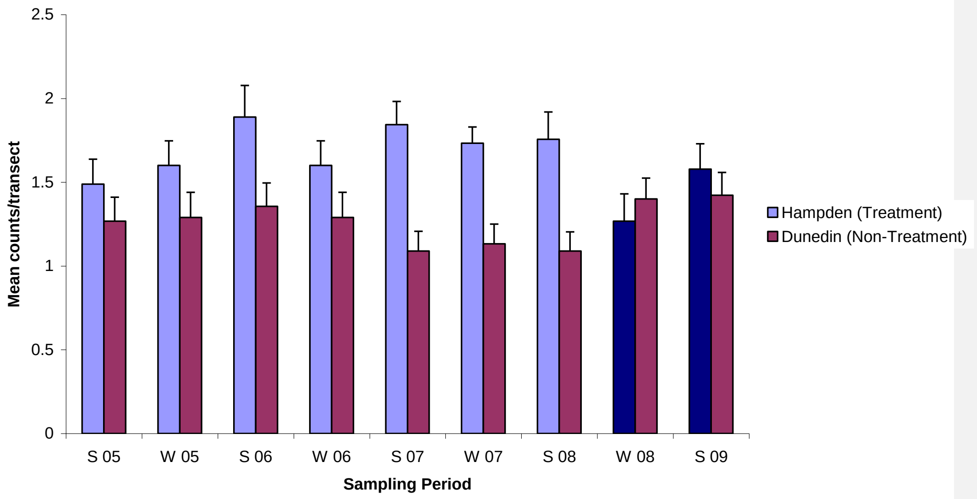

Figure 2: Mean number of bird counts/transect at study sites over the nine survey periods, summer and winter 2005 to summer 2008 (pre-2008 poisoning), with winter 2008 and summer 2009 (post-2008 poisoning). Sample periods are abbreviated with S and W representing the summer and winter periods respectively. Post poisoning winter 2008 survey periods are shown in darker blue.

6.2 Bird density

DS selects a model to best fit the data given. The models involve selecting whether to truncate the data at some set distance, what shape detection function to use and whether data should be grouped differently than provided initially in the programme.

For all the data the model was based on the achievable minimum Akaike's Information Criterion (AIC), and data groupings selected to include peaks of bird registrations. This parameter is used in model selection and is based on the Kullback-Leibler "distance" between two distributions. The model also relied upon data being grouped to include peaks of bird registrations. Generally the distance programme was left to select the detection function

based on the minimum AIC but during several sampling seasons data were truncated to give the best fit and lowest AIC. This truncation occurred at both sites (Table 1).

Table 1: Distance parameters for the truncated treatment and non-treatment samples. Distance parameters include:

- Akaike's Information Criterion (AIC), This parameter is used in model selection and is based on the Kullback-Leibler "distance" between two distributions.
- Density, birds detected per hectare; derived from distance sampling software
- Truncation model indicates which model was selected by DS data analyses.

Site	Sampling Period	Chosen AIC	Density	Truncation Model
Dunedin	Summer 05	446	0.9	1
	Winter 05	456	1.1	2
	Summer 06	429	2.1	2
	Summer 07	313	1.8	1
	Winter 07	376	1.7	2
	Summer 08	350	1.6	2
	Summer 09	468	1.9	2
Hampden	Summer 05	500	1.8	2
	Winter 08 (post Poison)	434	1.3	2

Comment [p11]: What happened to the data between Summer 05 and this date?

The distance histograms for all samples showed that there is a peak in observations close to the line.

Comment [p12]: Where?

For the ~~Otago~~ non-treatment(?) site~~s~~ an observational peak close to the transect line suggests that tomtits are being attracted to the observer. This is not evident in North Island tomtits where distance histograms show more of a bow-wave. This has been interpreted as resulting from tomtit observer avoidance.

Comment [p13]: Where is this data shown?

Comment [p14]: Need a reference to support this.

While there is a peak close to the observer in this study, gradual tail off out to the full 50 metres suggests that birds are not all drawn in. Similarly to the

North Island studies there is confidence that almost all birds close to the line were detected as per the assumptions of DS.

In the comparable season following the poison operation there was a change of – 0.6 and +0.9 (birds/ha) in the treatment area over the winter and summer seasons respectively (Table 3). This corresponded to a 32% decrease in bird density between the winters of 2007 and 2008. There was a increase of 38% between the summers at the treatment site. The increase in bird density is more marked if across seasons is calculated. While there was a marked drop off in bird density in winter 2008 directly following the 1080 aerial poison operation, bird density the following summer increased by over 250%, this change was significant (Figure 3). This increase in tomtit density was also significantly larger than any other winter summer increases during the study period.

For the non-treatment site tomtit densities were unchanged between winter counts, increased by 19% over summer and showed an increase of 12% between winter 2008 and summer 2009.decreased over comparable seasons and corresponded to a 60.9% (between winter 2002 and 2003) and 22.2% (between summer 2003 and 2004) reduction (Table 3). These changes in bird densities between and across seasons were not significant (Figure 3).

Comment [p15]: Wording not clear and confusing for the reader.

Table 3: Distance sampling tomtit density estimates between similar and adjoining seasons at the non-treatment and treatment sites.

Comment [p16]: If this is based on Density software then need to state as such.

	Hampden (treatment) birds/ha	Change	Dunedin (non-treatment) birds/ha	Change
Winter 2007	1.9		1.7	
Winter 2008	1.3		1.7	
		-0.6		0.0
Summer 2008	2.4		1.6	
Summer 2009	3.3		1.9	
		+0.9		+0.3
Winter 2007	1.9		1.7	
Summer 2008	2.4		1.6	
		+0.5		-0.1
Winter 2008	1.3		1.7	
Summer 2009	3.3		1.9	
		+2.0		+0.2

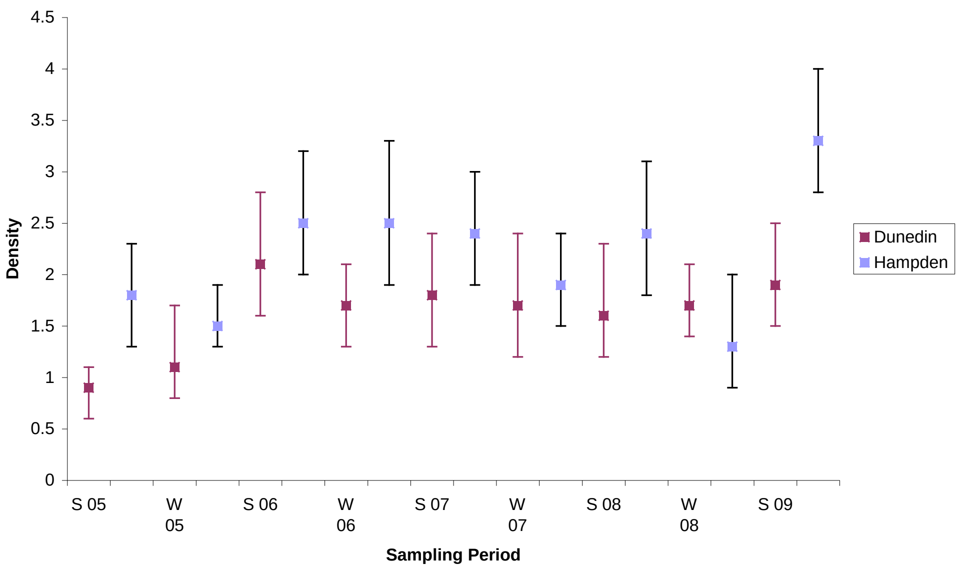

Figure 3: Tomtit density with upper and lower confidence intervals during 9 sampling periods at both the treatment and non-treatment sites.

Comment [p17]: See comment for Table 3 above.

Bird density in the breeding season following the 2008 aerial 1080 operation was not significantly different from the summer 2003 breeding season which also followed an aerial 1080 operation (Figure 4).

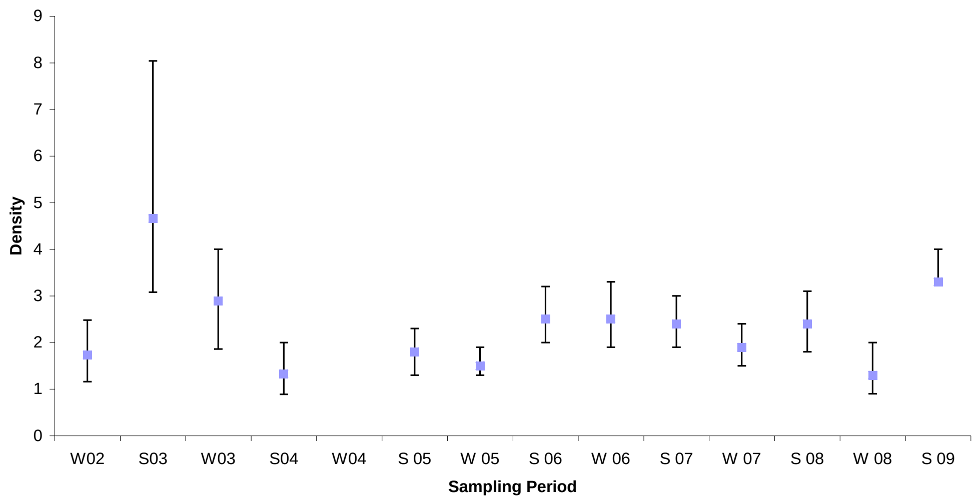

Figure 4: Tomtit density with upper and lower confidence intervals for the Hampden treatment site showing the 9 sampling periods of this study and including data from the Hamilton 2005 study.

Comment [p18]: Would be helpful to state, or show on graph, when possum control occurred.

7 Discussion

From this study it would appear that while gross bird counts per transect dropped off immediately following the poison operation at Hampden this was not the case with their overall density which showed a significant increase. This increase in tomtit density was over 250% in the breeding season immediately following the 1080 operation. This equates with an increase of 2 birds/hectare within the treatment area and while there was an increase in density during this time at the non-treatment site density only increased by 0.2 birds/hectare, a tenth of the increase seen at Hampden.

The significant increase in bird density in the breeding season immediately following an aerial 1080 operation was also seen in the Hamilton 2005 study. Here there an increase of almost 3 birds/hectare occurred at the same treatment site in the first breeding season following the aerial control operation. The results from this study and that of the 2005 study show that there is a marked increase in bird density immediately following aerial 1080 poison control operations. This initial increase in bird density tapers off to a level which may indicate the area habitat carrying capacity has been reached. Following this initial increase there are further fluctuations in population density occurring both seasonally and yearly.

Comparison between the non-treatment (Dunedin) and treatment (Hampden) sites show that the Dunedin area also has similar fluctuations in population density seasonally and yearly. But the population density in the non-treatment area is generally lower than the treatment area. Only in the season immediately following a poison control operation did the treatment density drop below that of the non-treatment area. Even then there was no significant difference between bird densities. This suggests that in these comparable sites some factor other than habitat is limiting the tomtit density within the non-treatment site. It is possible that the removal of introduced mammalian pests through the pest control operations is giving the treatment areas a

boost immediately following the operations and the removal of such pests results in a higher carrying capacity of the habitat.

Therefore, while the 2005 (Hamilton) study was unclear on whether the removal of pest species by aerial 1080 operations had a long-term conservation benefit for indigenous species such as the tomtit, these data collected during this study suggests that this could be occurring.

As in the previous study within the Hampden area distance sampling suggests that here tomtits appear to be attracted towards the observer. This movement towards the observer and thus the transect line, is in contradiction to what was found by Westbrooke *et al*. (2003). Unlike the 2005 (Hamilton) study this attracted behaviour was also evident at the Dunedin non-treatment site. It was suggested previously that the closed in habitat at Hampden did not allow for the detection of a high number of birds far away from the transect line. Therefore, as the Dunedin site also had a comparable closed in habitat over much of the area it is possible that distance sampling may be slightly affected by habitat variables. But, as DS detection models showed a high percentage of birds close to the line had been detected so DS assumptions were met and the methodology was appropriate.

As in the previous study interested parties contacted the observers relaying information as to large number of tomtit carcasses being found on the ground following the aerial 1080 operation. Again there was no evidence of such large deaths seen by the observer along the transect lines within Hampden in the weeks following the 1080 operation.

8 References

Akaike, H. 1985. Prediction and entropy. Pages 1-24 A. C. Atkinson, and S. E. Fienberg *in* (Eds.) . Springer, New York, NY. *A celebration of statistics*

Barraclough, R.K. 2000. Distance sampling: A discussion document produced for the Department of Conservation. *Science and Research internal report 175.* 26 pp.

Brockie, R. 1992. A living New Zealand forest. David Bateman Ltd., Auckland, New Zealand. pp 172.

Buckland, S.T., Anderson, D.R., Burnham, K.P, Laake, J.L. 1993. *Distance sampling: estimating abundance of biological populations.* Chapman and Hall, London. 446 pp.

Buckland, S T, Anderson, D.R, Burnham, K P, Laake, J L, Borchers, D L, Thomas, L 2001. *Introduction to Distance Sampling: estimating abundance of biological populations*. Oxford University Press. 448 pp.

Coleman, J.; Caley, P. 2000: Possums as a reservoir of bovine Tb. Pp. 92–104 in Montague, T.L. (Ed.): The brushtail possum: biology, impact and management of an introduced marsupial. Manaaki Whenua Press, Lincoln, New Zealand.

Cowan, P. 2001. Advances in New Zealand Mammalogy 1990 – 2000: brushtail possum. . *Journal of the Royal Society of New Zealand*. 31: 15-29.

Eason, C.T., Frampton, C.M., Henderson, R., Thomas, M.D., Morgan, D.R. 1994. Sodium monofluoroacetate and alternative toxins for possum control. *New Zealand Journal of Zoology*. 20: 329-334.

Heather, B.; Robertson, H. 1996. *Field guide to the birds of New Zealand.* Auckland, Viking

Hamilton, B. 2005. Effects of an aerial 1080 carrot bait operation on South Island tomtit (*Petroica macrocephala macrocephala*) populations within the

Hampden possum control operational area of Otago. Animal Health Board Research Report R-80574. Wellington, New Zealand.

.Livingstone, P.G. 1994. The use of 1080 in New Zealand. *In:* Seawright, A.A.; Eason, C.T. (Editors), *Proceedings of the science workshop on 1080*, pp. 1-9. The Royal Society of New Zealand, Wellington, N.Z.

Moffat, M., Minot, E.O. 1994. Distribution and abundance of forest birds in the Ruamahanga Ecological Area, North Island, New Zealand. *New Zealand journal of zoology 21:*135–150.

Morgan, D.R.; Thomas, M.D.; Meeken, D.; Nelson, P.C. 1997. Less 1080 bait useage in aerial operations to control possums. *Proceedings of the 50th New Zealand Plant Protection Society Conference:* 391-396.

Powlesland, R.G.; Knegtmans, J.W.; Marshall, I. 1999. Costs and benefits of aerial 1080 possum control operations using carrot baits to North Island robins (*Petroica australis longipes*), Pureora Forest Park. *New Zealand Journal of Ecology 23:* 149-159.

Powlesland, R.G., Knegtmans, J.W., Styche, A. 2000. Mortality of North Island tomtits (*Petroica macrocephala toitoi*) caused by aerial 1080 possum control operations, 1997 – 98, Pureora Forest Park. *New Zealand Journal of Ecology* 24: 161-168.

Powlesland, R.G., Barraclough, R.K. 2001. Proceedings of a workshop on distance sampling, Wellington, May 2000. *Conservation Advisory Science Notes No. 329*, Department of Conservation, Wellington.

Spurr, E.B. 1991. Effects of brushtail possum control operations on non-target bird populations. *Acta XX Congressus Internationalis Ornithologici* 2534-2545.

Spurr. E.B., Powlesland, R.G. 1997. Impacts of aerial application of 1080 on non-target native fauna. *Science for Conservation* No 62. Department of Conservation, Wellington. Pp 31.

Thomas, L., Laake, J.L., Rexstad, E., Strindberg, S., Marques, F.F.C., Buckland, S.T., Borchers, D.L., Anderson, D.R., Burnham, K.P., Burt, M.L., Hedley, S.L., Pollard, J.H., Bishop, J.R.B. and Marques, T.A. 2009. Distance

6.0. Release "x"[1]. Research Unit for Wildlife Population Assessment, University of St. Andrews, UK. http://www.ruwpa.st-and.ac.uk/distance/

Veltman, C.J.; Pinder, D.N. 2001. Brushtail possum mortality and ambient temperatures following aerial poisoning using 1080. *Journal of Wildlife Management 65:* 476-481.

Westbrooke, I.M., Powlesland, R.G. 2005. Comparison of impact between carrot and ceral 1080 baits on tomtits *(Petroica macrocephala)*. *New Zealand Journal of Ecology 29:* 143-147

Westbrooke, I.M., Etheridge, N.D., Powlesland, R.G. 2003. Comparing methods for assessing mortality impacts of an aerial 1080 pest control operation on tomtits (*Petroica macrocephala toitoi*) in Tongariro Forest. *New Journal of Ecology* 2: 115-123.

9 Acknowledgements

I would like to thank Bruce Kyle of the Department of Conservation for his time, help patience and experience. My thanks also go to Joe Bailey and Andrew Win of Southern Pest management who provided much of the information on the 1080 aerial operations. I would also like to thank Penny Fairbrother for her extreme patience while the draft was being submitted and her help in ensuring that the results were given proper airing. Also I thank Paul Livingstone for his ideas and incentive that made this research possible and hopefully has cleared up some misconceptions about 1080 aerial control operations and non-target species.

Last but not least a thank you to the Animal Health Board for their funding, without which none of this would have been possible.
