# Supplementary material for: Responses of New Zealand forest birds to management of introduced mammals
Source: Conserv Biol. 2020 Mar 23;35(1):35–49. doi: 10.1111/cobi.13456 (PMC7984369; doi:10.1111/cobi.13456)
Supplement: Supplementary file 12 — Supporting Material [file COBI-35-35-s012.docx]

**Appendix S19.** Tongariro data supplied by Jerome Guillotel (19-06-2017).

|  | | |  |  |  |  |
| --- | --- | --- | --- | --- | --- | --- |
| Year | 2002 | 2003 | 2004 | 2009 | 2010 | 2011 |
| N | 226 | 193 | 200 | 210 | 216 | 216 |
| Kereru sum | 85 | 40 | 64 | 79 | 88 | 157 |
| Kereru mean | 0.376106 | 0.207254 | 0.32 | 0.37619 | 0.407407 | 0.726852 |
| Kereru SD | 0.733427 | 0.466089 | 0.670708 | 0.584001 | 0.818814 | 0.870403 |
| Kereru SE | 0.048787 | 0.03355 | 0.047426 | 0.065705 | 0.087286 | 0.069466 |
| Kaka sum | 3 | NA | NA | NA | NA | NA |
| Kaka mean | 0.013274 | NA | NA | NA | NA | NA |
| Kaka SD | 0.148476 | NA | NA | NA | NA | NA |
| Kaka SE | 0.009877 | NA | NA | NA | NA | NA |
| Tui sum | 433 | 228 | 253 | 188 | 196 | 197 |
| Tui mean | 1.915929 | 1.181347 | 1.265 | 0.895238 | 0.907407 | 0.912037 |
| Tui SD | 2.01263 | 1.12882 | 1.289609 | 1.070919 | 1.048028 | 1.010017 |
| Tui SE | 0.133878 | 0.081254 | 0.091189 | 0.078105 | 0.074859 | 0.071961 |
| Kakariki sum | 1 | NA | NA | NA | NA | NA |
| Kakariki mean | 0.004425 | NA | NA | NA | NA | NA |
| Kakariki SD | 0.066519 | NA | NA | NA | NA | NA |
| Kakariki SE | 0.004425 | NA | NA | NA | NA | NA |
| Bellbird sum | 157 | 89 | 128 | 141 | 195 | 106 |
| Bellbird mean | 0.69469 | 0.46114 | 0.64 | 0.671429 | 0.902778 | 0.490741 |
| Bellbird SD | 1.107417 | 0.735833 | 0.885619 | 0.902756 | 0.947355 | 0.721695 |
| Bellbird SE | 0.073664 | 0.052966 | 0.062623 | 0.076026 | 0.067841 | 0.070097 |
| Robin sum | 44 | 70 | 30 | 41 | 51 | 61 |
| Robin mean | 0.19469 | 0.362694 | 0.15 | 0.195238 | 0.236111 | 0.282407 |
| Robin SD | 0.468727 | 0.606439 | 0.410295 | 0.442888 | 0.523665 | 0.527271 |
| Robin SE | 0.031179 | 0.043652 | 0.029012 | 0.069167 | 0.073328 | 0.06751 |
| Whitehead sum | 90 | 100 | 283 | 176 | 221 | 445 |
| Whitehead mean | 0.39823 | 0.518135 | 1.415 | 0.838095 | 1.023148 | 2.060185 |
| Whitehead SD | 0.87473 | 0.771182 | 1.446804 | 1.063746 | 1.309863 | 1.567427 |
| Whitehead SE | 0.058186 | 0.055511 | 0.102304 | 0.080183 | 0.088111 | 0.074303 |
| Silvereye sum | 274 | 72 | 131 | 150 | 193 | 213 |
| Silvereye mean | 1.212389 | 0.373057 | 0.655 | 0.714286 | 0.893519 | 0.986111 |
| Silvereye SD | 1.720214 | 0.673687 | 1.10548 | 1.112902 | 1.428206 | 1.341569 |
| Silvereye SE | 0.114427 | 0.048493 | 0.078169 | 0.090868 | 0.102805 | 0.091923 |
| Tomtit sum | 112 | 165 | 278 | 107 | 101 | 162 |
| Tomtit mean | 0.495575 | 0.854922 | 1.39 | 0.509524 | 0.467593 | 0.75 |
| Tomtit SD | 0.920134 | 0.691985 | 1.040825 | 0.72045 | 0.631621 | 0.716905 |
| Tomtit SE | 0.061206 | 0.04981 | 0.073597 | 0.069648 | 0.062849 | 0.056325 |
| Fantail sum | 91 | 96 | 69 | 9 | 22 | 63 |
| Fantail mean | 0.402655 | 0.497409 | 0.345 | 0.042857 | 0.101852 | 0.291667 |
| Fantail SD | 0.68104 | 0.630181 | 0.581088 | 0.203019 | 0.359323 | 0.504053 |
| Fantail SE | 0.045302 | 0.045361 | 0.041089 | 0.067673 | 0.076608 | 0.063505 |
| Rifleman sum | 21 | 6 | 7 | 0 | 17 | 5 |
| Rifleman mean | 0.09292 | 0.031088 | 0.035 | 0 | 0.078704 | 0.023148 |
| Rifleman SD | 0.427128 | 0.20173 | 0.253171 | NA | 0.358693 | 0.178941 |
| Rifleman SE | 0.028412 | 0.014521 | 0.017902 | NA | 0.086996 | 0.080025 |
| Gwarbler sum | 212 | 118 | 217 | 67 | 146 | 109 |
| Gwarbler mean | 0.938053 | 0.611399 | 1.085 | 0.319048 | 0.675926 | 0.50463 |
| Gwarbler SD | 0.991369 | 0.636784 | 0.831403 | 0.543002 | 0.833359 | 0.609977 |
| Gwarbler SE | 0.065945 | 0.045837 | 0.058789 | 0.066338 | 0.068969 | 0.058425 |
| Blackbird sum | 189 | 10 | 39 | 23 | 13 | 13 |
| Blackbird mean | 0.836283 | 0.051813 | 0.195 | 0.109524 | 0.060185 | 0.060185 |
| Blackbird SD | 0.954387 | 0.244543 | 0.546322 | 0.355954 | 0.274646 | 0.274646 |
| Blackbird SE | 0.063485 | 0.017603 | 0.038631 | 0.074222 | 0.076173 | 0.076173 |
| Chaffinch sum | 59 | 28 | 109 | 18 | 26 | 10 |
| Chaffinch mean | 0.261062 | 0.145078 | 0.545 | 0.085714 | 0.12037 | 0.046296 |
| Chaffinch SD | 0.764773 | 0.432639 | 1.529599 | 0.327796 | 0.456836 | 0.268822 |
| Chaffinch SE | 0.050872 | 0.031142 | 0.108159 | 0.077262 | 0.089593 | 0.085009 |
