# Supplementary material for: Responses of New Zealand forest birds to management of introduced mammals
Source: Conserv Biol. 2020 Mar 23;35(1):35–49. doi: 10.1111/cobi.13456 (PMC7984369; doi:10.1111/cobi.13456)
Supplement: Supplementary file 13 — Supporting Material [file COBI-35-35-s013.docx]

**Appendix S20a.** Values and calculations for areas receiving low intensity mammal control in New Zealand.

| **Low intensity mammal control (LIMC) in New Zealand** | | | | |
| --- | --- | --- | --- | --- |
| **Native Forest** | **Total area (ha)** | **% protected** | **Source** | **Area with formal protection (ha)** |
| Type: |  |  |  |  |
| Indigenous forest | 6368140 | 80.3 | Cieraad et al. (2015) | 5113616 |
| Manuka and kanuka | 1169320 | 31.1 | Cieraad et al. (2015) | 363659 |
| Broadleaved and indigenous hardwoods | 581850 | 35.4 | Cieraad et al. (2015) | 205975 |
| Sub-alpine shrubland | 423800 | 81.1 | Cieraad et al. (2015) | 343702 |
| Total |  |  |  | 6026952 (A) |
|  |  |  |  |  |
|  | **Treated area (ha)** | % of DOC managed land under LIMC (=B/C) |  |  |
| Total DOC area under LIMC | 1042000 (B) | 12.7 | EPA (2014) |  |
| Percentage of formally protected native forest under LIMC^a^  (=B/A) |  | 17.3 |  |  |
| **Sustained possum control**  **(aerial and ground treatment)** |  | **% of total NZ^b^** |  |  |
|  | 11000000 | 41.0 | Parkes et al. (2017) |  |
| Vertebrate pest control | 10000000 | 37.26 | Byrom et al. (2016) |  |
| Control of invasive mammals | 11800000 | 43.96 | Russell et al. (2015) |  |
| **Formally protected land** ^a^ | **Total area (ha)** | **% of formally protected land** |  |  |
| Agency: |  |  |  |  |
| DOC | 8200000 (C) | 96.5 | Parkes et al. (2017) |  |
| Maori-owned | 154000 | 1.8 |  |  |
| QE II Trust (private land) | 86000 | 1.0 |  |  |
| Regional councils | 62000 | < 1 |  |  |
| Total | 8502000 |  |  |  |

^a^ This figure is based on the assumption that the majority of forests where possums are targeted is native forest habitat, and that negligible areas of low-intensity mammal control are conducted on land that is not managed by the Department of Conservation (DOC)

^b^ Total land area of New Zealand is 26842401 ha ([http://archive.stats.govt.nz](http://archive.stats.govt.nz/))

**Appendix S20b.** Values and calculations for the area receiving high intensity mammal control in New Zealand.

| **High intensity mammal control (HIMC) in New Zealand** | **Treated area (ha)** |
| --- | --- |
| Mainland Islands  from Saunders and Norton (2001) | 19022 |
| Mammal-free offshore islands  from Parkes et al. (2017) | 61000 |
| Sanctuaries NZ – additional sites  (excluding offshore islands and mainland islands listed above)^a^ | 128650 |
| Total area under HIMC | 208672 |
| Percentage of NZ area under HIMC^b^ | 0.78% |

^a^ This figure excludes areas that are mainland islands and mammal-free offshore islands as we use different sources to estimate area protected as mainland islands (Saunders & Norton 2001) and on offshore islands (Parkes et al. 2017)

^b^ Total land area of New Zealand is 26842401 ha ([http://archive.stats.govt.nz](http://archive.stats.govt.nz/))

**Appendix S20c.** Projects receiving high intensity mammal control in New Zealand.

| **Mainland islands (MI)*** | **Site name** | **Treated area (ha)** | **Notes** |
| --- | --- | --- | --- |
| from Saunders and Norton (2001) | Eglinton Valley (Walker Creek and knobs Flat) | 2000 |  |
|  | Rotoiti Nature Recovery Project | 825 | core area only |
|  | Project River Recovery | 2500 |  |
|  | Hinewai | 980 |  |
|  | Hurunui | 6000 |  |
|  | Zealandia (the Karori Sanctuary Experience) | 225 |  |
|  | Boundary Stream Mainland Island Nature Restoration Project | 802 |  |
|  | Northern Te Urewera | 2500 |  |
|  | Trounson Kauri Park | 586 | now > 450 ha |
|  | Mapara | 1200 |  |
|  | Paengaroa Reserve | 117 |  |
|  | Bushy Park | 87 |  |
|  | Mount Stokes | 1200 |  |
|  | **Subtotal:** | **19022** |  |
|  |  |  |  |
| **Sanctuaries NZ** | **Project name** | **Treated area (ha)** | **Notes** |
| from the Sanctuaries NZ (2019) website | Aongatete Forest Restoration Project | 480 |  |
|  | Arthur's Pass Wildlife Trust | 405 |  |
|  | Ark In The Park | 2000 |  |
|  | Bluff Hill | 630 |  |
|  | Boundary Stream Mainland Island Nature Restoration Project | - | listed as MI above* |
|  | Bream Head Conservation Trust | 830 |  |
|  | Brook Waimarama Sanctuary | 715 |  |
|  | Bushy Park | - | listed as MI above* |
|  | Bushy Point Restoration Project | 14 |  |
|  | Cape Kidnappers | 2500 |  |
|  | Cleddau Delta Restoration Project | 40 |  |
|  | East Harbour - Mainland Island Restoration Organisation (MIRO) | 2360 | Northern forest block 2000 ha, Parangarahu Lakes 360 ha |
|  | East Taranaki Environment Trust | 13000 |  |
|  | Eglinton Valley (Walker Creek and knobs Flat) | - | listed as MI above* |
|  | Friends of Flora Inc | 5500 |  |
|  | Habitat te Henga | 600 |  |
|  | Halfmoon Bay Habitat Restoration Project | 210 |  |
|  | Hauturu / Little Barrier Island | - | excluded from subtotal** |
|  | Kaipupu Point Mainland Island | 40 |  |
|  | Kapiti Island Nature Reserve | - | excluded from subtotal** |
|  | Kepler Peninsular Conservation Project | 3000 |  |
|  | Kotuku Peninsula Sanctuary (Glenfern Sanctuary Charitable Trust) | 240 |  |
|  | Landsborough Valley | 900 |  |
|  | Longbush Ecosanctuary | 110 | aka Waikereru Ecosanctuary |
|  | Mahakirau Forest Estate | 580 |  |
|  | Mamaku Point Conservation Reserve | 160 |  |
|  | Mana Island | - | excluded from subtotal** |
|  | Matakohe / Limestone Island | 39 |  |
|  | Matiu/Somes Island Scientific and Historic Reserve | - | excluded from subtotal** |
|  | Maungataniwha | 6120 |  |
|  | Maungatautari Ecological Island | 3400 |  |
|  | Tuhua / Mayor Island | - | excluded from subtotal** |
|  | Mokoia Island | - | excluded from subtotal** |
|  | Motu Kaikoura Trust | 564 |  |
|  | Motuihe Island | - | excluded from subtotal** |
|  | Motuora Island | - | excluded from subtotal** |
|  | Ngapukeariki Mainland Island Project | 40 |  |
|  | Orokonui EcoSanctuary | 307 |  |
|  | Otamahua / Quail Island | 81 |  |
|  | Otanewainuku Kiwi Trust | 20 |  |
|  | Otari Native Botanic Garden and Wilton's Bush | 105 |  |
|  | Paengaroa Mainland Island | - | listed as MI above* |
|  | Parininihi | 2000 |  |
|  | Pirongia te Aroaro o Kahu Restoration Society | 880 |  |
|  | Pomona Island | 262 |  |
|  | Port Charles Rat Attack (Moehau Environment Group) | 3200 |  |
|  | Project Island Song - Ipipiri / Eastern Bay of Islands restoration | 600 |  |
|  | Project Janszoon | 22530 |  |
|  | Project Kaka | - | 22000 ha, discontinued 2017 |
|  | P?kaha National Wildlife Centre | 942 |  |
|  | Pukenui Forest | 400 | 440 ha rats, 2000 ha stoats |
|  | Puketi Forest | 5500 |  |
|  | Pupu Rangi Nature Sanctuary | 100 |  |
|  | Rangitoto & Motutapu Islands Restoration Project | - | excluded from subtotal** |
|  | Rotokare Scenic Reserve Trust | 230 |  |
|  | Rotopiko / Lake Serpentine Sanctuary | 40 |  |
|  | Rotoroa Island | - | excluded from subtotal** |
|  | Shakespear Open Sanctuary | 500 |  |
|  | Taranaki Mounga | 13870 |  |
|  | Tasman River | 7200 |  |
|  | Taurikura Ridge Possum and Predator Control project | 6000 | i.e. the peninsula at Whangarei Heads |
|  | Tawharanui Open Sanctuary | 588 |  |
|  | Te Kauri – Waikuku Trust | 1100 |  |
|  | Te Puka-Hereka / Coal Island | 1163 |  |
|  | Te Urewera Mainland Island | - | listed as MI above* |
|  | Tiritiri Matangi Island | - | excluded from subtotal** |
|  | Totara Reserve Regional Park | 340 |  |
|  | Trounson Kauri Park | - | listed as MI above* |
|  | Turitea Reserve | 600 |  |
|  | Ulva Island | - | excluded from subtotal** |
|  | Waikawau Bay Wetland Project | 75 |  |
|  | Wainuiomata Water Supply | 7600 |  |
|  | Waipapa Ecological Area | 1300 |  |
|  | Wairakei Golf Course | 180 |  |
|  | Wenderholm Regional Park | 134 |  |
|  | Whakaangi Landcare Trust | 1346 |  |
|  | Whakatane Kiwi Project | 2500 | core area is Ohope Scenic Reserve |
|  | Whinray - Motu Restoration Project | 430 |  |
|  | Windy Hill – Rosalie Bay | 700 |  |
|  | Young Nicks Head | 1350 |  |
|  | Zealandia (the Karori Sanctuary Experience) | - | listed as MI above* |
|  | **Subtotal:** | **128650^a^** |  |
|  |  |  |  |
| **Offshore islands (IE)**** | **Project name** | **Treated area (ha)** | **Notes** |
| from Sanctuaries NZ (2019) website | Hauturu / Little Barrier Island | 2817 |  |
|  | Kapiti Island Nature Reserve | 1965 |  |
|  | Mana Island | 217 |  |
|  | Matiu/Somes Island Scientific and Historic Reserve | 25 |  |
|  | Tuhua / Mayor Island | 1277 |  |
|  | Mokoia Island | 135 |  |
|  | Motuihe Island | 179 |  |
|  | Motuora Island | 80 |  |
|  | Pomona Island | 262 |  |
|  | Rangitoto & Motutapu Islands Restoration Project | 3811 | Rangitoto 2311 ha, Motutapu 1500 ha |
|  | Rotoroa Island | 82 |  |
|  | Tiritiri Matangi Island | 254 |  |
|  | Ulva Island | 267 |  |

^a^ This figure excludes areas that are mainland islands and mammal-free offshore islands as we use different sources to estimate area protected as mainland islands (Saunders & Norton 2001) and on mammal-free offshore islands (Parkes et al. 2017)

^b^ Total land area of New Zealand is 26842401 ha ([http://archive.stats.govt.nz](http://archive.stats.govt.nz/))

**Appendix S20.** Current coverage of mammal control across New Zealand.

**Literature Cited**

Byrom AE, Innes J, Binny RN. 2016. A review of biodiversity outcomes from possum-focused pest control in New Zealand. Wildlife Research **43**:228-253.

Cieraad E, Walker S, Price R, Barringer J. 2015. An updated assessment of indigenous cover remaining and legal protection in New Zealand's land environments. New Zealand Journal of Ecology **39**:309-315.

EPA. 2014. Annual Report on the Aerial Use of 1080. For the year ended 31 December 2014. Page 26. Environmental Protection Authority, Wellington, New Zealand. Available from <https://www.epa.govt.nz/assets/RecordsAPI/94365e9cf1/EPA-annual-report-on-aerial-1080-operations-2014.pdf> (accessed February 2020).

Parkes JP, Nugent G, Forsyth DM, Byrom AE, Pech RP, Warburton B, Choquenot D. 2017. Past, present and two potential futures for managing New Zealand's mammalian pests. New Zealand Journal of Ecology **41**:151-161.

Russell JC, Innes JG, Brown PH, Byrom AE. 2015. Predator-Free New Zealand: Conservation Country. Bioscience **65**:520-525.

Sanctuaries NZ. 2019. Available from <http://www.sanctuariesnz.org/> (accessed February 2020).

Saunders A, Norton DA. 2001. Ecological restoration at Mainland Islands in New Zealand. Biological Conservation **99**:109-119.
